# Systematics of the family Plectopylidae in Vietnam with additional information on Chinese taxa (Gastropoda, Pulmonata, Stylommatophora)

**DOI:** 10.3897/zookeys.473.8659

**Published:** 2015-01-20

**Authors:** Barna Páll-Gergely, András Hunyadi, Jonathan Ablett, Hào Văn Lương, Fred Naggs, Takahiro Asami

**Affiliations:** 1 Department of Biology, Shinshu University, Matsumoto 390-8621, Japan; 2 Adria sétány 10G 2/5., Budapest 1148, Hungary; 3 Department of Zoology, The Natural History Museum, London SW7 5BD, United Kingdom; 4 Center for Rescuing and Conservation of Organisms, Hoang Lien National Park, No. 123, Nguyen Chi Thanh Road, Sa Pa, Lao Cai, Vietnam

**Keywords:** Anatomy, revision, taxonomy, new species, Plectopylidae, Corillidae, mating behaviour, Vietnam, China

## Abstract

Vietnamese species from the family Plectopylidae are revised based on the type specimens of all known taxa, more than 600 historical non-type museum lots, and almost 200 newly-collected samples. Altogether more than 7000 specimens were investigated. The revision has revealed that species diversity of the Vietnamese Plectopylidae was previously overestimated. Overall, thirteen species names (*anterides* Gude, 1909, *bavayi* Gude, 1901, *congesta* Gude, 1898, *fallax* Gude, 1909, *gouldingi* Gude, 1909, *hirsuta* Möllendorff, 1901, *jovia* Mabille, 1887, *moellendorffi* Gude, 1901, *persimilis* Gude, 1901, *pilsbryana* Gude, 1901, *soror* Gude, 1908, *tenuis* Gude, 1901, *verecunda* Gude, 1909) were synonymised with other species. In addition to these, *Gudeodiscus hemmeni* **sp. n.** and *Gudeodiscus messageri raheemi* **ssp. n.** are described from north-western Vietnam. Sixteen species and two subspecies are recognized from Vietnam. The reproductive anatomy of eight taxa is described. Based on anatomical information, *Halongella* **gen. n.** is erected to include *Plectopylis schlumbergeri* and *Plectopylis fruhstorferi*. Additionally, the genus *Gudeodiscus* is subdivided into two subgenera (*Gudeodiscus* and *Veludiscus* **subgen. n.**) on the basis of the morphology of the reproductive anatomy and the radula. The Chinese *Gudeodiscus phlyarius werneri* Páll-Gergely, 2013 is moved to synonymy of *Gudeodiscus phlyarius*. A spermatophore was found in the organ situated next to the gametolytic sac in one specimen. This suggests that this organ in the Plectopylidae is a diverticulum. Statistically significant evidence is presented for the presence of calcareous hook-like granules inside the penis being associated with the absence of embryos in the uterus in four genera. This suggests that these probably play a role in mating periods before disappearing when embryos develop. *Sicradiscus mansuyi* is reported from China for the first time.

## Introduction

At present, 477 species and subspecies in 22 families of terrestrial pulmonates are known from Vietnam (Schileyko 2011). As in other southeast Asian gastropods, most of these (77%) were described between 1880 and 1920 with poor locality data and based on shell characters only. Several species were described by examining only a single shell. Internal anatomy and exact collecting locality have been documented only for a few taxa. Accordingly, the systematics of most Vietnamese land snails remains questionable. Without accurate knowledge on their distribution and taxonomy, the recognition of possible threats and the subsequent establishment of appropriate conservation measures of these populations are impossible.

The Plectopylidae are currently the fifth largest pulmonate family in Vietnam with 28 species, after the Camaenidae s.l. (=Camaenidae and Bradybaenidae: 127 sp., 9 ssp.), Clausiliidae (84 sp., 10 ssp.), Ariophantidae (68 sp., 3 ssp.) and Streptaxidae (47 sp., 2 ssp.) (Schileyko 2011). The Plectopylidae are a group of medium sized (5–35 mm), usually flat, sinistral or dextral species, which have internal lamellae and plicae on both the palatal and parietal walls. This family is currently included within the Plectopyloidea together with the south Asian family Corillidae Pilsbry, 1905 and the south-west family African Sculptariidae Degner, 1923 (Bouchet and Rocroi 2005). However, Schileyko (1999) classified Sculptariidae in the superfamily Acavoidea. Plectopylidae differ from the probably closest group, which are the Corillidae with one or two vertical lamellae on the parietal wall. The mainly Sri Lankan Corillidae only have horizontal plicae. Plectopylidae have a wide distribution from northeastern India through the majority of southeast Asia to Peninsular Malaysia, northern Vietnam and southern Japan (Páll-Gergely and Hunyadi 2013 and references therein).

Morlet (*schlumbergeri*; 1886a, 1886b) was the first to describe a Vietnamese plectopylid species. Mabille (*jovia* and *phlyaria*; 1887a, 1887b), Ancey (*villedaryi*; 1888), Fischer (*giardi* and *francoisi*: 1898b, 1898a) and Möllendorff (*choanomphala*, *emigrans*, *fruhstorferi*, *hirsuta*: 1901) followed. Gude (1897b, 1899a, 1899b, 1899c, 1899d, 1900, 1901a, 1901b, 1901c, 1908, 1909) described new species, revised the taxa and published drawings of every species that had not been previously figured. He received most of the shell material from French collectors, mainly from Messager and Mansuy.

Gude (1899c) proposed the subgeneric division of *Plectopylis* (equivalent to the current Plectopylidae) by erecting seven “sections” (subgenera) within *Plectopylis*: *Endothyra* (replaced by *Endothyrella* by Zilch 1960), *Chersaecia*, *Endoplon*, *Plectopylis*, *Sinicola*, *Enteroplax* and *Sykesia* Gude, 1897a. The last two have been removed from the Plectopylidae; *Enteroplax* has been placed in the Strobilopsidae (Zilch 1959; Solem 1968; Schileyko 1998). Gude (1914) and Schileyko (2001) assigned *Ruthvenia* Gude, 1911 (nomen novum pro *Sykesia*) to the Endodontidae and Schileyko (2010) and Raheem et al. (2014) to the Charopidae. Gude’s (1899c) subdivision was primarily based on the morphology of palatal plicae, the direction of coiling and the depth of the umbilicus.

According to Gude (1899c), only the “section” *Endoplon* Gude, 1899 occurs in Vietnam. Gude also placed two Burmese (Myanmar) species (including the type species, *Helix brachyplecta* Benson, 1863) in *Endoplon*. Some Vietnamese species were subsequently placed in the subgenus *Sinicola* (Möllendorff 1901, Gude 1908). However, in some species descriptions Gude did not specify subgenera (Gude 1901a, 1908, 1909) or these were mentioned only within the text (Gude 1908). He mentioned *Plectopylis tenuis* as the connection between *Sinicola* and *Endoplon* (1908). Schileyko (1999) elevated Gude’s (1899c) “sections” (*Endothyrella*, *Chersaecia*, *Endoplon*, *Plectopylis*, *Sinicola*) to genera. Schileyko (1999) followed Yen (1939) and Zilch (1960) in placing the Chinese genus *Amphicoelina* Haas, 1933 within the Plectopylidae but Páll-Gergely and Asami (2014) classified *Amphicoelina* within the Camaenidae, as originally proposed by Haas (1933).

After Gude’s publications, virtually no taxonomic information was published on Vietnamese members of the family. Jaeckel (1950) reported two juvenile shells of “*Plectopylis laminifera*” from the debris of an unknown Tonkinese (northern Vietnamese) river. Páll-Gergely and Hunyadi (2013) concluded that juvenile shells of *Sinicola jugatoria* (Ancey, 1885) (synonym: *laminifera*) cannot be distinguished from congeners, that their distribution in China (northern Chongqing, eastern Hubei and Guizhou provinces) lies far from Vietnam, and that it probably does not occur within the country.

Revision of the Chinese Plectopylidae (Páll-Gergely and Hunyadi 2013) also revealed that the two recorded Burmese species of *Endoplon* show considerable differences from Vietnamese species. Vietnamese species have regularly ribbed embryonic whorls and no long horizontal parietal plicae, whereas the Burmese species possess a comparatively smooth protoconch and long horizontal parietal plica. Because the type species of *Endoplon* is one of the Burmese species, all the former Vietnamese *Endoplon* species were moved to a new genus, *Gudeodiscus* Páll-Gergely, 2013. The two Burmese *Endoplon* species are probably closely related to *Plectopylis* and *Chersaecia* species, which inhabit similar geographic regions (Myanmar, northern Thailand and north-eastern India) (Páll-Gergely and Hunyadi 2013).

The genus *Sinicola* Gude, 1899 (with the type species *Helix fimbriosa* von Martens, 1875) differs from *Gudeodiscus* mainly in the keeled body whorl (rounded in *Gudeodiscus*) and the presence of deciduous periostracal folds in most species (always absent in *Gudeodiscus*). Former Vietnamese *Sinicola* species (*emigrans*, *fruhstorferi*, *soror* and *suprafilaris*) were all classified within *Gudeodiscus* by Páll-Gergely and Hunyadi (2013). So far, *Sinicola* species have only been found to inhabit Chinese provinces (Chongqing, northern Guangxi, Guizhou, Hubei, Hunan and Sichuan). The third genus, *Sicradiscus* Páll-Gergely, 2013 (Type species. *Plectopylis schistoptychia* Möllendorff, 1886) was established for some small bodied species. *Sicradiscus* consists of two species groups. One has a rounded body whorl and a strong apertural fold: *Sicradiscus invius* (Heude, 1885), *feheri* Páll-Gergely & Hunyadi, 2013, *Sicradiscus mansuyi* (Gude, 1908) (only Vietnamese species of the genus) and *Sicradiscus securus* (Heude, 1885). The other species group possesses a moderately shouldered body whorl and lacks an apertural fold: *Sicradiscus cutisculptus* (Möllendorff, 1882), *Sicradiscus diptychia* (Möllendorff, 1885), *Sicradiscus hirasei* (Pilsbry, 1904), *Sicradiscus ishizakii* (Kuroda, 1941) and *Sicradiscus schistoptychia*. *Sicradiscus transitus* Páll-Gergely, 2013 with an apertural fold and a shouldered body whorl connects these two species groups. All *Sicradiscus* species differ from *Sinicola* by the presence of the anterior lamella. The rounded *Sicradiscus* differs from *Gudeodiscus* by the small size, strong apertural fold connected to the callus and the smooth ventral surface.

For the present revision of the Vietnamese Plectopylidae, we examined all the type specimens as well as many available non-type material deposited in public institutions. All samples deposited in HNHM, NHMSB, NHM, MNHN, NHMW, SMF and SNM were investigated. Some “problematic” samples were loaned and identified from RBINS and USNM. Material (usually with GPS data) obtained from the following private collections were investigated: András Hunyadi, Jozef Grego, Christa and Jens Hemmen, Kenji Ohara, Jamen Uiriamu Otani and Wim Maassen. Altogether approximately two hundred samples with exact locality data were examined. Fischer and Dautzenberg (1904) mentioned two names (*Plectopylis anoplon* and *simulans*) from Vietnam but presented no formal descriptions. Although listed by Thanh (2008), these nomen nuda cannot be assigned to species. Gude’s material is deposited in NHM, and most samples from Lieutenant Colonel Messager are housed in MNHN. Messager probably sent only a few shells to Gude, who published on these in 1909. The six species described by Gude (1909) are problematic. Investigation of these specimens including Messager’s original material allowed us to gain a better understanding of species boundaries based on morphological gaps in continuously varying shell characters.

Here we present the outcome of systematic revision of Vietnamese Plectopylidae (see summary in Table 1) with reproductive anatomy and radula morphology of eight species. Additionally, we publish information on the radula of fifteen Chinese species. The genus *Gudeodiscus* is divided into two subgenera based on anatomical and radula information of Chinese and Vietnamese species.

**Table 1.** (Sub)generic division of Vietnamese Plectopylidae in Gude’s (1899c) revision, in the original description (in case of species described after Gude 1899c), and in this study. Synonymies are also indicated. Valid taxa with **bold italic**.

(sub)species	section in Gude 1899c	(sub)genus in the original publication	This study	synonym of
***anceyi*** Gude, 1901		not specified	Gudeodiscus (Gudeodiscus?)	
*anterides* Gude, 1909		not specified		*phlyarius*
*bavayi* Gude, 1901		not specified		*francoisi*
*choanomphala* Möllendorff, 1901		*Endoplon*		*villedaryi*
*congesta* Gude, 1899	*Endoplon*			*giardi*
***cyrtochila*** Gude, 1909		not specified	Gudeodiscus (Gudeodiscus?)	
***dautzenbergi*** Gude, 1901		not specified	Gudeodiscus (Gudeodiscus)	
***emigrans*** Möllendorff, 1901		*Sinicola*	Gudeodiscus (Veludiscus)	
*fallax* Gude, 1909		not specified		*phlyarius*
***fischeri*** Gude, 1901		not specified	Gudeodiscus (Gudeodiscus)	
***francoisi*** Fischer, 1898	*Endoplon*		Gudeodiscus (Gudeodiscus?)	
***fruhstorferi*** Möllendorff, 1901		*Sinicola*	*Halongella*	
***giardi*** Fischer, 1898	*Endoplon*		Gudeodiscus (Gudeodiscus)	
*gouldingi* Gude, 1909		not specified		*phlyarius*
*hirsuta* Möllendorff, 1901		*Endoplon*		*schlumbergeri*
***infralevis*** Gude, 1908		not specified	Gudeodiscus (Gudeodiscus?)	
*jovia* Mabille, 1887	*Endoplon*			*schlumbergeri*
*lepida* Gude, 1900		not specified		*francoisi*
***mansuyi*** Gude, 1908		not specified	*Sicradiscus*	
***messageri*** Gude, 1909		not specified	Gudeodiscus (Gudeodiscus)	
*moellendorffi* Gude, 1901	*Endoplon*			*phlyarius*
*persimilis* Gude, 1901		not specified		*dautzenbergi*
***phlyarius*** Mabille, 1887	*Endoplon*		Gudeodiscus (Gudeodiscus)	
*pilsbryana* Gude, 1901 (new name for *villedaryi*)		not specified		*schlumbergeri*
***quadrilamellatus*** Páll-Gergely, 2013		*Gudeodiscus*	Gudeodiscus (Veludiscus)	
***schlumbergeri*** Morlet, 1886	*Endoplon*		*Halongella*	
*soror* Gude, 1908		*Sinicola*		*infralevis*
***suprafilaris*** Gude, 1908		*Sinicola*	Gudeodiscus (Gudeodiscus?)	
*tenuis* Gude, 1901		not specified		*fischeri*
*verecunda* Gude, 1909		not specified		*phlyarius*
***villedaryi*** Ancey, 1888	*Endoplon*		Gudeodiscus (Gudeodiscus)	

## Materials and methods

Shell whorls (exactness 0.25) were counted according to Kerney and Cameron (1979: 13). Differences in size are indicated in the diagnosis using the following terms: very small (6–10 mm), small (10–15 mm), medium-sized (15–20 mm), large (20–25 mm), very large (25–30 mm).

**Figure 1.**
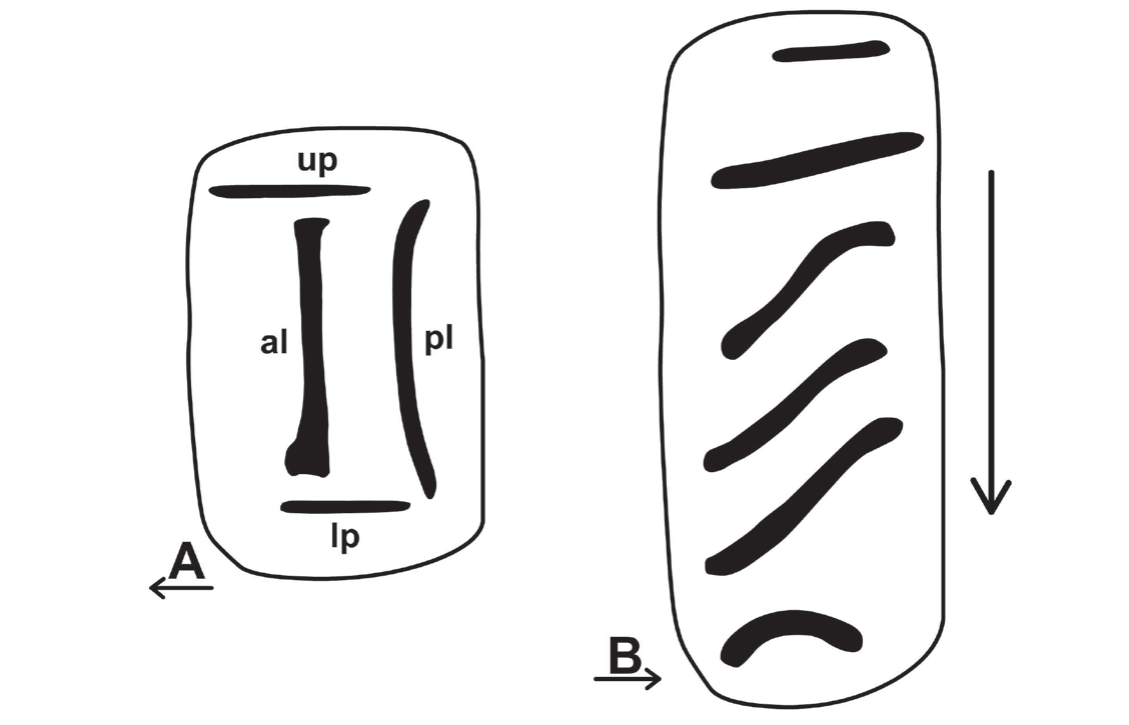
Nomenclature of parietal (**A**) and palatal (**B**) plicae and lamellae. Small arrows under the letters show the direction of the aperture. Large arrow next to figure B shows the direction of counting of palatal plicae (first above, last below). Abbreviations: al: anterior lamella; lp: lower plica; pl: posterior lamella; up: upper plica.

The palatal plicae can be observed from the interior and exterior view. This is indicated in the figure captions in all cases. If enough shell material was available, a shell fragment with the palatal plicae was broken out and the lamellae were observed directly (interior view). If shell material was limited, the plicae are figured as they were visible through the shell wall (external view). For nomenclature of lamellae (vertical parietal folds) and plicae (horizontal parietal folds and palatal folds) see Figure 1.

Examined specimens for each taxon are separately listed as types, museum material and new material. Most specimens in the last category are geo-referenced whereas precise localities are unknown for the majority of older museum material. The original code of locality is indicated before the locality of newly collected material. Certain populations are referred to by using these codes, and the inventory numbers in case of museum material, for example in the measurements and species remarks. In the distribution maps, localities which are closer to each other than 2 km were indicated with a single plot to make the map easier to understand. Chinese localities published by Páll-Gergely and Hunyadi (2013) are also indicated on the maps. The distances between parapatric populations (Table 2) were measured using Google Earth.

**Table 2.** Co-occurrence of Vietnamese Plectopylidae. Three stars indicate co-occurrence observed with newly-collected materials, which were collected by the same collector in each strict sympatry. Two stars indicate that the two species were collected at geographically close sites by the same or different collectors (*anceyi*-*fischeri*: 940 m; *dautzenbergi*-cf. *phlyarius*: 1160 m; *anceyi*-*suprafilaris*: 2340 m; *fischeri*-*emigrans quadrilamellatus*: 4650 m; *francoisi*-*phlyarius*: 85 m; *francoisi*-*suprafilaris*: 290 m; *giardi*-*phlyarius*: 350 m; *phlyarius*-*suprafilaris*: 370 m; *phlyarius*-*mansuyi*: 350 m). One star indicates frequent presence of the two species mixed within museum samples.

	***anceyi***	***fischeri***	***francoisi***	***giardi***	***phlyarius* (*gouldingi*/*fallax*)**	***phlyarius***	***hemmeni* sp. n.**	***mansuyi***
***cyrtochilus***								***
***dautzenbergi***						**		
***fischeri***	**							
***francoisi***	***							
***giardi***	***		***					***
***messageri***					*			
***messageri raheemi* ssp. n.**						***	***	
***phlyarius***	***		**	**				**
***emigrans quadrilamellatus***	***	**						
***suprafilaris***	**		**	***		**		***
***villedaryi***	***					***		

Ethanol-preserved specimens were dissected under a Leica stereomicroscope, with camera attached to provide photographs of the genital structure from which drawings were produced. In description of the reproductive system, we used the terms “distal” and “proximal” in relation to the genital atrium. At dissection of each specimen, we recorded whether embryos are present in the uterus and calcareous granules on the internal surface of penis (Table 3). Fisher’s exact test was used to examine the association of the presence of embryo and the absence of granules by treating all the examined individuals as replicates across the four genera because of limited sample sizes in each genus (Tables 4–5).

**Table 3.** Association between the presence of calcareous granules in the penis and embryos in the uterus in the genera of “Eastern Plectopylidae”. Source of information: 1: this study, 2: Páll-Gergely and Hunyadi (2013), 3: Páll-Gergely and Asami (2014).

Name	source	Country, province	elevation (m)	date	embryos	shape of granules	No. of specimens	Notes
*Gudeodiscus emigrans otanii*	3	China, Guangxi	180	November 13	present	no granules	2	the third specimen was aphallic
*Gudeodiscus eroessi eroessi*	3	China, Guangxi	153	November 9	present	no granules	2	
*Gudeodiscus fischeri*	1	Vietnam, Tuyên Quang	70	March 19	absent	hook-like	1	
*Gudeodiscus fischeri*	1	Vietnam, Bắc Kạn	335	November 19	present	no granules	1	
*Gudeodiscus giardi giardi*	1	Vietnam, Cao Bằng	430	November 16	absent	hook-like	1	
*Gudeodiscus giardi giardi*	3	China, Guangxi	308	January 10	absent	flat, oval	1	
*Gudeodiscus messageri raheemi*	1	Vietnam, Hòa Bình	1120	October 15	present	no granules	1	
*Gudeodiscus multispira*	2	China, Guangxi	160	October 14	present	no granules	3	
*Gudeodiscus multispira*	3	China, Guangxi	252	November 12	present	no granules	1	
*Gudeodiscus okuboi*, specimen1	3	China, Guangxi	131	November 9	present	no granules	1	
*Gudeodiscus okuboi*, specimen2	3	China, Guangxi	131	November 9	absent	no granules	1	
*Gudeodiscus phlyarius*	1	Vietnam, Lạng Sơn	370	April 1	present	no granules	1	
*Gudeodiscus phlyarius*	2	China, Guangxi	190	October 11	absent	hook-like	1	
*Gudeodiscus phlyarius*	2	China, Guangxi	360	October 23	present	no granules	1	
*Gudeodiscus phlyarius* (“*fallax*”)	1	Vietnam, Lào Cai	270	October 4	absent	flat, oval	2	
*Gudeodiscus pulvinaris pulvinaris*	3	Hong Kong	300-500	June	absent	hook-like	1	
*Gudeodiscus pulvinaris robustus*	2	China, Guangxi	140	October 17	present	no granules	1	
*Gudeodiscus villedaryi*	1	Vietnam, Thái Nguyên	365	May 20	present	no granules	1	
*Gudeodiscus villedaryi*	1	Vietnam, Thái Nguyên	365	November 12	absent	hook-like	1	
*Halongella fruhstorferi*	1	Vietnam, Quảng Ninh	20	August 14	present	very thin, flat, no particular shape	1	
*Halongella schlumbergeri*	1	Vietnam, Hải Phòng	20	April 4	present	flat, thin, with no particular shape	1	
*Halongella schlumbergeri*	1	Vietnam, Hải Phòng	30	November 22	absent	flat, thin, with no particular shape, or T-shaped	1	
*Sicradiscus invius*	3	China, Sichuan	1087	September 17	absent	no granules	2	
*Sicradiscus mansuyi*	1	Vietnam, Cao Bằng	570	May 28	present	no granules	2	subadult
*Sicradiscus schistoptychia*	2,3	China, Hunan	450	November 11	present	tiny flat rounded granules	1	
*Sicradiscus transitus*	3	China, Guangxi	650	September 12	absent	minute, flat, rounded	1	subadult
*Sinicola asamiana*	3	China, Sichuan	860	September 16	present	no granules	1	
*Sinicola emoriens*	2,3	China, Guangxi	125	November 8	present	no granules	2	
*Sinicola fimbriosa*	2	China, Hunan	590	October 20	absent	no granules	1	subadult
*Sinicola murata*	3	China, Sichuan	860	September 16	present	no granules	1	
*Sinicola murata*	3	China, Sichuan	1090	September 17	present	no granules	1	
*Sinicola reserata azona*	3	China, Guizhou	863	May 10	present	no granules	1	
*Sinicola stenochila*	3	China, Hubei	220	November 3	absent	globular or elongated	2	

**Table 4.** Association of the presence of embryo and the absence of granules within the genus *Gudeodiscus*.

		embryo		Probability
		present	absent	
**granule**	**present**	0	7	0.0001
	**absent**	12	1	

**Table 5.** Association of the presence of embryo and the absence of granules within all four genera (*Gudeodiscus*, *Halongella* gen. n., *Sicradiscus*, *Sinicola*).

		embryo		Probability
		present	absent	
**granule**	**present**	3	10	0.0006
	**absent**	18	3	

To demonstrate the continuous variation of shell heights and diameters across *Plectopylis anterides*/*gouldingi*, *Plectopylis fallax*, and Plectopylis fallax var. major specimens (synonyms of *Gudeodiscus phlyarius*; Figure 16), we randomly selected a few samples which can be assigned to those taxa.

The buccal mass was removed and soaked in 2 molar KOH solution for 5 hours before extracting the radula, which was preserved in 70% ethanol. Radulae were directly observed without coating under a low vacuum SEM (Miniscope TM-1000, Hitachi High-Technologies, Tokyo).

### Taxonomic treatment

This revision is based on morphology by examination of specimens and literature. Thus the present taxa are defined based on their morphological differences. The present species are hypothesized as species defined by the biological species concept (Mayr 1942), although evidence for differences in sympatry was not always available within the relevant species group. Table 2 shows sympatric species pairs. No specimens were found that show transitional characters between sympatric species. This suggests that these are biological species reproductively isolated from each other.

Previously recognized taxa are synonymized when their differences between traditionally recognized species (often present as only a few individuals) are considered to be very minor. Sometimes, these differences (mainly in the morphology of the plicae and lamellae) show a geographical pattern. If these minor differences fall within the range of the species’ morphological diversity, the taxa are synonymized.

### Abbreviations

HA Collection András Hunyadi (Budapest, Hungary);

HE Collection Hemmen (Wiesbaden, Germany);

HNHM Magyar Természettudományi Múzeum (Budapest, Hungary);

JG Collection Jozef Grego (Banská Bystrica, Slovakia);

NHMSB Natural History Museum, Sibiu (Romania), Bielz collection;

NHM & NHMUK Natural History Museum, London;

MNHN Muséum National d’Histoire Naturelle (Paris, France);

NHMW Naturhistorisches Museum Wien (Vienna, Austria);

OK Collection Kenji Ohara, Nishinomiya Shell Museum (Nishinomiya, Japan);

PGB Collection Barna Páll-Gergely (Mosonmagyaróvár, Hungary);

RBINS Royal Belgian Institute of Natural Sciences (Brussels, Belgium);

SMF Senckenberg Forschungsinstitut und Naturmuseum (Frankfurt am Main, Germany);

USNM Smithsonian National Museum of Natural History (Washington, USA);

VA Collection András Varga (Gyöngyöshalász, Hungary);

WM Collection Wim J. M. Maassen (Echt, The Netherlands);

ZMUC Zoological Museum, University of Copenhagen (Denmark);

coll collection of

jb juvenile/broken shells

leg collected by

ex from the collection of

D shell diameter

H shell height

## Results

### Radula information

Information on the radula morphology of Chinese Plectopylidae species has never been published. To provide a comprehensive basis of the radula morphology of Vietnamese species, we publish images of the radula of some Chinese species as well. The key characters of the radula (size of the central tooth in relation to the ectocone of the first lateral, the shape of the mesocone of the first lateral and the morphology of the marginals) are compiled in Table 6.

**Table 6.** Key characters of the radula of Chinese and Vietnamese Plectopylidae species. Abbreviations: **L** lateral **M** marginal.

taxon	L	M	size of central	shape of the first lateral	morphology of the marginals
Gudeodiscus (Veludiscus) emigrans otanii	9	11	slightly smaller than the ectocone of the first lateral	rhomboid, rather blunt	bicuspid or tricuspid with blunt inner cusp and shallow incision between the inner two cusps
Gudeodiscus (Veludiscus) eroessi	10	10	smaller than the ectocone of the first lateral	rhomboid, rather blunt	bicuspid or tricuspid with blunt inner cusp and shallow incision between the inner two cusps
Gudeodiscus (Veludiscus) okuboi	7	13	slightly smaller than the ectocone of the first lateral	rhomboid, rather blunt	tricuspid with blunt inner cusp and shallow incision between the inner two cusps
Gudeodiscus (Veludiscus) pulvinaris pulvinaris	7	14	smaller than the ectocone of the first lateral	rhomboid, rather blunt	tricuspid with blunt inner cusp and shallow incision between the inner two cusps
Gudeodiscus (Gudeodiscus) fischeri	9	13	as large as or larger than the ectocone of the first lateral	rhomboid, pointed	tricuspid, inner two rather blunt, incision between them deep
Gudeodiscus (Gudeodiscus) giardi	12	15	as large as the ectocone of the first lateral	rhomboid, pointed	bicuspid or tricuspid with blunt inner cusp and shallow incision between the inner two cusps
Gudeodiscus (Gudeodiscus) messageri raheemi	8	16	as large as or larger than the ectocone of the first lateral	rhomboid, pointed	tricuspid with rather sharp inner cusp and deep incision between the cusps
Gudeodiscus (Gudeodiscus) multispira	9	14	as large as or larger than the ectocone of the first lateral	slender oval	tricuspid with rather blunt inner cusp and deep incision between the cusps
Gudeodiscus (Gudeodiscus) phlyarius	9	12	as large as the ectocone of the first lateral	rhomboid, pointed	bicuspid or tricuspid with blunt inner cusp and shallow incision between the inner two cusps
Gudeodiscus (Gudeodiscus) villedaryi	9	10	as large as or slightly smaller than the ectocone of the first lateral	rhomboid, pointed	bicuspid or tricuspid with blunt inner cusp and shallow incision between the inner two cusps
*Halongella fruhstorferi*	8	12	much smaller than the ectocone of the first lateral	slender rhomboid	mostly bicuspid, some of them tricuspid with blunt inner cusps
*Halongella schlumbergeri*	10	14	smaller than the ectocone of the first lateral	oval	bicuspid or tricuspid with blunt inner cusp and shallow incision between the inner two cusps
*Sicradiscus invius*	7	8	as large as or larger than the ectocone of the first lateral	slender with parallel, straight margins and pointed end	tricuspid with pointed cusps and deep incision between them
*Sicradiscus mansuyi*	8	10	as large as the ectocone of the first lateral	slender with parallel, straight margins and pointed end	tricuspid with pointed cusps and deep incision between them, some of them quadricuspid
*Sicradiscus schistoptychia*	6	14	as large as or larger than the ectocone of the first lateral	slender with parallel, straight margins and pointed end	tricuspid with pointed cusps and deep incision between them
*Sicradiscus transitus*	6	10	as large as the ectocone of the first lateral	triangular	tricuspid with pointed cusps and deep incision between them
*Sinicola asamiana*	8	11	as large or almost as large as the ectocone of the first lateral	slender with parallel, straight margins and pointed end	tricuspid with pointed cusps and deep incision between them
*Sinicola emoriens*	6	14	as large as or larger than the ectocone of the first lateral	slender with parallel, straight margins and pointed end	tricuspid with pointed cusps and deep incision between them
*Sinicola fimbriosa*	10	15	larger than the ectocone of the first lateral	slender with concave inner line	tricuspid with pointed cusps and deep incision between them
*Sinicola jugatoria*	9	12	as large as the ectocone of the first lateral	slender with parallel, straight margins and pointed end	tricuspid with pointed cusps and deep incision between them
*Sinicola murata*	8	12	as large as the ectocone of the first lateral	slender with parallel, straight margins and pointed end	tricuspid with pointed cusps and deep incision between them
*Sinicola reserata azona*	11	14	as large as the ectocone of the first lateral	slender with parallel, straight margins and pointed end	tricuspid with pointed cusps and deep incision between them
*Sinicola stenochila*	8	13	as large as the ectocone of the first lateral	slender with parallel, straight margins and pointed end	tricuspid with pointed cusps and deep incision between them

The overall morphology of the radula was similar in all species. The lateral teeth are arranged along straight rows, whereas the marginals stand in oblique rows. The distinction between the last laterals and the first marginals is not easy, especially in those specimens in which their morphology (bi- or tricuspid) does not differ. Therefore, the data on the number of laterals and marginals are only guidelines.

### Systematic treatment Family Plectopylidae Möllendorff, 1898

#### Genus Gudeodiscus

Taxon classificationAnimaliaStylommatophoraPlectopylidae

Páll-Gergely, 2013

Gudeodiscus Páll-Gergely in Páll-Gergely & Hunyadi 2013, Archiv für Molluskenkunde, 142 (1): 4, 8.

##### Type species.

*Plectopylis phlyaria* Mabille, 1887, by original designation.

##### Included taxa.

Subgenus *Gudeodiscus* and subgenus *Veludiscus* subgen. n.

##### Diagnosis.

Shell rarely small, usually middle sized or large, dextral, body whorl rounded, without periostracal folds on the “upper keel” of the whorls. The whole protoconch is usually very finely, regularly ribbed (see Figure 10A). The only known exceptions are *Gudeodiscus villedaryi* (see Figure 10B) and *Gudeodiscus dautzenbergi*. Teleoconch usually has a reticulated sculpture; more prominent on the dorsal side; sometimes with very small periostracal filaments, but these are always arranged radially, never in spiral lines. A short apertural fold is present in the majority of the species. Palatal plicae usually 6, sometimes 5 or 7, they are usually free, very rarely connected by a ridge. Middle palatal plicae can be horizontal, oblique or almost vertical, they are usually depressed “Z” or “V”-shaped. The first plica is always straight and parallel with the suture, the last is slightly curved or oblique. On the parietal wall there are two vertical lamellae or the anterior one is missing or dissolved into small denticles or parallel horizontal plicae. Usually horizontal plicae are visible above and below the anterior lamella, near the sutures.

Penial caecum usually present (very rarely absent). Penis internally with longitudinal folds; the middle or proximal portion of the penis can have transverse or reticulated sculpture; the longitudinal folds are thickened on the apical part of the penis and form “pockets”, each of which holds a calcareous, usually hook- or claw-like translucent granule; these granules are probably present seasonally when the snails are reproductively active and disappear when embryos develop in the uterus; the pockets stand in one row or rarely in two rows on the opened penis wall. Epiphallus with simple internal longitudinal folds.

##### Differential diagnosis.

The body whorl of the species belonging to *Sinicola* is keeled or shouldered, often with flat, deciduous periostracal folds arranged in one row on the keel. In contrast, all *Gudeodiscus* species have rounded body whorl and never have periostracal folds arranged in a spiral line. Moreover, in *Sinicola* there are no “pockets” on the inner wall of the penis. The shells of *Halongella* gen. n. are indistinguishable from those of *Gudeodiscus*. *Halongella* gen. n. species have parallel, longitudinal folds on the inner wall of the penis with tiny, flat calcareous granules between the folds, all along the penis; there are no determined “pockets” for the granules at the apical part of the penis, which are so characteristic for *Gudeodiscus*. Additionally, the longitudinal folds inside the epiphallus of *Halongella* gen. n. species have characteristic transverse projections which overlap with those of neighbouring folds. In contrast, *Gudeodiscus* species have parallel folds on the inner wall of the epiphallus. Additionally, most anatomically examined *Gudeodiscus* specimens had a penial caecum, which is missing in both *Halongella* gen. n. species. See also under *Sicradiscus*.

#### Subgenus Gudeodiscus

Taxon classificationAnimaliaStylommatophoraPlectopylidae

Páll-Gergely, 2013

##### Diagnosis.

Shell indistinguishable from Gudeodiscus (Veludiscus) subgen. n. Anatomy: The epiphallus has a somewhat thickened proximal part; retractor muscle simple, inserts on the distal end of the penial caecum, or if it is missing, than on the distal end of the penis (at the penis-epiphallus transition). Radula: central tooth usually as large as or slightly larger than the ectocone of the first lateral; mesocone of the first lateral is moderately wide, in most cases has parallel edges. Marginals usually tricuspid with rather pointed inner cusp and rather deep incision between the inner two cusps.

##### Included taxa.

*anceyi* (Gude, 1901)(?), *concavus* Páll-Gergely, 2013(?), *cyrtochilus* (Gude, 1909)(?), *dautzenbergi* (Gude, 1901), *fischeri* (Gude, 1901), *francoisi* (Fischer, 1899)(?), *giardi* (Fischer, 1898), *hemmeni* Páll-Gergely & Hunyadi, sp. n.(?), *infralevis* (Gude, 1908)(?), *marmoreus* Páll-Gergely, 2014(?), *messageri* (Gude, 1909), *multispira* (Möllendorff, 1883), *phlyarius* (Mabille, 1887), *soosi* Páll-Gergely, 2013(?), *suprafilaris* (Gude, 1908)(?), *ursula* Páll-Gergely & Hunyadi, 2013(?), *villedaryi* (Ancey, 1888), *yanghaoi* Páll-Gergely & Hunyadi, 2013(?), *yunnanensis* Páll-Gergely, 2013(?).

##### Remarks.

All known *Gudeodiscus* species remain in this subgenus with the exception of *Gudeodiscus goliath* Páll-Gergely & Hunyadi, 2013 because of its similar shell and distribution area to *Gudeodiscus pulvinaris robustus* Páll-Gergely & Hunyadi, 2013 and *Gudeodiscus emigrans otanii* Páll-Gergely & Hunyadi, 2013. Those with unknown anatomy and radula morphology have questionable subgeneric assessment. The shell of *Gudeodiscus dautzenbergi* is very similar to the nearby occurring *Gudeodiscus villedaryi*, therefore we think there is no need to question the subgeneric status.

#### Gudeodiscus (Gudeodiscus?) anceyi

Taxon classificationAnimaliaStylommatophoraPlectopylidae

(Gude, 1901)

**Figure 2.**
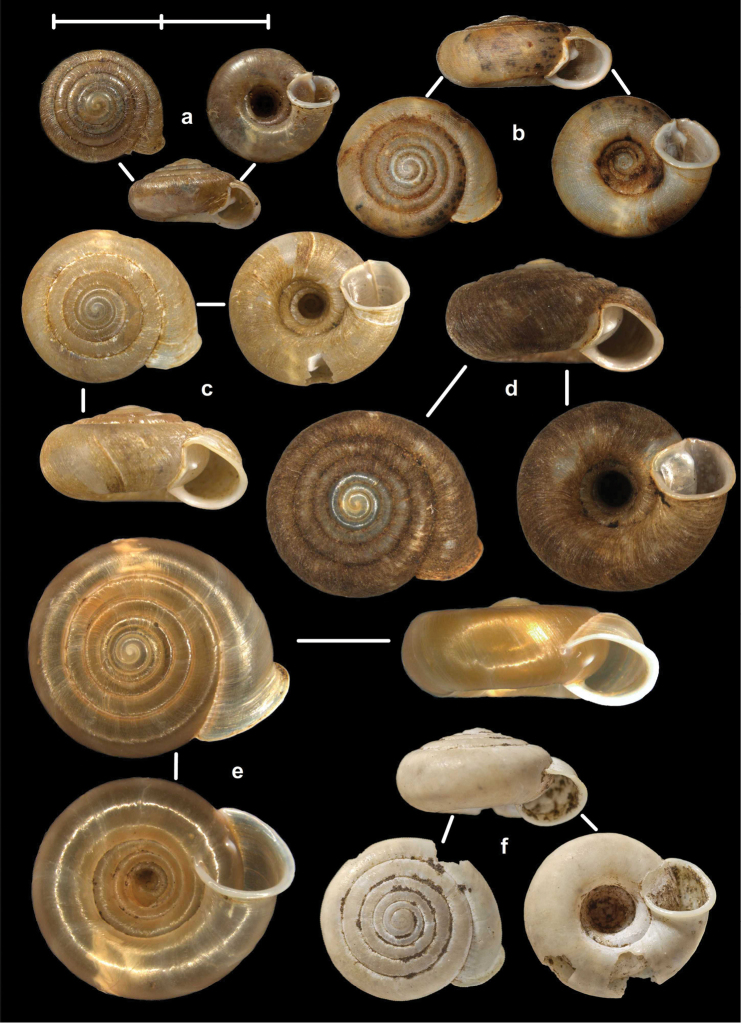
Shells of Vietnamese *Sicradiscus* and *Gudeodiscus* species. **A** *Sicradiscus mansuyi* (Gude, 1908), NHMUK 1907.2.20.19 (syntype) **B** Gudeodiscus (Gudeodiscus?) anceyi (Gude, 1901), Tonkin, Bac-Kan, leg. Messager, MNHN 24600 (syntype) **C** Gudeodiscus (Gudeodiscus?) hemmeni Páll-Gergely & Hunyadi, sp. n., 2012/61, HNHM 97458 (holotype) **D** Gudeodiscus (Gudeodiscus?) hemmeni, Vn10-103 **E** Gudeodiscus (Gudeodiscus) fischeri (Gude, 1901), 20090519B, coll. PGB **F** Gudeodiscus (Gudeodiscus?) cyrtochilus (Gude, 1909), NHMUK 1922.8.29.59. (syntype). Photos: H. Taylor (**A, F**), T. Deli (**B**) and B. Páll-Gergely (**C, D, E**). Scale represents 10 mm.

Figures 2B , 9G , 11C–F

Plectopylis Anceyi Gude 1901a, Journal de Conchyliologie, 49: 208–209., Figs 6a–e, Plate 6, Figs 6a–c. [“Bac-Kan (le type); secteur de Nac-Ri; entre Cho-Moi et That-Khé”]
Gudeodiscus anceyi , — Páll-Gergely & Hunyadi 2013, Archiv für Molluskenkunde, 142 (1): 8.

##### Types examined.

Tonkin, Bac-Kan, leg. Messager, MNHN 24600 (syntype, Figure 2B); Tonkin, Bac-Khan, NHMW 50858 (2 syntypes).

##### Museum material examined.

Tonkin, coll. Jetschin ex Berlier 1908, SMF 118124/2; Tonkin, Bac-Khan, coll. Jaeckel, S. H. ex Rolle, SMF 207668/1; Tonkin, Bac-Khan, coll. Dosch ex Rolle, SMF 172078/4; Tonkin, Than-Moi, probably ex Messager, SMF 150135/1; Central-Tonkin, Chiam-Hoa, coll. Möllendorff ex Fruhstorfer SMF 150134/1; Tonkin, Bac-Kan, leg. Messager, 22.11.1898, RBINS/5; Secteur de Nac-Ri, RBINS/1; Secteur de Nac-Ri, leg. Messager (n. 33), RBINS/1; Tonkin, entre Cho-Moi, et That-Khé, leg. Messager (n. 33), RBINS/11; Tonkin, Bac-Kan, RBINS/4; Muong-Kong, leg. Messager, MNHN-IM-2012-2139/1; Secteur de Nac-Ri, Bac-Kan, leg. Messager, MNHN-IM-2012-2250/343; Bac-Kan, leg. Messager, MNHN-IM-2012-2252/60; Bac-Kan, leg. Messager, MNHN-IM-2012-2258/38; That-Khé, coll. Mansuy, MNHN-IM-2012-2259/12; Cho-Moi, leg. Messager, MNHN-IM-2012-2263/48; Bac-Kan, leg. Messager, MNHN-IM-2012-2265/30; Long-Phai, leg. Messager, MNHN-IM-2012-2270/36; Cho-Moi, leg. Messager, MNHN-IM-2012-2275/30; Long-Phai, leg. Messager, MNHN-IM-2012-2277/26; Cho-Moi, leg. Messager, MNHN-IM-2012-2283/95; Cao-Bang, leg. Messager, MNHN-IM-2012-2468/1; Na-Ri, leg. Messager, MNHN-IM-2012-2285/40; Long- Phai, leg. Messager, MNHN-IM-2012-2286/36; Bac-Kan, coll. Letellier, 1949, MNHN-IM-2012-2287/1; Cho-Moi, leg. Messager, MNHN-IM-2012-2300/25; Bac-Kan, coll. Lavezzari, 1929, MNHN-IM-2012-2301/15; Bac-Kan, leg. Messager, MNHN-IM-2012-2305/62; Long-Phai, leg. Messager, MNHN-IM-2012-2312/30; Bac-Kan, coll. Staadt, 1969, MNHN-IM-2012-2313/4; Na-Ri, leg. Messager, MNHN-IM-2012-2376/34; Pakhé, leg. Messager, MNHN-IM-2012-2453/1; Tonkin, Bac-Khan, coll. Rolle, 4/11/08, NHMUK 20130585/3; Tonkin, Bac-Khan, coll. Rolle, 4/11/08, NHMUK 20130586/3; Tonkin, Bac-Kan, 13/6/01, NHMUK 20130587/3; Tonkin, Bac-Kan, coll. Rolle, 4/11/08, NHMUK 20130588/3; Tonkin, 4/11/8, NHMUK 20130589/2; Tonkin, Bac-Kan, coll. Salisbury ex Beddome, NHMUK 20130590/2; Tonkin, coll. Lucas, NHMUK 20130591/2; Tonkin, Bac-Khan, NHMUK 1916.03.16.1–2/2; Tonkin, NHMUK 1901.08.01.22/1; Tonkin, NHMUK 1901.7.11.89–90/2; Tonkin, Bac-Kan, coll. Rušnov ex Rolle ex Messager, NHMW 92556/6; Tonkin, Bac-Kan, coll. Wagner ex Messager, NHMW 92557/2; Tonkin, Cho-Moi, coll. Oberwimmer ex. Rosen, NHMW 71640/O/9480/1; Tonkin, Ngam-Son, coll. Wagner ex Messager, NHMW 82558/2; Tonkin, Cho-Moi, coll. Rosen, NHMW 71640/O/9479/2; Tonkin, Bac-Khan, coll. Rolle ex Messager, NHMW 50858/2; Tonkin, That-Khé, entre Cho-Moi, coll. Steenberg, ZMUC-GAS-1809/2.

##### New material examined.

**Vn10-33B** Bắc Kạn Province, Ba Bể Nat. Park, surroundings of Na Phoong cave, GPS not recorded, leg. Hemmen, Ch. & J., 10.10.2010., PGB/1; **GS21** Bắc Kạn Prov, Na Rì District, left side of road from Kim Hỷ to Bắc Kạn, 2 km after Kim Hỷ, in leaf litter bellow high limestone walls above road, 583 m, 22°16.861'N, 106°2.169'E, leg. Grego, J. & Śteffek, J., 06.04.2012., JG/1; **GS22** Bắc Kạn Prov, Na Rì District, 2 km S of Bản Dền (=Dền Village), limestone rocks at side of the valley near gold quarry, in small cavern in dense rain forest, ca 590 m, 22°14.547'N, 106°0.527'E, leg. Grego, J. & Śteffek, J., 06.04.2012., JG/1, PGB/1; **GS24** Bắc Kạn Prov, Na Rì District, 2 km S of Bản Dền, W slopes of a deep sinkhole covered with forest, leaf litter under high limestone wall, ca 640 m, 22°14.506'N, 106°0.521'E, leg. Grego, J. & Śteffek, J., 06.04.2012., JG/1; **2011/82** Lạng Sơn Province, Lũng Phầy Pass, Thất Khê N 13 km, 475 m, 22°20.363'N, 106°27.098'E, leg. Hunyadi, A., 15.11.2011., HA/4; **2011/91** Bắc Kạn Province, Ba Bể Nat. Park, 500 m on the path starting from the bungalows, 240 m, 22°25.072'N, 105°37.941'E, leg. Hunyadi, A., 17.11.2011., HA/3; **2011/93** Bắc Kạn Province, Ba Bể Nat. Park, Đầu Đằng Waterfall, above the waterfall, 175 m, 22°27.159'N, 105°34.193'E, leg. Hunyadi, A., 18.11.2011., HA/1; **2011/94** Bắc Kạn Province, Ba Bể Nat. Park, Ao Tiên, near the lake, 155 m, 22°26.831'N, 105°37.023'E, leg. Hunyadi, A., 18.11.2011., HA/3+1jb; **2011/96** Bắc Kạn Province, Ba Bể Nat. Park, Thẳm Kịt Cave 2 km, look-out tower, 335 m, 22°24.686'N, 105°37.710', leg. Hunyadi, A., 19.11.2011., HA/1; **2011/100** Bắc Kạn Province, Ba Bể Nat. Park, Bố Lù, 600 m from the harbour towards Pắc Ngòi, right side of the road, 175 m, 22°23.989'N, 105°37.523'E, leg. Hunyadi, A., 19.11.2011., HA/3; **2011/101** Bắc Kạn Province, Ba Bể Nat. Park, Na Phoong Cave, south of Bố Lù, 215 m, 22°23.341'N, 105°36.812'E, leg. Hunyadi, A., 19.11.2011., HA/3; **2012/45** Bắc Kạn Province, Na Rì Distr., Kim Hỷ SSE, 1.5 km on a by-road from the road nr. 279, 420 m, 22°16.988'N, 106°02.990'E, leg. Hunyadi, A., 29.05.2012., HA/3; **Vn10-68** Cao Bằng Province, right off old rd., ca. 33 km from Cao Bằng to Đông Khê, 22°27.547'N, 106°22.331'E, leg. Hemmen, Ch. & J., 26.03.2010., HE/1; **Vn11-159** Lạng Sơn Province, at km 74.8 on road 1B, Đồng Đăng to Thái Nguyên (8 km S Bắc Sơn), 21°54.543'N, 106°17.298'E, leg. Hemmen, Ch. & J., 02.04.2011., HE/7; **Vn11-31C** Bắc Kạn Province, Ba Bể Nat. Park, near Puổng Cave, 22°27.835'N, 105°38.997'E, leg. Hemmen, Ch. & J., 17.03.2011., HE/1; same data, leg. Hemmen, Ch. & J., 19.10.2009., PGB/2.

##### Diagnosis.

Shell very small, finely ribbed, whole shell with easily-visible spiral lines, spire elevated, umbilicus deep; aperture with well-developed, long apertural fold (Figure 9G). Parietal wall with two lamellae, the anterior is fused with the lower plica, upper plica missing (or short and fused to the anterior lamella); palatal plicae oblique, short, sometimes connected with a ridge (Figures 11C–F).

##### Measurements

(in mm): D = 7.4–7.9, D: 3.5–4 (shells from different localities, n=3); D = 9.2–9.8, H = 4.5–4.6 (Vn11-31C).

##### Differential diagnosis.

*Gudeodiscus messageri* is larger than *Gudeodiscus anceyi* and lacks the apertural fold and spiral lines on the ventral surface of the shell. *Gudeodiscus anceyi* is smaller than typical *Gudeodiscus phlyarius*, has stronger spiral lines, and has no horizontal plica under the lamellae, which are present in most populations assigned to *Gudeodiscus phlyarius*. The *Gudeodiscus phlyarius* populations living near the Chinese border (typical *anterides*, *gouldingi*, *fallax*, *verecunda*) are usually larger than *Gudeodiscus anceyi* and they often lack the apertural fold and the spiral lines on the ventral side of the shell. For differences with *Gudeodiscus hemmeni* sp. n. and *Sicradiscus mansuyi*, see under those species.

##### Intraspecific diversity.

Relatively low; shell characters, namely the size and general shell and aperture shape are rather stable. The morphology of the palatal plicae shows some diversity. The species is easily recognisable and can be separated from other plectopylid species without major problems.

##### Distribution

(see Figure 40): We have newly-collected material only from Bắc Kạn Province. The species was previously recorded from That Khé (Lạng Sơn Province) and Nac Ri (Hà Giang Province) (Gude 1901a, see also Figure 39).

#### Gudeodiscus (Gudeodiscus?) cyrtochilus

Taxon classificationAnimaliaStylommatophoraPlectopylidae

(Gude, 1909)

Figures 2F , 15E–G

Plectopylis cyrtochila Gude 1909, Proceedings of the Malacological Society of London, 8: 217–218., Plate 9, Figs 5, 5a–b. [“Muong-Kong”].
Gudeodiscus cyrtochilus , — Páll-Gergely & Hunyadi 2013, Archiv für Molluskenkunde, 142 (1): 11–12., Figs 17, 41, 75 (map).

##### Types examined.

Tonkin, Muong-Kong, leg. Messager, NHMUK 1922.8.29.59 (syntype, Figure 2F).

##### Museum material examined.

Muong-Kong, coll. Denis 1946, MNHN-IM-2012-2249/3; Muong-Kong, leg. Messager, MNHN-IM-2012-2251/14.

##### New material examined.

**2012/46** Hà Giang Province, Hà Giang 105.2 km towards Ðồng Văn, Vân Chải Commune, right side of the road nr. 4C, 23°08.865'N, 105°10.789'E, leg. Hunyadi, A., 31.05.2012., HA/7+4 jb; **2012/47** Hà Giang Province, Hà Giang 105.5 km towards Ðồng Văn, Vân Chải Commune, left side of the road 4C, 23°09.084'N, 105°10.774'E, leg. Hunyadi, A., 31.05.2012., HA/19+10jb, PGB/3; **2012/49** Hà Giang Province, Hà Giang 149.4 km towards Mèo Vạc, about 5 km SE from Ðồng Văn, right side of the road 4C, ca 1090 m, 23°15.528'N, 105°22.545'E, leg. Hunyadi, A., 01.06.2012., HA/9, PGB/1; **2012/50** Hà Giang Province, Ðồng Văn 7.5 km towards Mèo Vạc, left side of the road nr. 4C, 1260 m, 23°14.981'N, 105°23.657'E, leg. Hunyadi, A., 01.06.2012., HA/6jb; **Vn11-141** Hà Giang Province, km 105.5 on road 4c, between Yên Minh and Đồng Văn (NE of Hà Giang town), 23°08.996'N, 105°10.332'E, leg. Hemmen, Ch. & J., 21.03.2011., HE/16; **Vn11-144** Hà Giang Province, km 149.4 on road 4c, between Đồng Văn to Mèo Vạc (NE of Hà Giang Town), 23°15.507'N, 105°22.564'E, leg. Hemmen, Ch. & J., 23.03.2011., HE/4; **Vn11-145** Hà Giang Province, km 153 on road 4c, between Đồng Văn to Mèo Vạc (NE of Hà Giang Town), left side of road, 23°14.738'N, 105°23.786'E, leg. Hemmen, Ch. & J., 23.03.2011., HE/1; **Vn11-123A** Hà Giang Province, ca. 7.5 km from Đồng Văn to Mèo Vạc (right side off road), 23°14.906'N, 105°23.445'E, leg. Hemmen, Ch. & J., 23.03.2011., HE/3.

##### Diagnosis.

Shell very small to small, discoid, polished with very weak apertural rim, weak or missing callus and without apertural fold. Parietal wall with two lamellae and an upper and a lower horizontal plica; the plicae can be free from the anterior lamella or in contact with it; palatal plicae straight, parallel, horizontal, sometimes connected with a slight ridge (Figures 15E–G).

##### Measurements

(in mm): D = 8.9–9.9, H = 4.8–5.0 (n=4, MNHN-IM-2012-2251); D = 10.2–11.1, H = 5.3–5.6 (n=3, 2012/47); D = 10.2–11.2, H = 4.8–5.4. (Chinese specimens, n=4, see Páll-Gergely and Hunyadi 2013).

##### Differential diagnosis.

The Chinese *Gudeodiscus yunnanensis* has a similar shell shape but possesses only one vertical parietal lamella (the anterior one is absent). The two species can be separated only the basis of the presence or absence of the anterior lamella. In *Gudeodiscus soosi* and in most specimens of *Gudeodiscus multispira*, few denticles are present between the upper and lower plicae, at the place of the anterior lamella. Moreover, *Gudeodiscus multispira* has a greater number of whorls and the last whorl is wider in relation to the previous one than in *Gudeodiscus cyrtochilus*. *Gudeodiscus infralevis* is larger with a more elevated spire, stronger apertural lip and usually a weak apertural fold. See also under *Gudeodiscus fischeri*.

##### Intraspecific diversity.

Low; shell characters rather stable. The parietal plicae and lamellae and their respective position (reaching each other or not) show some diversity within the species. The palatal plicae are not variable, but in some shells they are connected to each other with a ridge, whereas in others they are free. It is possible that mature specimens tend to have a connection between the plicae. The species is easily recognisable and can be separated from other plectopylid species without major problems.

##### Distribution

(see Figure 41): The species was described from “Muong-Kong” (=Mường Khương, Lào Cai Province; see Figure 39). Material is noted from northeast of this locality, from northern Hà Giang Province and eastern parts of Yunnan Province (China) (see Páll-Gergely and Hunyadi 2013).

##### Remarks.

The drawing in the original description of *Gudeodiscus cyrtochilus* is incomplete (the posterior lamella was omitted).

Some fresh shells have a characteristic mosaic structure on the dorsal surface (yellowish and darker reddish areas are following each other). This coloration is known in some “*Chersaecia*” (*munipurensis* Godwin-Austen, 1875, *oglei* Godwin-Austen, 1879, *serica* Godwin-Austen, 1875) and *Plectopylis* (e.g. *anguina* Gould, 1847, *bensoni* Gude, 1914, *karenorum* W. Blanford, 1865) species.

#### Gudeodiscus (Gudeodiscus) dautzenbergi

Taxon classificationAnimaliaStylommatophoraPlectopylidae

(Gude, 1901)

Figures 8E–F , 9K–L , 14A–G

Plectopylis Dautzenbergi Gude 1901a, Journal de Conchyliologie, 49: 198–200., Figs 1a–f. Plate 6, Figs 1a–c. [“That Khé (le type); entre Cho-Moï et Bac-Kan; entre Bac-Kan et Nac-Ri”]
Plectopylis persimilis Gude 1901a, **syn. n.**, Journal de Conchyliologie, 49: 209–211., Figs 7a–f, Plate 6, Figs 7a–c. [“Environs de That-Khé”].
Plectopylis schlumbergeri , — Zilch 1959–1960, Handbuch der Paleozoologie, 6 (2) Euthyneura: Fig. 2094.
Gudeodiscus dautzenbergi , — Páll-Gergely & Hunyadi 2013, Archiv für Molluskenkunde, 142 (1): 8.

##### Types examined.

Tonkin, That-Khé, MNHN 24603 (holotype of *dautzenbergi*, Figure 8E); Environs de That-Khé, leg. Messager (n. 22.), MNHN 24602 (holotype of *persimilis*, Figure 8F).

##### Museum material examined.

Tonkin, Nja-Ba-Thà, coll. Dosch ex Rolle, SMF 341738/1; Tonkin, That-Khé, coll. Dorsch ex Rolle ex Messager, SMF 172083/2; Tonkin, coll. Jetschin ex Bonnet 1900, SMF 102823/1; Fr. Indochina, Tonkin, That Ké, leg. Demange, 1911, HNHM 10278/2; Tonkin, coll. Sayer 1969, MNHN-IM-2012-2273/1; Tonkin, coll. Letellier 1949, MNHN-IM-2012-2274/1; Bac-Kan, leg. Messager 1904, coll. Lavezzari, 1929, MNHN-IM-2012-2290/5; Tonkin, leg. Messager, MNHN-IM-2012-2292/2; Bac-Kan, leg. Messager, MNHN-IM-2012-2297/2; Tonkin, coll. Denis 1946, MNHN-IM-2012-2303/4; Bac-Kan, leg. Messager, MNHN-IM-2012-2314/7; That Khé, leg. Messager, MNHN-IM-2012-2327/4; Bac-Kan, leg. Messager, MNHN-IM-2012-2331/5; Bac-Kan, leg. Messager, MNHN-IM-2012-2437/1; Bac-Kan et That Khé, coll. Staadt 1969, MNHN-IM-2012-2280/2; Na-Ri, leg. Messager, MNHN-IM-2012-2461/1; That-Khé, leg. Messager, MNHN-IM-2012-2373/6; That-Khé, leg. Messager, MNHN-IM-2012-2378/4; Bac-Kan, leg. Messager, MNHN-IM-2012-2382/4; Bac-Kan, leg. Messager, MNHN-IM-2012-2383/4+14jb; Bac-Kan, leg. Messager, MNHN-IM-2012-2402/3; Than-Moi, coll. Staadt, 1969, MNHN-IM-2012-2336/1; Bac-Kan, leg. Messager, MNHN-IM-2012-2337/26+2jb; That-Khé, leg. Messager, MNHN-IM-2012-2354/4; Cao-Bang, leg. Messager, MNHN-IM-2012-2360/1; Tonkin, That-Khé, coll. Salisbury ex Beddome, Tonkin, coll. Lucas, Acc. no. 2351, NHMUK 20130614/2; Tonkin, coll. Lucas, Acc. no. 2351, NHMUK 20130615/1; Tonkin, coll. Trechmann, Acc. no. 2176, NHMUK 20130616/2; Tonkin, That Ke (?), coll. Kennard, A. S. ex auct. (Gude), NHMUK 20130617/1; Tonkin, That-Khe, coll. Rolle, 4/11/08, NHMUK 20130618/2; Tonkin, That-Khé, 13/6/03, NHMUK 20130619/2; Tonkin, That-Khé, NHMUK 1901.7.11.1/1; Tonkin, That-Khé, NHMUK 1920.1.20.18/1; Tonkin, That-Khé, NHMUK 1908.12.21.142–143/2; Tonkin, That-Khé, NHMW 46024/1; Tonkin, That-Khé, coll. Rolle, NHMW 92559/2; Tonkin, That-Khé, coll. Oberwimmer, NHMW 71640/O/10285/1; Tonkin, That-Ke, coll. Wagner ex Messager, NHMW 71640/O/10285/1 (mixed sample with *schlumbergeri*); Bac Kan, coll. Steenberg, ZMUC-GAS-1084/1; Tonkin, coll. Steenberg, ZMUC-GAS-1805/2.

##### New material examined.

**Vn10-44** Bắc Kạn Province, Chợ Mới (left bank of river); 21°52.682'N, 105°47.078'E, leg. Hemmen, Ch. & J., 17.03.2010., PGB/3; **Vn10-42** Thái Nguyên/Bắc Kạn Province, ca. 1 km S of Chợ Mới; 21°52.707'N, 105°46.172'E, leg. Hemmen, Ch. & J., 17.03.2010., PGB/3; **2011/103** Bắc Kạn Province, Chợ Mới, eastern bank of the river, Khuôn Thung cross 500 m towards Quảng Chu Commune, right side of the road, 21°52.508'N, 105°47.328'E, leg. Hunyadi, A., 21.11.2011., HA/10+4jb, PGB/1; **2011/104** Thái Nguyên Province, Chợ Chu (=Chu Market), rocky wall above the NE part of the village, 90 m, 21°54.613'N, 105°39.195'E, leg. Hunyadi, A., 21.11.2011., HA/3.

##### Diagnosis.

Shell medium-sized or large, with irregular growth lines, but appearing almost smooth; spire slightly elevated, apertural lip thick but blunt; apertural fold strong and oblique, connected to the callus, but reaching its maximum height some distance from the callus (Figures 9K–L). Parietal wall with two parietal lamellae; the anterior one has an anteriorly conspicuously elongated lower “leg”; this structure may have resulted from the connection of the anterior lamella and the lower plica; middle palatal plicae oblique (Figures 14A–G).

##### Measurements

(in mm): D = 16.7–20.6, H = 8.9–9.8 (n=3, Vn10-42); D = 16.1–17.8, H = 7.9–9.2 (n=2, Vn10-44).

##### Differential diagnosis.

*Gudeodiscus villedaryi*, which is probably the closest relative, differs from *Gudeodiscus dautzenbergi* by the presence of an additional horizontal parietal plica under the vertical lamellae, near the suture. Distinguishing *Gudeodiscus dautzenbergi* from some similar looking populations of *Gudeodiscus villedaryi* is impossible without breaking the shell and observing the parietal plicae. Most populations of *Gudeodiscus villedaryi* however, have a sharp periumbilical keel, which always absent in *Gudeodiscus dautzenbergi* (see also Remarks under *Gudeodiscus villedaryi*). *Gudeodiscus dautzenbergi* is flatter and more widely umbilicated than *Gudeodiscus giardi*. The latter species has a domed shell, thinner shell wall and thicker peristome. For comparisons with *Halongella schlumbergeri*, see under that species. Distinguishing *Gudeodiscus dautzenbergi* from *Halongella schlumbergeri* requires experience, but is possible without breaking the shell on the basis of the formation of the peristome and the apertural fold (Figures 9K–N).

##### Intraspecific diversity.

Low; shell characters stable.

##### Distribution

(see Figure 40): This species as well as *Plectopylis persimilis* (synonym of *Gudeodiscus dautzenbergi*) were described from That-Khé (northern Lạng Sơn Province) (see Figure 39). Our newly-collected material is from the border region of the Thái Nguyên and Bắc Kạn provinces.

##### Remarks.

The holotype of *Plectopylis persimilis* and that of *Plectopylis dautzenbergi* do not show significant differences in terms of shell shape, size, aperture shape and the formation of the plicae and lamellae; therefore we synonymise *Plectopylis persimilis* with *Plectopylis dautzenbergi*. These two species were described in the same publication (Gude 1901a), therefore the name introduced earlier (*dautzenbergi*, page 198) is considered a senior synonym.

*Gudeodiscus dautzenbergi* and *Gudeodiscus villedaryi* are separated here on the basis of the presence or absence of a lower plica, although the two species may be conspecific. More information is necessary to clarify the distinctness of *Gudeodiscus dautzenbergi*.

The specimen figured by Zilch (1960, Fig. 2094) under the name Plectopylis (Endoplon) schlumbergeri is missing. There is a note written by Zilch saying that he found the box empty on 11.12.1963 (Ronald Janssen, pers. comm., October 2013). Although the specimen could not be examined by us, we are confident in stating that the figure shows a shell of *Gudeodiscus dautzenbergi*.

#### Gudeodiscus (Gudeodiscus) fischeri

Taxon classificationAnimaliaStylommatophoraPlectopylidae

(Gude, 1901)

Figures 2E , 3A–C , 9P–Q , 15H–R , 17 , 18 , 28D , 29D , 29J , 30E , 31D , 34M–O

**Figure 3.**
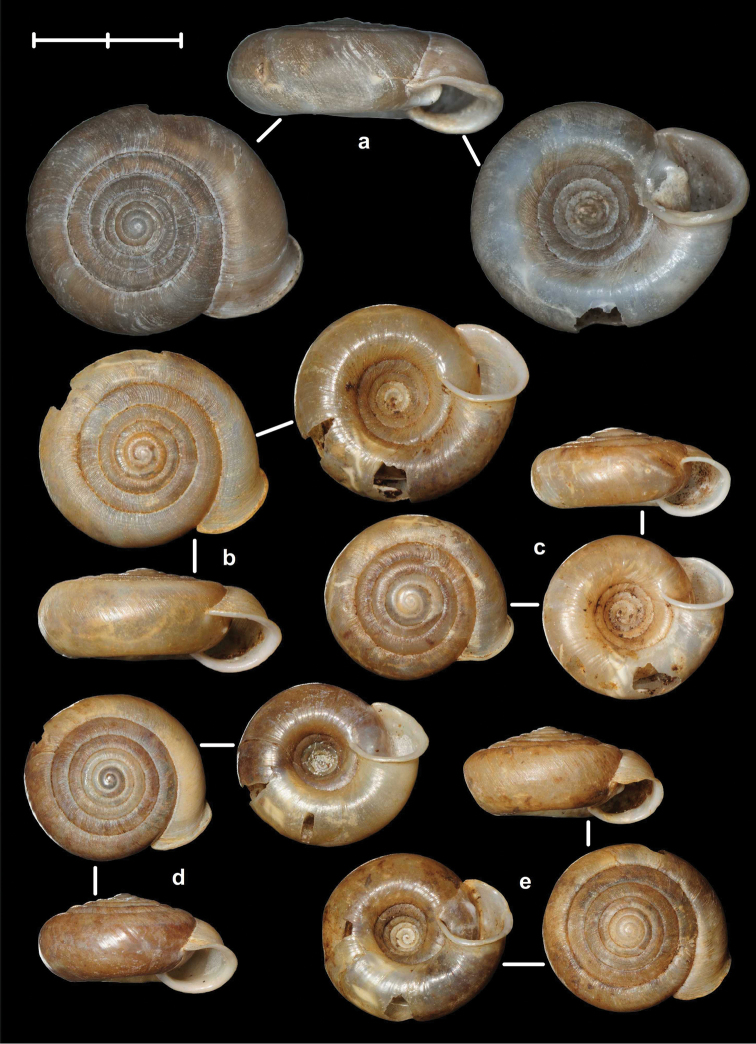
Shells of Vietnamese *Gudeodiscus* species. **A** Gudeodiscus (Gudeodiscus) fischeri (Gude, 1901), Vn10-120, coll. PGB **B** Gudeodiscus (Gudeodiscus) fischeri, MNHN 24579 (holotype of *Plectopylis fischeri*) **C** Gudeodiscus (Gudeodiscus) fischeri, MNHN 24587 (holotype of *Plectopylis tenuis*) **D** Gudeodiscus (Gudeodiscus?) infralevis (Gude, 1908), MNHN 24604 (holotype of *Plectopylis infralevis*) **E** Gudeodiscus (Gudeodiscus?) infralevis, MNHN 24585 (holotype of *Plectopylis soror*). Photos: B. Páll-Gergely (**A**) and T. Deli (**B–E**). Scale represents 10 mm.

**Figure 4.**
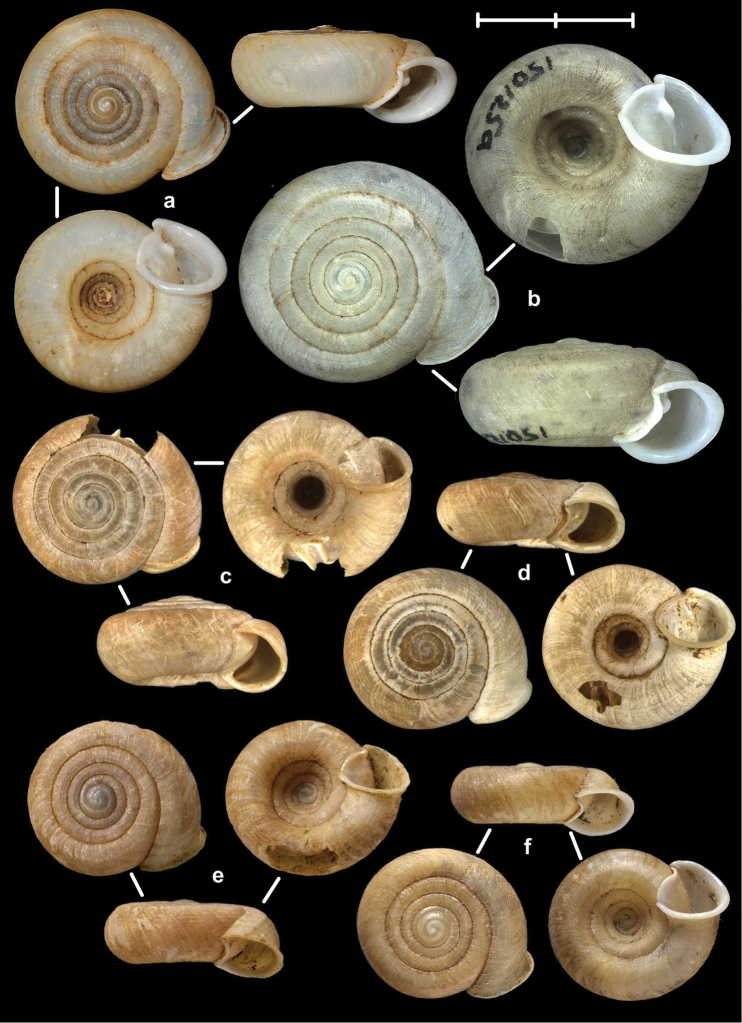
Shells of Vietnamese *Gudeodiscus* species. **A** Gudeodiscus (Gudeodiscus) phlyarius (Mabille, 1887), MNHN 24581 (syntype of *Plectopylis phlyaria*) **B** Gudeodiscus (Gudeodiscus) phlyarius, SMF 150125a (lectotype of *Plectopylis moellendorffi*) **C** Gudeodiscus (Gudeodiscus) cf. phlyarius, Vn10-41, coll. PGB **D** Gudeodiscus (Gudeodiscus) phlyarius, Vn09-06, coll. HE **E** Gudeodiscus (Gudeodiscus) phlyarius, NHMUK 1922.8.29.56 (holotype of *Plectopylis gouldingi*) **F** Gudeodiscus (Gudeodiscus) phlyarius, NHMUK 1922.8.29.57 (holotype of *Plectopylis anterides*). Photos: T. Deli (**A**), E. Neubert (**B**), B. Páll-Gergely (**C, D**) and H. Taylor (**F**). Scale represents 10 mm.

Plectopylis Fischeri Gude 1901a, Journal de Conchyliologie, 49: 204–205., Figs 4a–e, Plate 6, Figs 4a–c. [“Environs de Bac-Kan”].
Plectopylis tenuis Gude 1901a, **syn. n.**, Journal de Conchyliologie, 49: 202–204, 205., Figs 3a–e, Plate 6, Figs 3 a–c. [“Cho-Ra (le type); environs de Bac-Khan; environs de Cho Moi”].
Plectopylis Fischeri , — Dautzenberg & Fischer 1905b, Journal de Conchyliologie, 53: 360. [“Ha Giang”].
Plectopylis tenuis , — Gude 1909, Proceedings of the Malacological Society of London, 8: 215, 216.
Gudeodiscus fischeri , — Páll-Gergely & Hunyadi 2013, Archiv für Molluskenkunde, 142 (1): 8.
Gudeodiscus tenuis , — Páll-Gergely & Hunyadi 2013, Archiv für Molluskenkunde, 142 (1): 8.

##### Types examined.

Tonkin, Environs de Bac-Kan, leg. Messager, MNHN 24579 (holotype of *fischeri*, Figure 3B); Tonkin, Cho-Ra, leg. Messager, MNHN 24587 (holotype of *tenuis*, Figure 3C).

##### Museum material examined.

Tonkin, Bac-Kan, NHMUK 1908.12.21.144/1; Tonkin, environs de Bac-Kan, leg. Messager, (n. 28), RBINS/2; Tonkin, Ha-Giang, leg. Messager, RBINS/5; Ha Giang, leg. Mansuy, coll. M. H. Fischer, MNHN-IM-2012-2241/12 adult, 1jb; Ha Giang, coll. Mansuy, MNHN-IM-2012-2257/5; Tonkin, leg. Messager, MNHN-IM-2012-2390/1; Cho-Ra, leg. Messager, MNHN-IM-2012-2477/1; Bac-Kan, leg. Messager, MNHN-IM-2012-2466/3; Tonkin, Cho Rah, ex Rolle, USNM 207813/2 („*tenuis*“); Nga-Son, leg. Messager, MNHN-IM-2012-2233/2 („*tenuis*“); Nga-Son, leg. Messager, MNHN-IM-2012-2253/2 (“*tenuis*”); Tonkin, coll. Denis, 1946, MNHN-IM-2012-2338/3 (“*tenuis*”); Cho-Ra, leg. Messager, MNHN-IM-2012-2361/1 (“*tenuis*”); Tonkin, Bac-Kan, coll. Rolle, NHMW 71640/O/14028/1.

##### New material examined.

**20090519B** Tuyên Quang Province, Hàm Yên District, Yên Phú Commune, Đồng Tiến, Thống Nhất, ca 70 m, 22°08.673'N, 104°58.634'E, leg. Ohara, K., 19.05.2009., OK/12, PGB/3; **20090515C** Bắc Kạn Province, Ba Bể District, Ba Bể Nat. Park, Khâu Kum, ca 185 m, 22°26.465'N, 105°36.642'E, leg. Ohara, K., 15.05.2009., OK/8, PGB/2; **20081113C** Hà Giang Province, Hà Giang Town, Ngọc Đường Commune, Bản Cườm (= Cườm Village), ca 110 m, 22°51.180'N, 105°01.075'E, leg. Ohara, K., 13.11.2008., OK/1, PGB/1; **Vn10-118** Hà Giang Province, Tâm Village, ca. 7–8 km SE of Hà Giang (between Vị Xuyên and Bản Hãm = “Hãm Village”), 22°48.019'N, 105°00.888'E, leg. Hemmen, Ch. & J., 16.10.2010., PGB/2; **Vn11-138** Tuyên Quang Province, near Tôn Hông, road #185 from Tuyên Quang to Vĩnh Lộc (formerly Chiêm Hóa) (NE of Tuyên Quang), leg. Hemmen, Ch. & J., 19.03.2011., HE/1, PGB/1 (anatomically examined, see Figures 17, 28D, 29J, 31D, 34M–O); **Vn10-120** Hà Giang Province, ca. 9.8 km from Hà Giang to Tam Sơn (formerly Quản Bạ), left side off road, 22°52.907'N, 104°59.885'E, leg. Hemmen, Ch. & J., 17.10.2010., PGB/3; **2012/56** Hà Giang Province, Hà Giang 7 km towards Tam Sơn, left side of the road nr. 4C, 100 m, 22°51.650'N, 105°00.768'E, leg. Hunyadi, A., 03.06.2012., HA/4; **2012/57** Hà Giang Province, Hà Giang 9.8 km towards Tam Sơn, left side of the road 4C, 120 m, 22°52.881'N, 104°59.927'E, leg. Hunyadi, A., 03.06.2012., HA/20+7jb, PGB/2; **Vn11-179** Tuyên Quang Province, ca. 5.5 km E of Chương Dương (left bank of Lô River), leg. Hemmen, Ch. & J., 30.09.2011., HE/2; **20090517A** Bắc Kạn Province, Ba Bể District, Ba Bể Nat. Park, along the trekking road, near guest house, 205 m, 22°25.049'N, 105°37.699'E, leg. Ohara, K., 17.05.2009., OK/8, PGB/2 (“*tenuis*”, anatomically examined, see Figure 29D); **Vn10-28A** Bắc Kạn Province, ca 1 km from Ba Bể Nat. Park, headquarters to Ba Bể Lake, 22°24.829'N, 105°37.652'E, leg. Hemmen, Ch. & J., 20.10.2010., PGB/6 (“*tenuis*”); **Vn09-26** Bắc Kạn Province, Ba Bể Nat. Park, near bungalows (at Park Headquarters), leg. Hemmen, Ch. & J., 17.10.2009., HE/2 (“*tenuis*”); **2011/91** Bắc Kạn Province, Ba Bể Nat. Park, path starting from the bungalows 500 m, 240 m, 22°25.072'N, 105°37.941'E, leg. Hunyadi, A., 17.11.2011., HA/11+5jb, PGB/2 (“*tenuis*”); **2011/96** Bắc Kạn Province, Ba Bể Nat. Park, Thẳm Kịt Cave 2 km from the look-out tower, 335 m, 22°24.686'N, 105°37.710'E, leg. Hunyadi, A., 19.11.2011., HA/29+3jb, PGB/2 (“*tenuis*”, anatomically examined, see Figure 18); **2011/97** Bắc Kạn Province, Ba Bể Nat. Park, Thẳm Kịt Cave 1 km from the look-out tower, no GPS data, leg. Hunyadi, A., 19.11.2011., HA/8+4jb (“*tenuis*”).

##### Diagnosis.

Shell small to medium-sized, with smooth basal and usually finely ribbed apical surface (in some populations also smooth and glossy, see Figure 2E); shell usually flat, or with very slightly elevated spire, or only the protoconch is elevated from the dorsal surface; callus and apertural fold (if present) weak (Figures 9P–Q). Parietal wall with two lamellae (the anterior is exceptionally dissolved into small denticles); middle palatal plicae oblique, depressed Z or L-shaped, they are free or sometimes connected to each other (Figures 15H–N).

##### Measurements

(in mm): D = 16.6–18.6, H = 7–7.9 (n=3, Vn10-120); D = 12.1–12.4, H = 4.8–5.3 (n=3, 20090519B); D = 15.5–15.9, H = 7.1–7.2. (n=2, 20090515C); D = 14.6, H = 7.4–7.6. (n=2, 2011/91); D = 12.9–14.7, H = 6.4–7.3 (n=6, Vn10-28A).

##### Differential diagnosis.

*Gudeodiscus cyrtochilus* is smaller than *Gudeodiscus fischeri*, it has a narrower umbilicus, more regularly growing whorls (the last whorl is only slightly wider than the penultimate one), a shorter lower horizontal parietal plica and no apertural fold. The Chinese *Gudeodiscus multispira* and *Gudeodiscus soosi* are also smaller, have a greater number of densely-coiled whorls and at the position of the anterior lamella there are usually 2–4 clearly separated denticles (see also Remarks). In some populations of *Gudeodiscus multispira* the denticles are missing so that only the posterior lamella is present. *Gudeodiscus yunnanensis* has no anterior lamella, just a curved single lamella (homologous with the posterior lamella). *Gudeodiscus eroessi* never has an apertural fold and its anterior lamella is dissolved into small denticles, or missing. *Gudeodiscus infralevis* and *Gudeodiscus suprafilaris* have a more elevated spire, narrower umbilicus and rather straight, horizontal, parallel plicae.

##### Intraspecific diversity.

The variability is quite large in terms of shell size and shape, sculpture, strength of the callus and apertural fold and the formation of parietal plicae and lamellae. The combination of weak callus and apertural fold and the “nautiliform” shape helps in the identification of the species. See also Table 7.

**Table 7.** Diversity of shell characters within the species Gudeodiscus (Gudeodiscus) fischeri. Abbreviations: OAE: only apex elevated.

code	callus and apertural fold	anterior lamella	lamella and lower plica	shells opened	shell	spire	remarks
2012/57= Vn10-120	strong	dissolved	not in contact	2	thick, greyish	slightly elevated	large
2012/56	strong	normal	connected	1	thick, greyish	slightly elevated	
20081113C	strong	normal	connected	1	thick, greyish	slightly elevated	
Vn10-118	strong	normal	connected	1	thick, greyish	OAE	
20090515C	weak	normal	connected	2	thin, translucent, corneous	slightly elevated	typical *fischeri*
20090519B	weak	normal	connected	2	very thin, translucent, yellowish	OAE	small
2011/96= 2011/91= 2009.05.17A= Vn10-28A	weak	normal	not in contact	5	thin, translucent, corneous	elevated	typical *tenuis*

##### Description of the genitalia.

Two specimens were dissected, belonging to two different populations: “Specimen1” Tuyên Quang Province, near Tôn Hông, road #185 from Tuyên Quang to Vĩnh Lộc (formerly Chiêm Hóa) (NE of Tuyên Quang), leg. Hemmen, Ch. & J., 19.03.2011. (specimen without embryos in the uterus, but with calcareous hooks inside the penis, Figure 17, 31D); “Specimen2” Bắc Kạn Province, Ba Bể Nat. Park, Thẳm Kịt Cave 2 km from the look-out tower, 335 m, 22°24.686'N, 105°37.710'E, leg. Hunyadi, A., 19.11.2011. (typical *Plectopylis tenuis*; with a developing embryo in the uterus, Figure 18).

The penis is a cylindrical tube with several longitudinal, parallel folds on the inner wall; there are pockets formed by some of these folds; in the wall of the opened penis the series of pockets are arranged along a bell-shaped line (Figure 28D); there were calcareous hooks within the pockets of “Specimen1”; the base of the hooks were elongated, they lay within the pockets, whereas the tip portion projects out of the pockets (Figure 30E); epiphallus as long as the penis, with few parallel folds in the lumen (Figure 29D); distal portion of the penis and the proximal part of the epiphallus are connected with weak membrane; more closely to the genital opening these two organs are more stronger connected; penial caecum tapers toward the end, it is about a quarter as long as the penis; its inner wall with irregular folds arranged in longitudinal lines, with calcareous granules in between (mainly at the distal end); retractor muscle attaching on the apical part of the penial caecum is approximately as long as the caecum; there is an additional retractor muscle on the proximal part of the penis. Vagina is thickened and forms a “vaginal bulb”, which is attached to the body wall with several thin ligaments; inner wall of the vaginal bulb and the distal part of the vagina with well-developed, longitudinal, serrulate folds (Figure 31D); stem of the gametolytic sac is long and slim; it is attached hardly to the spermoviduct; diverticulum well-developed, free; the diverticulum of the specimen from the Ba Bể Nat. Park contained three long, slightly C-shaped spermatophores; the proximal side of the spermatophores were damaged, thus they might have been connected; spermoviductus slim and long.

Besides the presence or absence of embryos and calcareous penial hooks between the two specimens the only notable difference is the longer retractor muscle in “Specimen2” than in “Specimen1”, but the taxonomic value of this character is unknown.

##### Radula.

See Table 6 and Figures 34M–O.

##### Distribution

(see Figure 41): *Gudeodiscus fischeri* is known from Hà Giang, Tuyên Quang and Bắc Kạn Provinces.

##### Remarks.

Some samples from the Ba Bể Nat. Park (Vn10-28A, 20090517A, 2011/91, 2011/96) are identical with the type specimen of *Plectopylis tenuis* described from Cho Ra (see Figure 39). This town is situated approximately 7 km from the locality of our recent material. Some 3 km north of our *tenuis* localities there is a population (20090515C) which agrees with *tenuis* in every shell character except that the anterior parietal lamella and the lower horizontal plica are connected (typical in *fischeri*). Since no other shell characters are known to be different between *tenuis* and *fischeri*, and other populations of *fischeri* show relatively large variability in terms of several shell characters, we synonymize *Plectopylis tenuis* with *Plectopylis fischeri*.

The shells collected 9.8 km north of Hà Giang are relatively large and thick-walled, have the anterior lamella dissolved into 3–4 denticles, and have strong apertural denticle and callus (Figures 3A, 15L–M). The shells collected at Đồng Tiến are small and very glossy in appearance (Figure 2E).

#### Gudeodiscus (Gudeodiscus?) francoisi

Taxon classificationAnimaliaStylommatophoraPlectopylidae

(Fischer, 1898)

Figures 7A–C , 13E–K

Plectopylis Françoisi Fischer 1898b, Journal de Conchyliologie, 46: 214–218., Figs 1, 3–4. [“rochers calcaires Déo-Ma-Phuc”].
Plectopylis Françoisi Fischer 1899, Bulletin biologique de la France et de la Belgique, 32: 330–332., Figs 1, 3–4. [“rochers calcaires Déo-Ma-Phuc”].
Plectopylis françoisi , — Gude 1899b, Science Gossip, 6: 75–76., Figs 201a–e.
Plectopylis (Endoplon) françoisi , — Gude 1899c, Science Gossip, 4: 148.
Plectopylis (Endoplon) françoisi , — Gude 1899d, Science Gossip, 6: 175.
Plectopylis lepida Gude 1900, **syn. n.**, The Annals and Magazine of Natural History, 7 (5): 313. [“Tonkin, Tinh-Tuc”].
Plectopylis Bavayi Gude 1901a, **syn. n.**, Journal de Conchyliologie, 49: 200–202., Figs 2a–e, Plate 6, Figs 2a–c. [That Khé (le type); secteur de Nac-Ri].
Plectopylis lepida , — Gude 1901b, Journal of Malacology, 8: 48–49., Figs 4a–f.
Plectopylis Bavayi , — Dautzenberg & Fischer 1908, Journal de Conchyliologie, 56: 177. [Quang-Huyen].
Gudeodiscus francoisi , — Páll-Gergely & Hunyadi 2013, Archiv für Molluskenkunde 142 (1): 8.
Gudeodiscus lepidus , — Páll-Gergely & Hunyadi 2013, Archiv für Molluskenkunde 142 (1): 8.

##### Types examined.

Rochers calcaires de Déo-Ma-Phuc, leg. Dr. Billet, 23.10.1892, MNHN 9945 (holotype of *francoisi*, Figure 7B); That-Khé, leg. Messager, MNHN 24601 (holotype of *bavayi*, Figure 7A); Tonkin, Tinh-Tuc, NHMUK 1922.8.29.51 (holotype of *lepida*, Figure 7C).

##### Museum material examined.

Tonkin, coll. Jetschin ex Bonnet 1900, SMF 102826/1; Tonkin, That Khé, coll. Dosch ex Rolle, SMF 172090/4; Tonkin, That-Khé, coll. Dosch ex Rolle, SMF 172082/2; Tonkin, leg. Messager, MNHN-IM-2012-2227/6; Tonkin, leg. Messager, MNHN-IM-2012-2229/4; Tonkin, coll. Letellier 1949, MNHN-IM-2012-2267/1; Secteur de Nac-Ri, leg. Messager, MNHN-IM-2012-2268/5; That-Khé, coll. Lavezzari, 1929, MNHN-IM-2012-2276/5; Tonkin, leg. Messager, MNHN-IM-2012-2284/1; That Ké, Nac Ri, leg. Messager, MNHN-IM-2012-2333/8; Tonkin, leg. Messager, MNHN-IM-2012-2353/1; Na-Cham, leg. Messager, MNHN-IM-2012-2358/5; Na-Ri, leg. Messager, MNHN-IM-2012-2363/5; Tonkin, leg. Messager, MNHN-IM-2012-2428/1; Tonkin, leg. Messager, MNHN-IM-2012-2440/6; Tonkin, leg. Messager, MNHN-IM-2012-2430/7; Nac-Ri et That-Khe, coll. Staadt, 1969, MNHN-IM-2012-2386/2; Tonkin, leg. Messager, MNHN-IM-2012-2371/3; That-Khé, leg. Messager, MNHN-IM-2012-2377/30+3jb; Tonkin, That-Khé, coll. Salisbury ex Beddome, NHMUK 20130592/2; Tonkin, coll. Kennard, A. S. ex auct. (Gude), NHMUK 20130593/2; Tonkin, coll. Lucas, Acc. no. 2351, NHMUK 20130594/2; Tonkin, coll. Lucas, Acc. no. 2351, NHMUK 20130595/2; Tonkin, That-Khé, V.W. MacAndrew Coll, 13/6/01.114, NHMUK 20130596/2; Tonkin, NHMUK 1916.3.15.4–5/2 (“showing immature armature”); Tonkin, That Khé, NHMUK 1901.7.11.46/1; Tonkin, That-Khé, NHMUK 1908.12.21.118–119/2; Baie d’Along, coll. Staadt, 1969, MNHN-IM-2012-2311/1 (similar to the holotype of *Plectopylis lepida*); Tonkin, That-Khe Na-Ri, coll. Rušnov ex Rolle ex Messager, NHMW 92561/2; Tonkin, Phi-Mi, coll. Steenberg, ZMUC-1807/1; Tonkin, coll. Steenberg, ZMUC-GAS-1806/1; Tonkin, coll. Steenberg, ZMUC-GAS-1810/1.

##### New material examined.

**GS17** Bắc Kạn Province, Na Rì Distr., limestone cliffs on the left side of the road to Kim Hỷ, 2 km before Kim Hỷ, soil in small cavern, ca 560 m, 22°16.897'N, 106°2.754'E, leg. Grego, J. & Śteffek, J., 05.04.2012., JG/1, PGB/1; **GS22** Bắc Kạn Province, Na Rì District, 2 km S of Bản Dền (=Dền Village), limestone rocks at side of the valley near gold quarry, in small cavern in dense rain forest, ca 590 m, 22°14.547'N, 106°0.527'E, leg. Grego, J. & Śteffek, J., 06.04.2012., JG/1; **GS24** Bắc Kạn Prov, Na Rì Distr., 2 km S of Bản Dền, W slopes of a deep sinkhole covered with forest, leaf litter under high limestone wall, ca 640 m, 22°14.506'N, 106°0.521'E, leg. Grego, J. & Śteffek, J., 06.04.2012., JG/1, PGB/2; **2011/80** Cao Bằng Province, Đèo Mã Phục (pass) 1 km towards Quảng Uyên, right side of the road, 565 m, 22°43.918'N, 106°20.490'E, leg. Hunyadi, A., 14.11.2011., HA/2+2jb; **2012/41** Cao Bằng Province, Đèo Mã Phục (pass) 1 km towards Quảng Uyên, right side of the road, 570 m, 22°43.896'N, 106°20.484'E, leg. Hunyadi, A., 27.05.2012., HA/11+2jb, PGB/2.

##### Diagnosis.

Shell small to medium-sized, yellowish or mustard-coloured, glossy, with slowly increasing whorls, deep umbilicus, domed dorsal side; thin apertural lip and well-developed apertural fold. Parietal wall with two parietal lamellae; the anterior one is connected to the lower plica; middle palatal plicae oblique, depressed Z-shaped (Figures 13E–K).

##### Measurements

(in mm): D = 13.2, H = 6.7 (holotype of *lepida*); D = 19.6–19.8, H = 10.4–10.7 (N = 2, NHMUK 20130593); D = 17.8–18.0, H = 9.8–9.9 (n=2, NHMUK 1908.12.21.118–119).

##### Differential diagnosis.

The glossy, dark yellow shell, the characteristic apertural fold and shell shape makes this species easily distinguishable from most congeners. *Gudeodiscus francoisi* has a smoother shell, weaker apertural lip and more regular whorls than *Gudeodiscus giardi giardi*. In the type locality of *francoisi* (Déo-Ma-Phuc, see Figure 39) the species lives together with *Gudeodiscus giardi giardi*. In some cases the two species can be hardly distinguished, especially in the case of subadult *giardi* specimens which cannot be easily distinguished from *francoisi*. The possibility of hybridisation in that locality cannot be excluded; however specimens from other localities are easily distinguishable.

##### Intraspecific diversity.

The species shows little intraspecific variability in terms of shell characters. The “*lepida*-like” shells are considered to the results of abnormal growth.

##### Distribution

(see Figure 42): Newly-collected material from Cao Bằng and Bắc Kạn Provinces was examined. There is a single shell which is identical to the holotype of *Plectopylis lepida* and is labelled as being collected from Hạ Long Bay, but this collection locality is probably incorrect.

##### Remarks.

*Gudeodiscus bavayi* is a synonym of *Gudeodiscus francoisi*. The two holotypes are identical in shell shape and arrangement of the inner lamellae. The only difference is that the holotype of *Gudeodiscus francoisi* lacks an apertural fold because it is a subadult shell. Other shells collected from the type locality are identical with the holotype of *Plectopylis bavayi*. *Plectopylis lepida* was described on the basis of a single shell. During the revision of the Vietnamese Plectopylidae material in the MNHN, we found a single shell (Baie d’Along, coll. Staadt, 1969, MNHN-IM-2012-2311) which is identical in shell shape and plication with the holotype of *lepida*. These two shells differ from *Gudeodiscus francoisi* only by the absence of the posterior lamella and the weak apertural fold. The absence of the posterior lamella is probably the result of unusual development, which is also visible in a specimen of *Gudeodiscus suprafilaris* (see under that species). The weak apertural fold can be explained by subadult stages of these shells. Since no other shell characters distinguish *Plectopylis lepida* and *Gudeodiscus francoisi*, the former is treated as a junior synonym of *Plectopylis francoisi*.

#### Gudeodiscus (Gudeodiscus) giardi giardi

Taxon classificationAnimaliaStylommatophoraPlectopylidae

(Fischer, 1898)

Figures 7E–F , 8A , 9I , 13L–U , 19 , 28B , 29E , 30D , 32C , 35A–C , 45A

**Figure 5.**
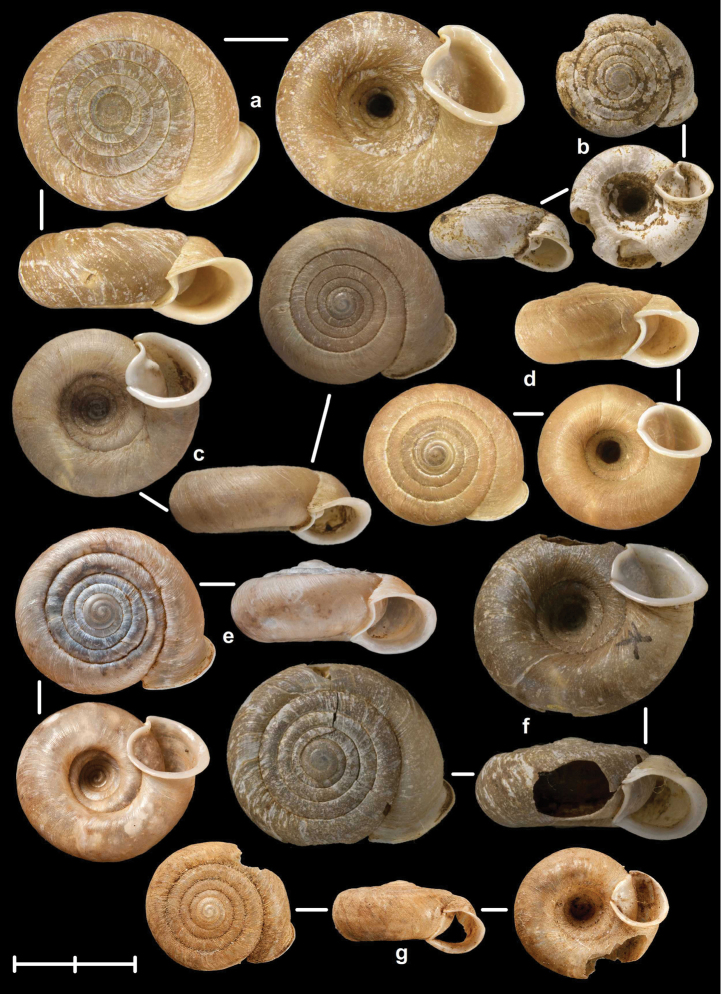
Shells of Vietnamese *Gudeodiscus* species. **A** Gudeodiscus (Gudeodiscus) phlyarius (Mabille, 1887) (typical “fallax var. major”), MNHN-IM-2012-2155 **B** Gudeodiscus (Gudeodiscus) phlyarius, NHMUK 1922.8.29.55 (holotype of *Plectopylis verecunda*) **C** Gudeodiscus (Gudeodiscus) phlyarius, NHMUK 1922.8.29.58 (holotype of *Plectopylis fallax*) **D** Gudeodiscus (Gudeodiscus) messageri raheemi Páll-Gergely & Hunyadi, ssp. n., Vn10-76, coll. PGB **E** Gudeodiscus (Gudeodiscus) messageri raheemi ssp. n., NHMUK 20110370.1 (holotype) **F** Gudeodiscus (Gudeodiscus) messageri messageri (Gude, 1909), NHMUK 1922.8.29.53 (holotype) **G** Gudeodiscus (Gudeodiscus) messageri messageri NHMUK 1922.8.29.54 (syntype of Plectopylis messageri var. minor). Photos: B. Páll-Gergely (**A, D**), H. Taylor (**B–C, E–G**). Scale represents 10 mm.

**Figure 6.**
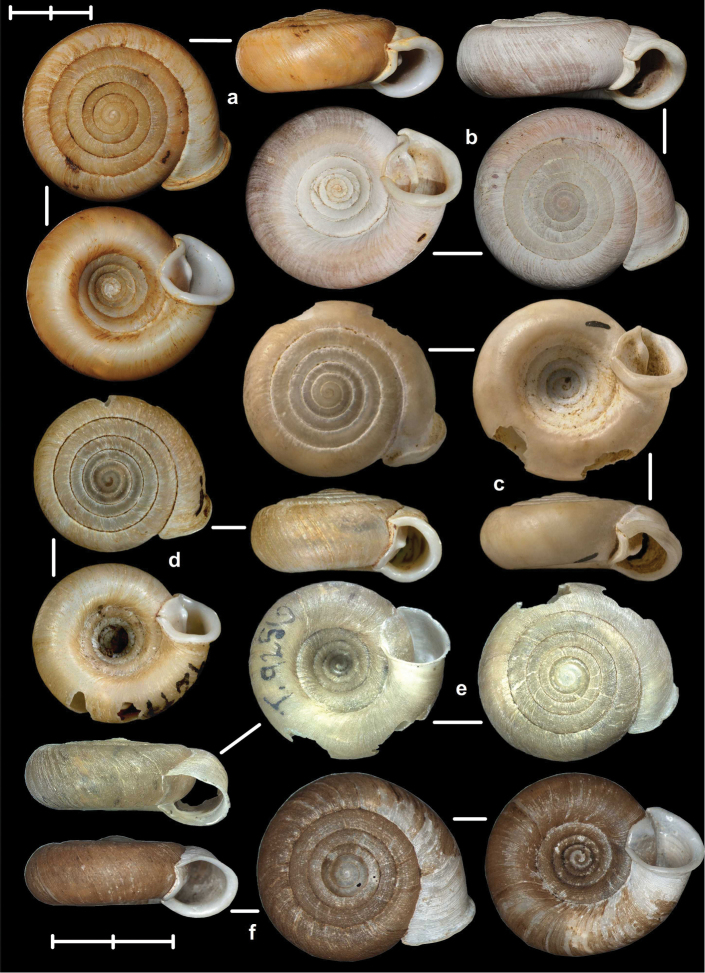
Shells of Vietnamese *Halongella* gen. n. and *Gudeodiscus* species. **A** *Halongella schlumbergeri* (Morlet, 1886), MNHN 24582 (syntype of Helix (Plectopylis) schlumbergeri) **B** *Halongella schlumbergeri*, MNHN 24580 (syntype of *Plectopylis jovia*) **C** *Halongella schlumbergeri*, NHMUK 1922.8.29.52 (holotype of *Plectopylis pilsbryana*) **D** *Halongella schlumbergeri*, SMF 9277 (lectotype of *Plectopylis hirsuta*) **E** Gudeodiscus (Veludiscus) emigrans emigrans (Möllendorff, 1901), SMF 9256 (lectotype) **F** Gudeodiscus (Veludiscus) emigrans quadrilamellatus Páll-Gergely, 2013, HNHM 97468 (holotype). Photos E and F were already published in Páll-Gergely and Hunyadi (2013). Photos: T. Deli (**A, B**), H. Taylor (**C**), S. Hof (**D**), E. Neubert (**E**) and B. Páll-Gergely (**F**). Scales represent 10 mm; upper scale belongs to **A–B**, lower scale belongs to **C–F.**

Plectopylis Giardi Fischer 1898a, Bulletin Biologique de la France et de la Belgique, 28: 320–322., Plate 17, Figs 17–21. [“Cao-Bang”].
Plectopylis Giardi Fischer 1898b, Journal de Conchyliologie, 46: 214–218., Figs 2, 5–6. [“rochers calcaires Déo-Ma-Phuc”].
Plectopylis Giardi Fischer 1899, Bulletin Biologique de la France et de la Belgique, 32: 330–332., Figs 2, 5–6.
Plectopylis giardi , — Gude 1899a, Science Gossip, 5: 332–333., Figs 95a–e [“Cao-Bang, Tonkin”].
Plectopylis congesta Gude 1899a, **syn. n.**, Science Gossip, 5: 332–333., Figs 96a–f [“Tonkin”, “Its exact locality, unfortunately, was not stated.”].
Plectopylis giardi , — Gude 1899b, Science Gossip, 6: 76., Fig. 103.
Plectopylis (Endoplon) giardi , — Gude 1899c, Science Gossip, 4: 148.
Plectopylis (Endoplon) congesta , — Gude 1899c, Science Gossip, 6: 148.
Plectopylis (Endoplon) giardi , — Gude 1899d, Science Gossip, 6: 175.
Plectopylis (Endoplon) congesta , — Gude 1899d, Science Gossip, 6: 175, 176.
Plectopylis congesta , — Gude 1901a, Journal de Conchyliologie, 49: 199, 202, 209, 211–212. [“Entre Bac-Kan, et Nac-Ri; environs de Bac-Kan; That-Khé”].
Plectopylis Giardi , — Gude 1908, Journal de Conchyliologie, 55: 346–348., Figs 1a–b [“Cao-Bang”, “Quang-Huyen”].
Gudeodiscus congestus , — Páll-Gergely & Hunyadi 2013, Archiv für Molluskenkunde, 142 (1): 8.
Gudeodiscus giardi giardi , — Páll-Gergely & Hunyadi 2013, Archiv für Molluskenkunde, 142 (1): 19–20., Figs 28, 53a–b, 58 (map).

##### Types examined.

Haut-Tonkin, Cao-Bang, leg. Billet, M., MNHN 9946 (2 syntypes of *giardi*, Figure 8A); Vietnam, Tonkin, environs de Bac-Kan, leg. Messager, MNHN IM-2010-12120 (syntype of *congesta*, Figure 8E); Vietnam, Tonkin, environs de Bac-Kan. leg. Messager, NHMUK 1922.8.29.49 (syntype of *congesta*, Figure 8F).

##### Museum material examined.

Tonkin, coll. Jetschin ex Bonnet 1900, SMF 341736/2; Tonkin, Möllendorff ex Fulton, SMF 150136/1; Tonkin, coll. Jetschin ex Berlier 1908, SMF 102817/1; Tonkin, environs de Bac-Kan, leg. Messager (n. 28), RBINS/1; Tonkin, Long-Phai, NHMSB 122815/1; Long-Phai, leg. Messager, 1901, MNHN-IM-2012-2231/13; Nga-Son, leg. Messager, MNHN-IM-2012-2235/1; Long-Phai, leg. Messager, 1901, MNHN-IM-2012-2236/16; Quang-Huyen, leg. Mansuy, MNHN-IM-2012-2238/14; Bac-Kan, leg. Messager, MNHN-IM-2012-2239/7; That-Khé, leg. Messager, MNHN-IM-2012-2240/9; Bac-Kan, leg. Messager, MNHN-IM-2012-2246/8; Quang-Huyen, Ha-Lang, Coll. Mansuy, MNHN-IM-2012-2248/14; That-Khé, coll. Letellier 1949, MNHN-IM-2012-2266/1; Than-Moi, coll. Staadt, 1969, MNHN-IM-2012-2278/1; Tonkin, coll. Letellier, 1949, MNHN-IM-2012-2293/1; Tonkin, coll. Mansuy, MNHN-IM-2012-2298/1; Entre Bac-Kan et Nac-Ri, coll. Lavezzari, 1929, MNHN-IM-2012-2302/6; Tonkin, coll. Letellier, 1949, MNHN-IM-2012-2308/1; Tonkin, coll. Levazzari, 1929, MNHN-IM-2012-2309/3; That-Khé, leg. Messager, MNHN-IM-2012-2310/6; Cao-Bang, leg. Messager, MNHN-IM-2012-2469/7; Tonkin, leg. Messager, MNHN-IM-2012-2460/9; Tonkin, leg. Messager, MNHN-IM-2012-2441/1; Halong Bay, leg. Messager, MNHN-IM-2012-2318/1; Halong Bay, leg. Messager, MNHN-IM-2012-2319/1; Halong Bay, leg. Messager, MNHN-IM-2012-2323/1; Tonkin, Bac-Kan, Na-Ri, leg. Messager, MNHN-IM-2012-2324/47; That Khé, leg. Messager, MNHN-IM-2012-2326/3; Po Ma, leg. Messager, MNHN-IM-2012-2328/7; That Khé, coll. Staadt 1969, MNHN-IM-2012-2330/3; That Khé, leg. Messager, MNHN-IM-2012-2341/28; Po Ma, leg. Messager, MNHN-IM-2012-2342/6; Col de Nuages, leg. Messager, MNHN-IM-2012-2343/4; Bac-Kan, leg. Messager, MNHN-IM-2012-2344/8; Tonkin, leg. Messager, MNHN-IM-2012-2345/8; That Khé, leg. Messager, MNHN-IM-2012-2346/5; Cold de Nuages, leg. Messager, MNHN-IM-2012-2349/4; Quang-Huyen, coll. Staadt, 1969, MNHN-IM-2012-2351/1; Tonkin, leg. Messager, MNHN-IM-2012-2352/10; Tonkin, leg. Messager, MNHN-IM-2012-2355/1; Na-Cham, leg. Messager, MNHN-IM-2012-2356/10; Na-Cham, leg. Messager, MNHN-IM-2012-2357/5; Cao-Bang, leg. Messager, MNHN-IM-2012-2359/4; That-Khé, leg. Messager, MNHN-IM-2012-2374/4; Tinh Tuc, secteur de Nguyen Binh, coll. Achat Boubée, MNHN-IM-2012-2385/1; Trinh-Thuong, leg. Messager, MNHN-IM-2012-2393/1; Tonkin, coll. Jousseaume, MNHN-IM-2012-2399/1; Bac-Kan, leg. Messager, MNHN-IM-2012-2432/1; Tonkin, leg. Messager, MNHN-IM-2012-2426/3; Bac-Kan, leg. Messager, MNHN-IM-2012-2435/1; Tonkin, coll. Lucas, Acc. no. 2351, NHMUK 20130604/2 (under the name “*persimilis*”); Tonkin, 3/10/08, NHMUK 20130605/2 (under the name “persimilis v. minor”); Tonkin, That-Khé, 3/10/08, NHMUK 20130606/3 (under the name “*persimilis*”); Tonkin, coll. Lucas, Acc. no. 2351, NHMUK 20130607/1; Tonkin, 27/6/00, 28, NHMUK 20130608/3 (“*congesta*”); Tonkin, Phi-Mi, coll. Salisbury ex Beddome, NHMUK 20130609/2 (“*congesta*”); Tonkin, coll. Kennard, A. S. ex Gude, NHMUK 20130610/1 (“*congesta*”); Tonkin, Quang-Huyen, NHMUK 1916.3.16.21/1; Tonkin, Quang-Huyen, NHMUK 1907.2.20.17–18/2; Haut-Tonkin, NHMUK 1904.8.1.1–2/2 (under the name “*persimilis*”); Tonkin, That-Khé, NHMUK 1900.2.13.221/1; Tonkin, That-Khé, NHMUK 1920.1.20.17/1; Tonkin, Long-Phai, coll. Wagner ex Messager, NHMW 71640/O/10289/1; Tonkin, Ngan-Son, coll. Wagner ex Messager, NHMW 71640/O/10288/1; Tonkin, Phi-Mi, NHMW 46023/2; Tonkin, Long-Phai, NHMW 46294/2; Tonkin, That-Khe, coll. Wagner ex Messager, NHMW 71640/O/10286/1; Tonkin, Po-Ma (?), coll. Wagner ex Messager, NHMW 71640/O/10287/1; Tonkin, Bac-Khuon, coll. Rolle, NHMW 103352/1 (mixed sample with *phlyarius*); Tonkin, Quang-Huyen, coll. Steenberg, ZMUC-GAS-1813/2.

##### New material examined.

**Vn10-58** Cao Bằng Province, ca. 31.5 km from Phục Hòa to Mã Phục (left off rd.), 22°42.212'N, 106°22.055'E, leg. Hemmen, Ch. & J., 20.3.2010., PGB/1; **Vn10-61** Cao Bằng Province, ca. 2 km from Quảng Uyên to Hạ Lang (right off rd.) 22°42.685'N, 106°27.232'E, leg. Hemmen, Ch. & J., 24.3.2010., PGB/2; **Vn10-59** Cao Bằng Province, ca. 30 km from Phục Hòa to Mã Phục (right off rd.), 22°41.787'N, 106°22.652'E, leg. Hemmen, Ch. & J., 23.3.2010., PGB/3; **Vn09-23** Cao Bằng Province, ca. 4.5 km from Mã Phục to Cao Bằng (NW of Cao Bằng), ca. 400 m, 22°42.814'N, 106°19.630'E, leg. Hemmen, Ch. & J., 16.10.2009., PGB/4; **Vn10-57** Cao Bằng Province, ca. 4.5 km from Mã Phục to Cao Bằng (left off rd.), 22°42.661'N, 106°19.627'E, leg. Hemmen, Ch. & J., 23.03.2010., PGB/3; **20081115D** Cao Bằng Province, Hòa An District, Nguyễn Huệ Commune, Hạ Lang, ca 390 m, 22°42.703'N, 106°19.606'E, leg. Ohara, K., 15.11.2008., OK/6, PGB/1; **20081116C** Cao Bằng Province, Trùng Khánh District, Cảnh Tiên Commune, Pắc Rảo., ca 545 m, 22°48.941'N, 106°30.549'E, leg. Ohara, K., 16.11.2008., OK/7, PGB/2; **Vn10-69** Cao Bằng Province, ca. 34.5 km from Cao Bằng to Đông Khê (left off new rd.), 22°27.439'N, 106°24.994'E, leg. Hemmen, Ch. & J., 26.03.2010. (typical “*congesta*”), PGB/3; **2011/81** Cao Bằng Province, Đèo Mã Phục (pass) 500 m towards Quảng Uyên, left side of the road, rock cavern, 610 m, 22°43.981'N, 106°20.333'E, leg. Hunyadi, A., 14.11.2011., HA/26, PGB/2; **2011/82** Lạng Sơn Province, Lũng Phầy (pass), Thất Khê N 13 km, 475 m, 22°20.363'N, 106°27.098'E, leg. Hunyadi, A., 15.11.2011., HA/8, PGB/1 (typical “*congesta*”); **2011/83** Cao Bằng Province, Đèo Lũng Phầy (pass) 2.5 km towards Đông Khê, right side of the road, 360 m, 22°21.654'N, 106°26.467'E, leg. Hunyadi, A., 15.11.2011., HA/17, PGB/2 (typical “*congesta*”); **2011/86** Cao Bằng Province, Quảng Uyên N, 206–207 cross, 300 m towards Hạ Lang, right side of the road, 445 m, 22°42.670'N, 106°27.260'E, leg. Hunyadi, A., 16.11.2011., HA/14, PGB/1; **2011/87** Cao Bằng Province, Quảng Uyên N, 206–207 cross, 430 m, 22°42.737'N, 106°27.223'E, leg. Hunyadi, A., 16.11.2011., HA/14, PGB/1 (anatomically examined, Figures 19, 28B, 29E, 30D, 32C, 35A–C); **2011/88** Cao Bằng Province, Quảng Uyên NW, 445 m, 22°42.562'N, 106°26.313'E, leg. Hunyadi, A., 16.11.2011., HA/6; **2011/89** Cao Bằng Province, Quảng Uyên W, Phi Hải-Ðầu Tuyền cross, 500 m, 22°42.188'N, 106°26.358'E, leg. Hunyadi, A., 16.11.2011., HA/5; **2011/90** Cao Bằng Province, Quảng Uyên S 2 km towards Hồng Ðịnh, left side of the road, 470 m, 22°40.761'N, 106°26.746'E, leg. Hunyadi, A., 16.11.2011., HA/1; **2012/42** Cao Bằng Province, Quảng Uyên 10 km towards Cao Bằng, left side of the road, 620 m, 22°42.772'N, 106°21.582'E, leg. Hunyadi, A., 27.05.2012., HA/9; **2012/43** Cao Bằng Province, Pắc Rảo, Cảnh Tiên Commune cross, 300 m towards Trùng Khánh, right side of the road, 530 m, 22°49.385'N, 106°30.742'E, leg. Hunyadi, A., 28.05.2012., HA/13; **2012/44** Cao Bằng Province, southern edge of Pắc Rảo, Trùng Khánh 3 km towards Quảng Uyên, left side of the road, 570 m, 22°48.961'N, 106°30.533'E, leg. Hunyadi, A., 28.05.2012., HA/35; **2011/85** Cao Bằng Province, Cao Bằng 34.5 km towards Đông Khê, left side of the road, 500 m, 22°27.487'N, 106°25.047'E, leg. Hunyadi, A., 15.11.2011., HA/35, PGB/5 (typical “*congesta*”); **2011/84** Cao Bằng Province, Đông Khê 3 km towards Đèo Lũng Phầy (pass), right side of the road, 390 m, 22°24.223'N, 106°25.937'E, leg. Hunyadi, A., 15.11.2011., HA/10, PGB/2 (typical “*congesta*”); Cao Bằng Province, Hòa An District, Nguyễn Huệ Commune, small hill just outside of Khau Trang Village, 22°33.510'N, 106°10.294'E, leg. Naggs, F. et al., 22.06.2011., NHMUK/1 (see Figure 45A).

##### Diagnosis.

Shell small to large, brownish (some Chinese populations are small and yellow, translucent), usually finely reticulated (resulting in a matt surface), umbilicus deep, dorsal side domed; apertural lip, callus and apertural fold very well-developed (Figure 9I). Parietal wall with two lamellae; the anterior one is usually connected to the lower plica; middle palatal plicae short, depressed Z-shaped, or almost vertical, sometimes connected to each other (Figures 13L–U).

##### Measurements

(in mm): D = 13.5–14.1, D = 7–7.7 (n=2, 2011/84); D = 15.6–17, H = 7.7–10 (n=2, 2011/85); D = 19.9–20.3, H = 11–11.6 (n=2, 2011/81); D = 21.3, H = 12.1 (n=1, 2011/86).

##### Differential diagnosis.

This species is most similar to *Gudeodiscus francoisi*. For comparisons, see under that species. *Gudeodiscus dautzenbergi* is larger, flatter, has wider umbilicus, a weaker apertural lip and the lower end of the anterior lamella is very much elongated anteriorly. *Gudeodiscus villedaryi* is also flatter and most populations have a keel around the umbilicus and an additional long plica below the parietal lamellae. *Gudeodiscus phlyarius* is usually flatter, has a wider umbilicus, slimmer peristome and lower callus. Most specimens of *Gudeodiscus phlyarius* have separated anterior lamella and lower plica, whereas these are always connected in *Gudeodiscus giardi giardi*. Typical *Plectopylis verecunda* shells (synonym of *Gudeodiscus phlyarius*) also have an elevated spire, but their shell shape is rather conical, whereas it is usually domed (rounded) in *Gudeodiscus giardi*.

##### Intraspecific diversity.

Two subspecies of *Gudeodiscus giardi* were described from China (see Páll-Gergely and Hunyadi 2013). The populations assigned to the nominotypical subspecies show larger variability in China in terms of shell size, colour and shape, than in Vietnam. In Vietnam *Gudeodiscus giardi giardi* is moderately variable. Most variability is observable in the formation of the parietal plicae and lamellae (see Remarks and Figures 13L–U).

##### Description of the genitalia.

One specimen was anatomically examined (see also Remarks). Locality: Cao Bằng Province, Quảng Uyên N, 206–207 cross, 430 m, 22°42.737'N, 106°27.223'E, leg. Hunyadi, A., 16.11.2011. (Figure 28B, 29E, 30D, 32C).

Penis very short, almost ball-like; penis wall conspicuously thickened, its inner surface is characterized by transversal lines at the proximal part and longitudinal pockets in the distal part, arranged in a straight row (Figure 28B); there are some calcareous, claw-like objects in the pockets; the claws have a wide, rounded basal part which is found within the pockets, and the short, hook-like part hangs out of the pockets; the base had a granulated surface, probably to provide a better attachment to wall of the pockets, whereas the tip was smooth (Figure 30D); epiphallus C-shaped, longer than the penis; its inner wall with three longitudinal parallel folds (Figure 29E); penis and epiphallus connected with weak membrane; penial caecum approximately as long as the penis; it has low tubercles on the inner wall and small calcareous rounded granules on each tubercle; retractor muscle attaches on the distal part of the penial caecum; it is longer and wider than the caecum; vas deferens convoluted near the vagina. Vagina very thick and long, it is attached to the body wall with several thin ligaments; one side of the vaginal bulb with very much thickened wall, the other side with thin, almost translucent wall, internally with fine, irregular, reticulated sculpture; inner wall of the distal portion of the vagina with well-developed, rather irregular transversal folds (Figure 32C); gametolytic sac and diverticulum slender, they are nearly the same length.

##### Radula.

See Table 6 and Figures 35A–C.

##### Distribution

(see Figure 42): Newly-collected material was examined from Cao Bằng Province and the northern part of Lạng Sơn Province. The localities of “Col de Nuages” and “Halong Bay” are probably erroneous. This species is also known from the western part of Guangxi, China (Páll-Gergely and Hunyadi 2013).

##### Remarks.

*Plectopylis congesta* Gude, 1899 was described without exact locality data. Some shells from populations in southern Cao Bằng and northern Lạng Sơn prefectures (Vn10-69; 2011/84, 2011/83, 2011/82, 2011/85) resemble the holotype of *Plectopylis congesta* on the basis of relatively weak peristome and callus, weak (low) posterior lamella and the anterior lamella which is fused to the upper parietal plica. These populations however, falls within the morphological range of the very variable *Gudeodiscus giardi giardi*, therefore *Plectopylis congesta* is here synonymised with *Gudeodiscus giardi giardi*.

The genital anatomy of a Chinese specimen of *Gudeodiscus giardi giardi* was described by Páll-Gergely and Asami (2014). The only notable difference between the Chinese and Vietnamese specimens is the much longer penis in the Chinese individual. It seems that the long, slender, proximal portion of the penis visible in the Chinese specimen is entirely missing in the Vietnamese one.

#### Gudeodiscus (Gudeodiscus?) hemmeni

Taxon classificationAnimaliaStylommatophoraPlectopylidae

Páll-Gergely & Hunyadi sp. n.

http://zoobank.org/5A98BC18-CF82-4C2F-BCE4-DCCA8DBBED3B

Figures 2C–D , 9F , 11G–J

##### Type material.

**2012/61** Sơn La Province, Hà Nội 156 km towards Mộc Châu, left side of the road nr. 6, rocky wall, 1110 m, 20°45.993'N, 104°53.868'E, leg. Hunyadi, A., 06.06.2012., holotype HNHM 97458 (Figure 2C), HA/11 paratypes+4jb (not paratype), PGB/3 paratypes; **2012/62** Sơn La Province, Hà Nội 156 km towards Mộc Châu, right side of the road nr. 6, rocky wall, 1110 m, 20°46.085'N, 104°53.888'E, leg. Hunyadi, A., 06.06.2012., HA/13 paratypes+2jb (not paratypes), PGB/1; **Vn12-104** Sơn La Province, right side off road Mộc Châu to Sơn La, 20°52.567'N, 104°35.310'E, leg. Hemmen, Ch., 02.10.2012., HE/1; **Vn10-103A** Hòa Bình Province, ca. km 156 old road Hà Nội to Sơn La (right side off road), 20°46.000'N, 104°53.885'E, leg. Hemmen, Ch. & J., 15.10.2010., HE/1 (Figure 2D); **Vn10-76A** Sơn La Province, ca. 32 km from Mộc Châu to Hà Nội (old road), 20°47.351'N, 104°50.063'E, leg. Hemmen, Ch. & J., 02.04.2010., HE/1.

##### Diagnosis.

Shell small, with slightly elevated spire, characteristically shaped aperture having wide upper sinulus and small apertural fold (Figure 9F); parietal wall with two lamellae and horizontal plicae above and below; palatal plicae depressed Z-shaped; free from each other, or connected to each other with a ridge (Figures 11G–J).

##### Description.

Shell very small to small, light brown to chocolate brown, with slightly elevated spire, consists of 5.25–5.5 whorls; suture relatively shallow, especially at the first 3–4 whorls; protoconch (2.25–2.5 whorls) glossy, very finely, regularly ribbed, but the ribs are sometimes hardly visible, they are more prominent at the upper part of the whorls, close to the suture; teleoconch without notable spiral lines, very finely regularly ribbed; sculpture strength equal on ventral and dorsal side; umbilicus narrow and deep; aperture with widened upper part (sinulus), apertural lip whitish, thin, slightly expanded but not reflexed; apertural denticle (fold) always present, very small, free from the callus or connected to it.

Two specimens were opened. Parietal side with a stronger anterior lamella with anteriorly widened lower part, and a slimmer posterior lamella; shorter upper and longer lower horizontal plicae free from the anterior lamella, the lower one a bit extends beyond the anterior lamella in the anterior direction. Palatal side with six plicae; first and last are straight, the others are depressed Z-shaped and are connected with a ridge.

##### Measurements

(in mm): D = 9.5–10.1, H = 4.3–5.2 (n=5, belonging to different populations).

##### Differential diagnosis.

*Gudeodiscus hemmeni* sp. n. differs from most *Gudeodiscus phlyarius* populations by the smaller shell, shorter denticle (fold) in the aperture, thinner apertural lip, the wider and reflexed apertural rim, the wide upper sinus of the aperture, lack of spiral lines in the sculpture and narrower umbilicus. *Gudeodiscus anceyi* is usually smaller, has a longer apertural fold, prominent spiral sculpture, a weaker callus and differently shaped aperture.

In all localities, *Gudeodiscus hemmeni* sp. n. lives sympatrically with *Gudeodiscus messageri raheemi* ssp. n., which is much larger, lacks the apertural fold, and usually has an anterior lamella which is dissolved into small denticles.

##### Intraspecific diversity.

Low; shell characters are stable, although only a few shells are known.

##### Etymology.

The new species is dedicated to Jens Hemmen (1944–2012), malacologist and much-valued friend, who contributed to our revision by providing shell and ethanol-preserved material.

##### Type locality.

Sơn La Province, Hà Nội 156 km towards Mộc Châu, left side of the road nr. 6, rocky wall, 1110 m, 20°45.993'N, 104°53.868'E.

##### Distribution

(see Figure 43). The new species is known from few locations in south-eastern Sơn La province.

#### Gudeodiscus (Gudeodiscus?) infralevis

Taxon classificationAnimaliaStylommatophoraPlectopylidae

(Gude, 1908)

Figures 3D–E , 15A–D

Plectopylis infralevis Gude 1908, Journal de Conchyliologie, 55: 345, 350, 352–353., Figs 3a–e, Plate 7, Figs 4–6. [“Quang Huyen”].
Plectopylis soror Gude 1908, **syn. n.**, Journal de Conchyliologie, 55: 355–357., Figs 5a–e, Plate 7, Figs 10–12. [“Quang Huyen”].
Gudeodiscus infralevis , — Páll-Gergely & Hunyadi 2013, Archiv für Molluskenkunde, 142 (1): 8.
Gudeodiscus soror , — Páll-Gergely & Hunyadi 2013, Archiv für Molluskenkunde, 142 (1): 8.

##### Types examined.

Tonkin, Quang-Huyen, leg. Mansuy, MNHN 24604 (holotype of *infralevis*, Figure 3D); Tonkin, Quang-Huyen, leg. Mansuy, MNHN 24585 (holotype of *soror*, Figure 3E).

##### Diagnosis.

Shell small, solid, discoid, with elevated spire, relatively deep umbilicus; relatively thin apertural lip and rather parallel, thick, straight palatal plicae. See also under remarks.

##### Measurements

(in mm): D = 13.9, D = 6.7 (*soror* holotype); D = 13.5, H = 6.6 (*infralevis* holotype).

##### Differential diagnosis.

Our knowledge on the intraspecific variety of the species is very limited (see Remarks). It seems that the thick, rather horizontal palatal plicae, the strong basal sculpture and the elevated spire distinguishes the species from the similar species (*Gudeodiscus eroessi*, *Gudeodiscus multispira*, *Gudeodiscus soosi*, *Gudeodiscus yunnanensis*, *Gudeodiscus cyrtochilus* and *Gudeodiscus fischeri*). The shell and aperture shape suggest that the closest relatives are *Gudeodiscus fischeri* and *Gudeodiscus suprafilaris* (see comparisons under those species).

##### Intraspecific diversity.

*Plectopylis infralevis* and *Plectopylis soror* are considered as conspecific (see Remarks). Only the holotypes of these taxa are known, therefore our knowledge on the intraspecific variability is limited.

##### Distribution.

The type specimens of *Plectopylis infralevis* and *Plectopylis soror* (synonym of *infralevis*) were collected in Quang Huyen (Quảng Uyên) (see Figure 39).

##### Remarks.

Only the holotypes of *Plectopylis infralevis* and *Plectopylis soror* are known. The notable differences between these two shells are the stronger sculpture, slightly shouldered body whorl and small apertural fold in *soror*. Additionally, there are three lamellae in *infralevis* versus only one in *soror*. The three vertical lamellae in the holotype of *infralevis* is possibly the result of abnormal development. No other species of Plectopylidae has three lamellae. Similar abnormal shells have been reported in *Gudeodiscus giardi* (see Gude 1908). Consequently, we do not know what the characteristic type of parietal lamellae in this species is (=one or two). The differences between the two specimens suggest only intraspecific variance. Unfortunately we have no freshly-collected material of these two forms, but because of the high similarity between the two holotypes and same type locality we here synonymise *soror* with *infralevis*. These two names were published in the same paper (Gude 1908), but *infralevis* was described earlier in terms of page numbers.

#### Gudeodiscus (Gudeodiscus) messageri

Taxon classificationAnimaliaStylommatophoraPlectopylidae

(Gude, 1909)

##### Diagnosis.

Shell small to medium-sized, with slightly elevated spire, dorsal surface somewhat domed; aperture almost circular, apertural fold missing; callus rather blunt and only slightly curved. Parietal wall with two lamellae (the anterior lamella may be dissolved into small denticles); lower parietal plica free or connected to the anterior lamella; palatal plicae oblique, or depressed Z-shaped, usually in contact with each other.

##### Differential diagnosis.

See under the two subspecies.

#### Gudeodiscus (Gudeodiscus) messageri messageri

Taxon classificationAnimaliaStylommatophoraPlectopylidae

(Gude, 1909)

Figures 5F–G , 9E , 12N–Q

Plectopylis messageri Gude 1909, Proceedings of the Malacological Society of London, 8: 214–215., Plate 9, Figs 4, 4a–b [“Moung-Hum”, “Nat-Son, Pac-Kha, and Trinh-Tuong”].
Gudeodiscus messageri , — Páll-Gergely & Hunyadi 2013, Archiv für Molluskenkunde 142 (1): 8.

##### Types examined.

Tonkin, Muong-Hum, leg. Messager, NHMUK 1922.8.29.53 (holotype of *Plectopylis messageri*, Figure 5F), Tonkin, Nat-Son, leg. Messager, NHMUK 1922.8.29.54 (holotype of messageri var. minor, Figure 5G).

##### Museum material examined.

Tonkin, coll. Dosch ex Rolle ex Messager, SMF 172088/4; Tonkin, coll. Dosch ex Rolle, SMF 172076/2; Tonkin, Trinh-Tuong, coll. Dosch ex Rolle, SMF 172086/4; Tonkin, Drinch-Tuom (Trinh-Thuong?), coll. Jaeckel ex Messager, SMF 207675/3; Tonkin, alw. Müller, coll. Kaltenbach, SMF 294867/2; Tonkin, Gia-Phu, coll. Dosch ex Rolle, SMF 172089/4; Tonkin, Muong-Bo, coll. Dosch ex Rolle, SMF 172087/4; Tonkin, Muong-Kong, coll. Pfeiffer, K. L. ex Naschloss (?) ex Rolle, January 1938, SMF 102820/1; Tonkin, coll. Dosch ex Rolle ex Messager, SMF 182088/4; Tonkin, Ba-Nat (?), NHMSB 131/200, 122812-122813/2; Pakhé, leg. Messager, MNHN-IM-2012-2129/9; Muong-Hum, leg. Messager, MNHN-IM-2012-2134/15; Nat-Son, Trinh-Thuong, leg. Messager, MNHN-IM-2012-2136/16 („var. *minor*“); Muong-Kong, leg. Messager, MNHN-IM-2012-2137/4; Trinh-Thuong, leg. Messager, MNHN-IM-2012-2142/2+4jb; Muong-Hum, leg. Messager, MNHN-IM-2012-2131/5; Muong-Hum, leg. Messager, MNHN-IM-2012-2143/3; Muong-Hum, leg. Messager, MNHN-IM-2012-2145/74; Pakhé, leg. Messager, MNHN-IM-2012-2149/1; Pac-Kha (Pakhé), leg. Messager, MNHN-IM-2012-2151/10; Nat-Son, leg. Messager, MNHN-IM-2012-2154/6; Muong-Kong, leg. Messager, MNHN-IM-2012-2159/1; Nat-Son, leg. Messager, MNHN-IM-2012-2162/29; Trinh-Thuong, leg. Messager, MNHN-IM-2012-2163/20; Nat-Son, leg. Messager, MNHN-IM-2012-2165/8+25jb; Bac-Kan, leg. Messager, MNHN-IM-2012-2166/6; Bac-Kan, leg. Messager, MNHN-IM-2012-2172/4; Muong-Hum, leg. Messager, MNHN-IM-2012-2173/3; Muong-Hum, leg. Messager, MNHN-IM-2012-2183/4; Pakhé, leg. Messager, MNHN-IM-2012-2184/1; Long-Ping, leg. Messager, MNHN-IM-2012-2186/8; Muong-Hum, leg. Messager, MNHN-IM-2012-2188/8; Bac-Kan, leg. Messager, MNHN-IM-2012-2194/3; Muong-Hum, leg. Messager, MNHN-IM-2012-2196/4; Nat-Son, leg. Messager, MNHN-IM-2012-2198/2; Nat-Son, leg. Messager, MNHN-IM-2012-2199/2; Tonkin, leg. Messager, MNHN-IM-2012-2202/1; Trinh Thuong, leg. Messager, MNHN-IM-2012-2205/12; Muong-Kong, leg. Messager, MNHN-IM-2012-2479/1; Bac-Kan, leg. Messager, MNHN-IM-2012-2475/10; Trinh-Thuong, leg. Messager, MNHN-IM-2012-2472/16; Cao-Bang, leg. Messager, MNHN-IM-2012-2471/1; Tonkin, Pakhé, leg. Messager, MNHN-IM-2012-2458/7; Long-Ping, leg. Messager, MNHN-IM-2012-2457/23; label not readable, leg. Messager, MNHN-IM-2012-2449/2; Bac-Kan, leg. Messager, MNHN-IM-2012-2403/1; Trinh-Thuong, coll. Levazzari, 1929, MNHN-IM-2012-2408/9; Tonkin, coll. Staadt, 1969, MNHN-IM-2012-2411/3; Nat-Son, coll. Letellier, 1949, MNHN-IM-2012-2414/2; Pac-Kha, coll. Letellier, 1949, MNHN-IM-2012-2415/2; Gia-Phu, MNHN-IM-2012-2418/3; Trinh-Thuong, coll. Lavezzari, 1929, MNHN-IM-2012-2419/10; Tonkin, leg. Messager, MNHN-IM-2012-2425/3; Gia-Phu, leg. Messager, MNHN-IM-2012-2215/33; Muong-Hum, leg. Messager, MNHN-IM-2012-2216/3; Long-Ping, leg. Messager, MNHN-IM-2012-2217/9; Trinh-Thuong, leg. Messager, MNHN-IM-2012-2219/12; Col de Nuages, leg. Messager, MNHN-IM-2012-2221/1; Trinh Tuong, leg. Messager, MNHN-IM-2012-2223/2; Tonkin, leg. Messager, MNHN-IM-2012-2225/4; Tonkin, leg. Messager, MNHN-IM-2012-2230/1; Long-Phai, leg. Messager, 1901, MNHN-IM-2012-2237/2; Muang-Kong, leg. Messager, MNHN-IM-2012-2242/3; Nat-Son, coll. Staadt, 1969, MNHN-IM-2012-2282/1; Tonkin, leg. M. Balansa, 1889 July, MNHN-IM-2012-2296/10; Pakhé, leg. Messager, MNHN-IM-2012-2339/1; Bac-Kan, leg. Messager, MNHN-IM-2012-2315/1; Gia-Phu, leg. Messager, MNHN-IM-2012-2364/2; Trinh-Thuong, leg. Messager, MNHN-IM-2012-2379/1; Trinh-Thuong, leg. Messager, MNHN-IM-2012-2394/1; Tonkin, Pac-Kha, NHMUK1916.3.16.15/1; Tonkin, Pac-Kha, coll. Kennard, A.S. ex auct. (Gude), NHMUK 20130620.2/1; Tonkin, Muong-Hum, coll. Biggs, H.E.J. ex Gygngell, 1930, Acc. no. 2258, NHMUK 20130626/2; Tonkin, Gia-Phu, coll. Kennard, A.S. ex auct. (Gude), NHMUK 20130627/2; Tonkin, Muong-Kong, coll. Salisbury ex Beddome, NHMUK 20130628/2; Tonkin, Muong-Kong, 31/3/09, NHMUK 20130629/3; Tonkin, Muong-Hum, 5/1/09, NHMUK 20130630/3; Tonkin, Pac-Kha, 3/11/08, NHMUK 20130631/2 (“var. *minor*”); Tonkin, 5/1/09, NHMUK 20130632/3; Tonkin, Muong-Hum, coll. Preston, NHMUK 20130633/2; Tonkin, Muong-Bo, 3/11/08, NHMUK 20130634/2 (“var. *major*”); Tonkin, That-Khé, coll. Salisbury ex Beddome, NHMUK 20130635/1; Tonkin, Muong-Hum, coll. Kennard, NHMUK 20130636/1; Tonkin, Muong-Hum, NHMUK 1909.3.17.29–31/3; Tonkin, Muong-Hum, NHMUK 1916.3.16.16-18/3; Tonkin, Pac-Kha, NHMUK 1909.3.17.32–34/3 (“var. *minor*”); Tonkin, Pac-Kha, NHMUK 1909.3.17.24–25/2; Tonkin, Muong-Bo, NHMUK 1909.3.17.35–36/2 (“var. *major*”); Tonkin, Gia-Phee, coll. Rušnov ex Rolle ex Messager, NHMW 92576/1; Tonkin, Trisch-Tuong, coll. Edlauer ex Werner, NHMW 75000/E/7983/2; Tonkin, Muong-Hum, coll. Oberwimmer ex Wagner ex Messager, NHMW 92573/2; Tonkin, Bac-Kan, coll. Oberwimmer, NHMW 71640/O/14028/1; Tonkin, Long-Ping, 3000 m, coll. Oberwimmer ex Wagner ex Messager, NHMW 92572/1; Tonkin, Muong Hum, coll. Rosen ex Messager, NHMW 71640/O/9476/2; Tonkin, Trinh-Tua (?), coll. Rolle, NHMW 92574/2; Tonkin, Ban-Tao, coll. Rušnov ex Blume, NHMW 92575/1; Tonkin, Muong Kong, NHMW 71640/O/46293/2; Tonkin, Nat-Son, coll. Rosen ex Messager, NHMW 71640/O/9477/1; Tonkin, Trisch Tuong, coll. Rušnov ex Rolle ex Messager, NHMW 92578/2; Tonkin, Ban-Lao, coll. Rolle, NHMW 92577/1; Tonkin, Bac-Kan, coll. Oberwimmer, NHMW 92567; Tonkin, Bac-Kan, coll. Oberwimmer, NHMW 103353/1; Tonkin, Nat-Son, coll. Rušnov ex Messager, NHMW 103355/1; Vietnam/132, Lao Cai Province, Cox-Xan, 400 m, leg. Topál & Matskási, 27.11.1971., VA/10.

##### Diagnosis.

At least one shell was opened of every larger samples. Anterior lamella normal (not dissolved into small denticles); lower parietal plica does not extend beyond the anterior lamella in the anterior direction (Figures 12N–Q).

##### Measurements

(in mm). D = 12.75–18.5 (according to the original description).

##### Differential diagnosis.

*Gudeodiscus messageri messageri* inhabits northern Vietnam and in many museum samples it is mixed with *Plectopylis gouldingi* or *Plectopylis fallax* (synonyms of *Gudeodiscus phlyarius*). These two forms have flat shells with a sharp and angled callus, and sometimes with an apertural denticle. Also, the aperture of *Gudeodiscus messageri* is rather rounded, whereas it is rather elongated in those populations of *Gudeodiscus phlyarius* (Figures 9D: *phlyarius*, Figure 9E: *messageri*). This allows *Gudeodiscus messageri* and *Gudeodiscus phlyarius* to be distinguished without breaking the shell. The lower parietal plica, which does not extend beyond the anterior lamella in the anterior direction, is characteristic of *Gudeodiscus messageri messageri* (see also Remarks), but almost always extends in “*Plectopylis fallax*” and “*Plectopylis gouldingi*”. “*Plectopylis verecunda*” (synonym of *Gudeodiscus phlyarius*) and typical *Gudeodiscus phlyarius* always have a strong apertural fold. Moreover, the lower parietal plica of the latter usually extends beyond the anterior lamella in the anterior direction. For comparison with *Gudeodiscus messageri raheemi* ssp. n., see there.

##### Intrasubpecific diversity.

Low; the shell size, and the relationship between the lower parietal plica and the anterior lamella show some variability (see remarks). The shell and aperture shape are stable characters.

##### Distribution

(see Figure 43): Only museum material was available for study, which suggested that this species is located along the Chinese (Yunnan) border.

##### Remarks.

In one sample (MNHN-IM-2012-2215) a specimen had longer lower plica which extended beyond the anterior lamella in the anterior direction.

#### Gudeodiscus (Gudeodiscus) messageri raheemi

Taxon classificationAnimaliaStylommatophoraPlectopylidae

Páll-Gergely & Hunyadi ssp. n.

Figures 5D , 5E , 10A , 12R–V , 20 , 28E , 29F–G , 31B , 35D–F

##### Type material.

Thanh Hoa Province, Cam Thuy District, Fish Stream, leg. Naggs, F. & Hao, L.V., 13.05.2008., NHMUK 20110370.1–3 (holotype and two paratype); **MAA10** Ninh Bình Province, Cúc Phương Nat. Park, path to fairy cave, approximate GPS position: 20°21'N, 105°54'E, leg. Vermeulen, J., coll. Maassen, W.J.M., 10.10.1998., PGB/1 paratype, WM/3 paratypes; **MAA1** Thanh Hóa Province, Pù Luông Nat. Park, NW corner of park near Hang village, limestone area near village, 20°31.84'N, 105°04.76'E, coll. Maassen, W.J.M., 19.09.2003., PGB/1 paratype, WM/3 paratypes; **MAA9** Thanh Hóa Province, Pù Luông Nat. Park, limestone hill opposite village Naca, 20°26.86'N, 105°11.57'E, coll. Maassen, W.J.M., 20.09.2003. WM/2 paratypes; **Vn10-76A** Sơn La Province, ca. 32 km from Mộc Châu to Hà Nội (old road), 20°47.351'N, 104°50.063'E, leg. Hemmen, Ch. & J., 07.10.2010., HE/1 paratype, PGB/2 paratypes; same locality data, leg. Hemmen, Ch., 01.10.2012., HE/1 paratype; **Vn10-103** Hòa Bình Province, ca. km 156 old road Hà Nội to Sơn La (right side off road), 20°46.000'N, 104°53.885'E, leg. Hemmen, Ch. & J., 15.10.2010., HE/2 paratypes, PGB/1 paratype, and one paratype in ethanol (anatomically examined, Figure 20); **20080509C** Nghệ An Province, Pù Mát Nat. Park, Con Cuông Dist., Lục Dạ Commune, Tân Hợp Village, ca 90 m, 18°57.80201'N, 104°54.67774'E, leg. Ohara, K., 09.05.2008., OK/5 paratypes, PGB/2 paratypes; **20071118A** Thanh Hóa Province, Trang Village, Bá Thước. (Bee Cave Mt.), Lân Sa Commune, ca 40 m, 20°19.92147'N, 105°12.49178'E, leg. Ohara, K., 2007.11.18., PGB/1 paratype; **20071118B** Thanh Hóa Province, Cây Đăng Cave, Lương Ngọc, Cẩm Lương C., (GPS not recorded), leg. Ohara, K., 18.11.2007., PGB/1 paratype; **20071116C** Ninh Bình Province, Cúc Phương Nat. Park, Cave of Prehistoric Man, ca 145 m, 20°15.53843'N, 105°42.38950'E, leg. Ohara, K., 16.11.2007., PGB/2 paratypes; **Vn10-104B** Sơn La Province, right side off road Mộc Châu to Sơn La, 20°52.567'N, 104°35.310'E, leg. Hemmen, Ch., 02.10.2012., HE/7 paratypes; same data, leg. Hemmen, Ch. & J., 08.10.2010., PGB/2 paratypes; same data, leg. Hemmen, Ch. & J., 14.10.2011., HE/17 paratypes; **2011/106** Ninh Bình Province, Cúc Phương Nat. Park, main entrance, 700 m towards Bống Village, 155 m, 20°15.231'N, 105°42.639', leg. Hunyadi, A., 22.11.2011., HA/12 paratypes +1jb (not paratype), PGB/2 paratypes; **2011/108** Ninh Bình Province, Cúc Phương Nat. Park, Ðộng Người Xưa (=Prehistoric Men Cave), around the cave, 20°17.615'N, 105°40.115'E, leg. Hunyadi, A., 23.11.2011., HA/6 paratypes; **2011/113** Thanh Hóa Province, Cẩm Lương, Ðộng Cây Ðăng (cave), around the cave, 60 m, 20°15.128'N, 105°23.404'E, leg. Hunyadi, A., 25.11.2011., HA/6 paratypes +5jb (not paratypes); **2012/10** Nghệ An Province, Con Cuông 20 km towards Anh Sơn, right side of the road, 40 m, 18°58.302'N, 105°00.796'E, leg. Hunyadi, A., 15.05.2012., HA/7 paratypes +11jb (not paratypes); **2012/60** Sơn La Province, Mộc Châu 5 km towards Sơn La, right side of the road nr. 6, 755 m, 20°52.551'N, 104°35.318'E, leg. Hunyadi, A., 06.06.2012., HA/6 paratypes +16jb (not paratypes), PGB/1 paratypes; **2012/61** Sơn La Province, Hà Nội 156 km towards Mộc Châu, left side of the road nr. 6, 1100 m, 20°45.993'N, 104°53.868'E, leg. Hunyadi, A., 06.06.2012., HA/3 paratypes +2jb (not paratypes); **2012/62** Sơn La Province, Hà Nội 156 km towards Mộc Châu, right side of the road nr. 6., rocky wall, 1110 m, 20°46.085'N, 104°53.888'E, leg. Hunyadi, A., 06.06.2012., HA/5 paratypes+2jb (not paratypes), PGB/1 paratype; **Vn12-80A** Thanh Hóa Province, Cẩm Thạch, opp. Cẩm Lương Fishstream (W Cẩm Thủy), 20°15.234'N, 105°23.530'E, leg. Hemmen, Ch., 08.10.2012., HE/5 paratypes; same data, leg. Hemmen, Ch. & J., 04.04.2010., HE/2 paratypes; **Vn11-215** Sơn La Province, ca. 34 km from Mộc Châu to Mai Châu, 20°45.219'N, 104°54.458'E, leg. Hemmen, Ch. & J., 15.10.2011., HE/1 paratype; **Vn11-230** Nghệ An Province, ca. 1.2 km left off rd 48, ca 23 km from Thái Hòa to Qùy Châu, 19°24.363'N, 105°26.521'E, leg. Hemmen, Ch. & J., 22.10.2011., HE/1 paratype; **Vn12-268** Thanh Hóa Province, km 585 on road 15 Yến Cát to Ngọc Lặc 1 km right off road 15, 19°45.589'N, 105°25.521'E, leg. Hemmen, Ch. & J., 14.04.2012., HE/3 paratype; **20080510A** Nghệ An Province, Pù Huống Nature Reserve, Con Coung District, Anh Son, Hoi Son, ca 30 m, 18°57.11872'N, 105°02.63029'E, leg. Ohara, K, Okubo, K & Otani, J. U., Sang, 10.05.2008., 1 paratype in ethanol, anatomically examined.

##### Diagnosis.

Anterior lamella normal or dissolved into small denticles, if normal, the lower plica extends beyond the anterior lamella in the anterior direction (Figures 12R–V).

##### Description.

Shell medium in size, light to dark brown or dark yellowish, sometimes almost flat but usually with slightly elevated spire, consists of 6.25–6.75 whorls; suture relatively shallow; protoconch (2.5–2.75 whorls) glossy, very finely, regularly ribbed; teleoconch very finely, rather irregularly ribbed, spiral lines visible mainly at the dorsal side where sometimes they are as strong as the ribs (resulting in a reticulated surface), in some specimens however hardly any spiral lines are visible; sculpture weaker on the ventral side but within the umbilicus are as strong as on the dorsal side; umbilicus relatively narrow and deep; aperture wide with whitish or light brown, thickened and reflexed apertural rim; callus slightly S-shaped, well-developed, with upper and with or without lower canal between the ends of callus and the apertural lip; apertural fold always missing.

More than ten specimens were opened belonging to different populations. Parietal side with two lamellae and upper and lower horizontal plicae above and below the anterior lamella; the lower plica usually extends beyond the anterior lamella in the anterior direction; in some populations the anterior lamella (or only the upper part of the lamella) is dissolved into several denticles. Palatal wall with six plicae; first and last are short and relatively straight, the four middle plicae are usually depressed Z-shaped and in many cases connected to each other with a ridge.

##### Measurements

(in mm). D = 12.9–14.4, H = 6.2–7.5 (n=3, Vn10-76); D = 14.2–14.4, H = 6.8–7.9 (n=3, 20071116C); D = 12.1, H = 6 (n=1, Vn11-230); D = 16–17.9, H = 7.3–7.9 (n=3, Vn11-104).

##### Differential diagnosis.

The lower parietal plica extends beyond the anterior lamella in the anterior direction, which is extremely rarely the case in the nominotypical subspecies. The anterior lamella was dissolved into small denticles in many samples, which has never been observed in the nominotypical subspecies (Figures 12N–Q: *messageri*, 12R–V: *raheemi* ssp. n.). The umbilicus of the new subspecies is narrower, it has more rounded whorls and a sharper, more angled callus, than in most samples of *Gudeodiscus messageri messageri*.

*Gudeodiscus messageri raheemi* ssp. n. lives sympatrically with an atypical form of *Gudeodiscus phlyarius* in Ninh Bình Province (see under *Gudeodiscus phlyarius*). *Gudeodiscus phlyarius* is flat and has an apertural fold, whereas *Gudeodiscus messageri raheemi* ssp. n. has somewhat elevated spire and always lacks the apertural fold. See also under *Gudeodiscus hemmeni* sp. n.

##### Intrasubspecific diversity.

Relatively variable; the colour, spire height, size and morphology of the palatal and parietal lamellae and plicae show considerable variability (see Table 8).

**Table 8.** Diversity of shell characters within Gudeodiscus (Gudeodiscus) messageri raheemi ssp. n.

code	shell colour	spire	anterior lamella	lower plica	shells opened
20071118B	yellow	very slightly elevated	dissolved	reaches lamella	1
2012/62	dark yellow	slightly elevated	normal or dissolved	exctends lamella	2
20080509C	yellowish-corneous	slightly elevated	normal	exctends lamella	1
2007.11.16C= 2011/106	dark yellow	very slightly elevated	dissolved	exctends lamella	2
Vn12-104= Vn10-103, 2012/60	light or dark brown	slightly elevated	normal or dissolved	reaches or exctends lamella	4
20071118A	dark brown	slightly elevated	dissolved	exctends lamella	1
Vn10-76	dark brown	slightly elevated	dissolved or with buttresses	reaches or almost reaches lamella	1
MAA1	yellowish-corneous	slightly elevated	dissolved	reaches lamella	1

##### Description of the genitalia.

Two specimens were anatomically examined. Both specimens had embryos developing in their uterus. Localities: “Specimen1”, Hòa Bình Province, ca. km 156 old road Hà Nội to Sơn La (right side off road), 20°46.000'N, 104°53.885'E, leg. Hemmen, Ch. & J., 15.10.2010. (with 3 embryos, Figures 20, 29F, 31B, 35D–F); “Specimen2”, Nghệ An Province, Pù Huống Nature Reserve, Con Coung District, Anh Son, Hoi Son, ca 30 m, 18°57.11872'N, 105°02.63029'E, leg. Ohara, K, Okubo, K & Otani, J. U., Sang, 10.05.2008. (Figures 28E, 29G).

Penis relatively short and slim, attached to the slightly shorter epiphallus by weak fibres; penis internally with longitudinal folds; the folds are more elevated in the distal part of the penis and they from characteristic “pockets” (Figure 28E); the pockets are arranged in two rows, the upper row (closer the distal end of the penis) is slightly curved on the opened penial wall, but the lower row follows a a wavy line with two peaks; epiphallus have longitudinal folds on the inner wall; penial caecum long; “Specimen1” had two times longer caecum than “Specimen2”; internally with small hollows arranged in longitudinal lines (Figure 29F); “Specimen2” had a few elongated and globular calcareous granules within the hollows (Figure 29G); retractor muscle very long and slim, attaches on the distal end of the penial caecum; vas deferens very long. Vagina extremely long, cylindrical in “Specimen1” and with well-developed vaginal bulb in “Specimen2”; inner wall of the vagina with 6–8 low, parallel or converging folds (Figure 31B); gametolytic sac and diverticulum of the same length, both relatively slim, although the gametolytic sac is a bit swollen.

##### Radula.

See Table 6 and Figures 35D–F.

##### Etymology.

The new subspecies is dedicated to and named after our colleague and much-valued friend, Dinarzarde Raheem.

##### Type locality.

Thanh Hoa Province, Cam Thuy District.

##### Distribution

(see Figure 43). The new subspecies is known from several localities in Ninh Bình, Thanh Hóa, Sơn La, Hòa Bình and Nghệ An provinces.

#### Gudeodiscus (Gudeodiscus) phlyarius

Taxon classificationAnimaliaStylommatophoraPlectopylidae

(Mabille, 1887)

Figures 4A–F , 5A–C , 9C–D , 10C–F , 11K–X , 12A–M , 21 , 22 , 28A , 28C , 31C , 35J–L

Plectopylis phlyaria Mabille 1887a, Molluscorum Tonkinorum diagnoses: 6. [type locality not specified].
Plectopylis phlyaria . Mabille 1887b, Bulletin de le Société Malacologique de France, 4: 100–101., Plate 2, Figs 1–3.
Plectopylis phlyaria , — Pilsbry 1893, Manual of Conchology..., 2(8): 158, Plate 43, Figs 40–42.
Plectopylis phlyaria , — Gude 1897b, Science Gossip, 4: 139., Figs 61a–b. [“Tonkin”].
Plectopylis (Endoplon) phlyaria , — Gude 1899c, Science Gossip, 4: 148.
Plectopylis (Endoplon) phlyaria , — Gude 1899d, Science Gossip, 6: 175.
Plectopylis (Endoplon) phylaria , — Gude 1901c, Journal of Malacology, 8: 113–115., Figs 3a–f. [“Than Moi”].
Plectopylis (Endoplon) moellendorffi 1901c Gude, Journal of Malacology, 8: 115–116., Figs 4a–f. [“Than-Moi”].
Plectopylis gouldingi Gude 1909, **syn. n.**, Proceedings of the Malacological Society of London, 8: 215, 217., Plate 9, Figs 1, 1a–b. [“Nat-Son”].
Plectopylis verecunda Gude 1909, **syn. n.**, Proceedings of the Malacological Society of London, 8: 215, Plate 9, Figs 3, 3a–b. [“Phony-Tho”].
Plectopylis fallax Gude 1909, **syn. n.**, Proceedings of the Malacological Society of London, 8: 217, Plate 9, Figs 6, 6a–b. [“Muong-Bo”].
Plectopylis anterides Gude 1909, **syn. n.**, Proceedings of the Malacological Society of London, 8: 216, Plate 9, Figs 2, 2a–b. [“Pac-Kha”].
Gudeodiscus phlyarius phlyarius (and *Plectopylis moellendorffi* is synonym), — Páll-Gergely & Hunyadi 2013, Archiv für Molluskenkunde, 142 (1): 25–28., Figs 31, 61a–b, 63–65, 75 (map) 77a–b, 112–114.
Gudeodiscus fallax , — Páll-Gergely & Hunyadi 2013, Archiv für Molluskenkunde, 142 (1): 8.
Gudeodiscus gouldingi , — Páll-Gergely & Hunyadi 2013, Archiv für Molluskenkunde, 142 (1): 8.
Gudeodiscus verecundus , — Páll-Gergely & Hunyadi 2013, Archiv für Molluskenkunde, 142 (1): 8.
Gudeodiscus phlyarius werneri Páll-Gergely in Páll-Gergely & Hunyadi 2013, **syn. n.**, Archiv für Molluskenkunde 142 (1): 13: Figs 32, 28–29, 34: Figs 76a–d.

##### Types examined.

Tonkin, Muong-Bo, leg. Messager, NHMUK 1922.8.29.58 (holotype of *fallax*, Figure 5C); Tonkin, Nat-Son, leg. Messager, NHMUK 1922.8.29.56 (holotype of *gouldingi*, Figure 4E); Tonkin, Pac-Kha, NHMUK 1922.8.29.57 (holotype of *anterides*, Figure 4F); Tonkin, MNHN 24581 (2 syntypes of *phlyaria*, Figure 4A); Tonkin, Than-Moi, collection Möllendorff ex Fruhstorfer, SMF 150125a (lectotype of *moellendorffi*, Figure 4B); Tonkin, Than-Moi, collection Möllendorff ex Fruhstorfer, SMF 150125b (paralectotype of *moellendorffi*); Tonkin, Phony-Tho, leg. Messager, NHMUK 1922.8.29.55 (holotype of *verecunda*, Figure 5B).

##### Museum material examined.

***fallax*-like shells.** Tonkin, région de Lao Kay, coll. Dosch ex Rolle, SMF 172081/4; Tonkin, Muong-Bo, coll. Dosch ex Rolle, SMF 172077/2; Tonkin, Muong-Kong, coll. Dosch ex Rolle, SMF 172080/4; Muong-Hum, leg. Messager, MNHN-IM-2012-2130/1; Pakhé, leg. Messager, MNHN-IM-2012-2132/19; Pakhé, leg. Messager, MNHN-IM-2012-2135/6; Muong-Kong, leg. Messager, MNHN-IM-2012-2138/2; Muong-Kong, leg. Messager, MNHN-IM-2012-2140/3; Muong-Hum, leg. Messager, MNHN-IM-2012-2144/1; Ban-Lao, leg. Messager, MNHN-IM-2012-2146/28; Trinh-Thuong, leg. Messager, MNHN-IM-2012-2147/29; Pakhé, leg. Messager, MNHN-IM-2012-2148/3 (“var. *major*”); Pac-Kha (Pakhé), leg. Messager, MNHN-IM-2012-2155/6 (“var. *major*”); Pac-Kha, leg. Messager, MNHN-IM-2012-2208/3 (“var. *major*”); Ban-Lao, leg. Messager, MNHN-IM-2012-2150/22; Pac-Kha (Pakhé), leg. Messager, MNHN-IM-2012-2157/19; Muong-Kong, leg. Messager, MNHN-IM-2012-2158/10; Pac-Kha (Pakhé), leg. Messager, MNHN-IM-2012-2160/22; Trinh-Thuong, leg. Messager, MNHN-IM-2012-2161/28; Pac-Kha (Pakhé), leg. Messager, MNHN-IM-2012-2168/14; Muong-Kong, leg. Messager, MNHN-IM-2012-2169/10; Muong-Hum, leg. Messager, MNHN-IM-2012-2174/1; Muong-Bo, leg. Messager, MNHN-IM-2012-2178/8; Pac-Kha, leg. Messager, MNHN-IM-2012-2180/4; Tonkin, leg. Messager, MNHN-IM-2012-2182/20; Muong-Hum, leg. Messager, MNHN-IM-2012-2190/7; Long-Ping, leg. Messager, MNHN-IM-2012-2192/11; Long-Ping, leg. Messager, MNHN-IM-2012-2206/16; Pac-Kha, leg. Messager, MNHN-IM-2012-2209/4; Pac-Kha, leg. Messager, MNHN-IM-2012-2210/2; Muong-Kong, leg. Messager, MNHN-IM-2012-2244/1; Pakhé, leg. Messager, MNHN-IM-2012-2245/9; Cao-Bang, leg. Messager, MNHN-IM-2012-2470/2; Na-Ri, leg. Messager, MNHN-IM-2012-2463/1; Col de Nuages, leg. Messager, MNHN-IM-2012-2451/6; Tonkin, leg. Messager, MNHN-IM-2012-2450/15; Nat-Son, leg. Messager, MNHN-IM-2012-2445/1; Tonkin, leg. Messager, MNHN-IM-2012-2442/2; Bac-Kan, leg. Messager, MNHN-IM-2012-2247/1; Nga-Son, leg. Messager, MNHN-IM-2012-2255/1; Environs de Yen Bai, ex coll. labo. de Géologie de la Sorbonne (entrée 1952), MNHN-IM-2012-2272/1; Pakhé, leg. Messager, MNHN-IM-2012-2340/12; Tonkin, leg. Messager, MNHN-IM-2012-2395/2; Tonkin, leg. Messager, MNHN-IM-2012-2396/2; Muong-Bo, coll. Staadt, 1969, MNHN-IM-2012-2406/4; Tonkin, coll. Letellier, 1949, MNHN-IM-2012-2410/1; Tonkin, coll. Staadt, 1969, MNHN-IM-2012-2412/1; Trinh-Thuong, coll. Staadt, 1969, MNHN-IM-2012-2416/5; Tonkin, coll. Staadt, 1969, MNHN-IM-2012-2420/1; Trinh-Thuong, coll. Lavezzari, 1929, MNHN-IM-2012-2421/10; Tonkin, Pac-Kha, NHMUK 1916.3.16.14/1; Tonkin, Trinh-Thuong, 5/1/09, NHMUK 20130621.1–2/2; Tonkin, Pac-Kha, 14/6/10, NHMUK 20110289/3 (labelled as „*anterides*“); Tonkin, Pac-Kha, coll. Preston, 3/11/08, NHMUK 20110290/2 (labelled as „*moellendorffi*“); Tonkin, Muong-Bo, coll. Salisbury ex Beddome, NHMUK 20110291/3 (labelled as „*fallax*=*moellendorffi*“); Tonkin, Lao Kay, NHMUK 1920.1.20.15–16/2; Tonkin, Muong-Bo, NHMUK 1909.3.14.18–20/3; Tonkin, Trinh-Thuong, coll. Rosen ex Messager, NHMW 71640/O/9481/1; Tonkin, Haut-Tonkin, Region de Lao-Kay, coll. Rolle, NHMW 92564/2; Tonkin, Muong-Kong, coll. Rušnov ex Rolle ex Messager, NHMW 92565/1; Tonkin, Pac-Kha, NHMW 46226/1; Tonkin, Long-Po (?), coll. Oberwimmer ex Wagner ex Messager, NHMW 92579/1; Tonkin, Muong-Bo, NHMW 46291/2.

***gouldingi*/*anterides*-like shells.** Pakhé, leg. Messager, MNHN-IM-2012-2133/53; Muong-Kong, leg. Messager, MNHN-IM-2012-2141/14; Na-Ri, leg. Messager, MNHN-IM-2012-2152/8; Nat-Son, leg. Messager, MNHN-IM-2012-2153/118; Pac-Kha (Pakhé), leg. Messager, MNHN-IM-2012-2156/4; Pac-Kha (Pakhé), leg. Messager, MNHN-IM-2012-2164/44; Bac-Kan, leg. Messager, MNHN-IM-2012-2167/29; Muong-Kong, leg. Messager, MNHN-IM-2012-2170/1; Tonkin, leg. Messager, MNHN-IM-2012-2175/8; Tonkin, leg. Messager, MNHN-IM-2012-2176/10; Muong-Bo, leg. Messager, MNHN-IM-2012-2179/1; Nac-Ri, leg. Messager, MNHN-IM-2012-2187/6; Muong-Hum, leg. Messager, MNHN-IM-2012-2189/1; Long-Ping, leg. Messager, MNHN-IM-2012-2193/1; Bac-Kan, leg. Messager, MNHN-IM-2012-2195/18; Long-Ping, leg. Messager, MNHN-IM-2012-2197/4; Pac-Kha, leg. Messager, MNHN-IM-2012-2200/32; Pac-Kha, leg. Messager, MNHN-IM-2012-2201/15; Tonkin, leg. Messager, MNHN-IM-2012-2203/1; Long-Ping, leg. Messager, MNHN-IM-2012-2207/4; Long-Ping, leg. Messager, MNHN-IM-2012-2213/2; Cho-Ra, leg. Messager, MNHN-IM-2012-2478/1; Bac-Kan, leg. Messager, MNHN-IM-2012-2476/2; Trinh-Thuong, leg. Messager, MNHN-IM-2012-2473/6; Bac-Kan, leg. Messager, MNHN-IM-2012-2465/4; Na-Ri, leg. Messager, MNHN-IM-2012-2464/1; Na-Ri, leg. Messager, MNHN-IM-2012-2462/8; Tonkin, leg. Messager, MNHN-IM-2012-2459/1; Pakhé, leg. Messager, MNHN-IM-2012-2454/8; Col de Nuages, leg. Messager, MNHN-IM-2012-2452/15; Nat-Son, leg. Messager, MNHN-IM-2012-2446/1; Col de Nuages, leg. Messager, MNHN-IM-2012-2214/9; Na-Ri, leg. Messager, MNHN-IM-2012-2220/8; Pakhé, leg. Messager, MNHN-IM-2012-2226/5; Tonkin, leg. Messager, MNHN-IM-2012-2228/1; Muang-Kong, leg. Messager, MNHN-IM-2012-2243/7; Nat-Son, leg. Messager, MNHN-IM-2012-2256/12; Phi-Mi, leg. Messager, MNHN-IM-2012-2334/1; Tonkin, leg. Messager, MNHN-IM-2012-2372/3; Muong-Kong, leg. Messager, MNHN-IM-2012-2429/8; Bac-Kan, leg. Messager, MNHN-IM-2012-2433/16; Bac-Kan, leg. Messager, MNHN-IM-2012-2436/1; Tonkin, leg. Messager, MNHN-IM-2012-2422/8; Pakhé, leg. Messager, MNHN-IM-2012-2389/2; Bac-Kan, leg. Messager, MNHN-IM-2012-2404/1; Tonkin, coll. Levazzari, 1929, MNHN-IM-2012-2405/3; Muong-Bo, coll. Staadt, 1969, MNHN-IM-2012-2407/1; Trinh-Thuong, coll. Levazzari, 1929, MNHN-IM-2012-2409/1; Bac-Kan, leg. Messager, MNHN-IM-2012-2438/1; Tonkin, leg. Messager, MNHN-IM-2012-2439/6; Tonkin, Pac-Kha, coll. Kennard, A.S. ex auct. (Gude), NHMUK 20130620/1; Tonkin, Pac-Kha, coll. Salisbury ex Beddome, NHMUK 20110285/1 (“gouldingi var. minor”); Tonkin, Pac-Kha, coll. Preston, 3/11/08, NHMUK 20110286/2; Tonkin, Pac-Kha, coll. Salisbury ex Beddome, NHMUK 20110287/2 (“*anterides*”); Tonkin, Pac-Kha, coll. Preston, 3/11/08, NHMUK 20110288/2 (“*anterides*”); Tonkin, Pac-Kha, 1909.3.17.21-23/3 (“*anterides*”); Tonkin, Long-Ping NHMUK 1916.3.16.3/1 (“*anterides*”); Tonkin, Pac-Kha, Tonkin, Pac-Kha, NHMUK 1909.3.17.26-28/3; Tonkin, Pac-Kha, coll. Rosen ex Messager, NHMW 71640/O/9478/2; Tonkin, Bac-Kha, coll. Rušnov ex Rolle ex Messager, NHMW 92566/2; Tonkin, Pac-Kha, NHMW 46225/2; Tonkin, Pac-Kha, coll. Wagner ex Messager, NHMW 71640/O/10290/1; Tonkin, Long-Phai, coll. Wagner ex Messager, NHMW 71640/O/10291/1; Tonkin, Pac-Kha, NHMW 92568/1; Tonkin, Pac-Kha, NHMW 46292/2; Tonkin, Bac-Kan, coll. Wagner ex Messager, NHMW 71640/O/10292/1; Tonkin, Bac-Kan, coll. Oberwimmer, NHMW 71640/O/14029/3; Tonkin, Nat-Son, coll. Rušnov ex Messager, NHMW 103354/1.

“**Mixed” *gouldingi*/*anterides*/*fallax* samples.** Bac-Kan, leg. Messager, MNHN-IM-2012-2171/20; Trinh-Thuong, leg. Messager, MNHN-IM-2012-2181/44; Pakhé, leg. Messager, MNHN-IM-2012-2185/31; Muong-Bo, leg. Messager, MNHN-IM-2012-2211/3; Col de Nuages, leg. Messager, MNHN-IM-2012-2218/25; Col de Nuages, leg. Messager, MNHN-IM-2012-2222/15; Tonkin, leg. Messager, MNHN-IM-2012-2224/13; Tonkin, Pac-Kha, coll. Dosch ex Rolle ex Messager, SMF 172079/4.

***phlyarius*-like shells.** Tonkin, Than-Moi, coll. Jetschin, SMF 207669/6; Tonkin, Than-Moi, coll. Möllendorff ex Fruhstorfer, SMF 150126/10; Tonkin, Chuot-Ki (?), coll. Jaeckel, S. H., SMF 207676/1; Tonkin, coll. Ehrmann ex Fruhstorfer, SMF 150127/2; Tonkin, Than-Moi, coll. Dosch ex Rolle, SMF 172092/4; Tonkin, Than-Moi, coll. Dosch ex Rolle, SMF 172091/4; Tonkin, Than-Moi, coll. Dosch ex Rolle, SMF 172093/2; Tonkin, Than-Moi, coll. Ehrmann ex Fruhstorfer, H., SMF 150138/1+1jb; Than-Moi, leg. Messager, MNHN-IM-2012-2212/5; Long-Phai, leg. Messager, 1901, MNHN-IM-2012-2232/1; Than-Moi, coll. Staadt, 1969, MNHN-IM-2012-2279/4; Tonkin, coll. Weiss, 1901, MNHN-IM-2012-2281/5; Province de Cao Lang, Lang-Son, Ky Lua, coll. Saurin, MNHN-IM-2012-2288/2; Na-Ri, leg. Messager, MNHN-IM-2012-2474/1; Tonkin, leg. Messager, MNHN-IM-2012-2427/3; Tonkin, leg. Messager, MNHN-IM-2012-2431/1; Tonkin, leg. Messager, MNHN-IM-2012-2391/1; Bac-Kan, coll. Staadt, 1969, MNHN-IM-2012-2392/2; Than-Moi, coll. Staadt, 1969, MNHN-IM-2012-2397/5; Than-Moi, coll. Staadt, 1969, MNHN-IM-2012-2398/1; Lang-Son, coll. Letellier, 1949, MNHN-IM-2012-2401/1; Than-Moi, coll. Staadt, 1969, MNHN-IM-2012-2413/8; Tonkin, coll. Denis, 1946, MNHN-IM-2012-2387/4; Tonkin, Pac-Kha, NHMUK 1916.3.16.13/1; Tonkin, coll. Salisbury ex Beddome, NHMUK 20130599/2; Tonkin, Muong-Bo, 3/11/08, NHMUK 20130600/2; Tonkin, 4/11/01/32, NHMUK 20130601/3; Tonkin, Phu Quac Oai, coll. Biggs, H.E.J., Acc. no. 2258, NHMUK 20130602/4; Tonkin, coll. Trechmann, Acc. no. 2176, NHMUK 20130603/2; Tonkin, Than-Moi, leg. Fruhstorfer, H., NHMUK 1901.12.12.206–208/3; Tonkin, „showing immature armature“, coll. Gude, G.K, NHMUK 1916.3.15.3/1; Tonkin, coll. Fruhstorfer, NHMW 40850/2; Tonkin, coll. Rušnov ex Blume, NHMW 92562/2; Tonkin, Than-Moi, NHMW 39292/4; Tonkin, Than-Moi, coll. Klemm, NHMW 79000/K/17483/1; Tonkin, Than-Moi, coll. Rušnov ex Rolle ex Messager, NHMW 92580/2; Tonkin, Than-Moi, coll. Rušnov ex Rolle, NHMW 92581/4; Tonkin, Than-Moi, coll. Rolle, NHMW 71640/O/12301/1; Tonkin, Than-Moi, coll. Edlauer, NHMW 75000/E/38490/3; Tonkin, That-Ké, coll. Oberwimmer, NHMW 71640/O/12300/1; Tonkin, coll. Fruhstorfer, NHMW 40851/1; Tonkin, That-Ke, coll. Oberwimmer, NHMW 92560/2; Tonkin, Bac-Khuon, coll. Rolle, NHMW 50857/1 (mixed sample with *giardi*).

***verecunda*-like shells.** Phong-Tho, leg. Messager, MNHN-IM-2012-2177/9; Nat-Son, leg. Messager, MNHN-IM-2012-2447/6; Phong-Tho, leg. Messager, MNHN-IM-2012-2443/4; Phong-Tho, leg. Messager, MNHN-IM-2012-2423/4; Lai-Chau, coll. Morlet, MNHN-IM-2012-2424/1; Son-Ma, coll. Fischer, MNHN-IM-2012-2417/1.

##### New material examined.

***fallax*-like shells. 2011/125** Lào Cai Province, 1.5 km N of Bắc Ngầm cross, valley on the left side of the road, 155 m, 22°24.149'N, 104°14.462'E, leg. Hunyadi, A., 02.12.2011., HA/1; **Vn11-187** Lào Cai Province, ca. 3 km SW of Nhà Văn Hóa, 22°25.513'N, 104°12.194'E, leg. Hemmen, Ch. & J., 04.10.2011., HE/21 (+2 specimens in ethanol, one of them anatomically examined, Figures 21, 28A).

***phlyarius*-like shells. Vn10-53** Lạng Sơn Province, right off rd. 1B Long Đống to Bình Gia, 21°53.938'N, 106°25.605'E, leg. Hemmen, Ch. & J., 20.3.2010., PGB/3; **Vn10-48** Lạng Sơn Province, ca. 6 km SE Bắc Sơn (rd. Bắc Sơn to Nga Hải, left off rd), 21°52.422'N, 106°21.508'E, leg. Hemmen, Ch. & J., 19.03.2010., PGB/3; **Vn09-24** Cao Bằng Province, ca. 1 km N of Mã Phục (right side off rd. 3), ca. 575 m, 22°43.938'N, 106°20.527'E, leg. Hemmen, Ch. & J., 23.03.2009., HE/1, PGB/3; **Vn10-49** Lạng Sơn Province, ca. 16 km SE Bắc Sơn (rd. Bắc Sơn to Nga Hải, left off rd), 21°50.019'N, 106°18.405'E, leg. Hemmen, Ch. & J., 19.03.2010., PGB/2+2jb; **Vn09-18** Lạng Sơn Province, ca. 27 km S of Thất Khê, right side off rd. #4 (Lạng Sơn-Thất Khê), ca. 300 m, 22°07.484'N, 106°35.427'E, leg. Hemmen, Ch. & J., 13.10.2009., PGB/7; **Vn09-19** Lạng Sơn Province, ca. 25 km S of Thất Khê, right side off rd. #4 (Lạng Sơn-Thất Khê), ca. 220 m, 22°06.477'N, 106°35.356'E, leg. Hemmen, Ch. & J., 13.10.2009., PGB/2; **Vn10-129** Lạng Sơn Province, ca. 58.5 km from Thái Nguyên to Bắc Sơn (right side off road), 21°51.166'N, 106°13.003'E, leg. Hemmen, Ch. & J., 22.10.2010., PGB/1; **Vn10-56** Lạng Sơn Province, ca. 7 km from Đồng Mỏ to Văn Quan (left off rd #279), no GPS data, approximate GPS position: 21.696000°N, 106.547271°E, leg. Hemmen, Ch. & J., 21.3.2010., PGB/5; **Vn09-16** Lạng Sơn Province, Tân Mỹ (N of Lạng Sơn), temple south of the entrance of village, ca. 240 m, 21°58.891'N, 106°40.265'E, leg. Hemmen, Ch. & J., 12.10.2009., PGB/3; **Vn10-128** Lạng Sơn Province, ca. 69 km from Thái Nguyên to Bắc Sơn (right side off road), 21°54.270'N, 106°15.801'E, leg. Hemmen, Ch. & J., 22.10.2010., HE/8, PGB/9; **Vn11-154** Lạng Sơn Province, km 47, 1 road # 1B between Văn Quan and Bắc Sơn, 21°52.785'N, 106°26.262'E, leg. Hemmen, Ch. & J., 01.04.2011., HE/6 (also in ethanol); **Vn11-155** Lạng Sơn Province, ca. 55 km from Bình Gia to Lạng Sơn on road 1B (no GPS data), leg. Hemmen, Ch. & J., 01.04.2011., HE/11; **Vn11-156** Lạng Sơn Province, ca. 10.6 km from Bình Gia to Lạng Sơn on road 1B, 21°53.639'N, 106°25.895'E, leg. Hemmen, Ch. & J., 01.04.2011., HE/70 (one of them is sinistral!), (anatomically examined, Figures 22, 28C, 35J–L); **Vn11-157** Lạng Sơn Province, ca. km. 50 of road 1B, 10 km to Bình Gia, 21°53.911'N, 106°25.664'E, leg. Hemmen, Ch. & J., 01.04.2011., HE/6 (anatomically examined, see Figure 31C); **2011/65** Lạng Sơn Province, Đồng Mỏ 2.5 km towards Văn Quan, right side of the road, 270 m, 21°40.358'N, 106°34.783'E, leg. Hunyadi, A., 10.11.2011., HA/5; **2011/66** Lạng Sơn Province, Đồng Mỏ 4.5 km towards Văn Quan, left side of the road, 330 m, 21°40.828'N, 106°34.531'E, leg. Hunyadi, A., 10.11.2011., HA/23, PGB/2; **2011/67** Lạng Sơn Province, Đồng Mỏ 6 km towards Văn Quan, left side of the road, 390 m, 21°41.034'N, 106°33.618'E, leg. Hunyadi, A., 10.11.2011., HA/20, PGB/2; **2011/68** Lạng Sơn Province, Đồng Mỏ 7 km towards Văn Quan, Vạn Linh cross., left side of the road, 370 m, 21°41.158'N, 106°33.588'E, leg. Hunyadi, A., 10.11.2011., HA/56, PGB/3; **2011/70** Lạng Sơn Province, Lạng Sơn, NNE side of Núi Vọng Phu, 21°51.183'N, 106°44.950'E, leg. Hunyadi, A., 11.11.2011., HA/3; **2011/72** Lạng Sơn Province, Na Sầm 12 km towards Thất Khê, left side of the road 210 m, 22°07.870'N, 106°35.038'E, leg. Hunyadi, A., 12.11.2011., HA/86, PGB/2; **2011/73** Lạng Sơn Province, Na Sầm 10 km towards Thất Khê, left side of the road, 190 m, 22°07.530'N, 106°35.381'E, leg. Hunyadi, A., 12.11.2011., HA/27, PGB/2; **2011/74** Lạng Sơn Province, Na Sầm 5.5 km towards Thất Khê, right side of the road, 165 m, 22°05.466'N, 106°35.425'E, leg. Hunyadi, A., 12.11.2011., HA/10; **2011/75** Lạng Sơn Province, Tân Mỹ, tunnel 200 m towards Na Sầm, 210 m, 21°59.110'N, 106°40.077'E, leg. Hunyadi, A., 12.11.2011., HA/19, PGB/2; **2011/76** Lạng Sơn Province, northern edge of Chi Lăng, pass next to the tourist path (N of Đồng Bành), 75 m, 21°34.945'N, 106°30.567'E, leg. Hunyadi, A., 13.11.2011., HA/1; **2011/78** Lạng Sơn Province, Đồng Mỏ 7 km towards Chi Lăng, right side of the road, leg. Hunyadi, A., 13.11.2011., HA/1; **2011/79** Lạng Sơn Province, Đồng Mỏ 5.2 km towards Chi Lăng, right side of the road, 40 m, 21°37.215'N, 106°32.538'E, leg. Hunyadi, A., 13.11.2011., HA/1; **2012/37** Lạng Sơn Province, Đồng Mỏ 2.7 km towards Chi Lăng, right side of the old road, cave, 70 m, 21°38.286'N, 106°33.391'E, leg. Hunyadi, A., 25.05.2012., HA/10; **2012/38** Lạng Sơn Province, Đồng Mỏ 4–5 km towards Chi Lăng, right side of the old road, 65 m, 21°37.479'N, 106°32.730'E, leg. Hunyadi, A., 25.05.2012., HA/6; **Vn11-159** Lạng Sơn Province, at km 74.8 on road 1B, Đồng Đăng to Thái Nguyên (8 km S Bắc Sơn), 21°54.543'N, 106°17.298'E, leg. Hemmen, Ch. & J., 02.04.2011., HE/1; **Vn11-158** Lạng Sơn Province, ca. 7.5 km foad 1B from Bình Gia to Bắc Sơn, 21°53.908'N, 106°25.661'E, leg. Hemmen, Ch. & J., 01.04.2011., HE/1; **Vn09-06** Ninh Bình Province, Cúc Phương Nat. Park, ca. half way from Park Headquarters to Thousand Year Old Tree, left path, ca 510 m, 20°21.366'N, 105°35.513'E, leg. Hemmen, Ch. & J., 03.10.2009., HE/2; **MAA10** Ninh Bình Province, Cúc Phương Nat. Park, path to fairy cave, 20°21'N, 105°54'E (approximate GPS position), leg. Vermeulen, J., coll. Maassen, W.J.M., 10.10.1998., NHMUK 19991444/2 + one juvenile/broken shell (marked with no. 3 on Figure 43); same data, WM/3; **Vn10-41** Thái Nguyên Province, Temple Chùa Hang (ca. 1 km S of Chợ Chu), 21°54.070'N, 105°38.856'E, leg. Hemmen, Ch. & J., 16.03.2010., HE/3 (marked with no.2 on Figure 43).

##### Diagnosis.

The species is very variable in terms of shell characters (spire height, presence/absence of the apertural fold, aperture shape, morphology of the parietal and palatal plicae and lamellae, fine morphology of the periostracum folds) between and within traditionally recognized species which are synonymized here. Therefore, it is impossible to give a general diagnosis.

##### Measurements

(in mm). D = 19.3–20.2, H = 8.8–9.1 (n=3, “*fallax*”, MNHN 2012-2155); D = 10.6–11.7, H = 4.5–4.7 (n=4, “*gouldingi*”, MNHN, IM-2012-2164); D = 13.2–13.4, H = 5.9–6 (n=2, “*phlyarius*”, Vn10-53); D = 14.7–15.5, H = 7.8–8.5 (n=3, “*phlyarius*”, Vn09-18); D = 12.4–12.7, H = 5.7–5.8 (n=2, “*phlyarius*”, MAA10); D = 15.5–17.1, H = 7.7–7.8 (n=2, “*phlyarius*”, Vn10-56); D = 15.8–16.6, H = 8.8–9 (n=3, *verecunda*, MNHN 2012-2177). The size range is continuous to from typical *anterides*/*gouldingi* to fallax var. major (see Figure 16).

##### Differential diagnosis.

See under *Gudeodiscus anceyi*, *Gudeodiscus emigrans*, *Gudeodiscus giardi*, *Gudeodiscus hemmeni* sp. n., *Gudeodiscus messageri* and *Halongella fruhstorferi*.

**Intrasubspecific diversity.** Extremely large. Table 9 summarized the conchological differences between newly collected Vietnamese *Gudeodiscus phlyarius* samples.

**Table 9.** Diversity of shell characters within newly collected Vietnamese Gudeodiscus (Gudeodiscus) phlyarius. Abbreviations: OCMA: only corroded material available.

code	spire	aperture shape	periostracal folds
Vn11-187	flat	elongated	normal
2011/66	slightly elevated	rounded	pointed
2011/67	flat/slightly elevated	rounded	pointed
2011/68	slightly elevated	rounded	pointed
2011/70	slightly elevated	rounded	OCMA
2011/72	slightly elevated	rounded	normal
2011/73	slightly elevated	rounded	OCMA
2011/75	flat/slightly elevated	rounded	normal
Vn09-16	slightly elevated	rounded	OCMA
Vn09-18	slightly elevated	rounded	normal
Vn09-19	slightly elevated/ elevated	rounded	OCMA
Vn09-24	flat/slightly elevated	rounded	OCMA
Vn10-128	flat/slightly elevated	rounded	normal
Vn10-129	slightly elevated	rounded	normal
Vn10-48	flat/slightly elevated	rounded	OCMA
Vn10-49	flat/slightly elevated	rounded	pointed
Vn10-53	flat	rounded	pointed
Vn10-56	flat/slightly elevated	rounded	pointed

##### Description of the genitalia.

**Typical *fallax*:** Two specimens were anatomically examined. Locality: Lào Cai Province, ca. 3 km SW of Nhà Văn Hóa, 22°25.513'N, 104°12.194'E, leg. Hemmen, Ch. & J., 04.10.2011. (Figures 21, 28A);

Penis rather spindle-shaped, very much thickened in the middle; internally with a fine papillated/reticulated structure (proximal part) which gradually becomes a laterally folded structure with flat calcareous granules between the folds; pockets are arranged in a rather straight line; epiphallus much shorter than penis, thickest at the penis-epiphallus transition, slowly becoming slimmer towards the vas deferens; penis and epiphallus connected with weak muscle fibres; penial caecum absent in one of the specimens and very small in the other; retractor muscle thick, short, inserts on the small penial caecum (or on the penis-epiphallus transition of the other specimen); vas deferens very long; the proximal section curves within a translucent, straight tube, most convolutions occurring proximally to the vaginal bulb, before becoming a solid, thick tube (until the sperm-oviduct). Vagina long, centrally with well-developed vaginal bulb; vaginal bulb thick-walled, internally with fine reticulated sculpture; distal part of the vagina internally with low, dense, transversal folds; gametolytic sac and diverticulum long, of equal length, extending in parallel; gametolytic sac spindle-shaped, diverticulum of equal thickness throughout.

**typical *phlyarius*:** Two specimens were anatomically examined, both contained a few embryos at an early developmental state. Localities: Lạng Sơn Province, ca. 10.6 km from Bình Gia to Lạng Sơn on road 1B, 21°53.639'N, 106°25.895'E, leg. Hemmen, Ch. & J., 01.04.2011. (Figures 22, 28C); Lạng Sơn Province, ca. km. 50 of road 1B, 10 km to Bình Gia, 21°53.911'N, 106°25.664'E, leg. Hemmen, Ch. & J., 01.04.2011. (Figure 31C).

Penis spindle-shaped with thickened middle section; internally with elongated folds of various thickness; this internal ribbed surface also continues in the small penial caecum; retractor muscle short, inserts on the penial caecum; epiphallus shorter and much slimmer than the penis; distally the penis and proximal part of epiphallus bound with connective tissue; vas deferens very long, proximally simple, slim, curved centrally and covered with a sheath distally simple and thickened. Vagina long with well-developed central vaginal bulb; internally the proximal part of the bulb is almost smooth; this sculpture changes to parallelly folded structure in distal direction (Figure 31C); the distal part of the vagina is strongly folded; gametolytic sac and diverticulum of equal length, both being relatively short.

##### Radula.

See Table 6 and Figures 35J–L.

##### Distribution

(see Figure 43). The populations assigned to *Gudeodiscus phlyarius* inhabit several regions of northern Vietnam (Lạng Sơn, Cao Bằng, Ninh Bình, and along the border region with the Chinese Yunnan Province) and the Chinese Guangxi. A single shell of typical *Plectopylis fallax* Gude, 1909 was collected in southern Yunnan, very close to the Vietnamese border (Honghe Hanizu Yizu Zizhizhou, Hekou Yaozu Zizhixian, Laofanzhai Xiang, Sierqi N 1.5 km towards Laofanzhai, 155 m, 22°44.637'N, 103°53.782'E, leg. Hunyadi, A., 19.03.2011., HA/1).

##### Remarks.

*Gudeodiscus phlyarius* and taxa of similar appearance are one of the most problematical groups in the Plectopylidae. Gude (1909) described six species (*anterides*, *cyrtochila*, *fallax*, *gouldingi*, *messageri*, *verecunda*) from the border region of northern Vietnam with the Chinese Yunnan Province. One species, *Plectopylis cyrtochila* differs from the rest of the species by the smooth, lenticular shell and week peristome and callus. Therefore, it is discussed separately, under the name *Gudeodiscus cyrtochilus*. In face of the obvious similarities between the remaining five species, *Plectopylis messageri* and *Plectopylis fallax* were only compared with *Plectopylis moellendorffi*, and *Plectopylis verecunda* was compared with *Plectopylis messageri*. The shell characters of *Plectopylis anterides* and *Plectopylis gouldingi* were only compared with each other. Shells having transitional characters were explained by hybrid origin. Gude (1909) mentions that a specimen of *messageri* from Pac-Kha might be a hybrid with *moellendorffi*, and another specimen from the same locality was believed to be a hybrid of *anterides* and *gouldingi*. The shell characters distinguishing *Plectopylis messageri* and the sympatric species referable to *fallax*, *gouldingi* and *anterides* are stable, therefore *Gudeodiscus messageri* is handled separately from the rest of the taxa.

In the recent revision of the Chinese members of the family (Páll-Gergely and Hunyadi 2013), *Gudeodiscus phlyarius* was reported from several localities in Guangxi. *Plectopylis moellendorffi* Gude, 1901 was synonymized with *Plectopylis phlyarius*. *Gudeodiscus phlyarius werneri* was described from two nearby localities near Duan city. All other Chinese *Gudeodiscus phlyarius* populations were assigned to the nominotypical subspecies. *Gudeodiscus phlyarius phlyarius* populations were listed in two separate groups based on their appearance, namely “*phlyarius*-like, mainly flat, small form” and “larger, strongly-built shell (transition to *werneri*)”.

Here we include the following taxa as synonyms of *Gudeodiscus phlyarius*: *anterides* Gude, 1909, *fallax* Gude, 1909, fallax var. major Gude, 1909, *gouldingi* Gude, 1909, *moellendorffi* Gude, 1901, *verecundus* Gude, 1909, *werneri* Páll-Gergely, 2013. The last taxon was described on the basis of a keel with a light band around the umbilicus, the dissolved anterior lamella, the posteriorly elongated upper and lower ends of the posterior lamella and the parallel, horizontal palatal plicae. All other formerly recognized species (*anterides*, *fallax*, *gouldingi*, *moellendorffi*, *verecundus*) have two well-developed lamellae and oblique, usually depressed Z-shaped palatal plica, often with Y-like posterior ends. However, this study revealed that *Gudeodiscus phlyarius* is a widely distributed, very variable species and at this moment we see no good reason to maintain one of the morphologically distinct forms as a subspecies. Consequently, we synonymize *Gudeodiscus phlyarius werneri* with *Gudeodiscus phlyarius*.

According to the original description the anterior lamella of *gouldingi* is simple whereas that of *anterides* is “provided with buttresses”. The upper parietal plica is in contact with the anterior lamella in *gouldingi*, but the lamella is shorter and free in *anterides*. Both the upper and lower plicae are shorter in *anterides*. The first palatal plica of *anterides* has a descending ridge; the same plica is straight in *gouldingi*. Additionally, the palatal plicae of *anterides* are not united by a vertical ridge and are more widely spaced than in *gouldingi* (the drawings in the original description show the reverse). All of the differences mentioned by Gude (1909) are unstable even within a single sample (assumed to be single population). For example, six shells were opened from a sample collected in Nat-Son (leg. Messager, MNHN-IM-2012-2153, containing 118 “*gouldingi*” shells). The length of the lower horizontal plica varies greatly, but extends beyond the anterior lamella in the anterior direction in every cases. One specimen had buttresses on the anterior lamella. Two specimens possessed an anterior lamella and the upper horizontal plica united, whereas in the case of four specimens this plica was free. Even among the few shells examined by Gude, he found that shells exhibited transitional character states between *anterides* and *gouldingi*. Therefore, these forms cannot be handled as separate species.

In the original description of *Plectopylis fallax*, Gude (1909) compared it only with *Plectopylis moellendorffi*. He did not compare *Plectopylis fallax* either with *Plectopylis anterides*, or with *Plectopylis gouldingi*. Based on the material housed in the NHM and the specimens mentioned in Gude’s (1909) paper, Gude received very few shells from Messager. Examining the type specimens of the above-mentioned taxa revealed that besides the difference in size (typical *fallax* is larger than *anterides* and *gouldingi*), the only distinguishing feature is the simple and free palatal plicae in *fallax* and the bifurcated and usually connecting plicae of *gouldingi* (syn: *anterides*). The palatal plicae are very variable even within the same sample (see Figures 11) and certainly cannot be used to separate these taxa. Larger shells usually have separated palatal plicae and smaller shells tend to have joint palatal plicae. In addition, the characteristic “nautiliform” shape of typical *fallax* shells is also not a reliable distinguishing feature from *Plectopylis gouldingi*/*anterides* as this trait is also variable across *gouldingi* and *fallax* samples.

Based on shell size, most of Messager’s samples in the MNHN can be assigned to three forms (approximately 11–13 mm: *gouldingi*, 14–16 mm: *fallax*, 19–21 mm: fallax var. major). However, the ranges of shell size overlaps within a few samples (see “mixed” samples under the material) and assigning some of these shells to one of the forms is impossible. The size range from typical *gouldingi* (11 mm) to fallax var. major (21 mm) shows a clinal variation without interruption (see Figure 16). On the other hand, we found one sample where the shells clearly differ from two separate forms, namely six typical “fallax var. major” (D: 18.9–20 mm) and *gouldingi* (D: 12.4–13.5) shells. Unfortunately, as in other samples, the collection locality is not exact enough to determine if these specimens were sympatric.

The apertural fold is always present on typical *Gudeodiscus phlyarius* shells, but can be rudimentary or missing in typical *anterides*/*fallax*/*gouldingi* shells. The edge of the periostracal folds has a pointed structure which seems to occur in a spiralling pattern on the shell of most Vietnamese *phlyarius* specimens, but these are always missing in *fallax* and *gouldingi* specimens (this trait is visible only in fresh shells) (Figures 10C–F). Typical *moellendorffi* specimens (synonym of *phlyarius*) possess a somewhat elevated spire, whereas typical *anterides*/*fallax*/*gouldingi* shells are almost always entirely flat. The only shell character found to be stable within typical Vietnamese *Plectopylis phlyarius* shells and *Plectopylis anterides*/*fallax*/*gouldingi* shells, however, is the rounded aperture in the former and the elongated aperture in the latter (Figures 9C–D). Even this difference is found to be variable in Chinese populations. The populations listed as “transitions to *werneri*” in Páll-Gergely and Hunyadi (2013) have rather elongated aperture, similar to that of typical Vietnamese *fallax* shells, but have elevated spire and overall similar shell shape to typical Vietnamese *phlyarius*. Therefore, we refer to *anterides*, *gouldingi* and *fallax* as synonyms of *Gudeodiscus phlyarius*.

The genital structure of typical *fallax* and typical *phlyarius* differ considerably. Namely, the former lacks the penial caecum or has only a very small one, and has a reticulated inner surface of the penis, whereas the latter has a short penial caecum and its penis has parallel folds on the inner wall. The size of the penial caecum however, may not have a strong taxonomic value because it was found to vary largely within species (e.g. *Gudeodiscus multispira*, see Páll-Gergely and Asami 2014). The sculpture of the wall of the proximal portion of the penis may have a seasonal variability (see under *Gudeodiscus villedaryi* and in Discussion).

A sample (MNHN 2012-2177) labelled *verecunda*, which contained 9 shells from the type locality (Phony-Tho) supports the synonymy of the taxon in relation to *gouldingi* and *fallax*, and therefore to *Gudeodiscus phlyarius*. Seven of the shells were typical *verecundus* with an elevated spire, a strong apertural fold connected to the callus, and an anterior lamella fused to the lower plica; the plica does not extending beyond the lamella anteriorly (confirmed in 3 shells). The two other shells however, have somewhat lower spires, the apertural fold is not connected to the callus and the lower plica is free from the anterior lamella and extended beyond it anteriorly (one of the two shells was opened). These two shells can be interpreted as transitional forms between *verecundus* and *fallax* in terms of spire height, apertural fold and parietal plicae/lamellae morphology. Since transitional forms were found between typical *verecunda* and *fallax* shells, *Plectopylis verecunda* can be interpreted as a local form of *fallax* having elevated spire and fused anterior lamella and lower plica. Therefore, we synonymise *Plectopylis verecunda* with *Gudeodiscus phlyarius*.

There are two Vietnamese “forms” of *Gudeodiscus phlyarius* which differ from all other typical Vietnamese *phlyarius* shells. One of the morphologically distinct forms inhabits Ninh Bình Province, where we have knowledge of two populations (number 3 on Figure 43). These shells are smaller and comparatively flatter than the usual *phlyarius*, and have a characteristic “nautiliform” shape, wider umbilicus, with the last whorl leaving the larger part of the penultimate whorl visible. No differences in the lamellae were recognized. The other form is known from one locality in north-western Thái Nguyên Province (number 2 on Figure 43). This has an elevated spire and narrow umbilicus. Only three specimens are known, and two of them were opened. One of the opened specimens had three very weak parietal lamellae (possibly an abnormal character state, similar to that of the holotype of *Plectopylis infralevis*), and the second has the anterior lamella and the lower plica fused; the plica did not extends beyond the anterior lamella in the anterior direction.

Two Chinese populations (near Baxianyan, number 1 on Figure 43) have an oblique anterior lamella and an aperture more reflected downwards.

#### Gudeodiscus (Gudeodiscus?) suprafilaris

Taxon classificationAnimaliaStylommatophoraPlectopylidae

(Gude, 1908)

Figures 9A–B , 9R , 14S–Y

**Figure 7.**
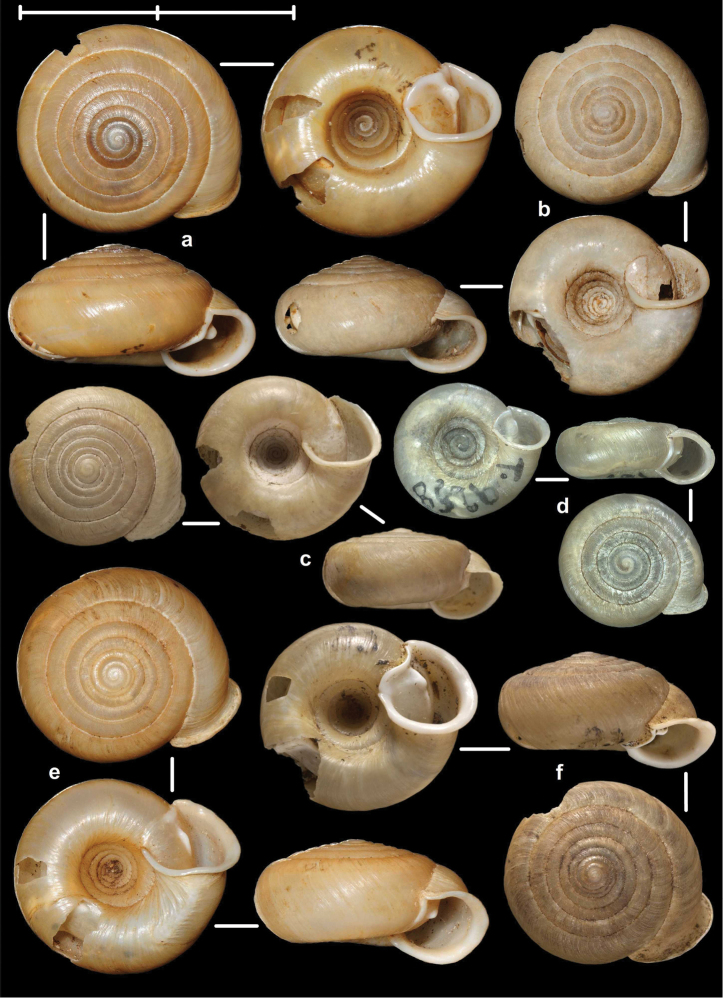
Shells of Vietnamese *Gudeodiscus* and *Halongella* gen. n. species. **A** Gudeodiscus (Gudeodiscus?) francoisi (Fischer, 1898), MNHN 24601 (holotype of *Plectopylis bavayi*) **B** Gudeodiscus (Gudeodiscus?) francoisi, MNHN 9945 (holotype of *Plectopylis francoisi*) **C** Gudeodiscus (Gudeodiscus?) francoisi, NHMUK 1922.8.29.51 (holotype of *Plectopylis lepida*) **D** *Halongella fruhstorferi* (Möllendorff, 1901), SMF 9258 (lectotype) **E** Gudeodiscus (Gudeodiscus) giardi giardi (Fischer, 1898), MNHN IM-2010-12120 (syntype of *Plectopylis congesta*) **F** Gudeodiscus (Gudeodiscus) giardi giardi, NHMUK 1922.8.29.49 (syntype of *Plectopylis congesta*). Photos: T. Deli (**A, B, E**), H. Taylor (**C, F**) and E. Neubert (**D**). Scale represents 20 mm.

**Figure 8.**
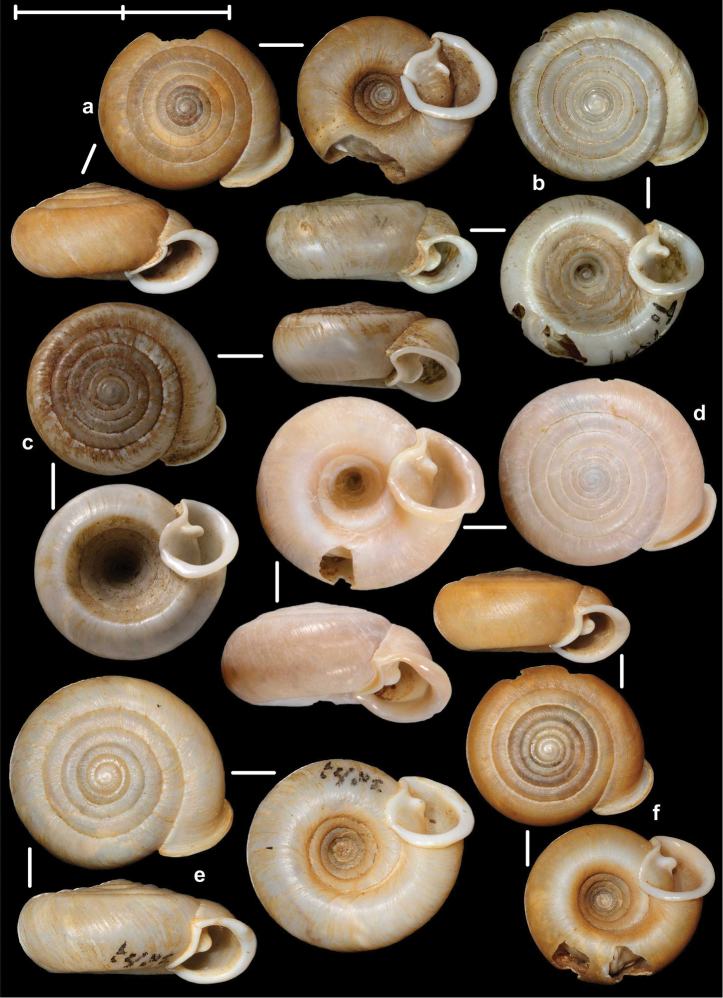
Shells of Vietnamese *Gudeodiscus* and *Halongella* gen. n. species. **A** Gudeodiscus (Gudeodiscus) giardi giardi (Fischer, 1898), MNHN 9946 (syntype of *Plectopylis giardi*) **B** Gudeodiscus (Gudeodiscus) villedaryi (Ancey, 1888), SMF 9279 (lectotype of *Plectopylis choanomphala*) **C** Gudeodiscus (Gudeodiscus) villedaryi, NHMUK 1930.9.12.38 (holotype of *Plectopylis villedaryi*) **D** Gudeodiscus (Gudeodiscus) villedaryi, Vn11-152, coll PGB **E** Gudeodiscus (Gudeodiscus) dautzenbergi (Gude, 1901), MNHN 24603 (holotype) **F** Gudeodiscus (Gudeodiscus) dautzenbergi, MNHN 24602 (holotype of *Plectopylis persimilis*). Photos: T. Deli (**A, E, F**), S. Hof (**B**), H. Taylor (**C**) and B. Páll-Gergely (**D**). Scale represents 20 mm.

**Figure 9.**
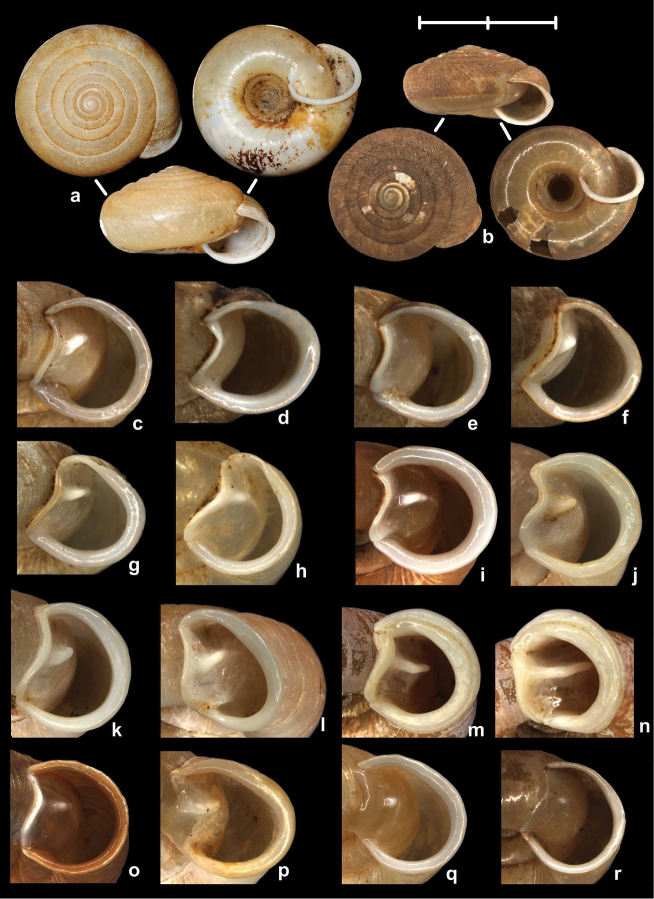
Shells (**A–B**) and apertural views (**C–R**) of Vietnamese *Gudeodiscus*, *Sicradiscus* and *Halongella* gen. n. species. **A** Gudeodiscus (Gudeodiscus?) suprafilaris (Gude, 1908), MNHN 24586 (holotype?) **B** Gudeodiscus (Gudeodiscus?) suprafilaris, 2011/81 **C** Gudeodiscus (Gudeodiscus) phlyarius (Mabille, 1887), Vn11-156 **D** Gudeodiscus (Gudeodiscus) phlyarius (Mabille, 1887) (typical “*anterides*/*gouldingi*”), MNHN-IM-2012-2164 **E** Gudeodiscus (Gudeodiscus) messageri messageri (Gude, 1909), MNHN-IM-2012-2215 **F** Gudeodiscus (Gudeodiscus?) hemmeni Páll-Gergely & Hunyadi, sp. n., Vn10-103A **G** Gudeodiscus (Gudeodiscus?) anceyi (Gude, 1901), GS22 **H** *Sicradiscus mansuyi* (Gude, 1908), 20081116C **I** Gudeodiscus (Gudeodiscus) giardi giardi (Fischer, 1898), 2011/81 **J** Gudeodiscus (Gudeodiscus) villedaryi (Ancey, 1888), Vn11-151 **K–L** Gudeodiscus (Gudeodiscus) dautzenbergi (Gude, 1901), Vn10-44 **M–N** *Halongella schlumbergeri* (Morlet, 1886), MAA3 **O** *Halongella fruhstorferi* (Möllendorff, 1901), Vn11-171 **P** Gudeodiscus (Gudeodiscus) fischeri (Gude, 1901), Vn10-120 **Q** Gudeodiscus (Gudeodiscus) fischeri (Gude, 1901), 20090515C **R** Gudeodiscus (Gudeodiscus?) suprafilaris (Gude, 1908), 2011/81. All photos by B. Páll-Gergely except for Figure 9A (T. Deli). Scale represents 10 mm and refers to **A** and **B.**

Plectopylis suprafilaris , — Gude 1908, Journal de Conchyliologie, 55: 353–355., Figs 4a–e, Plate 7, Figs 7–9. [“Quang Huyen”].
Gudeodiscus suprafilaris , — Páll-Gergely & Hunyadi 2013, Archiv für Molluskenkunde, 142 (1): 8.

##### Types examined.

Tonkin, Quang-Huyen, leg. Mansuy, MNHN 24586 (holotype?, Figure 9A).

##### Museum material examined.

Nga-Son, leg. Messager, MNHN-IM-2012-2234/2; Nga-Son, leg. Messager, MNHN-IM-2012-2254/3.

##### New material examined.

**Vn10-125** Cao Bằng Province, ca 60 km from Cao Bằng to Bảo Lạc (right side off road), 22°39.494'N, 105°51.059'E, leg. Hemmen, Ch. & J., 19.10.2010., PGB/1; **2011/70** Lạng Sơn Province, Lạng Sơn, NNE edge of Vọng Phu Mountain, 21°51.183'N, 106°44.950'E, leg. Hunyadi, A., 11.11.2011., HA/1jb; **2011/81** Cao Bằng Province, Đèo Mã Phục (pass) 500 m towards Quảng Uyên, left side of the road, rock cavern, 610 m, 22°43.981'N, 106°20.333'E, leg. Hunyadi, A., 14.11.2011., HA/73+10jb, PGB/3 (see Figure 9B); **2011/85** Cao Bằng Province, Cao Bằng 34.5 km towards Đông Khê, left side of the road, 500 m, 22°27.487'N, 106°25.047'E, leg. Hunyadi, A., 15.11.2011., HA/4jb; **2012/44** Cao Bằng Province, southern edge of Pắc Rảo, Trùng Khánh 3 km towards Quảng Uyên, left side of the road, 570 m, 22°48.961'N, 106°30.533'E, leg. Hunyadi, A., 28.05.2012., HA/1; **Vn10-67** Cao Bằng Province, right off old rd. 4A, ca 29 km from Cao Bằng to Đông Khê, 22°28.737'N, 106°21.767'E, leg. Hemmen, Ch. & J., 26.03.2010., HE/2.

##### Diagnosis.

Shell small, discoid-globular, with weak apertural lip and usually a small denticle in the aperture (Figure 9R). The sudden change of the shell sculpture (reticulated above, smooth below) is very characteristic of this species. For the morphology of the plicae see Remarks and Figures 14S–Y.

##### Measurements

(in mm). D = 13.1, D = 7.3 (n=1, Vn10-125); D = 11.1–12.1, H = 6.2–6.3 (n=3, 2011/81); D = 12–14.1, H = 6.2–7.2 (n=2, Vn10-67).

##### Differential diagnosis.

The shell shape of *Gudeodiscus suprafilaris* is similar to that of *Gudeodiscus infralevis*, but *Gudeodiscus suprafilaris* has more regular whorls, a more elevated spire and its sculpture changes suddenly from reticulated dorsally to smooth basally on the last whorl. The sudden change of the sculpture and the almost globular shell distinguishes the species from other species (*Gudeodiscus eroessi*, *Gudeodiscus multispira*, *Gudeodiscus soosi*, *Gudeodiscus yunnanensis*, *Gudeodiscus cyrtochilus* and *Gudeodiscus fischeri*). The Chinese *Gudeodiscus eroessi hemisculptus* Páll-Gergely & Hunyadi, 2013 and *Gudeodiscus yanghaoi* which have similar sculpture are larger, have a flatter shell and different lamellation.

##### Intraspecific diversity.

The species is very variable in terms of spire height, the formation of parietal and palatal plicae and lamellae, and the extent of the sculptured portion on the dorsal side of the shell. The distinctive aperture shape, minute apertural fold and the unique sculpture render this species distinctive and easy to identify. See also Remarks and Table 10.

**Table 10.** Diversity of shell characters within Gudeodiscus (Gudeodiscus?) suprafilaris. Abbreviations: OCMA: only corroded material available.

code	spire	anterior lamella	posterior lamella	palatal plicae	changing line of the sculpture
type series	high	short	present	long, united	middle line of the body whorl
2011/81	moderately high	long	present	long, united	lower than the middle line of the body whorl
2012/44	moderately high	unknown	unknown	short, free	middle line of the body whorl
Vn10-125	high	long	absent	only vertical line visible	middle line of the body whorl
Vn10-67	moderately high	unknown	unknown	short, united	lower than the middle line of the body whorl
2011/85	high	short	present	short, free	lower than the middle line of the body whorl
2011/70	high	short	present	short, free	OCMA

##### Distribution

(see Figure 41). Examined material was from only Cao Bằng and Lạng Sơn Provinces. The type locality (Quang-Huyen) lies in Cao Bằng Province (see Figure 39).

##### Remarks.

The palatal and parietal plicae and lamellae exhibit extreme variability between populations. The holotype exhibits relatively long, horizontal palatal plicae connected with a ridge; the parietal side possesses a well-developed posterior lamella, upper and lower plica, and a reduced, short anterior lamella (Figures 14S–T). The museum specimens we examined (probably from the same sample as the holotype) had similar palatal plicae and also a reduced anterior lamella. Two examples collected close to the type locality (2011/81, see Figures 14U–V and 2012/44) were examined. Shells belonging to both populations had identical palatal plicae to those of the holotype, but in contrast, had a much longer anterior lamella, free from the lower plica or almost united to it. Additionally, in the type series, the sculptured dorsal surface changes to a smooth surface at around the middle line of the body whorl. In contrast, in the two newly-collected samples the change between the two different sculptures occurs lower, closer to the umbilicus.

In a shell from another population (Vn10-125, see Figures 14X–Y) the palatal plicae were greatly reduced in length so that when viewed through the semi-transparent shell, they appear as though only a single vertical plica was present. The parietal wall of the same shell was ornamented by a strong anterior lamella entirely fused with the lower plica; the posterior lamella was absent, its position was indicated only by a very slight elevation within the structure of the shell.

#### Gudeodiscus (Gudeodiscus) villedaryi

Taxon classificationAnimaliaStylommatophoraPlectopylidae

(Ancey, 1888)

Figures 8B–D , 9J , 10B , 13V–Y , 23 , 24 , 28F–G , 30A–C , 30F , 32D , 35M–O

Plectopylis Villedaryi Ancey 1888, Le Naturaliste 2 (10): 71–72., Fig. 2. [“Région de Lang-son et de Bac-ninh”].
Plectopylis villedaryi , — Gude 1897b, Science Gossip, 4: 139., Figs 60 a–b. [“Lang-son and Bac-ninh, Tonkin”].
Plectopylis villedaryi , — Gude 1899a, Science Gossip, 5: 332.
Plectopylis (Endoplon) villedaryi , — Gude 1899c, Science Gossip, 4: 148.
Plectopylis (Endoplon) villedaryi , — Gude 1899d, Science Gossip, 6: 175.
Plectopylis Villedaryi , — Gude 1900, The Annals and Magazine of Natural History, 7 (5): 313.
Plectopylis villedaryi , — Gude 1901c, Journal of Malacology, 8: 116–117., Figs 5a–e. [“Than-Moi”].
Plectopylis (Endoplon) choanomphala Möllendorff 1901, Nachrichtsblatt der Deutschen Malakozoologischen Gesellschaft, 33 (5/6): 75. [“Than-moi”].
Plectopylis (Endoplon) villedaryi , — Gude 1901c, Journal of Malacology, 8: 116–117., Figs 5a–e. [“Than-Moi”].
Plectopylis Villedaryi , — Dautzenberg & Fischer 1905a, Journal de Conchyliologie, 53: 93. [“Dong-Trieu, dans les racines des arbustes qui poussent sur des rochers à ceux de la baie d’Along”].
Gudeodiscus villedaryi , — Páll-Gergely & Hunyadi 2013, Archiv für Molluskenkunde, 142 (1): 8.

##### Types examined.

Haut-Tonkin, NHMUK 1930.9.12.38 (holotype of *villedaryi*, Figure 8C); Tonking, Than-Moi, collection Möllendorff ex Fruhstorfer, SMF 9279 (lectotype of *choanomphala*, Figure 8B); Tonking, Than-Moi, SMF 9276 (paralectotype of *choanomphala*).

##### Museum material examined.

Tonkin, Nja-Ba-Thà, coll. Dosch ex Rolle, SMF 172084/4; Tonkin, Mui-Cho, SMF 172095/4; Tonkin, Than-Moi, coll. Ehrmann ex Fruhstorfer, SMF 150133/2; Tonkin, Muc Cho Nja Ba, coll. Jaeckel, S. H., SMF 207680/3; Tonkin, Mui Aro Nja Ba Thà, HNHM 9576/1; Than-Moi, coll. Letellier, 1949, MNHN-IM-2012-2306/3; Than-Moi, coll. Staadt, 1969, MNHN-IM-2012-2321/2; Than-Moi, coll. Staadt, 1969, MNHN-IM-2012-2335/10; Indo-China, coll. Krempf, MNHN-IM-2012-2400/7 juvenile shells; Tonkin, Nju Ba Thá, coll. Rolle, NHMW 50856/2; Tonkin, coll. Fruhstorfer, NHMW 40848/1; Tonkin, Phu-Ty, coll. Edlauer ex Rolle, NHMW 75000/E/7804/2; “China”, coll. Rolle, NHMW 71640/O/12303/1; Tonkin, Moi-Cho-Nja, coll. Rušnov ex Rolle ex Messager, NHMW 92586/2; Tonkin, Than Moi, coll. Edlauer ex Rolle, NHMW 75000/E/7816/3; Tonkin, Nja-Ba-Thá (?), coll. Rušnov ex Blume, NHMW 92584/1; Tonkin, Than-Moi, coll. Rušnov ex Rolle ex Messager, NHMW 92585/1; Tonkin, Than-Moi, coll. Käufel ex Klemm, NHMW 79000/K/17482/2; Tonkin, Cho-Moi, coll. Rolle, NHMW 71640/O/12302/1.

##### New material examined.

**Vn10-47A** Thái Nguyên Province, ca. 4 km NE of Đình Cả, Phượng Hoàng Cave, 21°46.554'N, 106°07.210'E, leg. Hemmen, Ch. & J., 18.03.2010., PGB/3; **20090520A** Thái Nguyên Province, Võ Nhai District, Phú Thượng Commune, Phượng Hoàng Cave, Mỏ Gà Vill., ca 150 m, 21°46.836'N, 106°07.107'E, leg. Ohara, K., 20.05.2009., OK/15, PGB/4 (anatomically examined, Figures 24, 28G, 32D); **Vn10-128** Lạng Sơn Province, ca. 69 km from Thái Nguyên to Bắc Sơn (right side off road), 21°54.270'N, 106°15.801'E, leg. Hemmen, Ch. & J., 22.10.2010., PGB/1; **2012/58** Thái Nguyên Province, northern edge of Lâu Thượng, 5 km W of Ðình Cả, 105 m, 21°44.484'N, 106°01.420'E, leg. Hunyadi, A., 04.06.2012., HA/4; **2011/65** Lạng Sơn Province, Đồng Mỏ 2.5 km towards Văn Quan, right side of the road, 270 m, 21°40.358'N, 106°34.783'E, leg. Hunyadi, A., 10.11.2011., HA/7+2jb, PGB/1; **2011/68** Lạng Sơn Province, Đồng Mỏ 7 km towards Văn Quan, Vạn Linh cross., left side of the road, 370 m, 21°41.158'N, 106°33.588'E, leg. Hunyadi, A., 10.11.2011., HA/1; **2011/76** Lạng Sơn Province, northern edge of Chi Lăng, pass next to the tourist path (N of Đồng Bành) 75 m, 21°34.945'N, 106°30.567'E, leg. Hunyadi, A., 13.11.2011., HA/15+1jb, PGB/2; **2011/79** Lạng Sơn Province, Đồng Mỏ 5.2 km towards Chi Lăng, right side of the road, 40 m, 21°37.215'N, 106°32.538'E, leg. Hunyadi, A., 13.11.2011., HA/3; **2011/102** Thái Nguyên Province, Ðình Cả NE 4 km, Phượng Hoàng cave, around the entrance of the cave, 365 m, 21°46.782'N, 106°07.189'E, leg. Hunyadi, A., 13.11.2011., HA/25+2jb, PGB/2 (anatomically examined, Figures 23, 28F, 30A–C, 30F, 35M–O); **2012/38** Lạng Sơn Province, Đồng Mỏ 4–5 km towards Chi Lăng, right side of the old road, 65 m, 21°37.479'N, 106°32.730'E, leg. Hunyadi, A., 25.05.2012., HA/12+1jb; **Vn11-159** Lạng Sơn Province, at km 74.8 on road 1B, Đồng Đăng to Thái Nguyên (8 km S Bắc Sơn), 21°54.543'N, 106°17.298'E, leg. Hemmen, Ch. & J., 02.04.2011., HE/1; **Vn11-163** Lạng Sơn Province, road 242 from Đình Cả to Hữu Lũng, SE Bình Long, 21°38.424'N, 106°11.761'E, leg. Hemmen, Ch. & J., 02.04.2011., HE/9; **Vn11-151** Thái Nguyên Province, ca. 48 km from Thái Nguyên to Bắc Son, near Lâu Thượng (SW Đình Cả), 21°43.522'N, 105°58.662'E, leg. Hemmen, Ch. & J., 29.03.2011., HE/8; **Vn11-161** Lạng Sơn Province, at km 90.5 on road 1B Đồng Đăng to Thái Nguyên, 21°49.656'N, 106°12.636'E, leg. Hemmen, Ch. & J., 02.04.2011., HE/1; **Vn11-152** Lạng Sơn Province, road 1B, ca. 23 km SE Bắc Sơn (between Đình Cả and Bắc Sơn), 21°49.155'N, 106°11.448'E, leg. Hemmen, Ch. & J, 29.03.2011., HE/3.

##### Diagnosis.

Shell medium-sized to large, strongly-built, nearly smooth, with thick apertural lip and an oblique, strong apertural fold (Figure 9J); umbilicus frequently keeled. The anterior parietal lamella is supported by an anteriorly elongated lower plica; an additional, long horizontal plica is present near the lower suture; middle palatal plicae oblique (Figures 13V–Y).

##### Measurements

(in mm): D = 19.5–21.7, H = 11–12.6 (n=4, Vn11-163); D = 15.4–18.4, H = 7.8–8.9 (n=3, Vn11-151); D = 21–23.4, H = 11.3–12.6 (n=3, Vn11-152); D = 15.4–16.5, H = 8.4–9.5 (n=3, 20090520A); D = 16.7–20.6, H = 8.9–9.8 (n=3, Vn10-42); D = 16.1–17.8, H = 7.9–9.2 (n=2, Vn10-44).

##### Differential diagnosis.

See under *Gudeodiscus dautzenbergi* and *Halongella schlumbergeri*.

##### Intraspecific diversity.

The morphology of palatal and parietal plicae and lamellae do not show significant variation. Conversely, shell size, aperture shape, shape of the dorsal side of the shell, spire height and the presence or absence of the periumbilical keel show considerable variation across populations. See also Table 11.

**Table 11.** Diversity of the periumbilical region within Gudeodiscus (Gudeodiscus) villedaryi.

code	keel
2012/58	absent
2011/65	present
2011/68	present
2011/76	present
2011/79=2012/38	present
2011/102= Vn10-47=20090520A	present
Vn10-128	slight keel
Vn11-159	slight keel
Vn11-151	slight keel
Vn11-152	absent
Vn11-161	slight keel
Vn11-163	present

##### Description of the genitalia.

Three specimens were anatomically examined; they were collected at the same locality at different times of the year (20090520A: 20 May, two specimens; 2011/102: 12 November, one specimen). One of the specimens from the 20090520A sample had abnormally developed genitalia. Namely, the penis was “normally” connected to the genital opening, but the vagina was only attached to the atrium area with weak fibres. Nevertheless, the gametolytic sac was filled with fragments of a spermatophore which is an indication of successful mating. An epiphallus was absent and the vas deferens started from the base of the vagina. The other specimen from the 20090520A sample (collected in May) had 18 embryos developed in its uterus, and had no claws between the folds on the inner wall of the penis, whereas the one collected in November was not gravid, but had several claws within the folds inside the penis. The claws had a moderately long base inside the pockets, whereas their hook-like tip was hanging out of the pockets. The SEM images revealed that the base had a granulated surface, probably to provide a better attachment to wall of the pockets, whereas the tip was smooth. Additionally, the specimen from November had parallel, dense, wavy, horizontal folds on the inner wall of the proximal part of the penis, and longitudinal, parallel folds on the distal portion of the penis. The other specimen sampled in May had only a slightly waved proximal part of the longitudinal folds. Other parts of the genitalia did not differ between the two specimens.

The penis is short, pear-shaped internally with pockets standing in a straight row at the distal part of the penis; the epiphallus is much more slender, and is somewhat shorter than the penis; there is no penial caecum, the retractor muscle attaches on the apical part of the penis (at the penis-epiphallus transition); epiphallus approximately as long as the penis, it transforms to vas deferens without obvious boundary; epiphallus internally with parallel folds; vagina long with a well-developed vaginal bulb, it is attached to the body wall with several ligaments; vaginal bulb with thickened wall, internally almost smooth, only with hardly visible longitudinal folds; inner wall of the distal part of the vaginal with low, parallel or converging, serrulate folds (Figure 32D); there is a shorter, thicker gametolytic sac and a longer, more slender diverticulum.

##### Radula.

See Table 6 and Figures 35M–O.

##### Distribution

(see Figure 40). The species is known from Thái Nguyên and Lạng Sơn provinces.

##### Remarks.

*Gudeodiscus villedaryi* is a very variable species in terms of shell characters. The species is recognised on the basis of the presence of an additional lower plica, which is absent in *Gudeodiscus dautzenbergi*. The latter species might be only a variety of *Gudeodiscus villedaryi* which has lost the lower plica. More information is needed to determine whether the populations assigned to *Gudeodiscus villedaryi* and *Gudeodiscus dautzenbergi* form monophyletic groups. See also under *Gudeodiscus dautzenbergi*.

#### Subgenus Veludiscus

Taxon classificationAnimaliaStylommatophoraPlectopylidae

Páll-Gergely subgen. n.

##### Type species.

*Gudeodiscus eroessi* Páll-Gergely & Hunyadi, 2013.

##### Diagnosis.

Shell indistinguishable from those of the subgenus Gudeodiscus (Gudeodiscus) and the genus *Halongella* gen. n. Anatomy: Epiphallus is slender, cylindrical; retractor muscle inserts on the distal end of the penial caecum, but the whole caecum is covered by additional, fine muscle fibres which insert on the distal end of the penis. Radula: central tooth smaller than the ectocone of the first lateral; mesocone of the first lateral is usually wide, rhomboid. Marginals bi- or tricuspid, with blunt inner cusp and shallow incision between the inner two cusps. See drawings and descriptions of the genital anatomy in Páll-Gergely and Hunyadi (2013) and Páll-Gergely and Asami (2014).

##### Content.

*emigrans* (Möllendorff, 1901), *eroessi* Páll-Gergely & Hunyadi, 2013, *goliath* Páll-Gergely & Hunyadi, 2013(?), *okuboi* Páll-Gergely & Hunyadi, 2013, *pulvinaris* (Gould, 1859).

##### Etymology.

The name *Veludiscus* is composed of two Latin words. Velum (=curtain, sail, covering) refers to the characteristic feature of the genitalia, namely the additional curtain-like muscle covering the penial caecum and the retractor muscle, and discus (=disc) refers to the shape of the shell. The genus is gender masculine.

##### Remarks.

Some conchologically similar species may belong to this subgenus, especially those which inhabit similar geographic regions. Future investigations on the anatomy and radula morphology of *Gudeodiscus* species should clarify the subgeneric status of the taxa with unknown anatomy.

#### Gudeodiscus (Veludiscus) emigrans

Taxon classificationAnimaliaStylommatophoraPlectopylidae

(Möllendorff, 1901)

##### Diagnosis.

A medium-sized to large species with dense, fine riblets; shell flat, callus always, apertural fold usually present. Parietal wall with C-shaped posterior lamella; anterior lamella (if present) slightly S-shaped; if anterior lamella is missing; one lower plica or four parallel plicae are visible in front of the lamella; palatal wall with almost straight, slightly oblique, depressed Z-shaped or Y-shaped plicae (Figures 13A–D).

##### Differential diagnosis.

*Gudeodiscus phlyarius* has stronger apertural fold, a straight anterior parietal lamella (in the Chinese populations assigned to *Gudeodiscus phlyarius werneri* Páll-Gergely, 2013 = synonym of *phlyarius*, sometimes dissolved into small denticles) and usually a somewhat elevated spire. *Gudeodiscus messageri*, *Gudeodiscus hemmeni* sp. n. and *Gudeodiscus anceyi* have two parietal lamellae or several small denticles standing in a line at the position of the first lamella.

##### General distribution.

The three subspecies of *Gudeodiscus emigrans* are known from northern Vietnam and northern Guangxi.

#### Gudeodiscus (Veludiscus) emigrans emigrans

Taxon classificationAnimaliaStylommatophoraPlectopylidae

(Möllendorff, 1901)

Figures 6E , 13A–B

Plectopylis (Sinicola) emigrans Möllendorff 1901, Nachrichtsblatt der Deutschen Malakozoologischen Gesellschaft, 33 (5/6): 75, 76. [“Mansongebirge”]
Gudeodiscus emigrans emigrans , — Páll-Gergely & Hunyadi 2013, Archiv für Molluskenkunde, 142 (1): 12., Figs 24, 44a–b, 58 (map).

##### Material examined.

See Páll-Gergely and Hunyadi (2013).

##### Diagnosis.

Spiral sculpture missing or not conspicuous, parietal wall with one lamella and a short lower parietal plica anterior to the lamella.

##### Measurements

(in mm). D = 17.3, H = 7.5 (holotype).

##### Differential diagnosis.

*Gudeodiscus emigrans emigrans* has weaker spiral sculpture than *Gudeodiscus emigrans quadrilamellatus*, and has only one horizontal parietal plica anterior to the lamella (close to the lower suture), whereas *Gudeodiscus emigrans quadrilamellatus* has four parallel horizontal plicae. The Chinese *Gudeodiscus emigrans otanii* has Y-shaped palatal plicae (these are simple in the nominotypical subspecies and in *Gudeodiscus emigrans quadrilamellatus*). Moreover, some specimens of *Gudeodiscus emigrans otanii* have two vertical lamellae (see Páll-Gergely and Asami 2014).

##### Intrasubspecific diversity.

Very few shells are known from museum collections. The subspecies is easily recognisable, but more material is needed to understand the intrasubspecific diversity.

##### Distribution.

Plectopylis (Sinicola) emigrans was described from the “Manson-Gebirge” = “Mau Son Mts, about 30 km E of Lang Son” (Schileyko 2011) (see Figure 39).

#### Gudeodiscus (Veludiscus) emigrans quadrilamellatus

Taxon classificationAnimaliaStylommatophoraPlectopylidae

Páll-Gergely, 2013

Figures 6F , 13C–D

Plectopylis emigrans Gude 1901a, Journal de Conchyliologie, 49: 206–208. Plate 6., Figs 5a–c. [“Bac Kan, secteur de Nac Ri, Baie d’Along”].
Gudeodiscus emigrans quadrilamellatus Páll-Gergely in Páll-Gergely & Hunyadi 2013, Archiv für Molluskenkunde, 142 (1): 15–17., Figs 27, 45a–b, 58 (map).

##### Material examined.

Samples not mentioned in Páll-Gergely and Hunyadi (2013) are the following: Hạ Long Bay, leg. Messager, MNHN-IM-2012-2320/1; Indochine, leg. Messager, MNHN-IM-2012-2455/2; Tonkin, coll. Letellier 1949, MNHN-IM-2012-2448/1.

##### Diagnosis.

Spiral sculpture conspicuous, parietal wall with one lamella and four parallel horizontal plicae in front of the single lamella.

##### Measurements

(in mm): D = 17.7–18.6, H = 7.1–7.6 (n=3, sample from the type locality).

##### Differential diagnosis.

See under *Gudeodiscus emigrans emigrans*.

##### Intrasubpecific diversity.

Low; shell characters are stable. The subspecies is easily recognisable and can be separated from other Vietnamese and Chinese taxa without problems.

##### Distribution

(see Figure 42): *Gudeodiscus emigrans quadrilamellatus* is known from Bắc Kạn and Tuyên Quang Provinces. Museum samples are labelled from Tam Đảo, on the border region of Thái Nguyên and Vĩnh Phúc Provinces (Au Nord de Ha Noi, Tam Dao, MNHN-IM-2012-2123/3). Records from the Hạ Long area (e.g. Gude 1901a) are probably incorrect (see also Páll-Gergely and Hunyadi 2013).

#### Genus Halongella

Taxon classificationAnimaliaStylommatophoraPlectopylidae

Páll-Gergely gen. n.

http://zoobank.org/F77AFB6D-87D8-4F33-B3F7-0F0F859A783F

##### Type species.

Helix (Plectopylis) Schlumbergeri Morlet, 1886.

##### Diagnosis.

Shells do not differ from those of *Gudeodiscus*; small to very large, body whorl rounded, callus and apertural fold; Parietal wall with two lamellae or the anterior one is reduced or absent; parietal side with straight, slightly curved, or depressed Z-shaped plicae.

Penial caecum absent. Penis internally with longitudinal, parallel folds, with tiny, flat, T-shaped calcareous granules between the folds, all along the penis; there are no determined “pockets” for the granules at the apical part of the penis. Epiphallus internally with longitudinal folds having several perpendicular projections which overlap with those of the neighbouring fold. Radula similar to Gudeodiscus (Veludiscus) subgen. n. by the smaller central tooth than the ectocone of the first laterals and the marginals which are bicuspid or tricuspid with blunt innermost cups and shallow incision between the two inner cusps.

##### Differential diagnosis.

*Sinicola* species have a keeled body whorl, whereas it is rounded in *Halongella* gen. n. Moreover, all *Sinicola* species have a penial caecum, a central tooth which is as large as or larger than the ectocone of the first laterals and clearly tricuspid marginals with deep incision between the innermost two, sharp cusps. The same radular morphology has been observed in *Sicradiscus* species. Additionally, “eastern” *Sicradiscus* species possess keeled shells, whereas the rounded shelled “western” species of the genus have determined pockets on the inner penial wall, similar to that of *Gudeodiscus*. For comparison with *Gudeodiscus*, see there.

##### Included taxa.

*fruhstorferi* Möllendorff, 1901 and *schlumbergeri* Morlet, 1886.

##### Etymology.

This generic name derives from the name of the Halong Bay, where both species occur. The genus is gender feminine.

##### Remarks.

Calcareous granules of complicated shape have been found in the vagina of *Halongella schlumbergeri*, and some granules not having characteristic shapes have been found in the vaginal lumen of *Halongella fruhstorferi*. The taxonomic value of these granules are unknown. No granules of characteristic shape have been found in the vaginas of *Gudeodiscus* species, therefore this can be a synapomorphy of *Halongella* gen. n.

#### Halongella fruhstorferi

Taxon classificationAnimaliaStylommatophoraPlectopylidae

(Möllendorff, 1901)

Figures 7D , 9O , 14O–R , 25 , 29C , 29I , 32A–B , 36A–C

Plectopylis (Sinicola) fruhstorferi Möllendorff 1901, Nachrichtsblatt der Deutschen Malakozoologischen Gesellschaft, 33(5/6): 114–115. [no locality specified].
Plectopylis (Sinicola) fruhstorferi , — Gude 1901c, Journal of Malacology, 8: 112–113., Figs 2a–e. [“Kebao”].
Plectopylis fruhstorferi , — Gude 1915, Records of the Indian Museum, 8: 513.
Gudeodiscus fruhstorferi , — Páll-Gergely & Hunyadi 2013, Archiv für Molluskenkunde, 142 (1): 8.

##### Types examined.

Tonkin, Kebao, collection Möllendorff ex Fruhstorfer 128, SMF 9258 (lectotype); Tonkin, Kebao, collection Möllendorff ex Fruhstorfer 128, SMF 9259 (paralectotype).

##### Museum material examined.

Tonkin, Kebao (Insel), SMF 150081/2; Kebao, leg. Fruhstorfer, 29.10.1900, RBINS/2; Kebao, coll. Rolle, NHMUK 20110239/2; Kebao, NHMUK 1901.12.23.41–43/3; Tonkin, NHMUK 1916.3.16.9/1.

##### New material examined.

**Vn11-171** Quảng Ninh Province, Vân Đồn Island (NE Cẩm Phả), Cái Rồng village, 21°3.560'N, 107°25.551'E, leg. Hemmen, Ch. & J., 14.08.2011., HE/23, HA/1, PGB/3 (anatomically examined, Figures 25, 29C, 29I, 32A–B, 36A–C).

##### Diagnosis.

Shell small, solid, thin-walled, almost flat and smooth, with weak apertural lip and sometimes a small apertural denticle (Figure 9O). Parietal wall with one parietal lamella with two short horizontal plicae anteriorly, one above and one below; palatal plicae short, oblique, depressed Z-shaped (Figures 14O–R).

##### Measurements

(in mm). D = 13.1–13.4, H = 5.8–6 (n=2, Vn11-171).

##### Differential diagnosis.

*Halongella fruhstorferi* and *Halongella schlumbergeri* are congeneric based on similarity of genital morphology. *Halongella fruhstorferi* is smaller than *Halongella schlumbergeri*, having a more fragile, lighter shell and weaker apertural lip and apertural fold. In shape, *Halongella fruhstorferi* resembles *Gudeodiscus fischeri*. However, *Halongella fruhstorferi* has a relatively smaller aperture, weaker sculpture (rather irregular growth lines instead of regular ribs) and an anterior lamella is absent. *Gudeodiscus phlyarius* and the similar species (*Gudeodiscus anceyi*, *Gudeodiscus hemmeni* sp. n., *Gudeodiscus messageri*) have a well-developed anterior lamella or denticles at the position of the anterior lamella.

##### Intraspecific diversity.

The species is known from a very small area, and only few specimens are known. The intraspecific diversity is low.

##### Description of the genitalia.

One specimen was examined anatomically. Locality: Quảng Ninh Province, Vân Đồn Island (NE Cẩm Phả), Cái Rồng village, 21°3.560'N, 107°25.551'E, leg. Hemmen, Ch. & J., 14.08.2011. (Figures 25, 29C, 29I, 32A–B).

Penis relatively long, spindle-shaped, inner wall with several (at least 20) parallel running folds (Figure 29C); between the folds flat and very fine calcareous granules were found; epiphallus shorter than the penis, its inner wall with six parallel folds; on the distal portion of the epiphallus the longitudinal folds have several perpendicular projections which overlap with those of the neighbouring fold (Figure 29I); penial caecum absent, the retractor muscle inserts on the penis-epiphallus transition. Vagina long, with a relatively well-developed vaginal bulb; it is attached to the body wall by connective tissue; inner wall of the vagina with at least 16, more or less parallel folds; a few irregularly shaped calcareous granules have been found between the folds (Figure 32A–B); stalk of gametolytic sac longer with thickened gametolytic sac, diverticulum slimmer without conspicuous distal thickening. There were two developing embryos in the uterus. The embryos were surrounded with egg capsules which had several calcareous granules.

##### Radula.

See Table 6 and Figures 36A–C.

##### Distribution

(see Figure 40): The species is known only from Kebao Island (Hạ Long Bay area).

#### Halongella schlumbergeri

Taxon classificationAnimaliaStylommatophoraPlectopylidae

(Morlet, 1886a)

Figures 6A–D , 9M–N , 14H–N , 26 , 29A–B , 29H , 30G–I , 33A–G , 36D–F , 45B

Helix (Plectopylis) Schlumbergeri Morlet 1886a, Journal de Conchyliologie, 34: 259, 272–274., Plate 12., Figs 2a–c. [“Baie d’Along et montagne de l’Éléphant”].
Helix (Plectopylis) Schlumbergeri Morlet 1886b, Diagnoses de mollusques terrestres et fluviatiles du Tonkin. 1–2.
Plectopylis Schlumbergeri , — Mabille 1887b, Bulletin de le Société Malacologique de France, 4: 101–102.
Plectopylis jovia Mabille 1887b, **syn. n.**, Bulletin de le Société Malacologique de France, 4: 99–100. [“Circa locum dictum Halong”].
Helix schlumbergeri , — Tryon 1887, Manual of Conchology. 2 (3): 166, Plate 36., Figs 25–28.
Plectopylis Schlumbergeri , — Ancey 1888, Le Naturaliste, 2(10): 72.
Plectopylis jovia , — Pilsbry 1893, Manual of Conchology..., 2 (8): 156–157.
Plectopylis villedaryi , — Pilsbry 1893, Manual of Conchology..., 2 (8): 158., Plate 43., Figs 36–39.
Plectopylis jovia , — Pilsbry 1894, Manual of Conchology...: 146., Plate 40., Figs 1–4.
Plectopylis schlumbergeri , — Gude 1897b, Science Gossip, 4: 138., Figs 58a–b. [“Halong Bay and Elephant Mountain, Tonkin”].
Plectopylis jovia , — Gude 1897b, Science Gossip, 4: 138–139., Figs 59a–b. [“Halong”].
Plectopylis schlumbergeri , — Gude 1899a, Science Gossip, 5: 332.
Plectopylis jovia , — Gude 1899a, Science Gossip, 5: 332.
Plectopylis (Endoplon) schlumbergeri , — Gude 1899c, Science Gossip, 4: 148.
Plectopylis (Endoplon) jovia , — Gude 1899c, Science Gossip, 4: 148.
Plectopylis (Endoplon) schlumbergeri , — Gude 1899d, Science Gossip, 6: 175.
Plectopylis (Endoplon) jovia , — Gude 1899d, Science Gossip, 6: 175.
Plectopylis (Endoplon) hirsuta Möllendorff 1901, **syn. n.**, Nachrichtsblatt der Deutschen Malakozoologischen Gesellschaft, 33 (5/6): 114–115. [“in insula Bah-mun”].
Plectopylis Schlumbergeri , — Gude 1901a, Journal de Conchyliologie, 49: 199.
Plectopylis Villedaryi , — Gude 1901a, Journal de Conchyliologie, 49: 212. [“Llots de la baie d’Along”].
Plectopylis jovia , — Gude 1901b, Journal of Malacology, 8: 47–48., Figs 1a–b.
Plectopylis schlumbergeri , — Gude 1901b, Journal of Malacology, 8: 47–48., Figs 2a–b.
Plectopylis villedaryi , — Gude 1901b, Journal of Malacology, 8: 47–48., Figs 3a–b.
Plectopylis pilsbryana Gude 1901c, **syn. n.**, Journal of Malacology, 8: 110., [“Lang-Son, Bac-Ninh (Vathelet). Isles in Along Bay (Messager). Tonkin (Fruhstorfer)”].
Plectopylis (Endoplon) hirsuta , — Gude 1901c, Journal of Malacology, 8: 111–112., Figs 1a–f. [“Island Bah-Mung”].
Plectopylis (Endoplon) jovia , — Gude 1901c, Journal of Malacology, 8: 111–112., Figs 1a–f.
Plectopylis Schlumbergeri , — Dautzenberg & Fischer 1905a, Journal de Conchyliologie, 53: 93.
Plectopylis jovia , — Dautzenberg & Fischer 1905a, Journal de Conchyliologie, 53: 93.
Plectopylis Villedaryi , — Dautzenberg & Fischer 1905a, Journal de Conchyliologie, 53: 93.
Gudeodiscus schlumbergeri , — Páll-Gergely & Hunyadi 2013, Archiv für Molluskenkunde 142 (1): 8.
Gudeodiscus pilsbryana , — Páll-Gergely & Hunyadi 2013, Archiv für Molluskenkunde, 142 (1): 8.
Gudeodiscus jovius , — Páll-Gergely & Hunyadi 2013, Archiv für Molluskenkunde, 142 (1): 8.
Gudeodiscus hirsutus , — Páll-Gergely & Hunyadi 2013, Archiv für Molluskenkunde, 142 (1): 8.

##### Types examined.

Llots de la Baie d’Along, leg. Messager (n. 23.), MNHN IM-2010-12119. (cited in Journal de Conchyliologie, 49: 212. as *villedaryi*); Tonkin, Halong, leg. l’Abbé Vathelet, MNHN 24580 (one adult and one juvenile syntypes of *jovia*, Figure 6B); Tonkin, NHMUK 1922.8.29.52 (holotype of *pilsbryana*, Figure 6C); Tonkin, MNHN 24582 (2 syntypes of *schlumbergeri*, Figure 6A); Tonkin, Bah-Mun, coll. Möllendorff ex Fruhstorfer, SMF 9277 (lectotype of *hirsuta*, Figure 6D); same data, SMF 9278 (2 paralectotypes of *hirsuta*).

##### Museum material examined.

Tonkin, That-Khé, coll. Dosch ex Rolle ex Messager, SMF 341737/2; Tonkin, ex Fruhstorfer, SMF 150132/2; Tonkin, Tafel Insel, ex Fruhstorfer, H. 126, SMF 150131/2; Tonkin, Isle de la Table, coll. Ehrmann ex Webb, W. F., SMF 150130/3; Tonkin, Isle de la Table, coll. Ehrmann ex Webb, W. F., SMF 150124/1; Tonkin, coll. Ehrmann ex Fruhstorfer, H., SMF 150123/1; Tonkin, rochers de Kuy-Dong-Kay, coll. Jaeckel, S. H., SMF 207677/2; Tonkin, Isle de la Table, SMF 207678/1; Tonkin, rochers de Nuy-Dong-Nay, coll. Schlickum 3969 ex Staid (?), SMF 277560/2; Tonkin, Than-Moi, coll. Jaeckel, S. H., SMF 207670/4; Tonkin, rochers de Nuy-Dong-Nuy, coll. Pfeiffer, K. L. ex Sundler, October 1940, SMF 102825/2; Tonkin, Ile de la Table, Baie d’Along, SMF 294868/2; Tonkin, coll. Dosch ex Rolle, SMF 172096/2; Tonkin, Ile de la Table, coll. Dosch ex Rolle ex Webb, SMF 172094/2; Tonkin, Ile des Merveilles, coll. Möllendorff ex Fruhstorfer 130, SMF 150129/2; Tonkin, Hai-fong, coll. Möllendorff ex Fruhstrofer, SMF 150128/1; Hongay, leg. Drimmer, 09.11.1986. ex Kovács, Gy., HNHM 67079/2; Hongay, leg. Drimmer, 09.11.1986., HNHM 78324/4; Nuy Dong Nay, leg. Drimmer, HNHM 67068/1; Tonkin: Roches de Nuy-Dong-Nay HNHM 37877/2; Tonkin, coll. Mansuy, MNHN-IM-2012-2260/4; Tonkin, coll. Sayer 1969, MNHN-IM-2012-2261/1; Tonkin, leg. abbe Wathelet, MNHN-IM-2012-2262/3; Baie d’Along, Ile de le Table, coll. Lavezzari ex Bernays, MNHN-IM-2012-2264/3; Tonkin, coll. Balansa 1887, MNHN-IM-2012-2269/4; Baie d’Halong, excoll. labo. de Géologie de la Sorbonne (entrée 1952), MNHN-IM-2012-2271/2; Baie d’Halong, coll. Staadt, 1969, MNHN-IM-2012-2280/1 juvenile shell; Baie d’Along, Ile de la Table, MNHN-IM-2012-2289/3; Tonkin, coll. Staadt 1969, MNHN-IM-2012-2291/24; Tonkin, coll. Balansa 1887, MNHN-IM-2012-2294/4; Halong, MNHN-IM-2012-2295/2; Grotte des Merveilles, coll. Saurin, MNHN-IM-2012-2299/7; Tonkin, coll. Letellier, 1949, MNHN-IM-2012-2304/1; Halong Bay, leg. Messager, MNHN-IM-2012-2316/1; Halong Bay, leg. Messager, MNHN-IM-2012-2317/4; Halong Bay, leg. Messager, MNHN-IM-2012-2322/4; No locality, leg. V. Demange, 29.01.1931, coll. Staadt, 1969, MNHN-IM-2012-2329/298; Tonkin, coll. Denis, MNHN-IM-2012-2332/6; Rochers de Nuy-Dong-Nay, MNHN-IM-2012-2481/529; Tonkin, coll. Staadt. 1969, MNHN-IM-2012-2444/366; Dong-Trien, coll. Blaise, 1902, MNHN-IM-2012-2347/1; Dong-Trien, coll. Blaise, 1903, MNHN-IM-2012-2348/1; Ile de la Table, coll. Staadt, 1969, MNHN-IM-2012-2350/4; Ile Krieu, coll. Blaise, MNHN-IM-2012-2362/2 juvenile shells; Lang-Son, coll. Letellier, 1949, MNHN-IM-2012-2366/1; Ile de la Table, coll. Demange, MNHN-IM-2012-2367/5; Dong-Trieu, coll. Blaise, MNHN-IM-2012-2368/2; Halong Bay, leg. Messager, MNHN-IM-2012-2369/3; Halong Bay, leg. Messager, MNHN-IM-2012-2370/3; Halong Bay, leg. Messager, MNHN-IM-2012-2375/6; Tonkin, coll. Fischer, ex Crosse, MNHN-IM-2012-2380/2; Ilots de la Baie d’Along, leg. Messager, MNHN-IM-2012-2381/2; Tonkin, leg. Messager, MNHN-IM-2012-2388/1; Tonkin, coll. Lucas, Acc. no. 2351, NHMUK 20130622/2; Hanoi, Ile de la Table, coll. Biggs, H.E.J. ex Tomlin, 1931, Acc. no. 2258, NHMUK 20130623/8; Tonkin, coll. Salisbury ex Beddome, NHMUK 20130624/1; Tonkin, Ile de la Table, NHMUK 20130625/4; Tonkin, Ile de la Table, NHMUK 1901.12.12.211–212/2; Tonkin, Ile des Merveilles, NHMUK 1901.12.12.232–233/2; Tonquin, NHMUK 1889.9.23.1. (2 shells); Tonkin, Bah-Mun, coll. Dosch ex Rolle, SMF 172085/2 (“*hirsuta*”); Tonkin, Bah-Mun, coll. Ehrmann ex Fruhstorfer, SMF 150137/2 (“*hirsuta*”); Bah-Mun, leg. Fruhstorfer, 29.10.1900, RBINS/2 (“*hirsuta*”); Golfe de Tonkin, coll. Achat Boubée, MNHN-IM-2012-2307/1 (“*hirsuta*”); Tonkin, coll. Salisbury ex Beddome, NHMUK 20110254/1 (“*hirsuta*”); Tonkin, coll. Rolle, 4/11/01-25, NHMUK 20110264/3 (“*hirsuta*”); Tonkin, NHMUK 1916.3.16.10/1 (“*hirsuta*”); Tonkin, Bah-Mun, NHMUK 1901.12.23.32–34/3 (“*hirsuta*”); Tonkin, That-Khé, coll. Werner ex Rolle, NHMW 75000/E/7814/2; Tonkin, That-Khé, coll. Klemm, NHMW 79000/K/17484/3; Golf de Tonking, Ile de la Table, coll. Edlauer, NHMW 75000/E/14744/2; Tonkin, Ile Table, coll. Rušnov, NHMW 92583/2; Ile de la Table, Ban Valong (?), coll. Oberwimmer ex Caziot, NHMW 71640/O/9650/2; Tonkin, Ile de la Table, NHMW 92582/2; Tonkin, coll. Fruhstorfer, NHMW 40849/1; Tonkin, That-Ke, coll. Wagner ex Messager, NHMW 103351/2 (mixed sample with *dautzenbergi*); Tonkin, NHMW 46025/1 (“*hirsutus*”); Cha-Ban, Baie d’Along, Tonkin, coll.Steenberg, ZMUC-GAS-1814/2.

##### New material examined.

**20081119A** Hải Phòng Province, Hải Phòng City, Cát Bà Isl., Cát Bà Nat. Park, beyond Mây Bầu, ca 160 m, 20°47.763'N, 107°00.758'E, leg. Ohara, K. 19.11.2008., PGB/2, OK/13; **20071122B** same data, leg. Okubo, K., 22.11.2007., PGB/2; **20071122A** Hải Phòng Province, Hải Phòng city, Cát Bà Island, Cát Bà Nat. Park, near pass in front of Mây Bầu, ca 100 m, 20°47.81769'N, 107°00.42256'E, leg. Ohara, K., 22.11.2007., OK/4, PGB/1; **20081118A** Quảng Ninh Province, Hạ Long Bay, Đầu Gỗ Isl., near Đầu Gỗ Cave, ca 15 m, 20°54.696'N, 107°01.069'E, leg. Ohara, K., 18.11.2008., OK/14, PGB/2; **GS25** Quảng Ninh Province, Hạ Long Bay, Đầu Gỗ Cave, N. Đầu Gỗ Island, in leaf litter in limestone crackings, leg. Grego, J., 08.04.2012., PGB/1 broken specimen; **20071122C** Hải Phòng City, Cát Bà Island, Cát Bà N.P., beyond Mây Bầu peak, 165 m, 20°47.70504'N, 107°00.85709'E, leg. Ohara, K., 22.11.2007., PGB/1; **MAA7** Quảng Ninh Province, Hạ Long Bay Area, Áng Dù Island, 20°47.61'N, 107°08.05'E, coll. Maassen, W.J.M., 15.09.2003., PGB/2, WM/8; **MAA8** Hải Phòng Province, Cát Bà Island, half way path lake Ao Ek and Park HQ, 20°47.45'N, 107°00.00'E, leg. Vermeulen, J., coll. Maassen, W.J.M., 27.09.2003. (2 shells); **MAA11** Quảng Ninh Province, Hạ Long Bay Area, Tiên Ông Cave on Hang Trai? Island, collected near the entrance of the cave, 20°48.96'N, 107°07.33'E, coll. Maassen, W.J.M. 06.09.2003., (1 shell).; **no code** Quảng Ninh Province, Hạ Long Bay area, Cây Chanh Island, Cống Đỏ area, 20°52.56'N, 107°11.14'E, leg. Hemmen, Ch. & J., 2003, PGB/2 shells+1jb; **MAA5** same data, coll. Maassen, W.J.M., 13.09.2003., PGB/2, WM/14; **MAA2** Quảng Ninh Province, Hạ Long Bay Area, Cống Đỏ Isl., NE coast, 20°52.44'N, 107°12.10'E, leg. Vermeulen, J., 03.10.2003., coll. Maassen, W.J.M., WM/2; **MAA3** Quảng Ninh Province, Hạ Long Bay Area, unnamed island in Cống Đỏ area, 20°52.47'N, 107°11.72'E, coll. Maassen, W.J.M., 03.10.2003., PGB/1, WM/3); **MAA4** Quảng Ninh Province, unnamed island in Đảo Mới Temper area, 20°55.69'N, 107°09.40'E, coll. Maassen, W.J.M., 13.09.2003., PGB/2, WM/18; **MAA6** Quảng Ninh Province, Hạ Long Bay Area, Phao Trong Island, 20°49.80'N, 107°08.32'E, coll. Maassen, W.J.M., 11.09.2003., PGB/1, WM/5; **2012/26** Hải Phòng Province, Ðảo Cát Bà (island), Cát Bà Nat. Park, 500 m from the entrance towards Ao Ếch, 60 m, 20°47.945'N, 106°59.653'E, leg. Hunyadi, A., 22.05.2012., HA/1+2jb; **2012/28** Hải Phòng Province, Ðảo Cát Bà, Cát Bà Nat. Park, Ao Ếch 500 m towards Mây Bầu, 60 m, leg. Hunyadi, A., 22.05.2012., HA/25+1jb; **2012/32** Quảng Ninh Province, Đèo Bụt (pass) 1 km towards Cẩm Phả, right side of the road, 10 m, 20°58.680'N, 107°11.089'E, leg. Hunyadi, A., 23.05.2012., HA/11+1jb; **2012/34** Quảng Ninh Province, ÐảoTrà Bản (island), Cảng Bản Sen (harbour) 1.5 km towards Cảng Tân Lập (harbour), right side of the road, 30 m, 20°56.943'N, 107°29.772'E, leg. Hunyadi, A., 24.05.2012., HA/84+3jb; **2012/35** Quảng Ninh Province, ÐảoTrà Bản (island), Cảng Bản Sen (harbour) towards the Cảng Tân Lập (harbour) cross, 200 m, right side of the road, 35 m, 20°56.456'N, 107°29.870'E, leg. Hunyadi, A., 24.05.2012., HA/12; **Vn11-172** Hải Phòng Province, Cát Bà Island, behind cemetery of Gia Luận village, 20°50.092'N, 106°58.560'E, leg. Hemmen, Ch. & J., 10.04.2011., HE/6 (anatomically examined); **Vn11-173** Hải Phòng Province, Cát Bà Island, at km 4 road Gia Luận village to Cát Bà village, 20°49.991'N, 106°58.382'E, leg. Hemmen, Ch. & J., 10.04.2011., HE/11, PGB/1 (in ethanol); **Vn11-174** Hải Phòng Province, Cát Bà Island, between Hiền Hào and Cát Bà village near Xuân Đán, 20°45.479'N, 106°58.556'E, leg. Hemmen, Ch. & J., 10.04.2011., HE/8; **Vn11-175** Hải Phòng Province, Cát Bà Island, between Hiền Hào and entrance of Cát Bà N.P. (road over Hiền Hào), 20°47.681'N, 106°59.068'E, leg. Hemmen, Ch. & J., 11.04.2011., HE/4; **Vn11-38A** Hải Phòng Province, Cát Bà Island, Hoa Cương Cave (=Dong Da Hoang?), near Gia Luận, ca. 30 m, 20°50.268'N, 106°59.019'E, leg. Hemmen, Ch. & J., 10.04.2011., HE/5; **Vn11-165** Quảng Ninh Province, ca. 8.3 km west of Cẩm Phả ca 200 m right of road 18 (no GPS-data), leg. Hemmen, Ch. & J., 03.04.2011., HE/1; **VERM1** Cát Bà, Hải Phòng Province, Cát Bà Island, path from Nat. Park HQ to lake Ao Ek, 20°47.45'N, 107°00.45'E, Primary forest on limestone. Mainly handpicked. leg. Vermeulen, J.J. & Whitten, A.J., 25.09.1998, NHMUK 19991447/4; **VERM3** Hạ Long Quảng Ninh Province, Hạ Long-Cẩm Phả area. Limestone hill S of Hạ Long, with marked regrowth and bamboo thickets, 20°57.00'N, 107°04.43'E, handpicked + soil sample, leg. Vermeulen, J.J. & Whitten, A.J., 28.09.1998 ex Vermeulen, nr. 6527, NHMUK 19991445/3; **20071122D** Hải Phòng Province, Hải Phòng City, Cát Bà Island, Cát Bà Nat. Park, between Cát Bà N.P., ranger st. and Quan Y, GPS not recorded, leg. Ohara, K, Okubo, K. & Otani, J. U., 22.11.2007., coll PGB (in ethanol, anatomically examined).

##### Diagnosis.

Shell medium-sized to very large, thick shelled, almost smooth or with very fine periostracal ribs; apertural lip well-developed; apertural fold long, more or less equally long in its total length, connected to the callus. Parietal wall with missing or short anterior lamella (always distant from the upper plica) and well-developed posterior lamella; palatal plicae depressed Z-shaped.

##### Measurements

(in mm). D = 16.6–17.1, H = 8.3–8.5 (n=2, MAA5); D = 17.4–19.9, H = 7.9–9.2 (n=2, MAA4); D = 16.1–19.8, H = 7–9.4 (n=2, MAA6); D = 23.1–23.4, H = 10.8–11 (n=2, 20081119A); D = 24.8–25.6, H = 11.7–13 (n=4, Vn11-174); D = 26–28.1, H = 12.8–13.1 (n=3, Vn11-175); D = 16.9–17.4, H = 8.2–8.4 (n=3, NHMUK 20110264, “*hirsuta*”); D = 16.5–17.3, H = 8.1–8.5 (n=3, NHMUK 1901.12.23.32–34, “*hirsuta*”) (see also Figure 44).

##### Differential diagnosis.

*Gudeodiscus dautzenbergi* and some populations of *Gudeodiscus villedaryi* resemble *Halongella schlumbergeri* in terms of general, but the inner lamellae are entirely different, namely, *Gudeodiscus dautzenbergi* and *Gudeodiscus villedaryi* have strong, well-developed anterior lamella with an anteriorly elongated lower “leg”, whereas most *Halongella schlumbergeri* shells lack the anterior lamella. It is possible to distinguish *Halongella schlumbergeri* from the other two species without breaking the shell, on the basis of the long apertural fold reaching the callus, which is short in *Gudeodiscus dautzenbergi* and *Gudeodiscus villedaryi*, and has an elevated “knob” part in some distance from the callus. See also under *Halongella fruhstorferi*.

##### Intraspecific diversity.

The species is very variable in terms of shell size and the formation of plicae and lamellae on the parietal wall.

##### Description of the genitalia.

Two specimens were examined anatomically each from one of two different samples. “Specimen1”: Hải Phòng Province, Cát Bà Island, behind cemetery of Gia Luận village, 20°50.092'N, 106°58.560'E, leg. Hemmen, Ch. & J., 10.04.2011. (with embryo in its uterus, Figures 26, 29H, 33B, F); “Specimen2”: Hải Phòng Province, Hải Phòng City, Cát Bà Island, Cát Bà Nat. Park, between Cát Bà N.P., ranger st. and Quan Y, GPS not recorded, leg. Ohara, K, Okubo, K. & Otani, J. U., 22.11.2007. (without embryo in its uterus, Figures 29A–B, 30G–I, 33A, C–E, G).

Penis relatively long, slimmer proximally and slightly thicker distally; inner wall with several (16–18) parallel running folds (Figures 29A–B); between the folds flat, T-shaped calcareous granules were found (both specimens had granules between the folds, see Figures 30G–I); epiphallus of similar length to the penis, proximally thicker than distally; its inner wall with six parallel folds; on the distal portion of the epiphallus the longitudinal folds have several perpendicular projections which overlap with those of the neighbouring fold (Figures 29H); penial caecum absent, the retractor muscle inserts on the penis-epiphallus transition. Vagina long, with a weak vaginal bulb; it is attached to the body wall with several filaments of connective tissue; inner wall of the vagina with 6–11 parallel, rather regular longitudinal folds; in “Specimen2” there are several, translucent calcareous granules on the folds; the granules have a widened base portion which attaches to the folds, and an apical part with some (1–10) pointed needles (Figure 33A, C–E, G); “Specimen1” had tiny rounded granules (“sand”) in the vagina lumen, not attached to the vagina wall (Figure 33F); stalk of the gametolytic sac with conspicuously thickened gametolytic sac is longer than the much slimmer diverticulum.

##### Radula.

See Table 6 and Figures 36D–F.

##### Distribution

(see Figures 40 and 44). The species has only been recorded in the Hạ Long Bay area (Hải Phòng and Quảng Ninh provinces).

##### Remarks.

Gude (1901b) figured specimens of all three “species”: *schlumbergeri*, *jovia* and *villedaryi* (later re-named *pilsbryana*). His observations were based on one specimen from each “species”. He wrote the following: “A comparison of these three species has shown that that they are very closely allied, and that there is no difference of diagnostic value between the armature. They differ, however, in external aspect sufficiently to rank as separate forms. *Plectopylis jovia* is the largest of the three, while *Plectopylis villedaryi* is the smallest, *Plectopylis schlumbergeri* being intermediate in size.” The additional differences mentioned by Gude, namely the strength of the callus, direction and small differences in the shape of the palatal and parietal lamellae and plicae are not sufficient to separate species. We had the possibility to observe and measure a number of shells collected in the Hạ Long Bay Area and provided with exact GPS data. The outer shell characters exhibit little variation other than in size. Therefore, we suggest synonymising the three species under one name.

The shell differences between *Plectopylis schlumbergeri* (and its synonyms) and *Plectopylis hirsuta*, namely the short or missing anterior lamella in *schlumbergeri* and the relatively “normal” anterior lamella of *hirsuta* are considered to be very minor. This trait shows clinal variation across shells assigned to *hirsuta* and *schlumbergeri* (and its synonyms). We therefore synonymize *Plectopylis hirsuta* with *Halongella schlumbergeri*.

#### Genus Sicradiscus

Taxon classificationAnimaliaStylommatophoraPlectopylidae

Páll-Gergely, 2013

##### Type species.

*Plectopylis schistoptychia* Möllendorff, 1886, by original designation.

##### Diagnosis.

See introduction.

##### Differential diagnosis.

*Gudeodiscus* differs from the keeled shell of *Sicradiscus* by the rounded body whorl. *Sicradiscus* species having rounded body whorl differ from *Gudeodiscus* by the combination of small shells with glossy base, a strong apertural fold connected to the callus, and short or divided palatal plicae. In contrast, *Gudeodiscus* species have usually large, mainly finely ribbed shells with weak apertural folds free from the callus (often absent) and long, depressed Z-shaped palatal plicae. See also under *Halongella* gen. n. and under the Discussion.

#### Sicradiscus mansuyi

Taxon classificationAnimaliaStylommatophoraPlectopylidae

(Gude, 1908)

Figures 2A , 9H , 11A–B , 27 , 31A , 36J–L

Plectopylis Mansuyi Gude 1908, Journal de Conchyliologie, 55: 347, 348–351., Figs 2a–e, Plate 7., Figs 1–3. [“Ha-Lang, Tonkin”]
Sicradiscus mansuyi , — Páll-Gergely & Hunyadi 2013, Archiv für Molluskenkunde, 142 (1): 50.

##### Types examined.

Tonkin, Ha-Lang, leg. Mansuy, NHMUK 1907.2.20.19 (syntype, Figure 2A).

##### Museum material examined.

Ha-Lang, coll. Mansuy, MNHN-IM-2012-2365/6; Ha-Lang, leg. Mansuy, MNHN-IM-2012-2384/7; HaLang, Tonkin, coll. Steenberg, ZMUC-GAS-1808/2.

##### New material examined.

**20081116C** Cao Bằng Province, Trùng Khánh District, Cảnh Tiên Commune, Pắc Rảo Village, ca 545 m, 22°48.941'N, 106°30.549'E, leg. Ohara, K., 16.11.2008., OK/66, PGB/5; **2011/81** Cao Bằng Province, Đèo Mã Phục (pass) 500 m towards Quảng Uyên, left side of the road, rock cavern, 610 m, 22°43.981'N, 106°20.333'E, leg. Hunyadi, A., 14.11.2011., HA/10; **2012/43** Cao Bằng Province, Pắc Rảo, Cảnh Tiên Commune cross, 300 m towards Trùng Khánh, right side of the road, 530 m, 22°49.385'N, 106°30.742'E, leg. Hunyadi, A., 28.05.2012., HA/9+5 jb; **2012/44** Cao Bằng Province, southern edge of Pắc Rảo, Trùng Khánh 3 km towards Quảng Uyên, left side of the road, 570 m, 22°48.961'N, 106°30.533'E, leg. Hunyadi, A., 28.05.2012., HA/226; **2012/47** Hà Giang Province, Hà Giang 105.5 km towards Ðồng Văn, Vân Chải Commune, left side of the road 4C, 23°09.084'N, 105°10.774'E, leg. Hunyadi, A., 31.05.2012., HA/4; **Vn11-141** Hà Giang Province, km 105.5 on road 4c, between Yên Minh and Đồng Văn (NE of Hà Giang town), 23°08.996'N, 105°10.332'E, leg. Hemmen, Ch., 21.03.2011., HE/6; **Vn11-143** Hà Giang Province, km 120 on road 4c, between Yên Minh and Đồng Văn (NE of Hà Giang town), no GPS-data, leg. Hemmen, Ch. & J., 22.03.2011., HE/3; **Vn10-60** Cao Bằng Province, ca. 6.5 km from Quảng Uyênto Mã Phục (left off road), 22°41.293'N, 106°23.422'E, leg. Hemmen, Ch. & J., 24.03.2010., HE/2; **20050327A** China, Guangxi (广西), Daxin Xian (大新), Xialei Zhen (下雷鎮), Detianpubu (德天瀑布) (Detian waterfalls), leg. Ohara, K. & Moriya Shigeki, 27.03.2005., PGB/1 (with glossy dorsal surface and without denticles posterior to the palatal plicae).

##### Diagnosis.

A very small species with reticulated dorsal and glossy ventral surface, elevated spire, elevated, sharp callus and well-developed apertural fold connected to the callus (Figure 9H). Parietal wall with two lamellae, the anterior one separated from both the lower and upper plicae; middle palatal plicae short, connected with a ridge and sometimes ornamented with small denticles posteriorly (Figures 11A–B).

##### Measurements

(in mm). D = 6.7–7, H = 3.4–3.9 (n=4, 20081116C).

##### Differential diagnosis.

All other similar congeners inhabit China. *Sicradiscus feheri* Páll-Gergely & Hunyadi, 2013 is larger, flatter with a wider umbilicus and a shinier dorsal surface, has a longer, horizontal palatal plicae without additional posterior denticles, and has a more elevated and longer apertural fold. *Sicradiscus transitus* Páll-Gergely & Hunyadi, 2013 has a lower spire and a wider umbilicus with slightly shouldered whorls, sometimes strong radial lines on the ventral surface, and a more elevated callus. Moreover, the anterior lamella of *Sicradiscus transitus* is in contact with both the upper and the lower plicae, which are free from the lamella in *Sicradiscus mansuyi*. *Sicradiscus invius* is flatter (has shallower umbilicus) with only the protoconch elevated from the dorsal surface; it has weaker dorsal sculpture resulting in a glossy surface (*mansuyi* is densely reticulated), and lacks the additional small denticles posterior to the palatal plicae, which are usually present in *Sicradiscus mansuyi*. *Gudeodiscus anceyi* is larger and has a ribbed shell with spiral lines on the whole shell. Species possessing a glossy ventral surface (*Gudeodiscus cyrtochilus*, *Gudeodiscus fischeri*) are also larger and have weaker or no apertural fold.

##### Intraspecific diversity.

Low; shell characters stable. The species is easily recognisable and can be separated from other plectopylid species without difficulty.

##### Description of the genitalia.

Two specimens were anatomically examined (Cao Bằng Province, southern edge of Pắc Rảo, Trùng Khánh 3 km towards Quảng Uyên, left side of the road, 570 m, 22°48.961'N, 106°30.533'E, leg. Hunyadi, A., 28.05.2012. (Figures 27, 31A).

Penis with a shorter, slimmer proximal section and a thinner, somewhat longer distal portion; internally with parallel folds which are more elevated in the thinner distal portion, forming pocket-like structures (similar to that of *Sicradiscus transitus*, see Páll-Gergely and Asami 2014); these “pockets” did not contain granules; epiphallus approximately as long as the penis but much slimmer; internally penis and epiphallus wall with longitudinal, parallel folds; retractor muscle short, inserts on the penis-epiphallus transition; penial caecum absent. Vagina long, with distal vaginal bulb; vaginal bulb and other parts of the vagina with approximately 8, more or less parallel, serrulate folds (Figure 31A); vas deferens long, thicker distally and more slender proximally; gametolytic sac and diverticulum are of equal length, in parallel.

##### Radula.

See Table 6 and Figures 36J–L.

##### Distribution.

This species was described from Hạ Lang (eastern part of Cao Bằng Province, see Figure 39). We have seen newly collected material from northern Hà Giang and Cao Bằng provinces. The first occurrence of the species from China is reported. This locality is situated very close to the Vietnamese border.

## Concluding remarks

### Identification and species recognition

For this revision of the Vietnamese Plectopylidae, we examined the type specimens of all known taxa, 197 newly collected specimens with detailed locality data and 631 historical lots deposited in a variety of public collections. Altogether we examined more than 7000 shells (see Table 12). We found specimens of most species in European museum collections, probably because of intensive shell exchanges at the beginning of the 20^th^ Century. The present scale of specimen examination allowed us to understand species boundaries in the Vietnamese Plectopylidae better than the preceding studies.

**Table 12.** Numbers of specimens examined in each taxon.

taxon	new samples	museum samples	all individuals
*anceyi*	16	49	1079
*cyrtochilus*	8	2	71
*dautzenbergi*	4	38	151
*emigrans emigrans*	0	2	3
*emigrans quadrilamellatus*	4	23	68
*fischeri*	15	14	169
*francoisi*	6	31	142
*fruhstorferi*	1	5	37
*giardi giardi*	21	74	557
*hemmeni* sp. n.	5	0	38
*mansuyi*	8	3	351
*messageri*	0	102	551
*messageri raheemi*	23	0	152
typical *phlyarius*	34	44	555
*phlyarius gouldingi*/*fallax*	2	139	1138
*schlumbergeri*	28	78	1682
*suprafilaris*	7	2	102
*verecundus*	0	6	25
*villedaryi*	15	19	171
**SUM**	**197**	**631**	**7042**

Although the plicae and lamellae (especially on the parietal wall) are common characteristics of the family and useful for identification of some species, their value in species recognition has been somewhat overestimated. This appears to have led to descriptions of several species that differ only slightly in palatal and parietal plication. Our recognition of distinct species is primarily based on general shell and aperture shape, and secondarily on the morphology of plicae and lamellae.

### Key characters for identification (see also identification key)

As a summary, below we present the most important shell characters for identification of each species from others within the Vietnamese Plectopylidae. In the case of *Gudeodiscus emigrans emigrans* and *Gudeodiscus infralevis*, however, available shell specimens were insufficient to provide help for “routine” identification.

*anceyi* (Figs 2B, 9G, 11C–F): small size, spiral lines on the ventral surface

*cyrtochilus* (Figs 2F, 15E–G): small size, thin peristome and callus, no apertural fold

*dautzenbergi* (Figs 8E–F, 9K–L, 14A–G): shell shape, characteristic aperture and apertural fold, free lower parietal plica absent

*emigrans quadrilamellatus* (Figs 6F, 13C–D): flat shell, spiral lines

*fischeri* (Figs 2E, 3A–C, 9P–Q, 15H–N): nautiliform shape (body whorl is conspicuously wider than the previous), blunt callus and apertural fold

*francoisi* (Figs 7A–C, 13E–K): slowly expanding whorls, characteristic aperture

*fruhstorferi* (Figs 7D, 9O, 14O–R): few whorls, aperture with thin rim and apertural fold

*giardi* (Figs 7E–F, 8A, 9I, 13L–U): shell shape, narrow umbilicus, thick peristome

*hemmeni* (Figs 2C–D, 9F, 11G–J): small size, minute apertural fold, characteristic aperture shape

*mansuyi* (Figs 2A, 9H, 11A–B): small size, glossy ventral surface

*messageri messageri* (Figs 5F–G, 9E, 12N–Q): slightly elevated spire, callus not angled in the middle, apertural fold always missing

**Figure 10.**
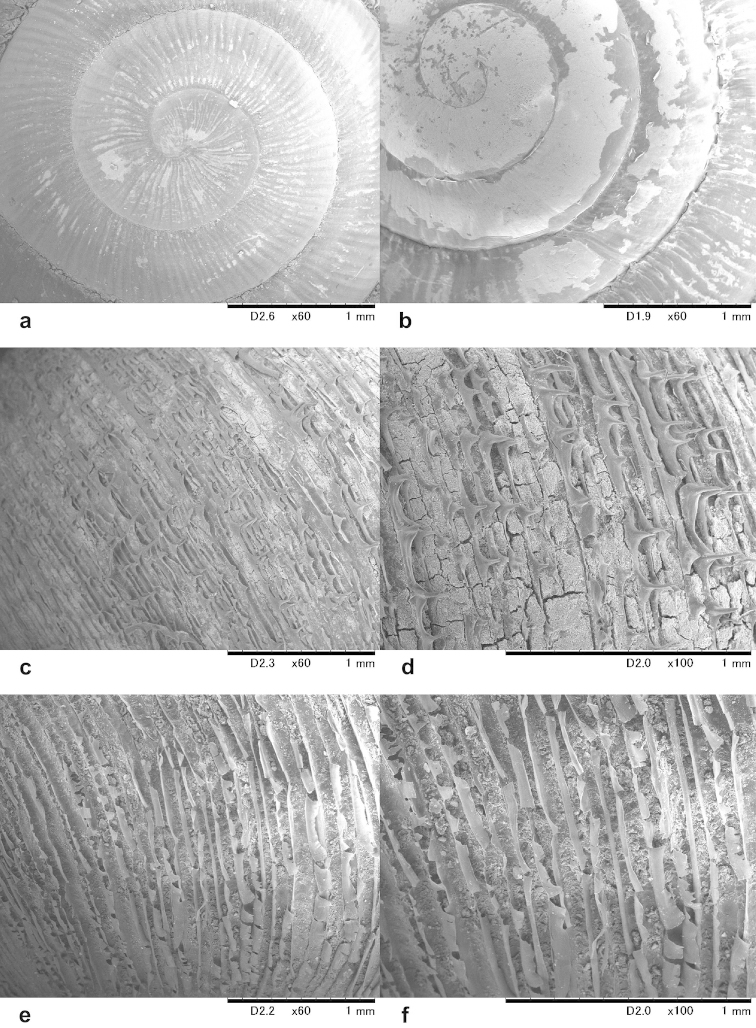
SEM images of *Gudeodiscus* shells. **A** protoconch of Gudeodiscus (Gudeodiscus) messageri raheemi Páll-Gergely & Hunyadi, ssp. n., Vn12-104, coll. HE **B** protoconch of Gudeodiscus (Gudeodiscus) villedaryi (Ancey, 1888), Vn11-163, coll. HE **C–D** sculpture of Gudeodiscus (Gudeodiscus) phlyarius (Mabille, 1887), Vn10-56, coll. HE **E–F** sculpture of Gudeodiscus (Gudeodiscus) phlyarius (Mabille, 1887) (typical *fallax* specimen), Vn11-187, coll HE. Images: B. Páll-Gergely.

*messageri raheemi* (Figs 5D, 5E, 10A, 12R–V): body whorl less shouldered than that of the nominotypical subspecies, but plicae have to be observed for correct identification

*phlyarius* (typical *phlyarius*; Figs 4A–B, 10C–D, 12A–M): characteristic rounded aperture, apertural fold always present

**Figure 11.**
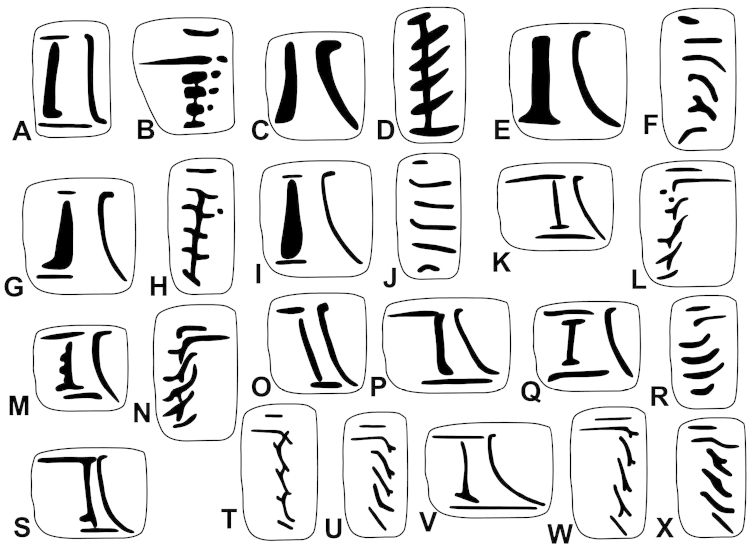
Parietal (**A, C, E, G, I, K, M, O–Q, S, V**) and palatal (**B, D, F, H, J, L, N, R, T–U, W–X**) plicae and lamellae of *Sicradiscus* and *Gudeodiscus* species. **A–B** *Sicradiscus mansuyi* (Gude, 1908), 20081116C (two different specimens) **C–F** Gudeodiscus (Gudeodiscus) anceyi (Gude, 1901) **C–D** figures in Gude (1901a) **E** MNHN-IM-2012-2263, **F** GS22 **G–J** Gudeodiscus (Gudeodiscus?) hemmeni Páll-Gergely & Hunyadi, sp. n. **G–H** 2012/62, spec.1 **I–J** 2012/62, spec.2 **K–P** Gudeodiscus (Gudeodiscus) phlyarius (Mabille, 1887) (typical *gouldingi* and *anterides* shells) **K–L** *Plectopylis gouldingi* (after Gude 1909) **M–N** *Plectopylis anterides* (after Gude 1909) **O–P** MNHN-IM-2012-2153 **Q–X** Gudeodiscus (Gudeodiscus) phlyarius (typical *fallax* and fallax var. major shells) **Q–R** *Plectopylis fallax* (after Gude 1909) **S** MNHN-IM-2012-2157 **T–U** MNHN-IM-2012-2132 (2 different specimens) **V–W** MNHN-IM-2012-2155/6 (“var. *major*”, two different specimens), **X** Vn11-187. Inner view: **D, L, N, R**; Outer view: **B, F, H, J, T, U, W, X.**

*phlyarius* (typical *fallax*; Figs 5A, 5C, 10E–F, 11Q–X): flat shell, callus angled in the middle, shell large, nautiliform (body whorl conspicuously wider than *messageri raheemi*)

*phlyarius* (typical “*anterides*” and “*gouldingi*”; Figs 4–F, 9D, 11K–P): small, flat shell, callus angled in the middle

*phlyarius* (typical “*verecunda*”; Figs 5B): elevated spire, strong apertural fold

*schlumbergeri* (Figs 6A–D, 9M–N, 14H–N): robust shell, callus and aperture shape (including the formation of the fold)

*suprafilaris* (Figs 9A–B, 9R, 14S–Y): narrow umbilicus, solid aperture, sculpture changing suddenly on the body whorl

*villedaryi* (Figs 8B–D, 9J, 10B, 13V–Y): aperture shaped characteristically, unique keel around the umbilicus in some populations, free lower parietal plica present

**Figure 12.**
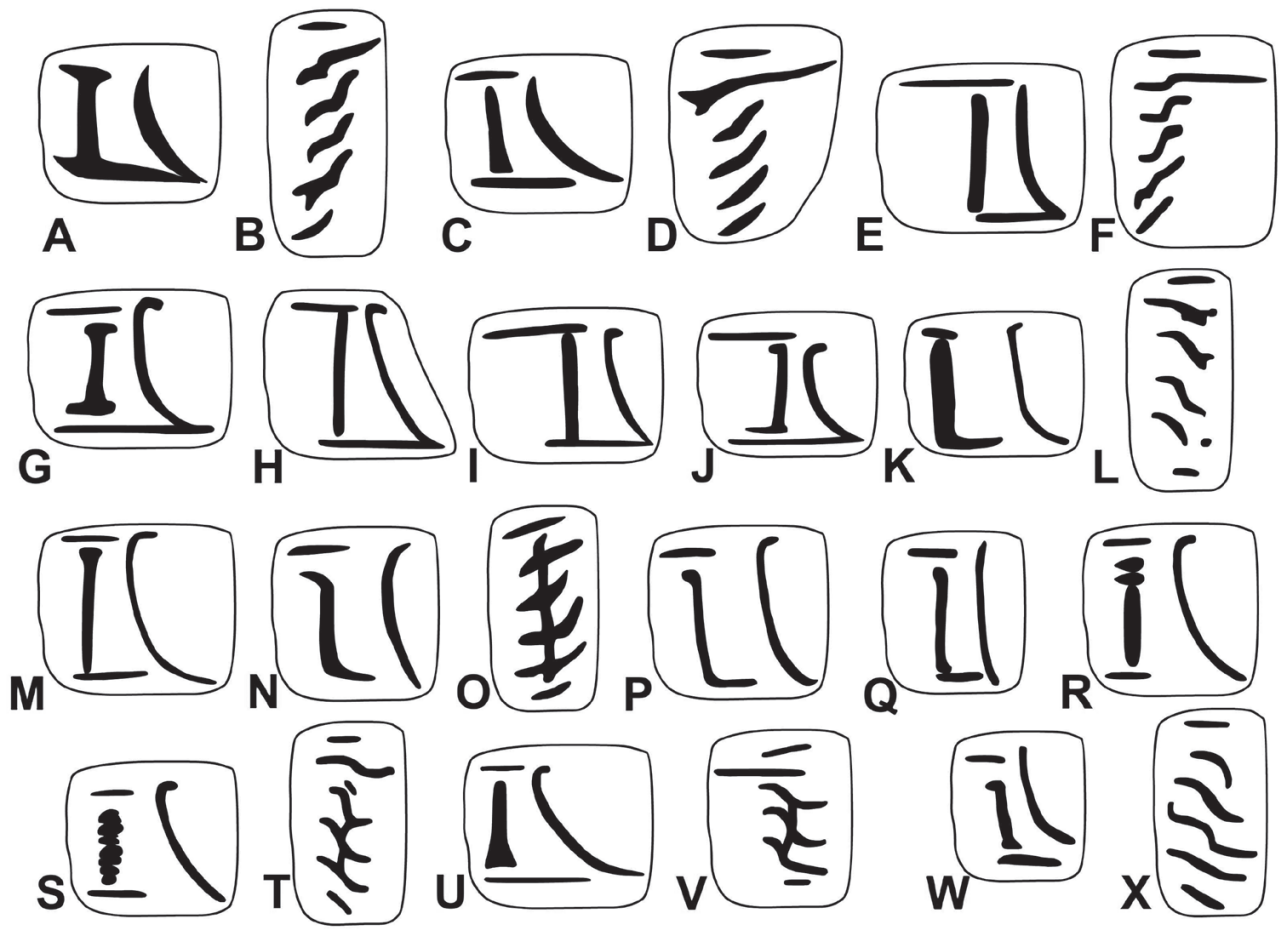
Parietal (**A, C, E, G–K, M, N, P–S, U, W**) and palatal (**B, D, F, L, P, T, V, X**) plicae and lamellae of *Gudeodiscus* species. **A–M** Gudeodiscus (Gudeodiscus) phlyarius (Mabille, 1887) **A–B** *Plectopylis phlyaria* (after Gude 1901c) **C–D** *Plectopylis moellendorffi* (after Gude 1901c) **E–F** Vn10-49, **G** Vn09-24 **H** Vn10-56, **I** Vn9-16, spec.1 **J** Vn9-16, spec.2 **K–M** *Plectopylis verecunda*, MNHN 2012-2177 (3 different specimens) **N–Q** Gudeodiscus (Gudeodiscus) messageri messageri (Gude, 1909) **N–O** *Plectopylis messageri* (after Gude 1909) **P** MNHN-IM-2012-2162, **Q** MNHN-IM-2012-2165 **R–V** Gudeodiscus (Gudeodiscus) messageri raheemi ssp. n, **R** 20071116C, spec.1. **S–T** 20071116C, spec.2. **U–V** 20080509C **W–X** Vn10-104B. Inner views: **B, D, F, O**; Outer views: **L, T, V, X.**

**Figure 13.**
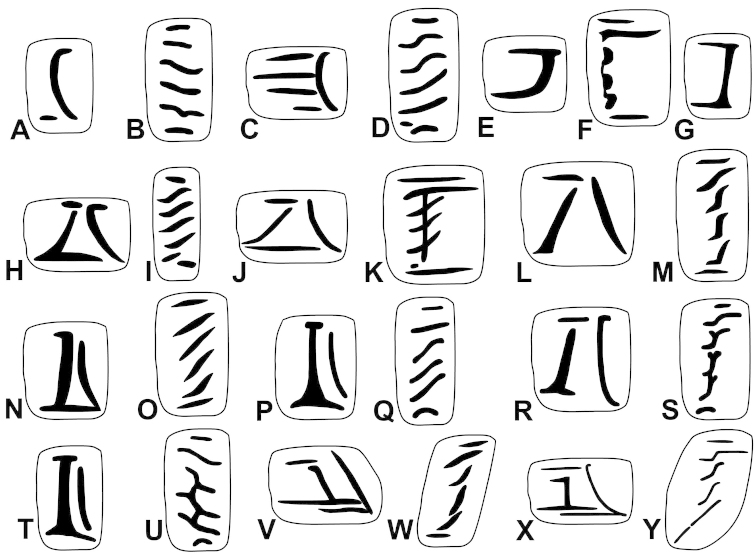
Parietal (**A, C, E, F, H, J, L, N, P, R, T, V**, X) and palatal (**B, D, G, I, K, M, O, Q, S, U, W, Y**) plicae and lamellae of *Gudeodiscus* species. **A–B** Gudeodiscus (Veludiscus) emigrans emigrans (Möllendorff, 1901), holotype (after Páll-Gergely and Hunyadi 2013) **C–D** Gudeodiscus (Veludiscus) emigrans quadrilamellatus Páll-Gergely, 2013 (after Páll-Gergely and Hunyadi 2013) **E–K** Gudeodiscus (Gudeodiscus?) francoisi (Fischer, 1898) **E–F** holotype of *Plectopylis lepida* Gude, 1901 (after Gude 1901b), **G** MNHN-IM-2012-2311 **H–I** holotype of *Plectopylis bavayi* Gude, 1901 (after Gude 1901a) **J–K** *Plectopylis francoisi* (after Gude 1899b) **L–U** Gudeodiscus (Gudeodiscus) giardi giardi (Fischer, 1898) **L** *Plectopylis giardi* (after Gude 1899a) **M** *Plectopylis giardi* (after Gude 1899b) **N–O** *Plectopylis congesta* Gude, 1899 (after Gude 1899a); **P–Q** Vn10-69 **R–S** Vn10-59 **T–U** 2011/85 **V–Y** Gudeodiscus (Gudeodiscus) villedaryi (Ancey, 1888) **V–W** holotype of Plectopylis (Endoplon) choanomphala Möllendorff, 1901 (after Gude 1901c) **X–Y** Vn10-47A. Inner views: **D, F, I, K, M, O, Q, S, W, Y**; Outer views: **B, U.**

**Figure 14.**
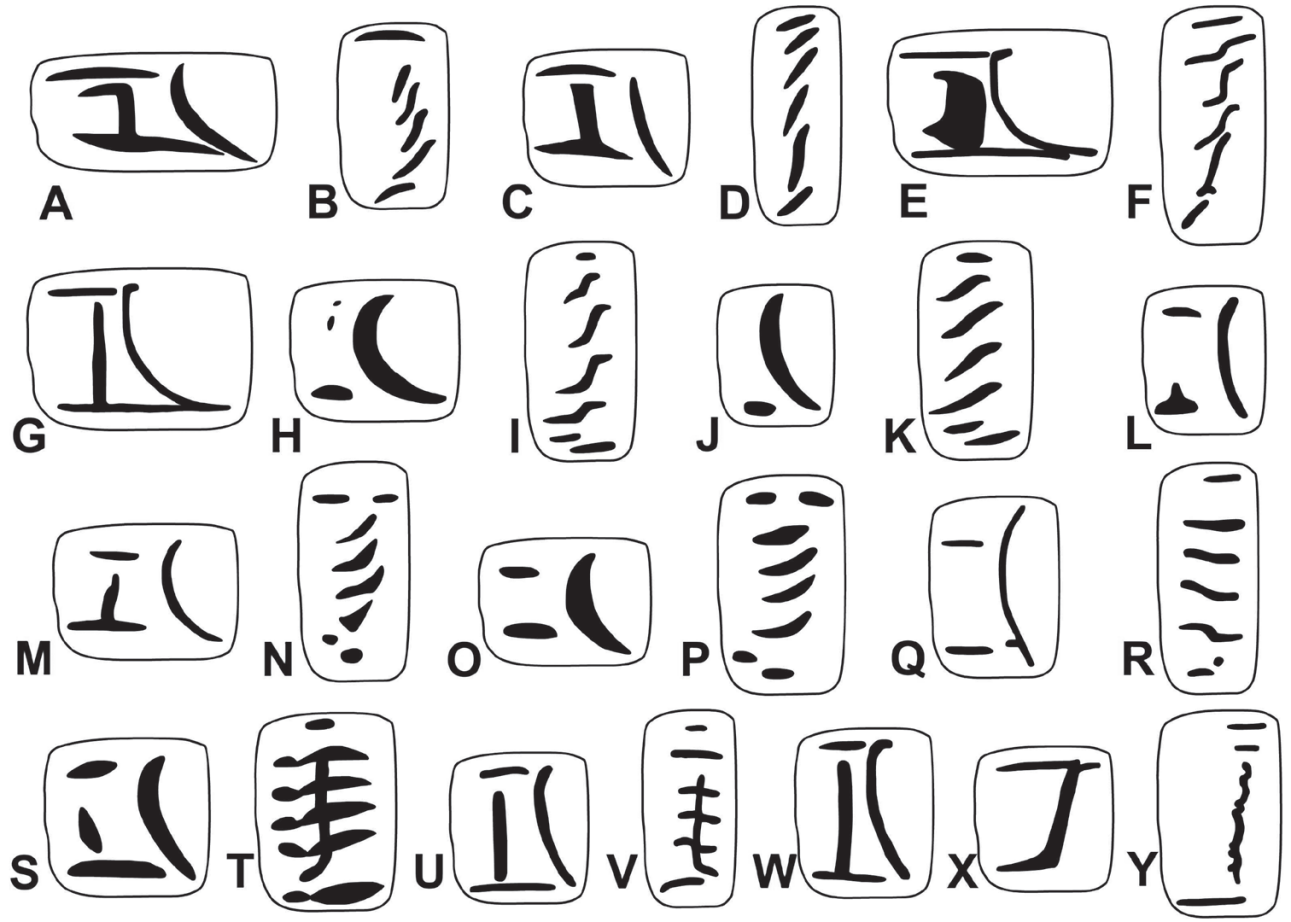
Parietal (**A, C, E, G, H, J, L, M, O, Q, S, U, W, X**) and palatal (**B, D, F, I, K, N, P, R, T, V, Y**) plicae and lamellae of *Gudeodiscus* and *Halongella* gen. n. species. **A–G** Gudeodiscus (Gudeodiscus) dautzenbergi (Gude, 1901) **A–B** *Plectopylis dautzenbergi* (after Gude 1901a) **C–D** *Plectopylis persimilis* Gude, 1901 (after Gude 1901a) **E–F** Vn10-44, **G** Vn10-44 **H–N** *Halongella schlumbergeri* (Morlet, 1886) **H–I** *Plectopylis jovia* (after Gude 1901b) **J–K** *Plectopylis schlumbergeri* (after Gude 1901b) **L** MNHN-IM-2012-2481 **M–N** holotype of *Plectopylis hirsuta* Möllendorff, 1901 (after Gude 1901c) **O–R** *Halongella fruhstorferi* (Möllendorff, 1901) **O–P** after Gude (1901c) **Q–R** Vn11-171 **S–Y** Gudeodiscus (Gudeodiscus?) suprafilaris (Gude, 1908) **S–T** after Gude (1908) **U–V** 2011/81, spec.1. **W** 2011/81, spec.2. **X–Y** Vn10-125. Inner views: **B, D, F, I, K, N, P, T**; Outer views: **R, V, Y.**

**Figure 15.**
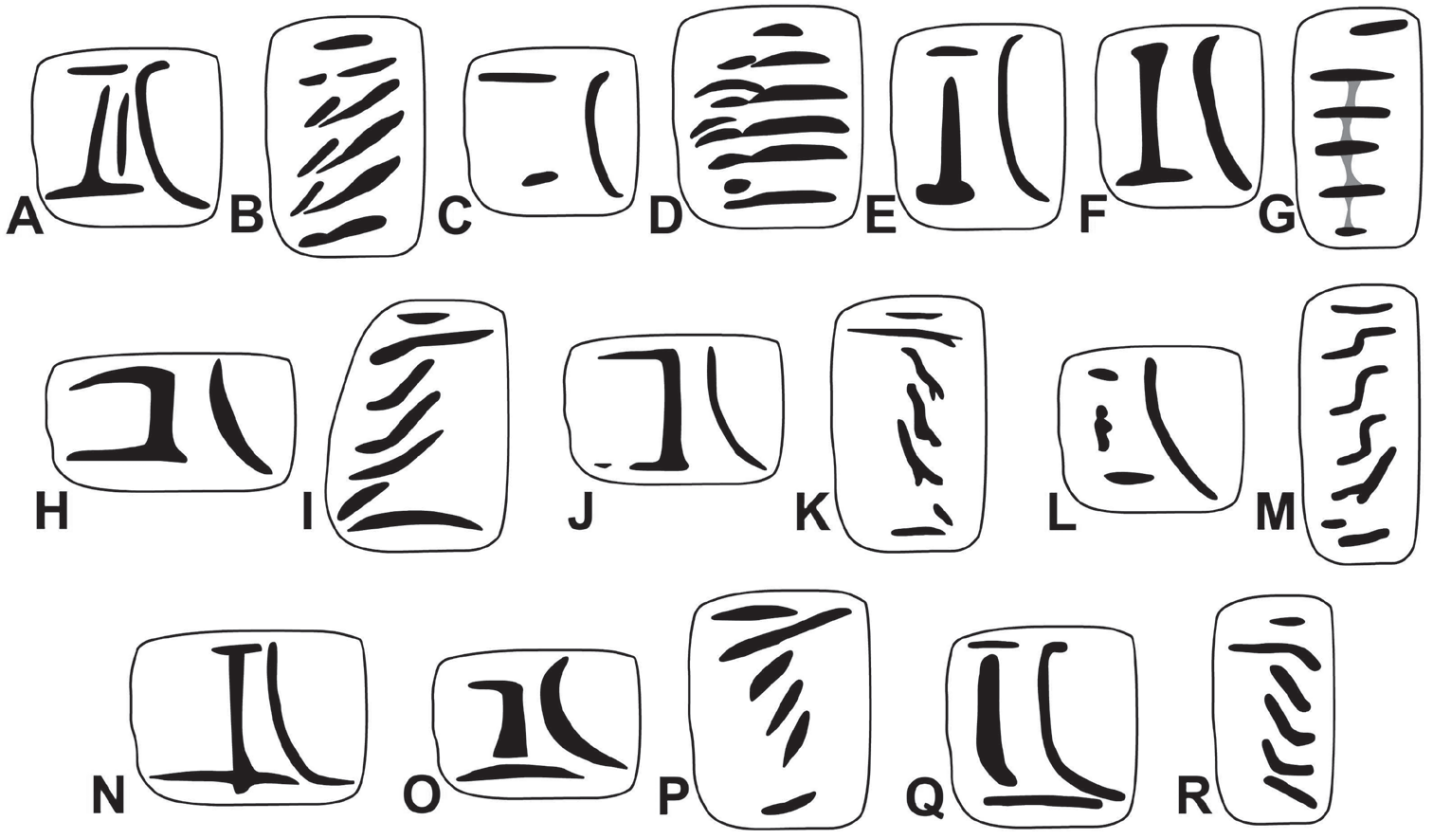
Parietal (**A, C, E, F, H, J, L, N, O, Q**) and palatal (**B, D, G, I, K, M, P, R**) plicae and lamellae of *Gudeodiscus* species. **A–D** Gudeodiscus (Gudeodiscus) infralevis (Gude, 1908) **A–B** holotype of *Plectopylis infralevis* (after Gude 1908) **C–D** Holotype of *Plectopylis soror* (after Gude 1908) **E–G** Gudeodiscus (Gudeodiscus?) cyrtochilus (Gude, 1909) **E–F** MNHN-IM-2012-2251 (two different specimens) **G** 2012/47 **H–N** Gudeodiscus (Gudeodiscus) fischeri (Gude, 1901) **H–I** *Plectopylis fischeri* (after Gude 1901a) **J–K** 20090515C **L–M** Vn10-120 **N** MNHN-IM-2012-2241 **O–R** Gudeodiscus (Gudeodiscus) fischeri (Gude, 1901) (identical with the holotype of *tenuis*) **O–P** after Gude (1901a) **Q–R** Vn10-28A (two different specimens). Inner views: **B, D, I, M, P**; Outer views: **G, K, R.**

**Figure 16.**
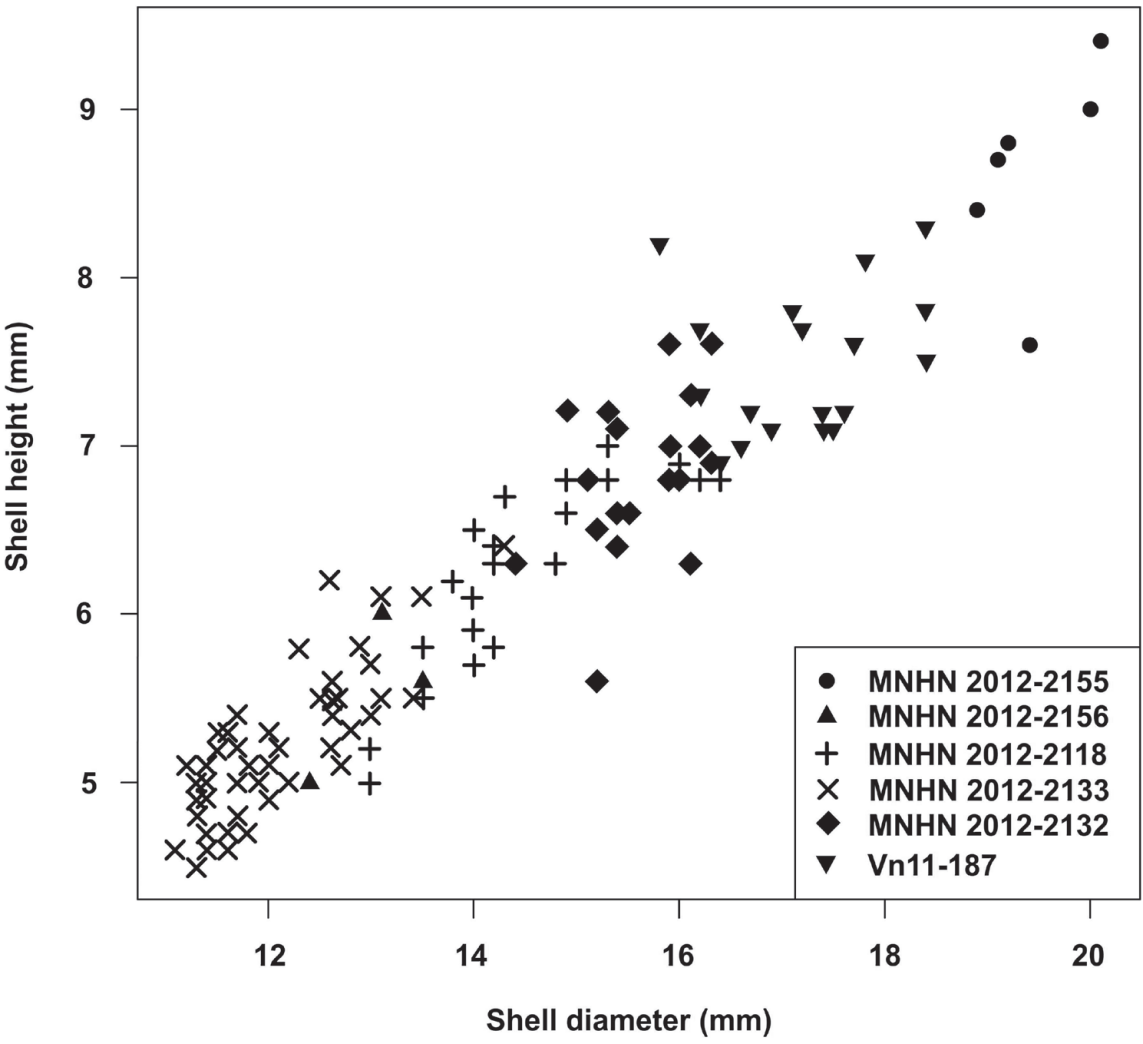
Plot of shell height against shell width (diameter) for 122 adults of Plectopylis cf. anterides/*gouldingi* (MNHN 2012-2133, MNHN 2012-2156, partly MNHN 2012-2218), Plectopylis cf. fallax (Vn11-187, MNHN 2012-2132, partly MNHN 2012-2218) and Plectopylis cf. fallax var. major (MNHN 2012-2155) from northern Vietnam. Samples MNHN 2012-2155 and MNHN 2012-2156 originally belonged to the same sample.

**Figure 17.**
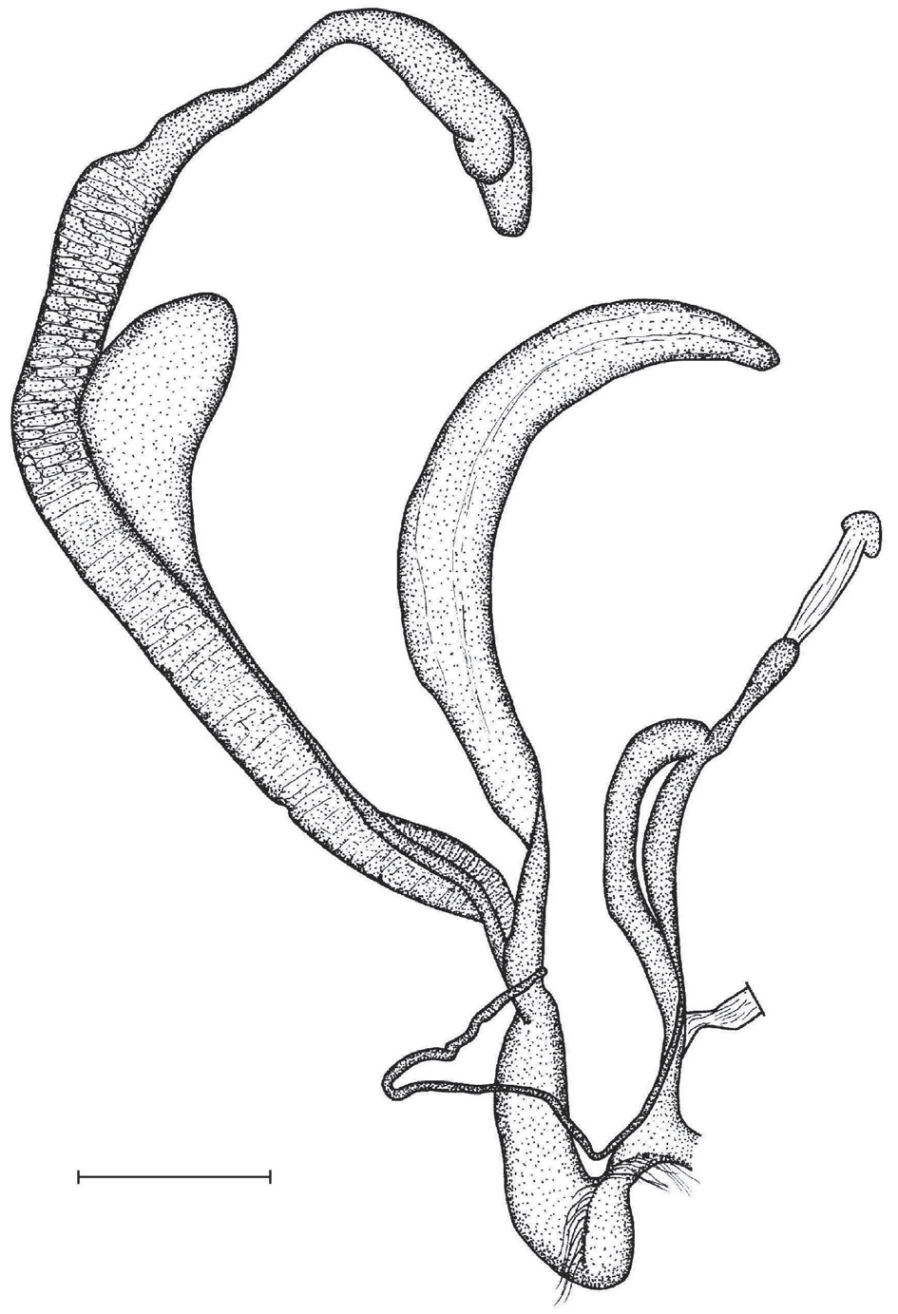
Reproductive anatomy of Gudeodiscus (Gudeodiscus) fischeri (Gude, 1901). Locality information: Tuyên Quang Province, near Ton Hông, road #185 from Tuyên Quang to Vĩnh Lộc (formerly Chiêm Hóa) (NE of Tuyên Quang), leg. Hemmen, Ch. & J., 19.03.2011. Scale represents 5 mm.

**Figure 18.**
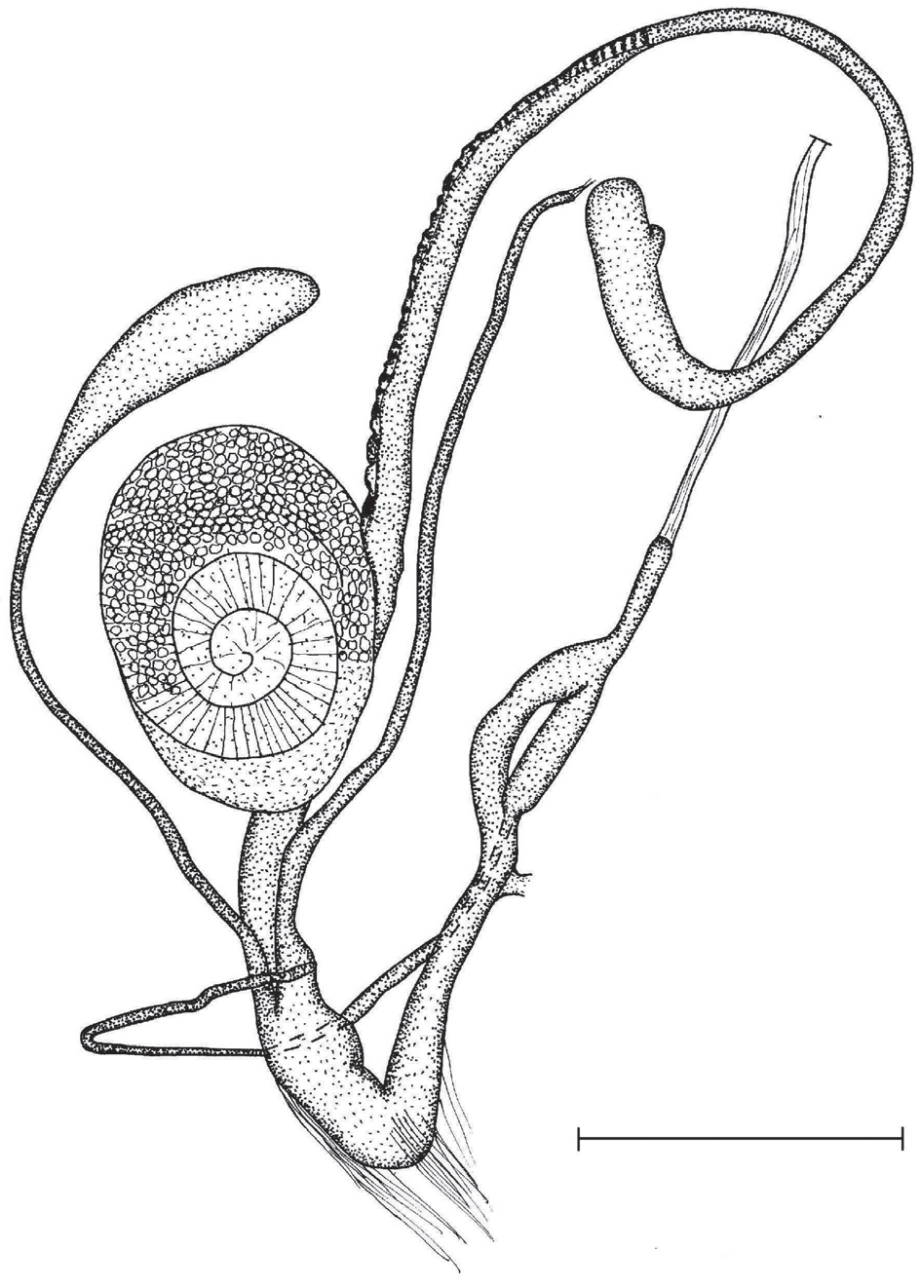
Reproductive anatomy of Gudeodiscus (Gudeodiscus) fischeri (Gude, 1901) (typical *tenuis* specimen). Locality information: Bắc Kạn Province, Ba Bể Nat. Park, Hang Thẳm Kit 2 km from the look-out tower, 335 m, 22°24.686'N, 105°37.710'E, leg. Hunyadi, A., 19.11.2011. Scale represents 5 mm.

**Figure 19.**
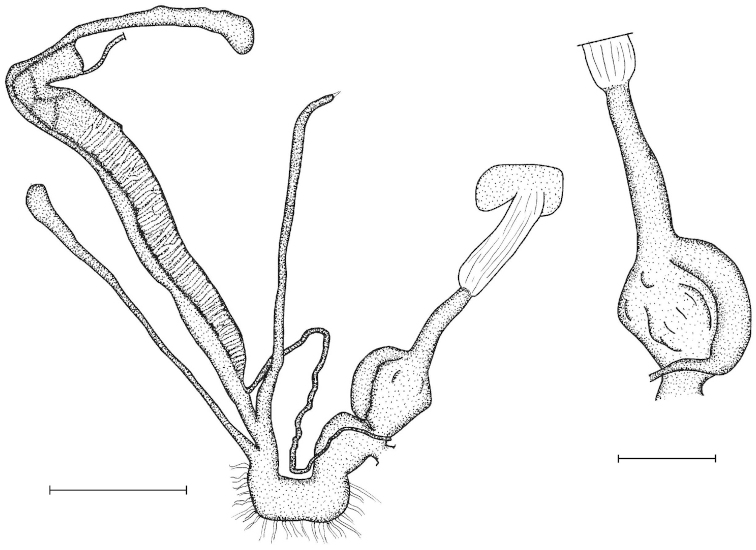
Reproductive anatomy of Gudeodiscus (Gudeodiscus) giardi giardi (Fischer, 1898). Locality information: Cao Bằng Province, Quảng Uyên N, 206–207 cross, 430 m, 22°42.737'N, 106°27.223'E, leg. Hunyadi, A., 16.11.2011. Scales represents 5 mm (left) and 2 mm (right).

**Figure 20.**
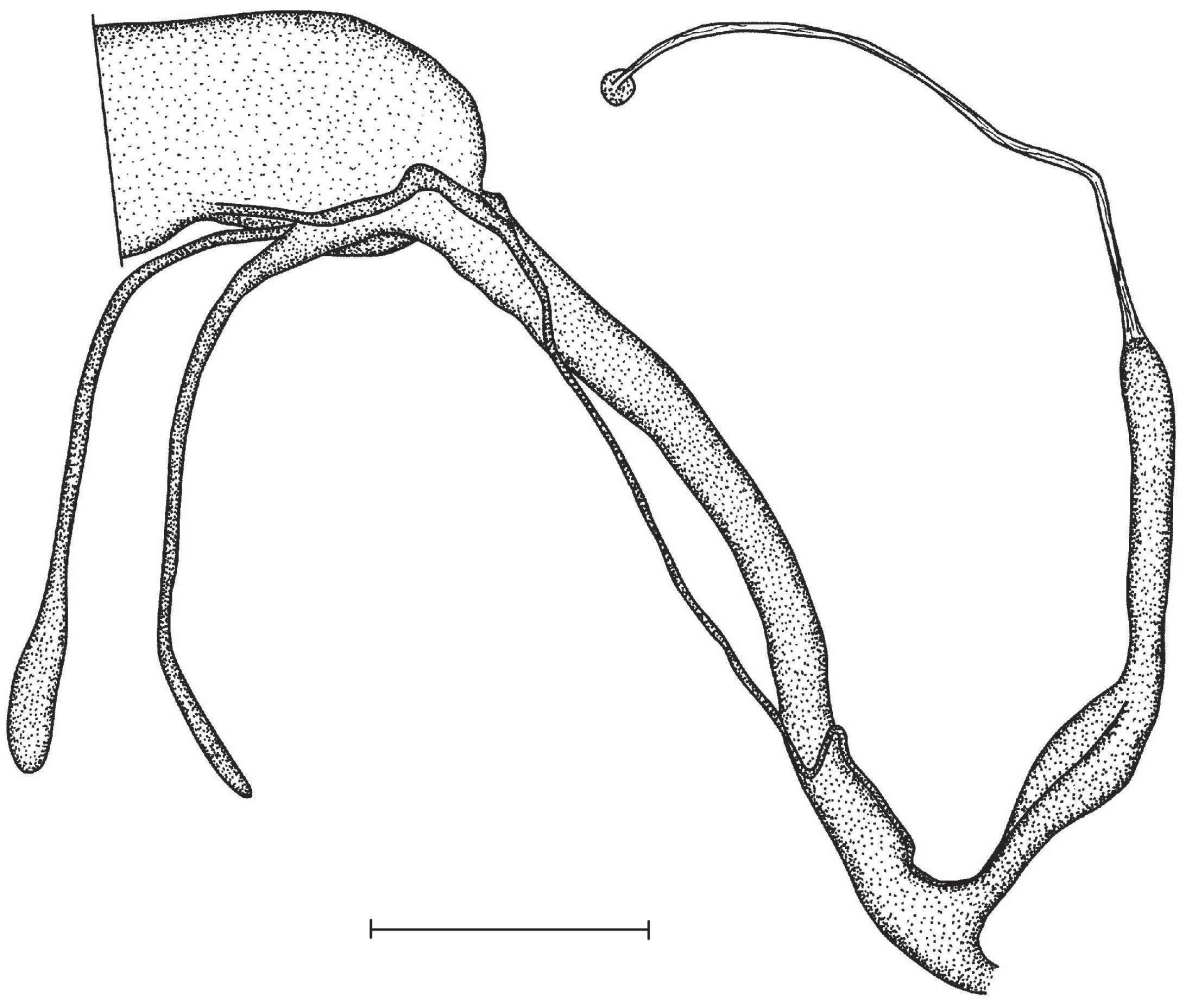
Reproductive anatomy of Gudeodiscus (Gudeodiscus) messageri raheemi Páll-Gergely & Hunyadi, ssp. n. Locality information: Hòa Bình Province, ca. km 156 old road Hà Nội to Sơn La (right side off road), 20°46.000'N, 104°53.885'E, leg. Hemmen, Ch. & J., 15.10.2010. Scale represents 5 mm.

**Figure 21.**
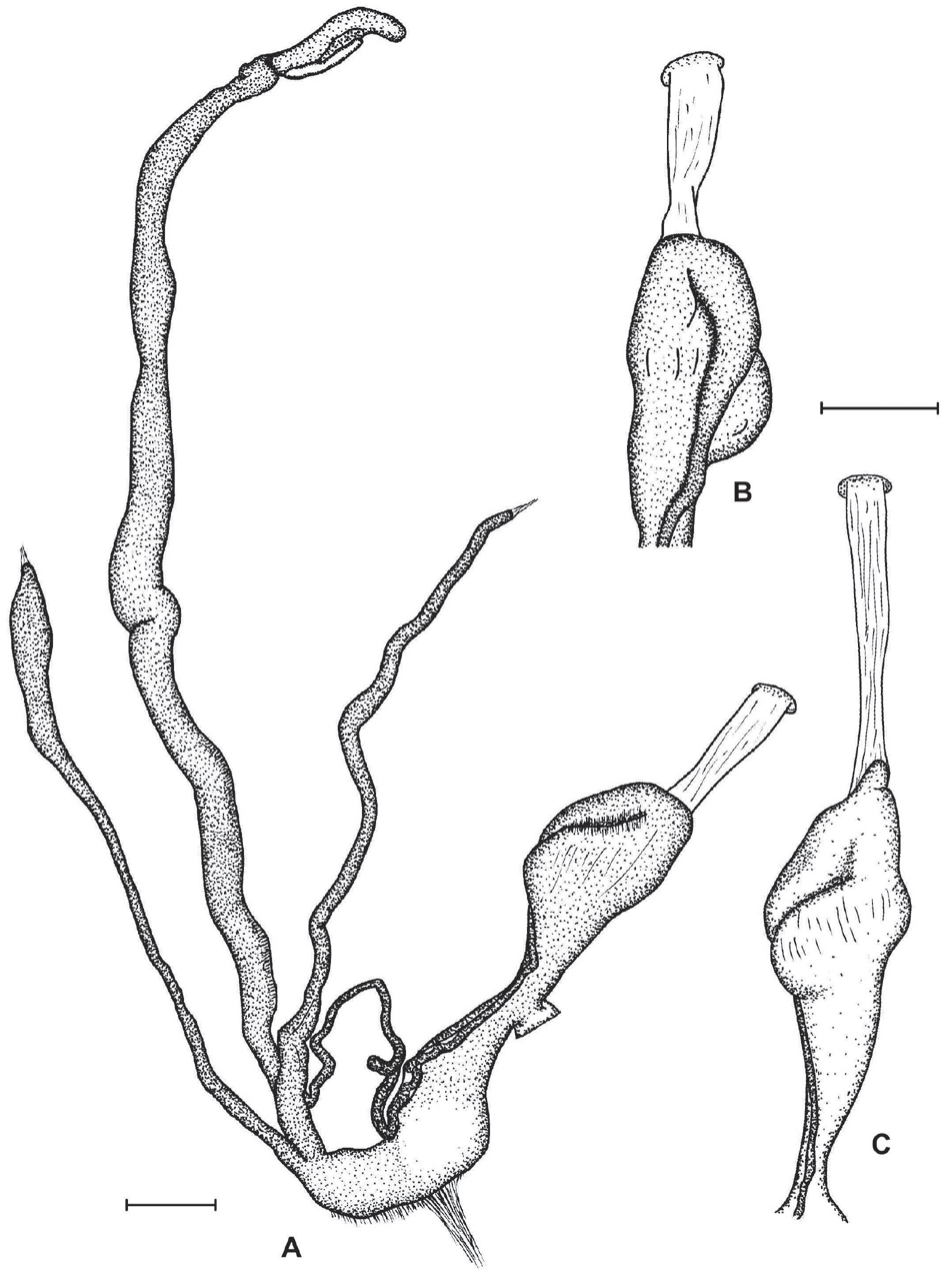
Reproductive anatomy of Gudeodiscus (Gudeodiscus) phlyarius (Mabille, 1887) (typical *fallax*). Locality information: Lào Cai Province, ca. 3 km SW of Nhà Văn Hóa, 22°25.513'N, 104°12.194'E, leg. Hemmen, Ch. & J., 04.10.2011. **A–B** “Specimen1” **C** “Specimen2”. Scale represents 1 mm.

**Figure 22.**
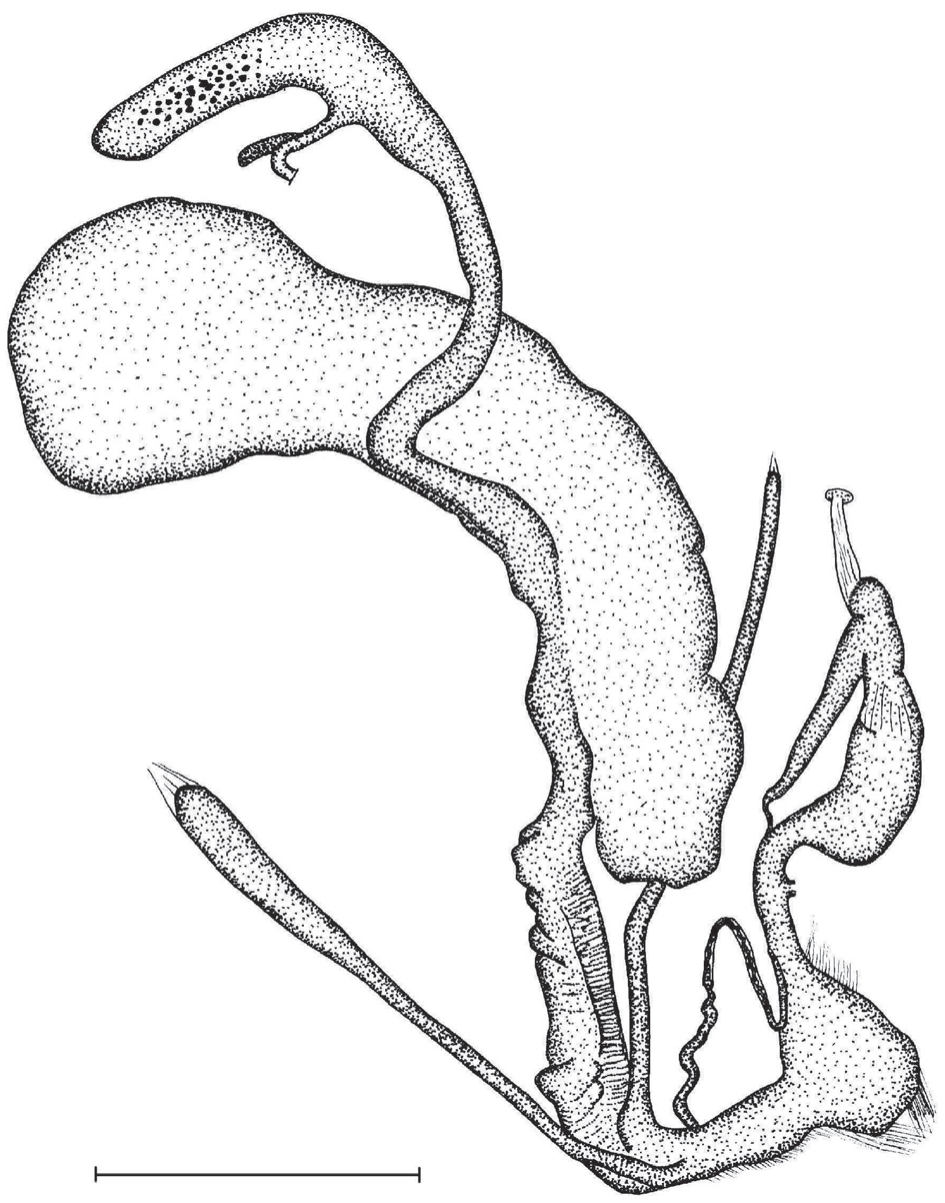
Reproductive anatomy of Gudeodiscus (Gudeodiscus) phlyarius (Mabille, 1887). Locality information: Lạng Sơn Province, ca. 10.6 km from Bình Gia to Lạng Sơn on road 1B, 21°53.639'N, 106°25.895'E, leg. Hemmen, Ch. & J., 01.04.2011. Scale represents 5 mm.

**Figure 23.**
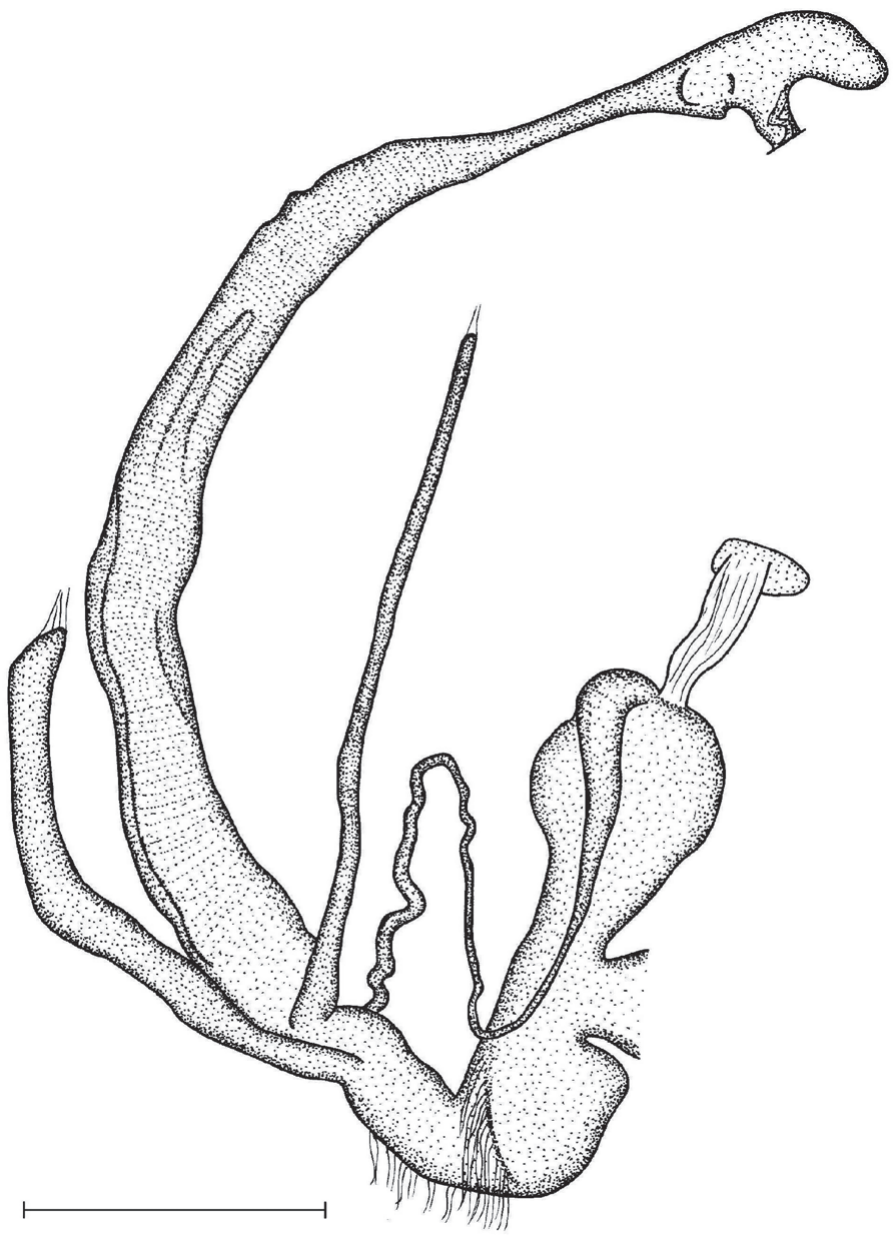
Reproductive anatomy of Gudeodiscus (Gudeodiscus) villedaryi (Ancey, 1888). Locality information: Thái Nguyên Province, Ðình Cả NE 4 km, Phượng Hoàng cave, around the entrance of the cave, 365 m, 21°46.782'N, 106°07.189'E, leg. Hunyadi, A., 13.11.2011. Scale represents 5 mm.

**Figure 24.**
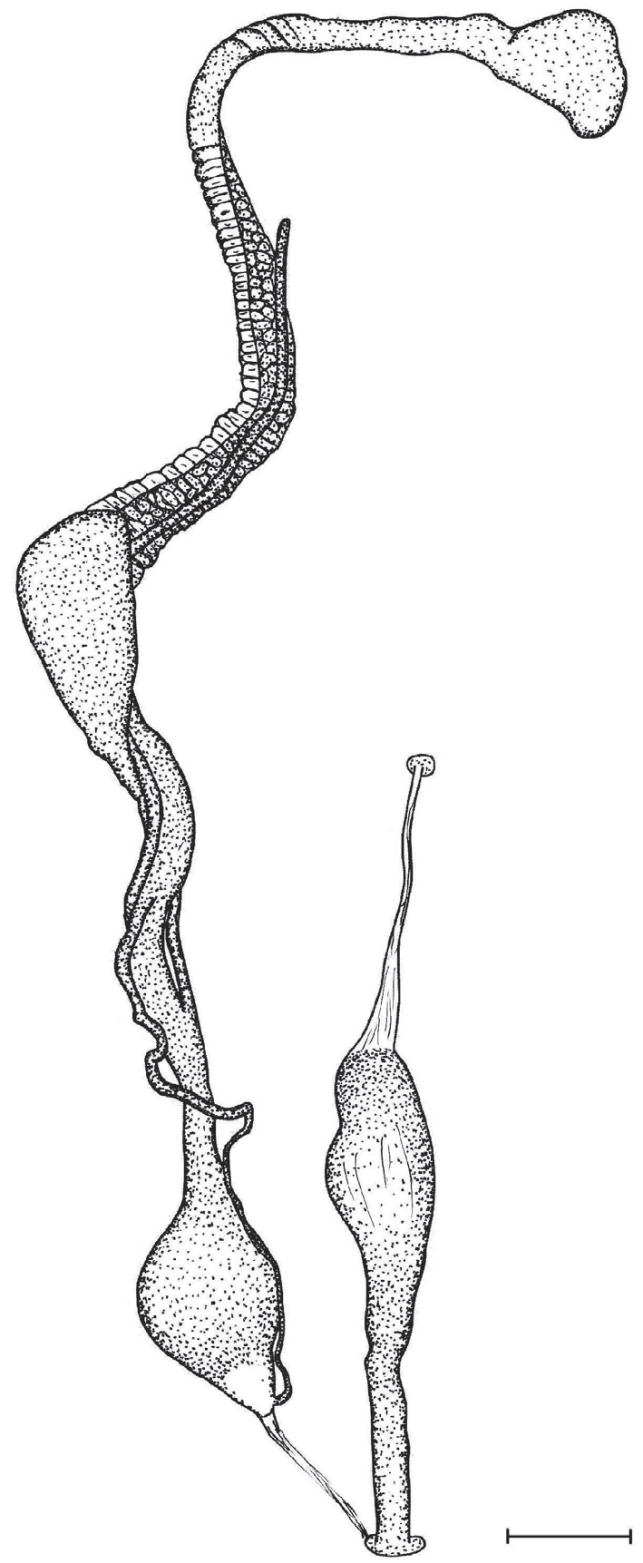
Reproductive anatomy of Gudeodiscus (Gudeodiscus) villedaryi (Ancey, 1888), abnormal specimen. Locality information: Thái Nguyên Province, Võ Nhai District, Phú Thượng Commune, Phượng Hoàng Cave, Mỏ Gà Vill., ca 150 m, 21°46.836'N, 106°07.107'E, leg. Ohara, K., 20.05.2009. Scale represents 2 mm.

**Figure 25.**
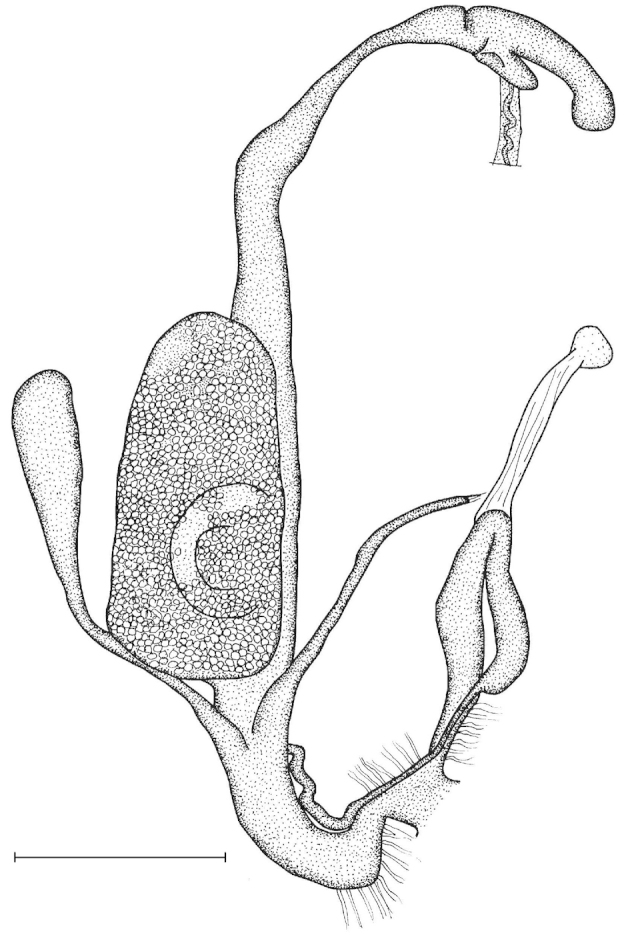
Reproductive anatomy of *Halongella fruhstorferi* (Möllendorff, 1901). Locality information: Quảng Ninh Province, Vân Đồn Island (NE Cẩm Phả), Cái Rồng village, 21°3.560'N, 107°25.551'E, leg. Hemmen, Ch. & J., 14.08.2011. Scale represents 5 mm.

**Figure 26.**
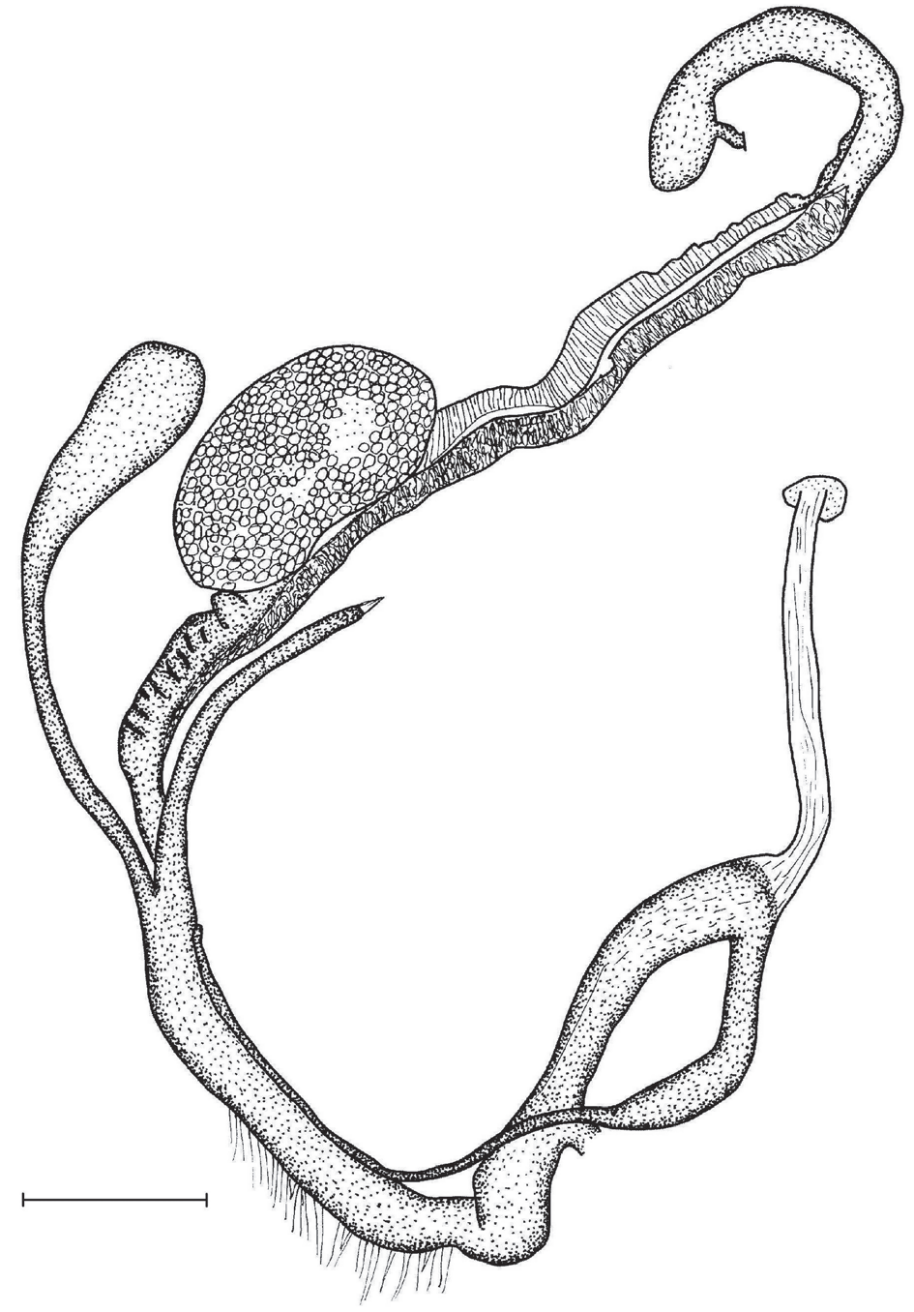
Reproductive anatomy of *Halongella schlumbergeri* (Morlet, 1886), “Specimen1”. Locality information: Hải Phòng Province, Cát Bà Island, behind cemetery of Gia Luận village, 20°50.092'N, 106°58.560'E, leg. Hemmen, Ch. & J., 10.04.2011. Scale represents 5 mm.

**Figure 27.**
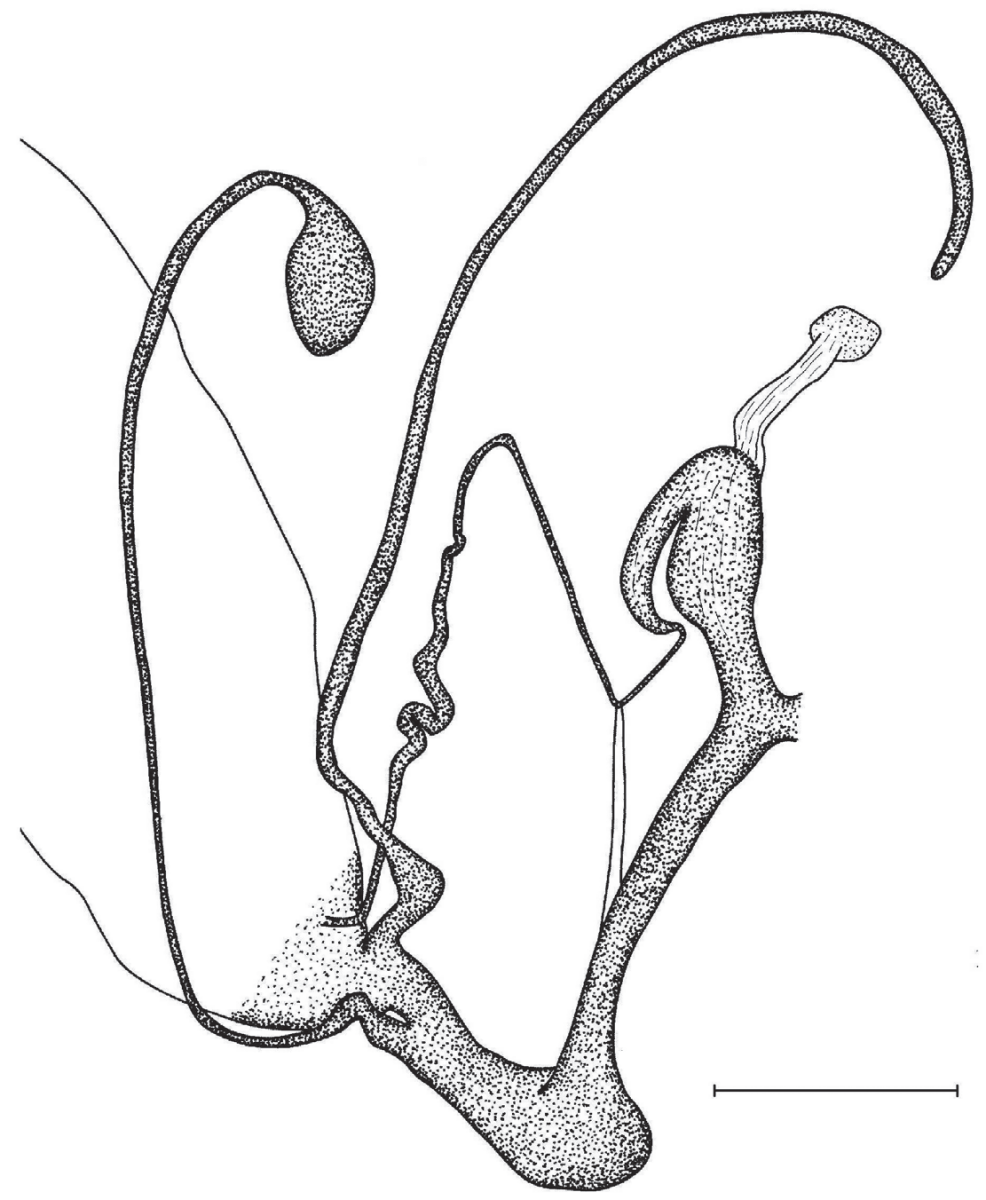
Reproductive anatomy of *Sicradiscus mansuyi* (Gude, 1908). Locality information: Cao Bằng Province, southern edge of Pác Rải, Trùng Khánh 3 km towards Quảng Uyên, left side of the road, 570 m, 22°48.961'N, 106°30.533'E, leg. Hunyadi, A., 28.05.2012. Scale represents 2 mm.

**Figure 28.**
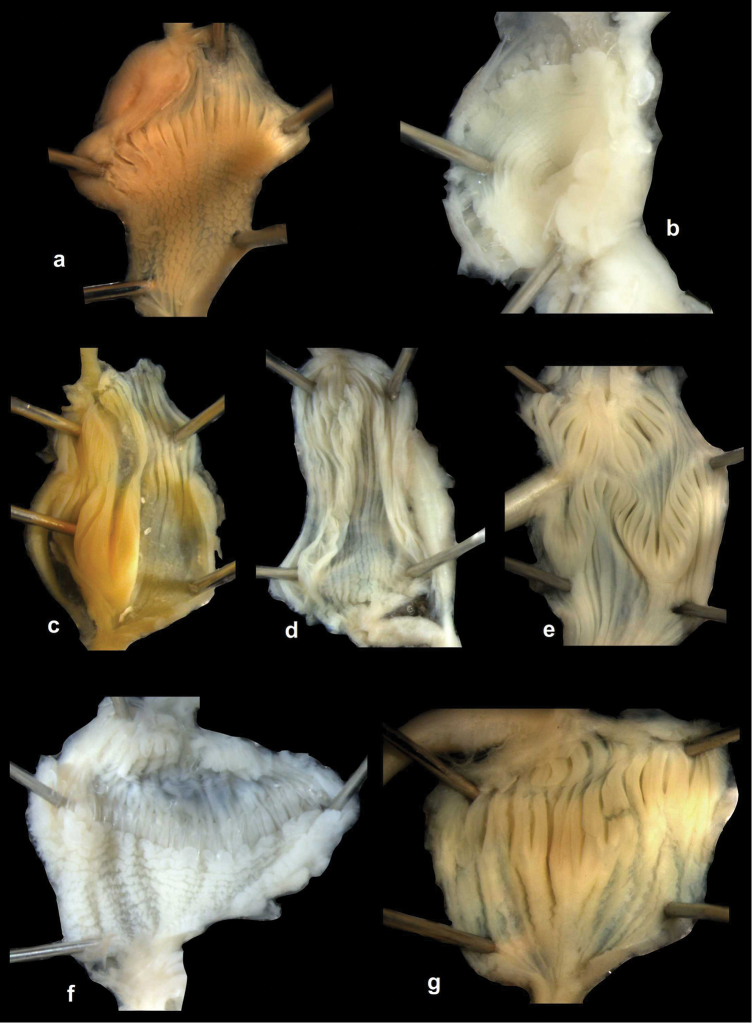
Inner walls of the penis of *Gudeodiscus* Páll-Gergely, 2013 species. **A** Gudeodiscus (Gudeodiscus) phlyarius (Mabille, 1887) (typical *fallax* specimen, for locality see Figure 21) **B** *Gudeodiscus giardi giardi* (Fischer, 1898) (for locality see Figure 19) **C** Gudeodiscus (Gudeodiscus) phlyarius (Mabille, 1887) (for locality see Figure 22) **D** Gudeodiscus (Gudeodiscus) fischeri (Gude, 1901) (for locality see Figure 17) **E** Gudeodiscus (Gudeodiscus) messageri raheemi Páll-Gergely & Hunyadi, ssp. n., 20080510A **F–G** Gudeodiscus (Gudeodiscus) villedaryi specimens collected at the same locality in two different dates: **F** November (2011/102) and **G** May (20090520A). All photos by B. Páll-Gergely.

**Figure 29.**
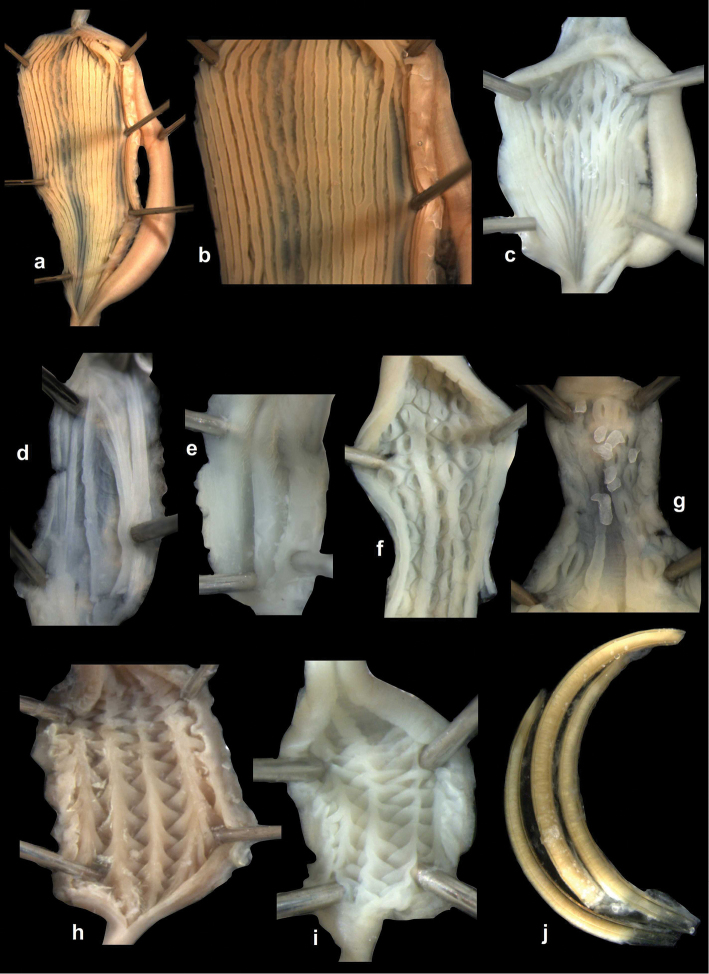
Inner walls of male reproductive organs of *Gudeodiscus* and *Halongella* gen. n. species. **A–B** penis of *Halongella schlumbergeri* (Morlet, 1886), 20071122D **C** penis of *Halongella fruhstorferi* (Möllendorff, 1901) (for locality see Figure 25) **D** epiphallus of *Gudeodiscus fischeri* (Gude, 1901), (for locality see Figure 18) **E** epiphallus of *Gudeodiscus giardi giardi* (Fischer, 1898), (for locality see Figure 19) **F** penial caecum of Gudeodiscus (Gudeodiscus) messageri raheemi Páll-Gergely & Hunyadi, ssp. n. (for locality see Figure 20) **G** penial caecum of Gudeodiscus (Gudeodiscus) messageri raheemi ssp. n. (for locality see Figure 28E) **H** epiphallus of *Halongella schlumbergeri* (for locality see Figure 26) **I** *Halongella fruhstorferi*, (for locality see Figure 25) **J** spermatophore of Gudeodiscus (Gudeodiscus) fischeri, (for locality see Figure 17). All photos by B. Páll-Gergely.

**Figure 30.**
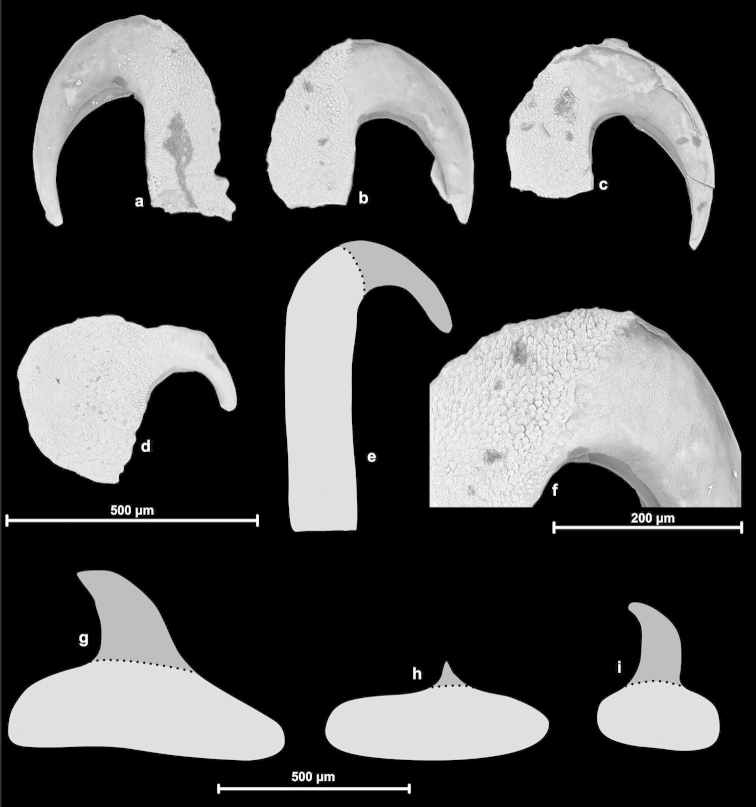
Calcareous claws found in pockets on the inner penial wall of *Gudeodiscus* and *Halongella* gen. n. species. **A–C, F** Gudeodiscus (Gudeodiscus) villedaryi (Ancey, 1888) (for locality see Figure 23) **D** *Gudeodiscus giardi giardi* (Fischer, 1898) (for locality see Figure 19) **E** Gudeodiscus (Gudeodiscus) fischeri (Gude, 1901) (for locality see Figure 19) **G–I** *Halongella schlumbergeri* (Morlet, 1886) (for locality see Figure 29A–B). The claws in case of Gudeodiscus (Gudeodiscus) fischeri and *Halongella schlumbergeri* were too fragile for dissecting out, therefore drawings are presented. All images by B. Páll-Gergely.

**Figure 31.**
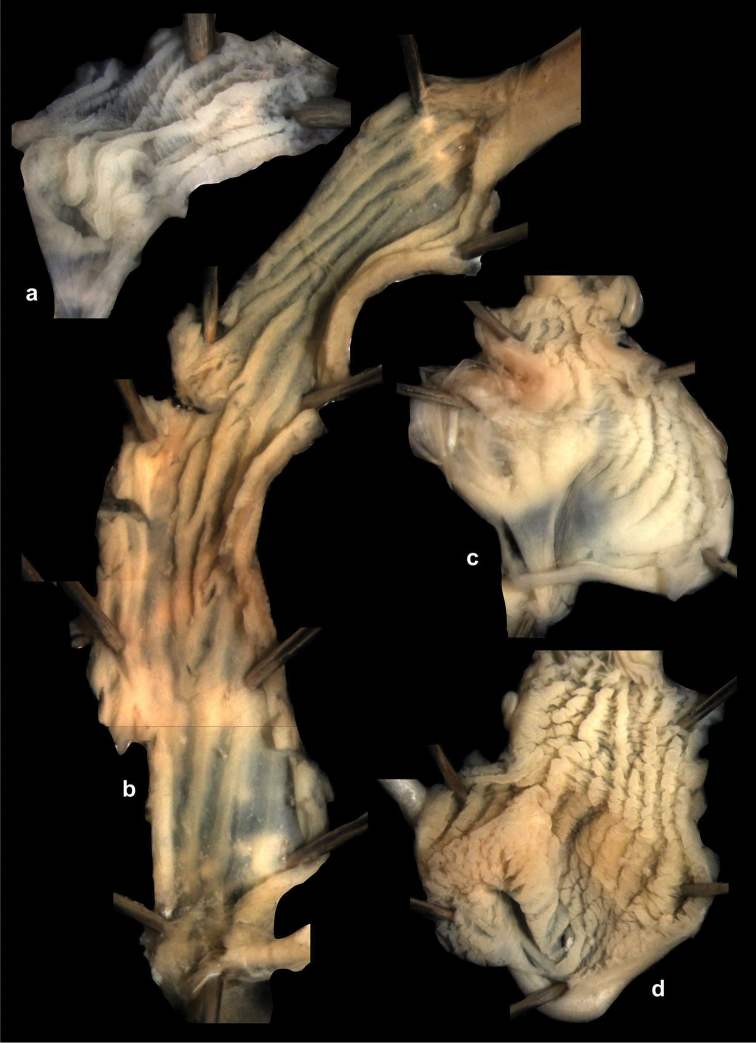
Inner wall of the vagina of *Sicradiscus* and *Gudeodiscus* species. **A** *Sicradiscus mansuyi* (Gude, 1908), (for locality see Figure 27) **B** Gudeodiscus (Gudeodiscus) messageri raheemi Páll-Gergely & Hunyadi, ssp. n. (for locality see Figure 20) **C** Gudeodiscus (Gudeodiscus) phlyarius (Mabille, 1887), Vn11-157 **D** Gudeodiscus (Gudeodiscus) fischeri (Gude, 1901) (for locality see Figure 17).

**Figure 32.**
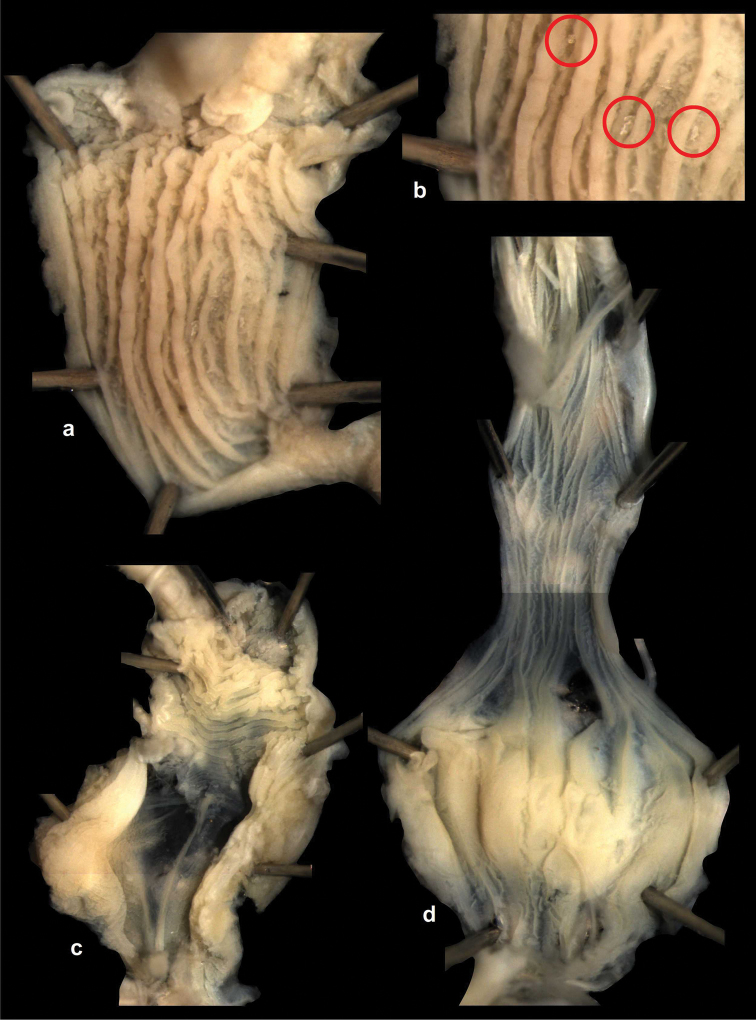
Inner wall of the vagina of *Halongella* gen. n. and *Gudeodiscus* species. **A–B** *Halongella fruhstorferi* (Möllendorff, 1901), red circles indicate calcareous granules (for locality see Figure 25) **C** Gudeodiscus (Gudeodiscus) giardi giardi (Fischer, 1898) (for locality see Figure 19) **D** Gudeodiscus (Gudeodiscus) villedaryi (Ancey, 1888) (for locality see Figure 24).

**Figure 33.**
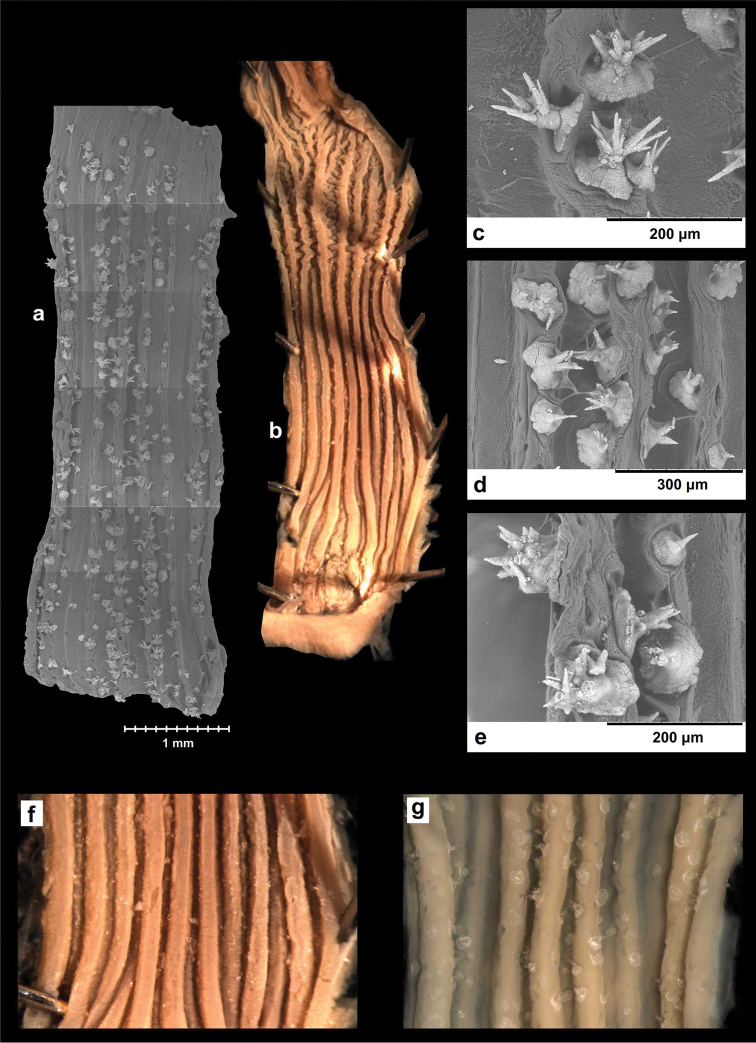
Inner wall of the vagina (**A–B, F–G**) and vaginal granules (**C–E**) of *Halongella schlumbergeri* (Morlet, 1886). “Specimen1” (gravid specimen, locality Vn11-172): **B, F**; “Specimen2” (not gravid specimen, locality 20071122D): **A, C–E, G.**

**Figure 34.**
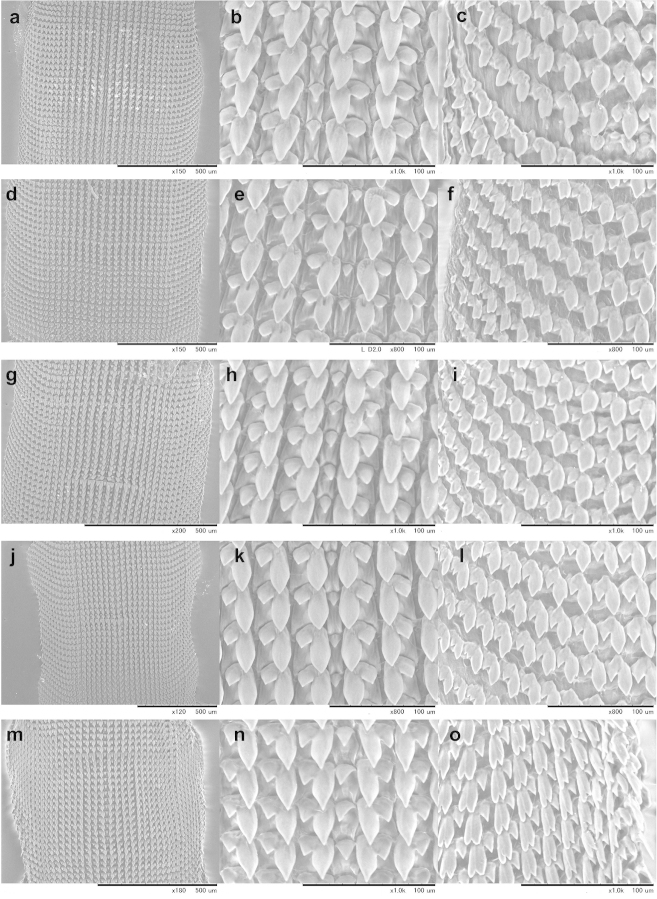
SEM images of radulae of *Gudeodiscus* species. **A, D, G, J, M** show the middle part of the radula **B, E, H, K, N** show the central tooth and the first 2–3 pairs of laterals **C, F, I, L, O** show the marginals. **A–C** Gudeodiscus (Veludiscus) emigrans otanii Páll-Gergely & Hunyadi, 2013, China, Guangxi, Yizhou Shi, Aishan Xiang, Xiannuyan, ca 170 m, 24°29.292'N, 108°34.057'E, leg. Nakahara, Y., Ohara, K., Okubo, K. & Otani, J. U., 13.11.2004. **D–F** Gudeodiscus (Veludiscus) eroessi eroessi Páll-Gergely & Hunyadi, 2013, China, Guangxi, Guigang Shi, Guzhang Xiang, beyond Chuanshan village, ca 155 m, 23°20.848'N, 109°19.256'E, leg. Nakahara, Y., Ohara, K., Okubo, K. & Otani, J. U., 09.11.2004 **G–I** Gudeodiscus (Veludiscus) okuboi Páll-Gergely & Hunyadi, 2013, Guangxi, Guigang Shi, Guzhang Xiang, road to Wushan Xiang, ca 130 m, 23°21.178'N, 109°17.432'E, leg. Nakahara, Y., Ohara, K., Okubo, K. & Otani, J. U., 09.11.2004. **J–L** Gudeodiscus (Veludiscus) pulvinaris pulvinaris (Gould, 1859), China, Hong Kong Peak, leg. Miu Yeung, June 2013 **M–O** Gudeodiscus (Gudeodiscus) fischeri (Gude, 1901), (for locality see Figure 17). All photos by B. Páll-Gergely.

**Figure 35.**
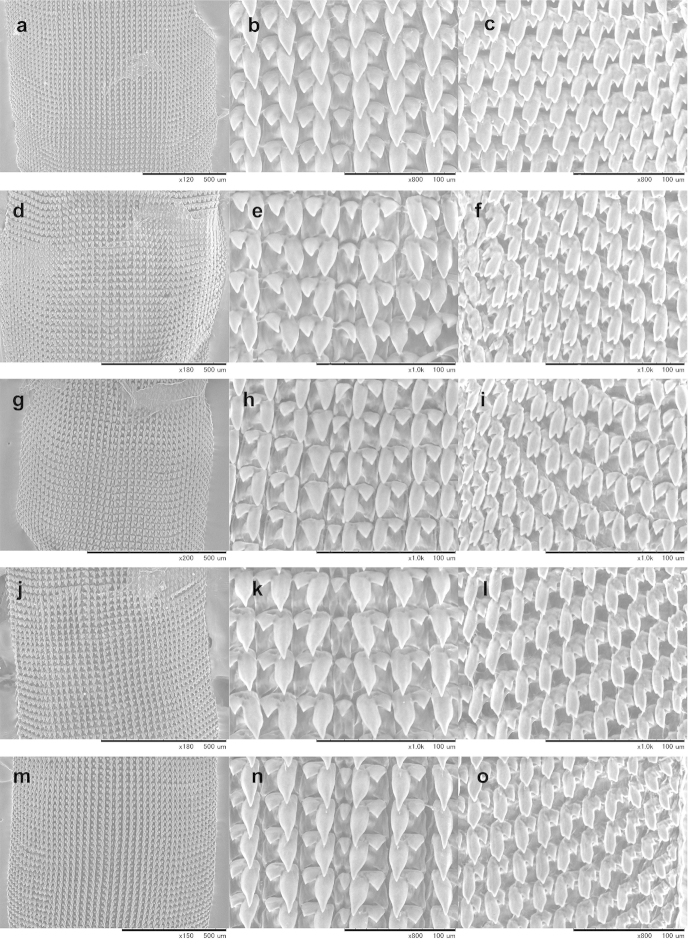
SEM images of radulae of *Gudeodiscus* species. **A, D, G, J, M** show the middle part of the radula **B, E, H, K, N** show the central tooth and the first 2–3 pairs of laterals **C, F, I, L, O** show the marginals. **A–C** Gudeodiscus (Gudeodiscus) giardi giardi (Fischer, 1898) (for locality see Figure 19) **D–F** Gudeodiscus (Gudeodiscus) messageri raheemi Páll-Gergely & Hunyadi, ssp. n. (for locality see Figure 20) **G–I** Gudeodiscus (Gudeodiscus) multispira (Möllendorff, 1883), China, Guangxi, Qingshan, Qingshan Zhen, Lipu Xian, ca 250 m, 24°26.189'N, 110°20.008'E, leg. Nakahara, Y., Ohara, K., Okubo, K. & Otani, J. U., 12.11.2004. **J–L** Gudeodiscus (Gudeodiscus) phlyarius (Mabille, 1887), Lạng Sơn Province, ca. km. 50 of road 1B, 10 km to Bình Gia, 21°53.911'N, 106°25.664'E, leg. Hemmen, Ch. & J., 01.04.2011. **M–O** Gudeodiscus (Gudeodiscus) villedaryi (Ancey, 1888), (for locality see Figure 23). All photos by B. Páll-Gergely.

**Figure 36.**
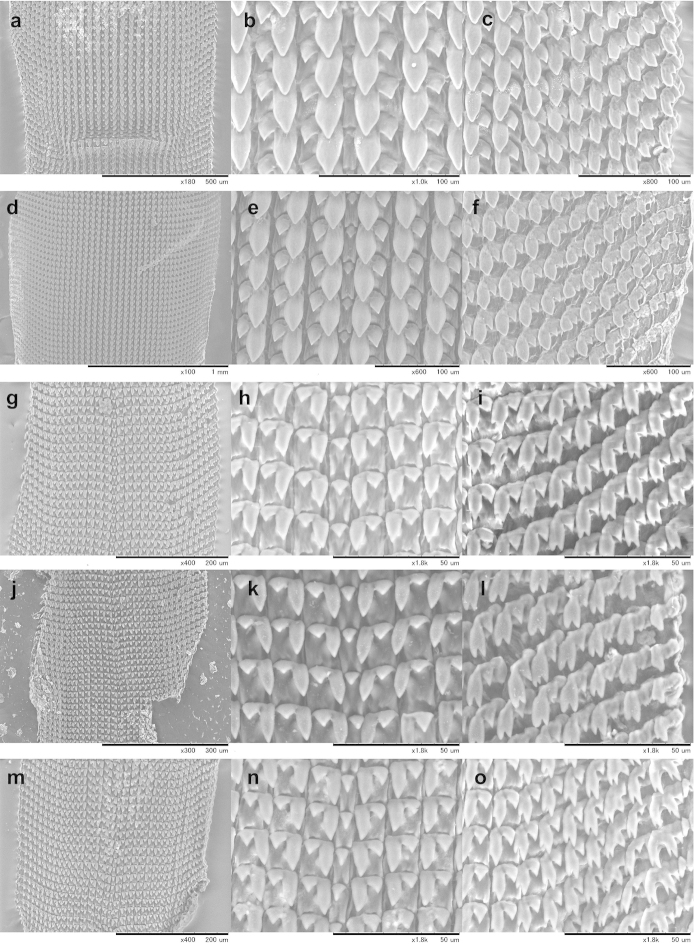
SEM images of radulae of *Halongella* and *Sicradiscus* species. **A, D, G, J, M** show the middle part of the radula; **B, E, H, K, N** show the central tooth and the first 2–3 pairs of laterals **C, F, I, L, O** show the marginals. **A–C** *Halongella fruhstorferi* (Möllendorff, 1901) (for locality see Figure 25) **D–F** *Halongella schlumbergeri* (Morlet, 1886) (for locality see Figure 29A–B) **G–I** *Sicradiscus invius* (Heude, 1885), China, Sichuan, Dujiangyan Shi, Taian Zhen, Sanlong Shuijingrongdong, ca 1090 m, 30°55.039'N, 103°29.662'E, leg. Hosoda, T., Ohara, K., Okubo, K., Otani, J. U., 17.09.2013. **J–L** *Sicradiscus mansuyi* (Gude, 1908), (for locality see Figure 27) **M–O** *Sicradiscus schistoptychia* (Möllendorff, 1886), China, Hunan, Yongzhou Shi, Ningyuan Xian, Jiuyishan Yaozuxiang, Jiuyishan Guojia Senlin Gongyuan, old maple forest, 25°21.200'N 111°58.696'E, 450 m, leg. Hunyadi, A., 11.11.2010. All photos by B. Páll-Gergely.

**Figure 37.**
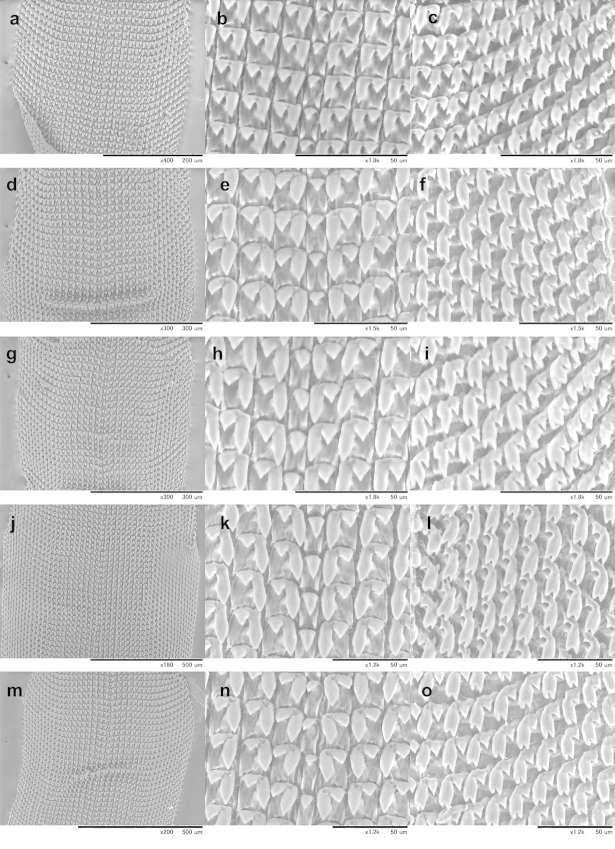
SEM images of radulae of *Sicradiscus* and *Sinicola* species. **A, D, G, J, M** show the middle part of the radula **B, E, H, K, N** show the central tooth and the first 2–3 pairs of laterals **C, F, I, L, O** show the marginals. **A–C** *Sicradiscus transitus* Páll-Gergely, 2013, Guangxi, Hechi Shi, Tiane Xian, Qimu Xiang, near Lahaoyan, 650 m, 24°51.359'N, 107°11.407'E, leg. Hunyadi, A. & Szekeres, M., 12.09.2013. **D–F** *Sinicola asamiana* Páll-Gergely, 2013, Sichuan, Dujiangyan Shi, Qingchengshan Zhen, Jinbian Yan, 30°55.234'N, 103°29.483'E, 930 m, leg. Hosoda, T., Ohara, K., Okubo, K., Otani, J. U., 16.09.2013. **G–I** *Sinicola emoriens* (Gredler, 1881), Hunan, Yongzhou Shi, Lingling Qu, Dengjiachong, rocky wall, 125 m, 26°13.808'N, 111°35.907'E, leg. Hunyadi, A., 8.11.2010. **J–L** *Sinicola fimbriosa* (von Martens, 1875), China, Hunan, Hengyang Shi, Nanyue Qu, Yuelin Xiang, southern part of Heng Shan, Chuanyan Shilin, near Ban Shanting, 590 m, 27°16.435'N 112°42.195'E, leg. A. Hunyadi 20.10.2010. **M–O** *Sinicola jugatoria* (Ancey, 1885), China, Hubei, Yichang Shi, Changyang Tujiazu Zizhixian, Qingjiang Hualang Fengjingqu, Geheyan Shuiku, Wuluozhougli Shan, 260 m, 30°25.805'N 110°59.254'E, leg. A. Hunyadi 31.10.2010. All photos by B. Páll-Gergely.

**Figure 38.**
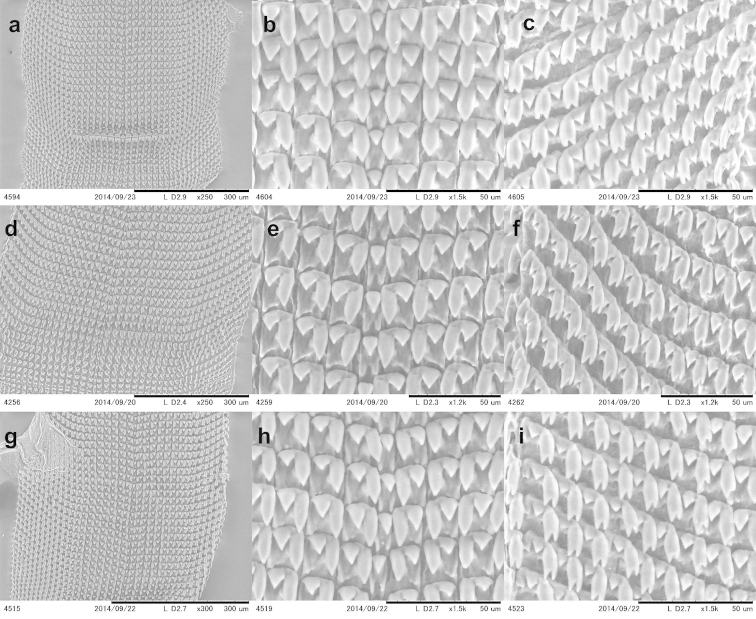
SEM images of radulae of *Sinicola* species. **A, D, G** show the middle part of the radula **B, E, H** show the central tooth and the first 2–3 pairs of laterals **C, F, I** show the marginals. **A–C** *Sinicola murata* (Heude, 1885), Sichuan, Dujiangyan Shi, Qingchengshan Zhen, Jinbian Yan, 30°55.234'N, 103°29.483'E, 930 m, leg. Hosoda, T., Ohara, K., Okubo, K., Otani, J. U., 16.09.2013. **D–F** *Sinicola reserata azona* (Gredler, 1887), Guizhou, Tongren Shi, Wanshanchen dirt road, Xianrendong, ca 865 m, 27°31.785'N, 109°13.008'E, leg. Ohara, K., Okubo, K. & Otani, J. U., 10.5.2010. **G–I** *Sinicola stenochila* (Möllendorff, 1885), Hubei, Enshi Tujiazu Miaozu Zizhizhou, Badong Xian, Badong E, Bashan Senlin Gongyuan, 300 m W from the entrance, 220 m, 31°01.684'N, 110°25.094'E, leg. Hunyadi, A., 3.11.2010. All photos by B. Páll-Gergely.

**Figure 39.**
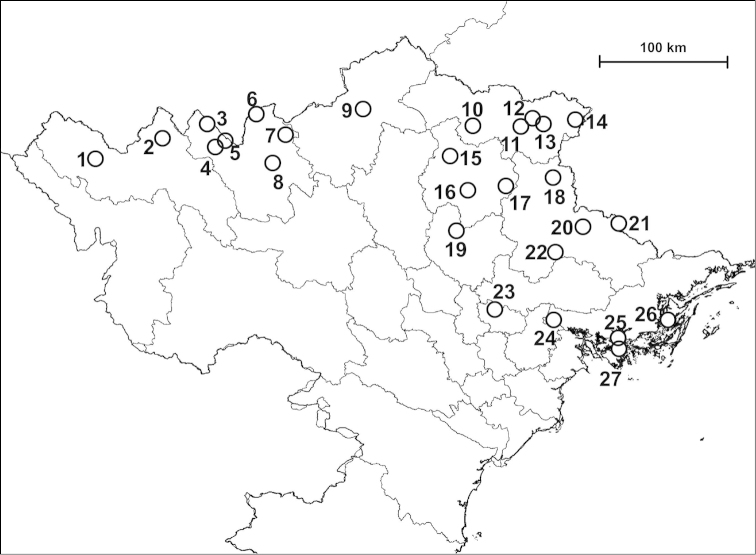
Locations mentioned in literature on plectopylid taxonomy. **1** Muong-Bo **2** Phony-Tho **3** Trinh-Thuong **4** Muong-Hum **5** Nat-Son (Nhat Son) **6** Muong-Kong **7** Long-Ping **8** Pac-Kha **9** Ha-Giang **10** Tinh-Tuc **11** Cao-Bang **12** Déo-Ma-Phuc **13** Quang-Huyen **14** Ha-Lang **15** Cho-Ra **16** Bac-Khan **17** Nac-Ri **18** That-Khé **19** Cho-Moi **20** Lang-Son **21** Mansongebirge **22** Than-Moi **23** Bac-Ninh **24** Dong-Trieu **25** Bah-Mun **26** Kebao **27** Baie d’Along. The locations of “Col de Nuages” (Clouds Pass) could not be located. It is probably situated on Lao Kay Province, close to Muong-Hum.

**Figure 40.**
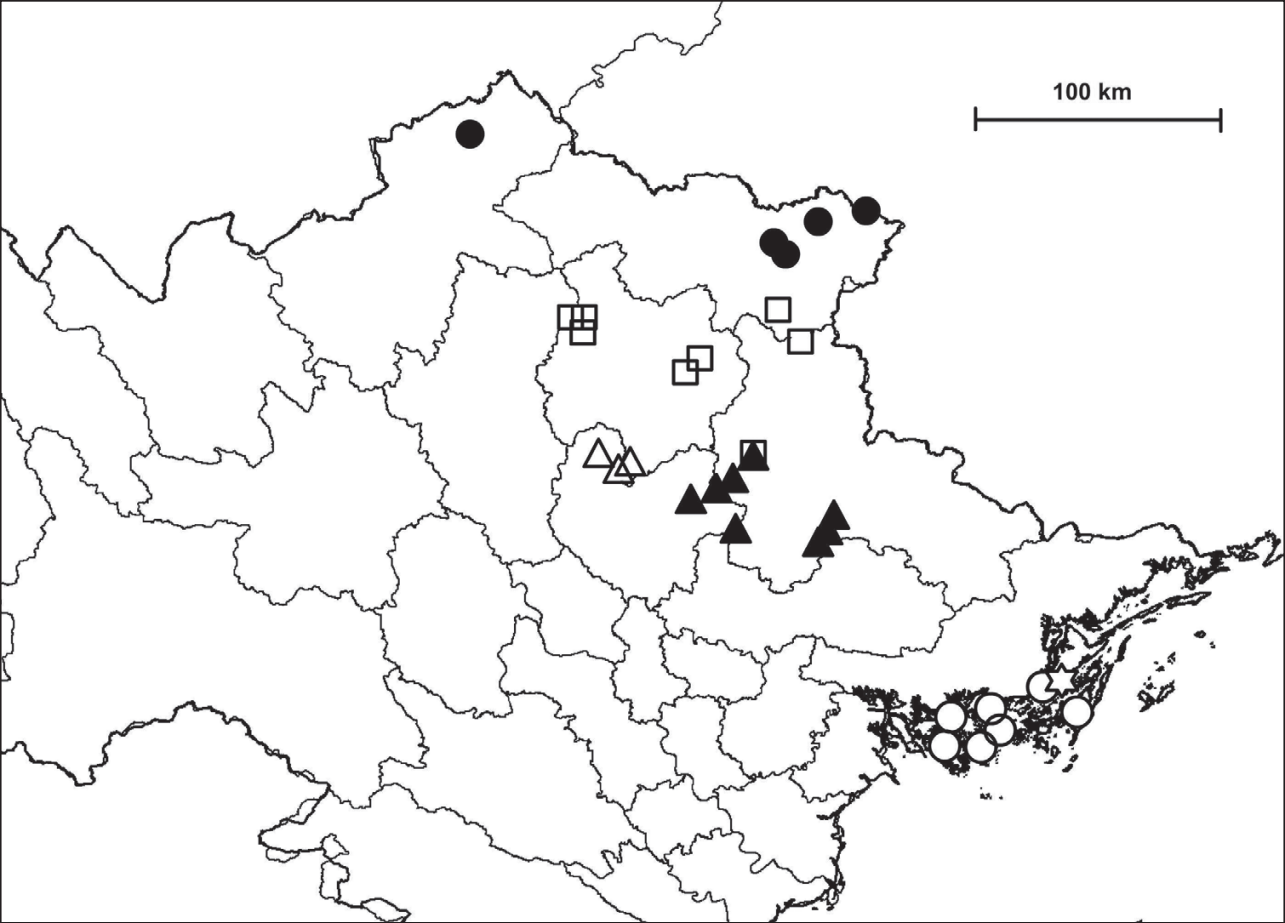
Distribution of *Gudeodiscus*, *Halongella* gen. n. and *Sicradiscus* species. Legends: empty circle: *Halongella schlumbergeri* (Morlet, 1886), star (close to the circles): *Halongella fruhstorferi* (Möllendorff, 1901), empty triangle: Gudeodiscus (Gudeodiscus) dautzenbergi (Gude, 1901), filled triangle Gudeodiscus (Gudeodiscus) villedaryi (Ancey, 1888), empty square: Gudeodiscus (Gudeodiscus) anceyi (Gude, 1901), filled circle: *Sicradiscus mansuyi* (Gude, 1908).

**Figure 41.**
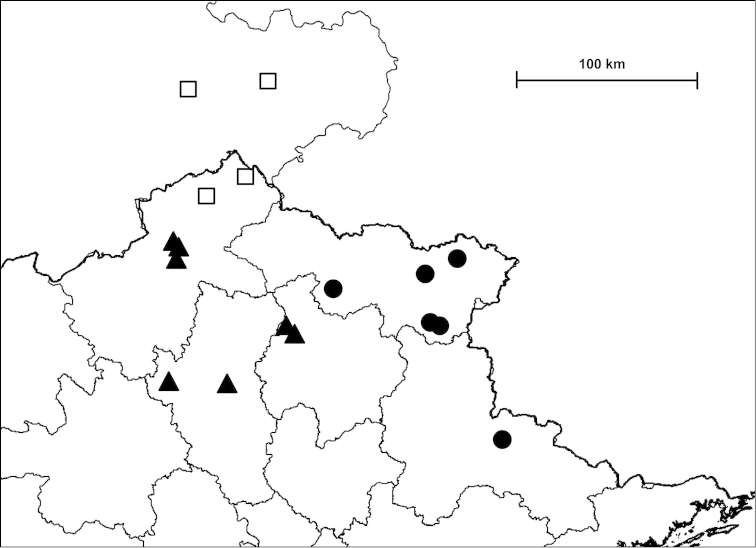
Distribution of *Gudeodiscus* species. Legends: filled circle: Gudeodiscus (Gudeodiscus?) suprafilaris (Gude, 1908), triangle: Gudeodiscus (Gudeodiscus) fischeri (Gude, 1901), empty square: Gudeodiscus (Gudeodiscus?) cyrtochilus (Gude, 1909).

**Figure 42.**
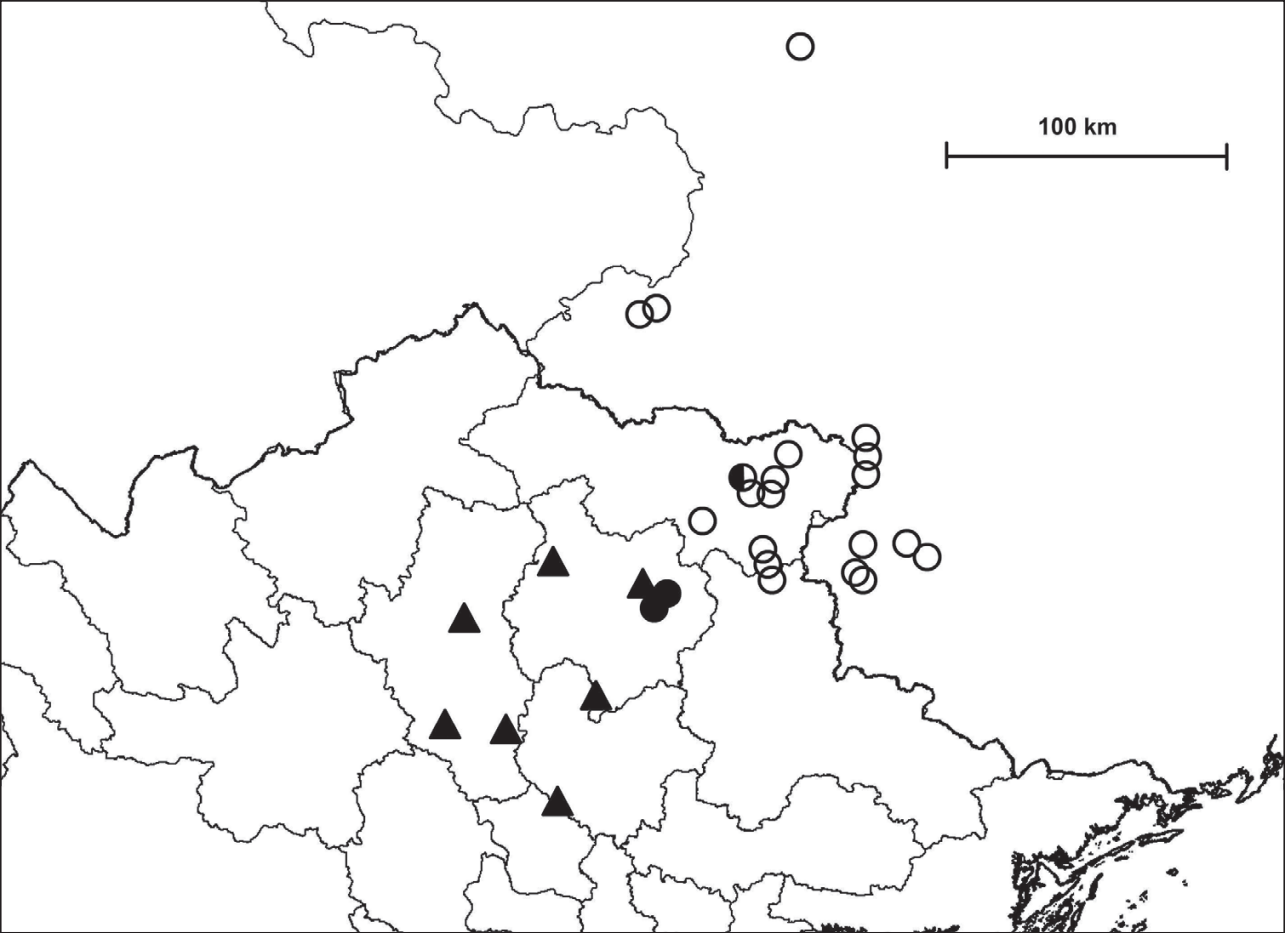
Distribution of *Gudeodiscus* species. Legends: filled triangle: Gudeodiscus (Veludiscus) emigrans quadrilamellatus Páll-Gergely, 2013, empty circle: Gudeodiscus (Gudeodiscus) giardi giardi (Fischer, 1898), filled circle: Gudeodiscus (Gudeodiscus?) francoisi (Fischer, 1899), semi filled circle: co-occurrence of the latter two species.

**Figure 43.**
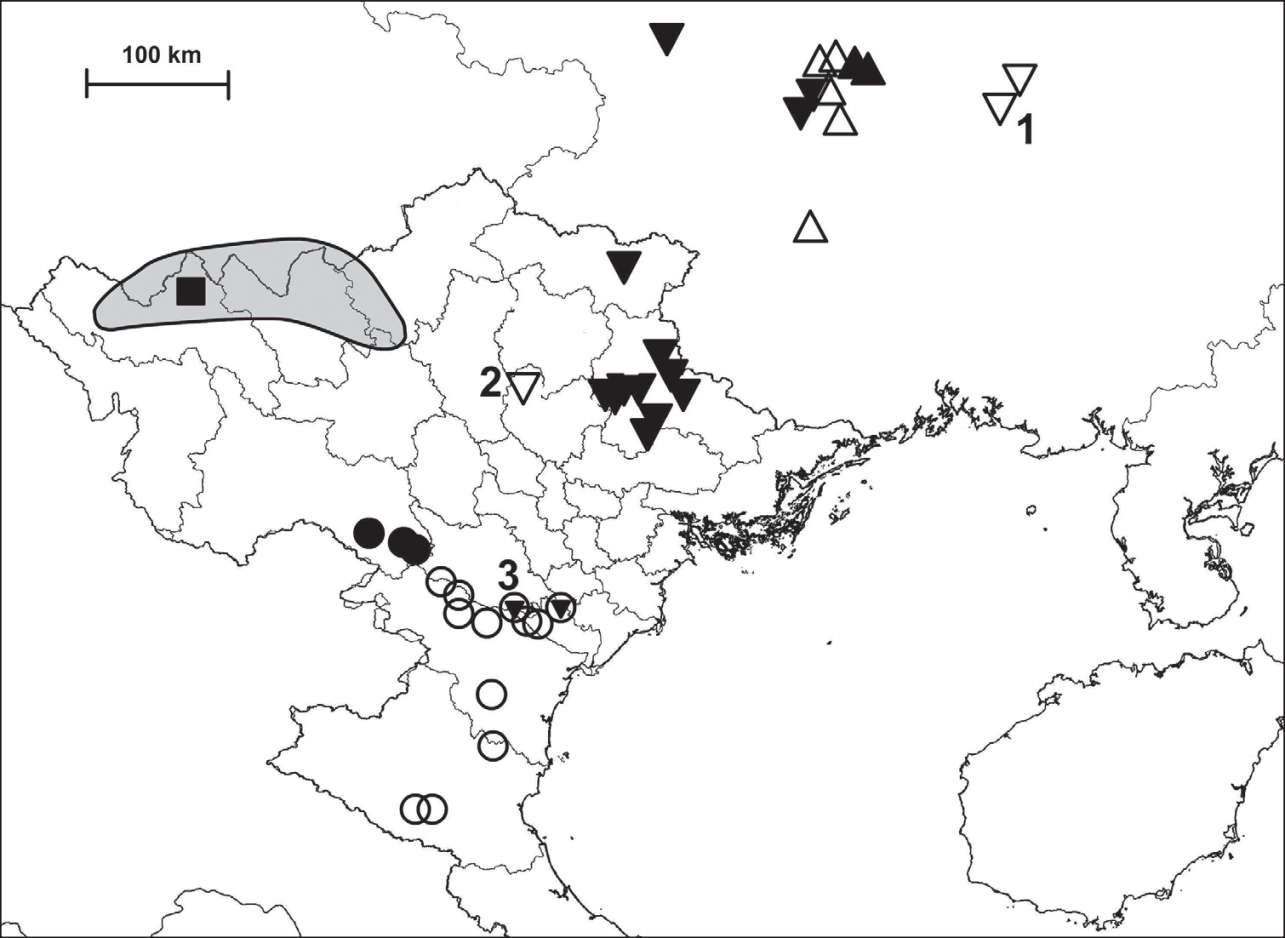
Distribution of *Gudeodiscus* Páll-Gergely, 2013 species. Legends: filled triangle, top down: typical Gudeodiscus (Gudeodiscus) phlyarius (Mabille, 1887), filled triangle, top up: “*Gudeodiscus phlyarius werneri* Páll-Gergely, 2013” (synonym of *phlyarius*); empty triangle, top up: Gudeodiscus (Gudeodiscus) phlyarius populations showing transitional characters towards *werneri* in terms of shell shape; empty triangle, top down: atypical Gudeodiscus (Gudeodiscus) phlyarius; empty circle: *Gudeodiscus messageri raheemi* ssp. n., filled circle: Gudeodiscus (Gudeodiscus?) hemmeni sp. n. (in all localities it co-occurs with Gudeodiscus (Gudeodiscus) messageri raheemi ssp. n.); circle with filled triangle in the middle: co-occurrence of Gudeodiscus (Gudeodiscus) messageri raheemi ssp. n. and atypical Gudeodiscus (Gudeodiscus) phlyarius. The shaded area indicates the area inhabited by Gudeodiscus (Gudeodiscus) messageri messageri (Gude, 1909) and “*anterides*”, “*fallax*” and “*gouldingi*”-like populations of Gudeodiscus (Gudeodiscus) phlyarius. Filled square indicates the position of Phong-Tho, the type locality of *Plectopylis verecunda* Gude, 1909 (synonym of *Gudeodiscus phlyarius*). Numbers 1–3 refer to atypical populations assigned to Gudeodiscus (Gudeodiscus) phlyarius. For explanation, see text.

**Figure 44.**
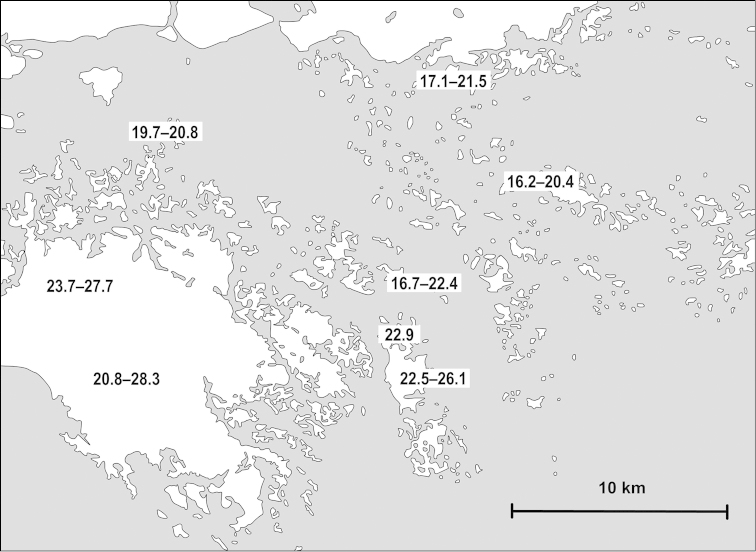
Shell widths of *Halongella schlumbergeri* (mm) in the Halong Bay Area.

**Figure 45.**
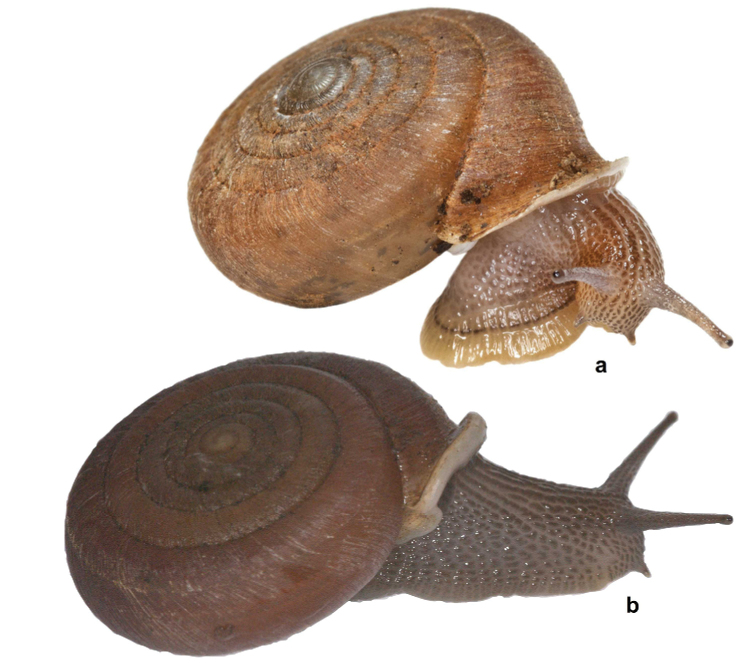
Living specimens of Gudeodiscus (Gudeodiscus) giardi giardi (Fischer, 1898) (**A**), Cao Bằng Province, Hòa An District, Nguyễn Huệ Commune, small hill just outside of Khau Trang Village, 22°33.510'N, 106°10.294'E, leg. Naggs, F. et al. 22.06.2011.; and *Halongella schlumbergeri* (Morlet, 1886) (**B**), Halong Bay area, Vietnam. Photos: F. Naggs.

### Identification key to Vietnamese and Chinese plectopylid genera

**Table:** 

1	body whorl keeled	**2**
–	body whorl rounded	**3**
2(1)	anterior lamella absent or present as small denticles	***Sinicola***
–	anterior lamella present	***Sicradiscus***
3(1)	shell smaller than 9 mm, smooth at its base, and has a strong apertural fold	***Sicradiscus***
–	shell larger than 9 mm; if it is smaller than 12 mm and smooth, then it has no apertural fold	**4**
4(3)	inner penial wall with distinct pockets standing in 1 or 2 rows	**5**
–	inner penial wall with parallel folds without large pockets	***Halongella***
5(4)	penial retractor simple	**Gudeodiscus (Gudeodiscus)**
–	penial retractor is covered with additional muscle fibres which attach on the distal end of the penis	**Gudeodiscus (Veludiscus)**

### Identification key to Vietnamese species (regardless of generic association)

**Table:** 

1	shell smaller than 12 mm	**2**
–	shell larger than 12 mm	**5**
2(1)	apertural fold well visible	**3**
–	apertural fold missing or inconspicuous, very weak	***cyrtochilus***
3(2)	ventral surface smooth, glossy	***mansuyi***
–	ventral surface sculptured	**4**
4(3)	free plicae above and below the anterior lamella absent	***anceyi***
–	upper and lower plicae free from the anterior lamella	***hemmeni* sp. n.**
5(1)	dorsal reticulate and ventral smooth areas change abruptly	***suprafilaris***
–	dorsal and ventral sculpture do not change abruptly	**6**
6(5)	parietal wall with a single lamella	**7**
–	parietal wall with two lamellae (or the anterior lamella is dissolved into small denticles)	**10**
7(6)	anterior to the parietal lamella there are four parallel horizontal plicae	***emigrans quadrilamellatus***
–	anterior to the lamella there are two horizontal plicae, one above, one below	**8**
8(7)	shell about 13–14 mm	***fruhstorferi***
–	shell larger than 15 mm	**9**
9(8)	shell strongly-built, seemingly smooth, callus elevated	***schlumbergeri***
–	shell relatively thin, regularly ribbed, callus weak	***emigrans emigrans***
10(6)	shell thin-walled, callus weak, sculpture weak, rather glossy	**11**
–	shell more strongly-built, callus strong	**12**
11(10)	shell flat or nearly flat, umbilicus wide	***fischeri***
–	spire somewhat elevated, umbilicus rather narrow	***infralevis***
12(10)	umbilicus very narrow, dorsal surface domed	**13**
–	umbilicus moderately narrow, dorsal surface moderately domed	**14**
13(12)	shell yellowish, callus blunt, rather low	***francoisi***
–	shell brownish, callus very much elevated, high, rather sharply defined	***giardi***
14(12)	shell regularly ribbed, rather thin walled	**15**
–	shell thick walled, strongly built	**17**
15(14)	anterior lamella usually free from the lower plica	***phlyarius***
–	anterior lamella in contact with the lower plica, or the lower plica is dissolved into denticles	**16**
16(15)	anterior lamella is in contact with the lower plica, lower plica do not extend beyond the lamella in anterior direction	***messageri messageri***
–	anterior lamella dissolved into small denticles; or if not dissolved, the lower plica extends beyond the lamella in anterior direction	***messageri raheemi* ssp. n.**
17(14)	apertural fold horizontal	***schlumbergeri***
–	apertural fold oblique	**18**
18(17)	additional lower plica present under the lamellae	***villedaryi***
–	additional lower plica absent under the lamellae	***dautzenbergi***

### Taxonomic positions of the genera *Gudeodiscus*, *Halongella* gen. n., *Sicradiscus* and *Sinicola*

The “Eastern Plectopylidae” (see Páll-Gergely and Hunyadi 2013), namely taxa inhabiting China, Vietnam, Taiwan and Okinawa (Japan) are conchologically relatively diverse. Their common features are the ribbed protoconch and the absence of the long parietal horizontal plica. The genus *Endothyrella*, which mainly inhabits north-eastern India also shares these features with the genera of “Eastern Plectopylidae”. Therefore *Endothyrella* is possibly a close relative to the genera *Gudeodiscus*, *Halongella* gen. n., *Sicradiscus* and *Sinicola*. The genera of “Western Plectopylidae” (*Endoplon*, *Chersaecia* and *Plectopylis*) have smooth but matt or “tuberculated” embryonic whorls and usually long horizontal parietal plicae (a main plica and a lower plica) which run to the peristome. Some *Endothyrella* species have long lower and main plicae, but these may not be homologous with those in the *Chersaecia* and *Plectopylis*. Some species which have been assigned to the genus *Chersaecia* (*andersoni* W. Blanford, 1869, *laomontana* L. Pfeiffer, 1863, *oglei*, *serica*, *munipurensis*) also possess ribbed protoconchs. These probably do not belong to any of the genera mentioned herein, and their taxonomic status require revision.

In the revision of the Chinese Plectopylidae (Páll-Gergely and Hunyadi 2013), three genera were recognized, namely *Gudeodiscus*, *Sicradiscus* and *Sinicola*. The most important shell characters for recognition of *Sinicola* are the following: body whorl keeled; periostracal folds usually present on the keel; apertural fold almost always absent; the anterior parietal lamella is absent or present only in some small, separate denticles. *Gudeodiscus* exhibits the following characters: body whorl rounded; periostracal folds absent; apertural fold often present; anterior parietal lamella often present. Both genera inhabit restricted geographical areas with minor overlaps; *Sinicola* ranges from Middle Sichuan to northern Guangxi, Guangdong and eastern Hunan, whereas *Gudeodiscus* ranges from northern Vietnam to southern Hunan and southern Guangdong. Reproductive anatomical investigations (Páll-Gergely and Hunyadi 2013, Páll-Gergely and Asami 2014) found that *Sinicola* species exhibit a ribbed inner penial wall with a few tiny calcareous granules. The ribs are more prominent in the distal part of the penis or continuous until the atrium but this varies between individuals. Examples of *Gudeodiscus* usually also have parallel folds, but they have characteristic small pockets arranged in one or two more or less straight transverse lines in the distal penis. These pockets contain calcareous granules, probably only during the mating period (see discussion on anatomy and biology). The genus *Gudeodiscus* is divided into two groups based on the morphology of the distal penis-penial caecum-retractor muscle complex. In one type, the epiphallus is slender, cylindrical, and addition to the retractor muscle, which attaches on the penile caecum, several muscle fibres attach to the penis itself. In the other type the epiphallus has a somewhat thickened proximal part, and has no additional muscle fibres attached to the penis (Páll-Gergely and Hunyadi 2013, Páll-Gergely and Asami 2014). It may not be legitimate to subdivide the genus on the basis of this anatomical difference, when the shell characters do not show clear distinction (Páll-Gergely and Asami 2014). For example, *Gudeodiscus eroessi* (first type) and *Gudeodiscus multispira* (second type) are conchologically very similar. However, we found that radula traits distinguish between them as well as the genital anatomy does. Therefore we find it well supported to separate these two groups into different subgenera (*Gudeodiscus* and *Veludiscus* subgen. n.).

The taxonomic position of the species classified within *Sicradiscus* is problematic. *Sicradiscus* was erected for several, small bodied species which inhabit a large area ranging from Sichuan to Okinawa, Japan. There is continuous variation across the genus *Sicradiscus* in terms of shell characters. *Sicradiscus invius*, *Sicradiscus securus*, *Sicradiscus mansuyi* and *Sicradiscus feheri* have a rounded body whorl and possess a strong apertural fold. In contrast, *Sicradiscus schistoptychia*, *Sicradiscus diptychia*, *Sicradiscus cutisculptus*, *Sicradiscus ishizakii* and *Sicradiscus hirasei* have a shouldered body whorl and lack the apertural fold. The two groups are within the same genus because *Sicradiscus transitus* is similar to *Sicradiscus schistoptychia* in possessing divided palatal plicae and a keeled body whorl, at the same time having a strong apertural fold similar to that of *Sicradiscus feheri*. Moreover, *Sicradiscus transitus* ranges between *Sicradiscus feheri* and *Sicradiscus schistoptychia* geographically. The present and a previous study (Páll-Gergely and Asami 2014) revealed that the inner morphology of the penis in *Sicradiscus schistoptychia* is similar to that of *Sinicola*, whereas *Sicradiscus invius*, *Sicradiscus mansuyi* and *Sicradiscus transitus* are similar to *Gudeodiscus* in that trait. Separating some *Sicradiscus* species into *Gudeodiscus* and others in *Sinicola* based on the penial morphology does not resolve their taxonomy because of the large conchological similarity among *Sicradiscus* species. An alternative classification might be to place all *Gudeodiscus*, *Sicradiscus* and *Sinicola* species into one genus because of the transitional features of *Sicradiscus* between *Sinicola* and *Gudeodiscus*. However, our study does not support this because both *Sinicola* and *Gudeodiscus* show clear synapomorphic characters and signs of their separate major radiations in different geographic areas. The most possible explanation is that *Sicradiscus* species represent basal lineages within the *Gudeodiscus*–*Sicradiscus*–*Sinicola* complex, in which others diverged into the two lineages, one with the keeled body whorl and folded penial wall and the other with the rounded body whorl and pocketed penial wall. *Sicradiscus* species may probably have undergone only slight conchological changes. This hypothesis is supported by the geographic distribution of most *Sicradiscus* species, roughly between the areas of *Gudeodiscus* and *Sinicola*.

*Plectopylis schlumbergeri* and *Plectopylis fruhstorferi* had parallel folds on the inner penial wall and calcareous granules were found between the parallel folds all along the penis. In both subgenera of the genus *Gudeodiscus* however, the pockets for calcareous granules are arranged in one or two rows, and they are absent elsewhere. Based on this morphological character, they are moved to a new genus, *Halongella*. Additionally, *Halongella* gen. n. species lack a penial caecum, which was found in the majority of *Gudeodiscus* species.

### Anatomy and biology

Stoliczka (1871) described the organ proximal to the gametolytic sac of *Plectopylis* as “a shorter, more muscular gland which appears to represent the arrow or amatorial gland”. Pilsbry (1894) noted this as “an organ of unknown homology, either a dart sack, a diverticulum of the spermatheca or an appendicula”. A spermatophore was found inside this organ of *Gudeodiscus fischeri*. This suggests that the organ is a diverticulum, starting from the wall of the distal end of the vagina/beginning of pedunculus. In most stylommatophoran land snails the diverticulum derives from the stalk of the gametolytic sac. The only exception known before this study was the subfamily Garniierinae (family Clausiliidae), in which the diverticulum derives from the pedunculus (Szekeres 1998).

The inner walls of the male genital organs, especially the penis, show a large diversity across the genera *Gudeodiscus*, *Halongella* gen. n., *Sicradiscus* and *Sinicola*. *Sinicola* and *Halongella* gen. n. have parallel folds on the inner penial wall, occasionally with tiny, usually flat calcareous granules, often without characteristic shapes. The penial wall of *Gudeodiscus* species is usually also characterized by folds, but also pockets arranged in one or two rows in the distal part of the penis. The rows can be straight (e.g. *Gudeodiscus giardi* and *Gudeodiscus villedaryi*), can follow a bell-shaped line (*Gudeodiscus fischeri*), or waves (*Gudeodiscus messageri raheemi* ssp. n.) on the opened penal wall. *Sicradiscus* species have both types of penial sculpture (with and without pockets) (Páll-Gergely and Asami 2014, and this study). In most *Gudeodiscus* specimens the granules are hook or claw-like, and each of them is placed within a pocket on the wall of the head of the penis. Two dissected specimens of *Gudeodiscus phlyarius* (typical *fallax* specimens), however, had flat, oval granules within the penial pockets. It is not clear whether this shape of granules is stable throughout the life span or dependent on season or age. In the revision of the Chinese species (Páll-Gergely and Hunyadi 2013) we described that calcareous hooks are easily removable from the folds in the penial internal wall. In the case of Vietnamese specimens (*Gudeodiscus giardi*, *Gudeodiscus fischeri* and *Gudeodiscus villedaryi*), however, the claws were attached into the wall inside the pocket and were difficult to remove. The SEM images of removed claws revealed that the base of each claw, which was buried into the pocket wall, is granulated in the surface, whereas the exposed tip of each claw was smooth. The hooks from the penis lumen of Chinese *Gudeodiscus phlyarius* (figured specimen in Páll-Gergely and Hunyadi 2013) dissolved with no remains in 90% lactic acid. Thus, these granules may consist of calcium carbonate.

The penial claws or hooks known in other stylommatophoran families (e.g. Zonitidae s.l., Streptaxidae, *Cryptazeca*) do not seasonally disappear and are fixed to the internal wall, because to our knowledge, hook-less specimens have not been reported in contrast to those in Plectopylidae (see also Páll-Gergely and Hunyadi 2013). Those of *Cryptazeca* and Streptaxidae are not calcareous (Visser 1973, Verdcourt 1979, 1985, Gómez 1991), whereas Zonitidae have calcareous claws (Schileyko 2003). The hook-like granules of *Gudeodiscus* and the minute, flat, or sometimes elongated or globular granules of other plectopylid genera may have similar roles but a different origin from the fixed claws of other Stylommatophora.

In some *Gudeodiscus* specimens the proximal (lower) part of the penial wall is ornamented with longitudinal folds only, but in others it has transverse and dense wrinkles (e.g. in *Gudeodiscus giardi giardi* and in one specimen of *Gudeodiscus villedaryi*). The transverse and longitudinal arrangement may result in a reticulated surface of the inner penial wall, such as those in *Gudeodiscus phlyarius* (*fallax*-like specimens). These traits need to be used for taxonomy with careful attention to collection dates and instead may provide opportunities for studies of functional roles for reproductive success for the following reason: two specimens of *Gudeodiscus villedaryi* collected in different periods of the year (20 May and 12 November) from the same locality greatly differed in these traits. The one collected in May was gravid, and its penis had only longitudinal folds on its inner wall, with slightly waved proximal portions of the folds. In contrast, a specimen collected in November was not gravid and had conspicuous, dense and transversal folds on the proximal portion of the inner wall of the penis. This transversal folded structure turned suddenly to a longitudinal folded area with calcareous claws between the pockets. This result suggests that the morphology of fine sculpture of the inner penial wall (at least inside the proximal half of the penis) may be seasonally variable. The gravid individual may have lost hooks in a mating period before collected in May. The latter individual with no embryo may have been in a period for copulation. Our observation suggests that the penial internal wall may be restructured to regenerate the hook-like calcareous claws for copulation. Further studies are necessary to test this hypothesis.

The other organs of male genitalia, penial caecum and epiphallus have generally a simpler inner surface, usually with parallel and longitudinal folds, than the penis. In smaller species it is difficult to open these very slim organs, especially the epiphallus. The longitudinal folds on the inner wall of the epiphallus of *Halongella* gen. n. species have perpendicular projections which overlap with those of the neighbouring fold. Besides this, all other species have an epiphallus with simple internal longitudinal folds. The inner wall of the penial caecum is also ornamented by longitudinal folds, which are sometimes wavy, and form hollows with the neighbouring fold. This structure is similar to the penial sculpture of *Sinicola* species. A function of these hollows would probably be to hold the small calcareous granules. In some species the sculpture of the penial caecum is more complex; *Gudeodiscus messageri raheemi* has deep sinuses with the calcareous granules. *Gudeodiscus giardi giardi* has pockets formed by two neighbouring papillae (Páll-Gergely and Asami 2014). The calcareous granule within the caecum can be elongated or globular without any characteristic shape, such as in one of the dissected *Gudeodiscus messageri raheemi* specimens, or the granules can be hook-like, similar to, but smaller than those found in the penial lumen, such as in a specimen of *Gudeodiscus pulvinaris pulvinaris* (see Páll-Gergely and Asami 2014).

Specimens that were fixed in 70% ethanol were used for this investigation. Thus, at this stage of study, we are not able to rule out a possibility that some of the granules appeared as observed because of the process of preservation. However, hook structure corresponds to pocket structure in the penial internal surface. Each hook is regularly located in a pocket in a determined orientation. Further, they exhibit a taxonomically characteristic and sophisticated shape. For these reasons, the presence of hooks and granules in the present family cannot be ascribed to an artefact during preservation.

The absence of embryos in the uterus was statistically significantly associated with the presence of calcareous granules inside the penis, within *Gudeodiscus* (p = 0.0001) and also across all the four genera (p = 0.0006) (Tables 3, 4, 5). This strongly suggests that these granules may function as a disposable male mating apparatus. These granules disappear perhaps through repeated copulation in a mating season. It could require some time to gain the granules again if they lose granules and bear offspring. Thus, for some time during the mating season, they might remain with no granules before embryos develop. If so, these would exhibit no granules or embryos. However, this was the case only in three of 34 specimens examined in this study. Our results illuminate the importance of further studies on their reproductive life history and the ecological function of these granules.

The function of the calcareous hooks and granules inside the penis are unknown, although they probably play some role as a mating apparatus as well as the non-calcareous hooks in other groups. It has been classically postulated that these may function for mechanical stimulation for mating success like other penial structures or darts (Tompa 1984; Atkinson and Atkinson 1987). However, later studies have shown that love darts are not for physical stimulation but to inject mucus which includes a substance that increases paternity by inducing reconfiguration of partner’s organs for spermatophore digestion (Koene and Chase 1998; Chase and Blanchard 2006; Kimura et al. 2014). Separately, De Winter et al. (1999) proposed that the spines on the penial wall play a role in the process of spermatophore formation in the streptaxid genus *Sinistrexcisa*. This is probably not the case in Plectopylidae, because they have the structurally distinguishable epiphallus. Their spermatophores are formed in this organ instead of the penis, and thus the structure of parallel inner folds in the epiphallus matches the morphology of spermatophore. Tompa (1984) also suggested that the penial hooks may function as mechanical holdfasts during mating. The present study provides a systematic ground for further studies on the evolution of mating apparatus inside the penis.

The function of the characteristic vaginal granules in one of the *Halongella schlumbergeri* specimens are also unknown. To our knowledge, no disposable granules have been reported in land snails which are attached to the vagina wall. The presence of vaginal granules in a non-gravid specimen and the presence of “vaginal sand” in a gravid specimen indicate that these granules are present only seasonally, probably related to the mating period. The characteristic shape of the granules, namely the flat base portion and the needle-bearing apical part does not support the hypothesis that they are artefacts formed during preservation.

To our knowledge, information on plectopylid radulae was published by Stoliczka (1871; *Plectopylis achatina*, *Plectopylis cyclaspis* and *Endothyrella pinacis*), Solem (1966; *Chersaecia simplex*) and Chang and Ookubo (1999; *Sicradiscus ishizakii*). Here we publish the radula morphology of 23 Chinese and Vietnamese species. Our limited information suggests that the relative size of the central tooth and the shape of the marginal teeth may be used in the systematics of the family. The genera *Sicradiscus*, *Sinicola* and the subgenus *Gudeodiscus* have relatively large central tooth (as large as or larger than the ectocone of the first laterals), and their marginal teeth are tricuspid with pointed cusps and deep incision between the cusps. In contrast, *Plectopylis*, *Halongella* gen. n., and Gudeodiscus (Veludiscus) subgen. n. possess smaller central tooth than the ectocone of the first lateral, and their marginals are bicuspid, or even if they are tricuspid, the innermost cusp is blunt and small, and there is a shallow incision between the inner two cusps. Stoliczka (1871) mentioned that *Endothyrella pinacis* (that time *Plectopylis pinacis*) has a larger central tooth than the two *Plectopylis* species, but did not provide a description or drawing of the marginal teeth. The description of the radula of *Chersaecia simplex* by Solem (1966) is accurate but he did not publish drawings. In that species, the central tooth is “tiny”, supposedly smaller than the ectocones of the first laterals. The ectocones of the outer marginals are “reduced and split” (= marginals are tricuspid). This information on the marginals, however, is insufficient to allow comparison with our data.

### Habitat

Plectopylid species seem to be associated with calcareous areas. Living specimens occur at the base of large limestone rocks surrounded by leaf litter and humus. Thus, they are not rock-dwelling but ground-dwelling. Most living species have reticulated sculpture on the dorsal shell side, which is often covered with soil and this may be of value in providing camouflage.

### Geographical coverage of the Vietnamese plectopylid fauna

At the beginning of the 20^th^ Century all the available information on the distribution and taxonomy of Plectopylidae came with specimens from northern and eastern part of northern Vietnam (Tonkin) (Figure 39). We were able to examine only a few newly collected northern Tonkinese samples. Therefore, our knowledge on those species reported from the northern border region of Vietnam is mainly based on museum specimens. On the other hand, we examined several newly collected samples from the eastern part of northern Vietnam (Tonkin). Almost all of these specimens were identified to hitherto known species. Most of these species were found in several localities. Thus, this study covered the taxonomic diversity of plectopylids in the eastern Tonkinese area relatively well. Plectopylid specimens from western Tonkin have been examined for the first time. This resulted in the present description of a new species and a new subspecies.

Little information on plectopylid diversity has been obtained in the lowlands of the Red River, although these areas may not provide suitable habitats for land snails that prefer limestone outcrops or mountainous areas. Molluscan fauna in the border region of Sơn La and Yên Bái Provinces (Phan Xi Păng= “Farsipan” Mountain and its vicinity) is nearly unknown, maybe due to their high abundance in the limestone-free bedrock. Humid mountain forests there, however, may provide suitable habitats for plectopylids.

The southernmost Vietnamese county where plectopylids have been recorded is Nghệ An. The southern part of Vietnam may have been less intensively studied than the northern area (Tonkin). Accordingly the southernmost distribution of the family remains undetermined.

## Supplementary Material

XML Treatment for Gudeodiscus

XML Treatment for Gudeodiscus

XML Treatment for Gudeodiscus (Gudeodiscus?) anceyi

XML Treatment for Gudeodiscus (Gudeodiscus?) cyrtochilus

XML Treatment for Gudeodiscus (Gudeodiscus) dautzenbergi

XML Treatment for Gudeodiscus (Gudeodiscus) fischeri

XML Treatment for Gudeodiscus (Gudeodiscus?) francoisi

XML Treatment for Gudeodiscus (Gudeodiscus) giardi giardi

XML Treatment for Gudeodiscus (Gudeodiscus?) hemmeni

XML Treatment for Gudeodiscus (Gudeodiscus?) infralevis

XML Treatment for Gudeodiscus (Gudeodiscus) messageri

XML Treatment for Gudeodiscus (Gudeodiscus) messageri messageri

XML Treatment for Gudeodiscus (Gudeodiscus) messageri raheemi

XML Treatment for Gudeodiscus (Gudeodiscus) phlyarius

XML Treatment for Gudeodiscus (Gudeodiscus?) suprafilaris

XML Treatment for Gudeodiscus (Gudeodiscus) villedaryi

XML Treatment for Veludiscus

XML Treatment for Gudeodiscus (Veludiscus) emigrans

XML Treatment for Gudeodiscus (Veludiscus) emigrans emigrans

XML Treatment for Gudeodiscus (Veludiscus) emigrans quadrilamellatus

XML Treatment for Halongella

XML Treatment for Halongella fruhstorferi

XML Treatment for Halongella schlumbergeri

XML Treatment for Sicradiscus

XML Treatment for Sicradiscus mansuyi

## Supplementary materials

Supplementary material 1 Exact locality data of Vietnamese Plectopylidae species. Data type: Table. Explanation note: This Excel file contains all exact locality data of Vietnamese Plectopylidae. The localities are subdivided into three columns (verbal description of the locality; latitude; longitude). This dataset is made available under the Open Database License (http://opendatacommons.org/licenses/odbl/1.0/). The Open Database License (ODbL) is a license agreement intended to allow users to freely share, modify, and use this Dataset while maintaining this same freedom for others, provided that the original source and author(s) are credited.Barna Páll-Gergely, András Hunyadi, Jonathan Ablett, Hào Văn Lương, Fred Naggs, Takahiro Asami
